# Repurposing FDA‐approved drugs to treat chemical weapon toxicities: Interactive case studies for trainees

**DOI:** 10.1002/prp2.1229

**Published:** 2024-07-04

**Authors:** Lauren M. Aleksunes, Joshua P. Gray, Jaclynn Meshanni, Jeffrey D. Laskin, Debra L. Laskin

**Affiliations:** ^1^ Department of Pharmacology and Toxicology Rutgers University, Ernest Mario School of Pharmacy Piscataway New Jersey USA; ^2^ Environmental and Occupational Health Sciences Institute Piscataway New Jersey USA; ^3^ Department of Science US Coast Guard Academy New London Connecticut USA; ^4^ Department of Environmental and Occupational Health and Justice Rutgers University, School of Public Health Piscataway New Jersey USA

**Keywords:** chemical weapon, counteract, countermeasures, curriculum, repurposing, toxicity

## Abstract

The risk of a terrorist attack in the United States has created challenges on how to effectively treat toxicities that result from exposure to chemical weapons. To address this concern, the United States has organized a trans‐agency initiative across academia, government, and industry to identify drugs to treat tissue injury resulting from exposure to chemical threat agents. We sought to develop and evaluate an interactive educational session that provides hands‐on instruction on how to repurpose FDA‐approved drugs as therapeutics to treat toxicity from exposure to chemical weapons. As part of the Rutgers Summer Undergraduate Research Fellowship program, 23 undergraduate students participated in a 2‐h session that included: (1) an overview of chemical weapon toxicities, (2) a primer on pharmacology principles, and (3) an interactive session where groups of students were provided lists of FDA‐approved drugs to evaluate potential mechanisms of action and suitability as countermeasures for four chemical weapon case scenarios. The interactive session culminated in a competition for the best grant “sales pitch.” From this interactive training, students improved their understanding of (1) the ability of chemical weapons to cause long‐term toxicities, (2) impact of route of administration and exposure scenario on drug efficacy, and (3) re‐purposing FDA‐approved drugs to treat disease from chemical weapon exposure. These findings demonstrated that an interactive training exercise can provide students with new insights into drug development for chemical threat agent toxicities.

## Graphical abstract

This interactive session explores how FDA‐approved drugs can be tested as new therapeutics to treat toxicities associated with chemical weapon injury.

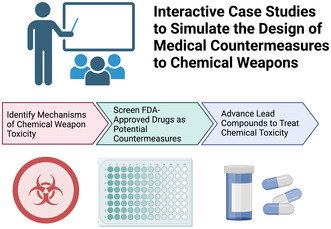

## 1 INTRODUCTION

Disaster preparedness and response are necessary to ensure the health and safety of the public. This ranges from extreme weather events and natural disasters to pandemics and terrorist threats. In particular, the release of chemical, biological, radiological, and nuclear weapons to citizens, first responders, and the military has great potential to paralyze the medical and societal infrastructure. Following the terrorist attacks at the World Trade Center and Pentagon and the dissemination of anthrax on Capitol Hill in 2001, the United States signed into law the Project BioShield of 2004. Its purpose was to accelerate the research, development, purchase, and availability of effective medical countermeasures against biological, chemical, radiological, and nuclear agents. To coordinate these efforts, the Biomedical Advanced Research Development Authority (BARDA) was created to procure and develop countermeasures with a goal of regulatory approval by the Food and Drug Administration (FDA). One of the initiatives of BARDA alongside the National Institutes of Health (NIH) was the creation of the CounterACT program as a transdisciplinary initiative involving academic centers and pharmaceutical companies to develop new and improved medical countermeasures to prevent and treat pathologies caused by chemical threat agents.^1^, ^2^, ^3^ Chemicals of high concern included vesicating or blistering agents (e.g., mustard gas, lewisite), respiratory agents (e.g., chlorine, phosgene), nerve agents (e.g., sarin, soman), cyanides, and antimuscarinic agents. The goals of various CounterACT centers across the country have largely focused on (1) advancing pharmaceuticals to treat toxicities and diseases associated with exposure to chemical weapons, (2) adding medical countermeasure drugs to the strategic national stockpile, and (3) designing curricula that train students and scientists in medical countermeasures.

Traditional training in disaster preparedness and response often centers on decontamination, triage, personal protective equipment, incident command, and disaster management; however, there has been a gap in curricula that address the development of new therapeutics to treat the toxicities resulting from chemical weapons. Developing medications to treat chemical toxicities involves the discovery of new medicines as well as the repurposing of existing FDA‐approved drugs.^1^, ^4^ The advantage of repurposing medications is the wealth of established pharmacology and safety data and the lower threshold of new data required to extend FDA approval to include additional therapeutic indications. Training in this area has critical value for students from diverse backgrounds including traditional undergraduate majors (biology, chemistry, pharmacology, and toxicology) and entry level professional healthcare programs in many fields including nursing, pharmacy, public health, and pre‐medicine.

To date, the majority of curricula developed in terror medicine and disaster response has been largely targeted to health care professionals,^5^, ^6^, ^7^ medical,^8^, ^9^ veterinary,^10^ nursing,^11^ and graduate students^12^ with few lessons and formal trainings at the undergraduate level.^13^, ^14^ In this activity, undergraduate students from biomedical, basic science, or health professions, were presented an interactive lecture on chemical toxicity and tasked with applying pharmacology concepts to review a chemical weapon exposure scenario, prioritize drugs for repurposing, and develop a sales pitch to compete for a NIH grant. Each case scenario required students to work in small groups and utilize a drug‐scoring rubric to develop a new therapeutic intervention against chemical weapon‐induced damage. Concepts in entrepreneurship and innovation were integrated into the sales pitch final presentations.^15^

The primary learning goals for this pilot activity were for students to (1) understand the regulatory infrastructure established in the United States to develop countermeasures against chemical weapon toxicities, (2) consider how route of administration, mechanism of action, storage conditions, and safety impact the therapeutic utility of a drug, (3) learn the acute and long‐term toxicities associated with chemical weapons, and (4) discuss the steps involved in repurposing a FDA‐approved drug for a new therapeutic indication.

## 2 METHODS

### 2.1 Pre‐activity preparation and assessment

In 2021, this lesson was taught to 23 undergraduate students participating in a 10‐week, full‐time in‐person summer fellowship program at our university. The students were largely rising juniors and seniors from a range of academic majors including pharmacy, chemistry, biochemistry, molecular biology, cell biology, and neuroscience. Approximately half of the students were from our university; the remainder were from colleges across the U.S. Due to the COVID‐19 pandemic, this lesson was held over Zoom although it is adaptable for in‐person instruction. The Zoom format allowed students to readily meet in small groups using breakout rooms and for instructors to navigate from group to group.

Student understanding of concepts was self‐assessed using pre‐ and post‐activity polling with three questions (Table 1). The first question focused on the toxicity of chemical weapons (learning goal 3). The second question related to the process of repurposing FDA drugs (learning goals 1 and 4). The final question focused on pharmacology principles (learning goal 2).

**TABLE 1:** Assessment questions.

Pre‐ and post‐lesson	Type of assessment	Questions^1^
Pre‐ and post‐activity	Multiple choice question 1	How would you rate your understanding of how chemical weapons cause long‐term toxicities?
		None
		Slight
		Somewhat
		Moderate
		High
Pre‐ and post‐ activity	Multiple choice question 2	How would you rate your understanding of how to re‐purpose FDA‐approved drugs for a new therapeutic indication?
		None
		Slight
		Somewhat
		Moderate
		High
Pre‐ and post‐ activity	Multiple choice question 3	How would you rate your understanding of how route of administration and exposure scenarios affect the usefulness of a drug?
		None
		Slight
		Somewhat
		Moderate
		High
Post‐activity	Overall activity assessment	How likely would you recommend this session to your colleague?
		Not at all likely
		Not very likely
		Somewhat likely
		Very likely
		Extremely likely
Post‐activity	Word cloud	Use one word to describe what you learned today

^1^ With the exception of the word cloud, student responses were treated as a 5‐point Likert scale^1^, ^2^, ^3^, ^4^, ^5^ for data analysis.

### 2.2 Didactic Instruction

The session began with a 30‐min didactic lecture with an overview of threats (biological, radiological, chemical, etc.) and regulatory steps needed for the development of countermeasures to treat the toxicities of chemical weapons (Table 2, File S1—Pharmacology Principles and CounterACT Program). This overview included a description of CounterACT as a trans‐agency collaboration with academia, pharmaceutical companies, and contract research organizations.

**TABLE 2:** Timeline of pharmacology principles and CounterACT program.

Slides	Description	Time	Details of instruction
1–2	Overview of types of threats	ca. 3 min	Examples of terrorist attacks, biological threats, and radiation threats are provided. Instructors can describe each of these events as well as others as time permits
3–5	Regulatory activities	ca. 5 min	These slides walk students through legislation and regulatory activities taken to address chemical threats
6–8	CounterACT program	ca. 5 min	Explain the overall goal of CounterACT and its organization as a trans‐agency collaboration to repurpose FDA‐approved drugs as countermeasures. There are some new molecules developed however there are larger regulatory hurdles to obtain safety data for these investigational new drugs. Repurposed drugs are advantageous as they have already completed their safety studies in animals and humans
9–10	Properties of effective drugs	ca. 5 min	Ask students to describe some properties that would make a drug desirable as a countermeasure. Answers can include targeting the mechanism of chemical toxicity, easy to administer, selective and specific with limited off‐target actions, low propensity for toxicity. This question can be done as an open Q‐and‐A, word cloud, up/down voting, etc
11–12	Pathogenesis of toxicity and disease	ca. 4 min	This is a brief overview of stages of toxicity and disease. Examples of each stage should be provided: (1) disruption of cellular process (mitochondrial injury, endoplasmic reticulum stress, adduct formation, etc), (2) disruption of cellular functions and inflammation (necrosis, apoptosis, cell membrane leakage, etc), and (3) tissue damage and symptoms (reduced pulmonary function, seizures, etc). Ask students which of these three stages is best to intervene with a drug. Answers should be focused on the first and second stages rather than treating symptoms in the third stage. This question can be done as a multiple choice, up/down vote, or drop‐pin, etc
13	Mechanisms of drug action	ca. 3 min	This slide provides examples of drug action including the ability of drugs to (1) act as agonists and/or antagonists and (2) change toxicant pharmacokinetics (i.e., metabolism)
14–15	Exposure scenarios for chemical threats	ca. 3 min	The utility of different therapeutic approaches may depend upon where they need to be administered. These slides describe four possible scenarios. Ask students about which environments would make it difficult to administer a drug that requires refrigeration for long‐term storage. It would be difficult to administer a refrigerated drug outside of a medical facility (such as the subway system or park) or on the battlefield. This question can be done as a multiple choice, up/down vote, or drop‐pin, etc
16–17	Route of drug administration	ca. 3 min	Dosage forms and routes of administration impact the usefulness of drugs under certain exposure scenarios. Administration on the skin, to the eyes and lungs and by mouth can be done practically anywhere. Ask students which route of administration would be difficult if you needed to administer the countermeasure at an indoor concert. The correct answer would be injectables. That said, there are examples (Epipen®) of injectables that are available in community settings (such as schools)

*Note*: The lesson provides an overview of chemical threat toxicities and pharmacology concepts while actively asking students questions relating to the material. The didactic portion of this activity can be delivered synchronous or as an asynchronous flipped classroom video using the S1. Pharmacology Principles and CounterACT Program Slides. This portion of the lesson is estimated to last ca. 30 min.

The activity transitioned to a discussion of the desired properties of effective drugs. This portion of the lesson included a number of open‐ended and multiple‐choice questions (Table 2, File S1). Questions included: (1) What properties does an effective drug possess (slide 10)? and (2) Which stage will be best to intervene with a drug (slide 12)? An important consideration that was highlighted was the need to develop drugs that target mechanisms involved in toxicity, rather than symptoms of disease. Similarly, the instructor discussed the importance of storage conditions for drugs and ease of administration in various settings (e.g., community, battlefield, ambulance, and hospital). This was covered with a multiple‐choice question about storage in slide 15. The route of drug administration was next discussed including the advantages and disadvantages of the various options (topical, ocular, oral, injectable). Using an example of an indoor concert and chemical weapon release, this was assessed with a multiple‐choice question on slide 17.

### 2.3 Demonstration case and student group‐based case studies

In order to prepare students to work in groups on chemical weapon scenarios, the instructor provided an overview of the activity and led an example case study (Table 3, File S2—Drug Repurposing). This demonstration case was designed for students to subsequently mirror the activities and decision‐making process of the instructor. The demonstration case opened with a discussion of how to develop grading rubrics that could be used to prioritize specific attributes of an ideal pharmaceutical. Next, the instructor reviewed a list of potential drugs that were available. This included a brief description of the mechanisms of action for the potential drugs.

**TABLE 3:** Timeline of activity overview and example case study.

Slides	Description	Time	Details of instruction
1–2	Overview of session	ca. 3 min	These slides introduce the activity to students and assign students to one of four groups
3–6	Create a drug scoring rubric	ca. 20 min	Introduce the rubric that the groups will use to score their proposed therapies. Remind the students of some of their prior answers regarding ideal drug characteristics (Table 2) including targeted mechanism of action, storage, and route of administration, safety, etc. Explain that some properties should be weighted more (i.e., assigned more points in the rubric) than others based on the group's prioritization. Prepare the students for the 1st breakout and what to expect and then turn it over to the groups for 15 min (possibly by opening Zoom breakout rooms if run online). Moderators can be used to assist the groups and keep them on task. Return the group and review your example drug scoring rubric (slide 6). Explain that groups can further tweak their rubric during their next breakout as well
7–9	Review potential drugs	ca. 15 min	Depending upon the educational level of students, the instructor can provide the completed drug tables. However, for advanced students, instructors can assign the table as pre‐work and require students to complete the table before class (i.e., route of administration, mechanism of action, major side effects). If the instructor needs to reduce in‐person class time, it is best to assign this part as pre‐work for students. Otherwise, instructors can provide the completed tables in this lesson. Students should take turns reading over the different drugs in small groups
10–13	Example case study	ca. 5 min	Read the example case study about mustard gas to the students. Highlight background information, mechanism of toxicity (Slide 11), the exposure scenario (Slide 12), and the question being posed for therapeutic intervention (Slide 13)
14–18	Using rubric for case study	ca. 25 min	Explain how students will use their table of drugs to determine which ones could be suitable for addressing the question in the case study. The instructor should explain that they selected two possible therapies from the list of drugs and that you evaluated them using the rubric. Naproxen receives high points for easy storage (room temperature), route of administration (oral), cost (generic drug), etc. However, it is not as targeted to the mechanism of mustard injury and generally aims to reduce inflammation. By comparison, anti‐TNFalpha drugs are more selective and specific in their mechanism of intervention but they are injectable drugs which are more expensive and have a greater need for storage and administration. The instructor tallies up the possible points for each therapy and shows that anti‐TNF‐alpha therapy would be the best option to move forward for repurposing to treat mustard gas lung toxicity
19–23	Sales pitch	ca. 30–35 min	Students return to the entire class and share which drug their group has selected for repurposing. The instructor should explain to the students that they will spend their next breakout session preparing their sales pitch during the final breakout. Students return from their 15‐min breakout and each group presents their case scenario and sales pitch for their planned drug to test for repurposing (ca. 3 min/group). Presentations should be succinct and hit the high points similar to a sales pitch. The entire class along with instructors and moderators then votes for the best sales pitch and team that will be awarded the NIH grant to develop their countermeasure

The instructor then transitioned to present the example case which involved sulfur mustard. Relevant background information about sulfur mustard or ‘mustard gas’ dating back to World War I was described including its multiorgan toxicity (ocular, dermal, and pulmonary). For this case demonstration, the instructor described a baseball game where over 100 people were exposed to mustard gas and developed symptoms over the prior 2 h. Key symptoms were respiratory and include shortness of breath, coughing, and painful breathing. For this scenario, the instructor needed to consider a therapeutic agent that could be administered “in the field” by emergency responders to prevent long‐term pulmonary toxicity. The instructor reminded students of the various drug options available in the case. These included drugs that were irrelevant to addressing pulmonary toxicity such as topiramate, midazolam, and other drugs that act on the central nervous system such as zolpidem and baclofen. The remaining options included dimethyl fumarate, naproxen, and antibody therapies dupilumab and adalimumab. The instructor selected naproxen and adalimumab to consider for the treatment of sulfur mustard‐induced lung toxicity. Both target inflammation although adalimumab was more specific for a key pathway involved in mustard toxicity. By comparison, naproxen targeted downstream inflammation pathways. While adalimumab was more specific in its mechanism of action, it was costly and required parenteral administration and specialized storage which reduced its score on the rubric. Naproxen was inexpensive and easy to administer although it would likely have lower efficacy in targeting the mechanism of lung pathogenesis. The instructor compared the two potential treatments and demonstrated that adalimumab has a higher score on the rubric.

The instructor next described the final component of the activity which involved a sales pitch to obtain grant funding to repurpose their medication.

### 2.4 Student group‐based case studies and final presentations

The lesson then transitioned to the group‐based case studies. Each group was assigned one of the four cases. After introducing themselves to the group, 15 min were allocated for the students to generate their rubrics. These rubrics allowed students to apply a standard set of criteria to evaluate various drug options. Groups were reminded that they could continue to revise their rubric in the next breakout. The activity then progressed to a review of potential drug therapies for 10 min. Each case included a list of potential drugs that student groups could consider for repurposing. Some of the drugs had limited utility (e.g., irrelevant mechanism of action, delayed onset, etc.), whereas others were more beneficial for symptom relief rather than targeting mechanisms of toxicant action.

After reviewing the various therapeutic options, the students were ready to review their case study. An overview of the four chemical weapon cases is provided in Table 4. Each case included (1) background information and historical context (where available), (2) mechanism of toxicity (if it is known), (3) hypothetical scenario for exposure, and (4) clinical question. The cases ranged according to chemical weapon as well as the type of organ toxicity and exposure scenario (Files S3—Case 1, Files S4—Case 2, Files S5—Case 3, Files S6—Case 4). The key organs involved include skin, lungs, and central nervous system. The exposure scenarios were broad and included terrorist attacks as well as accidental exposures. Students returned to their breakout groups for 10 min to 1 ) learn their chemical weapon, (2) review its pathogenesis, (3) select potential drugs from their list, and (4) score the top two or three drugs using the rubric they have prepared. Once groups completed ranking their potential drugs for repurposing, the students worked collaboratively for an additional 10 min on a “sales pitch” to compete for NIH grant funding. Rather than developing long presentations, groups had 3 min to provide an overview of their case and the advantages of their potential intervention. Some groups included slides whereas others did not. All students within the groups assisted in the preparation, critique, and delivery of the sales pitch to the entire class.

**TABLE 4:** Overview of chemical weapon cases.

Case	Chemical weapon	Exposure scenario	Target organ for toxicity
1	Phosgene oxime	Soldier and civilian exposure	Skin rash and intense pain
2	Tetramethylene‐disulfotetramine	Subway station	Central nervous system
3	Parathion	Disposed cylinder found in lake	Respiratory and central nervous system
4	Chlorine gas	Lab accident at home	Respiratory system

*Note*: The table lists the relevant exposure scenario and target organs described in the four case studies.

### 2.5 Activity assessment

After all groups have completed their sales pitch, attendees voted on the best sales pitch to determine which group would receive a hypothetical NIH grant to test their new intervention. These attendees included program directors, graduate students and a NIH program officer. At the end of the activity, students were asked to answer the same three self‐assessment questions from the beginning (Table 1). As this activity was conducted during a summer program, students were also asked to rate the likelihood they would recommend this activity to colleagues (Table 1). Lastly, students were also asked to describe what they learned during this session using single words (Table 1)—these words were used to assemble a word cloud. Assessment of this activity was reviewed and exempted as a secondary data collection by the Rutgers Institutional Review Board (protocol: Pro2021002542).

### 2.6 Statistical analysis

A five‐point Likert scale^1^, ^2^, ^3^, ^4^, ^5^ was applied to polling responses (none, slight, somewhat, moderate, high). Unpaired *t*‐tests (two‐tailed) were used to assess differences in responses from the pre‐ and post‐activity assessments using GraphPad Prism v10.0.3 (Boston, MA). A *p* < .05 was considered statistically significant.

### 2.7 Nomenclature of targets and ligands

Key protein targets and ligands in this article are hyperlinked to corresponding entries in http://www.guidetopharmacology.org, the common portal for data from the IUPHAR/BPS Guide to PHARMACOLOGY (Harding et al., 2018), and are permanently archived in the Concise Guide to PHARMACOLOGY 2019/20 (Alexander et al., 2019).

## 3 RESULTS AND DISCUSSION

**FIGURE 1:**
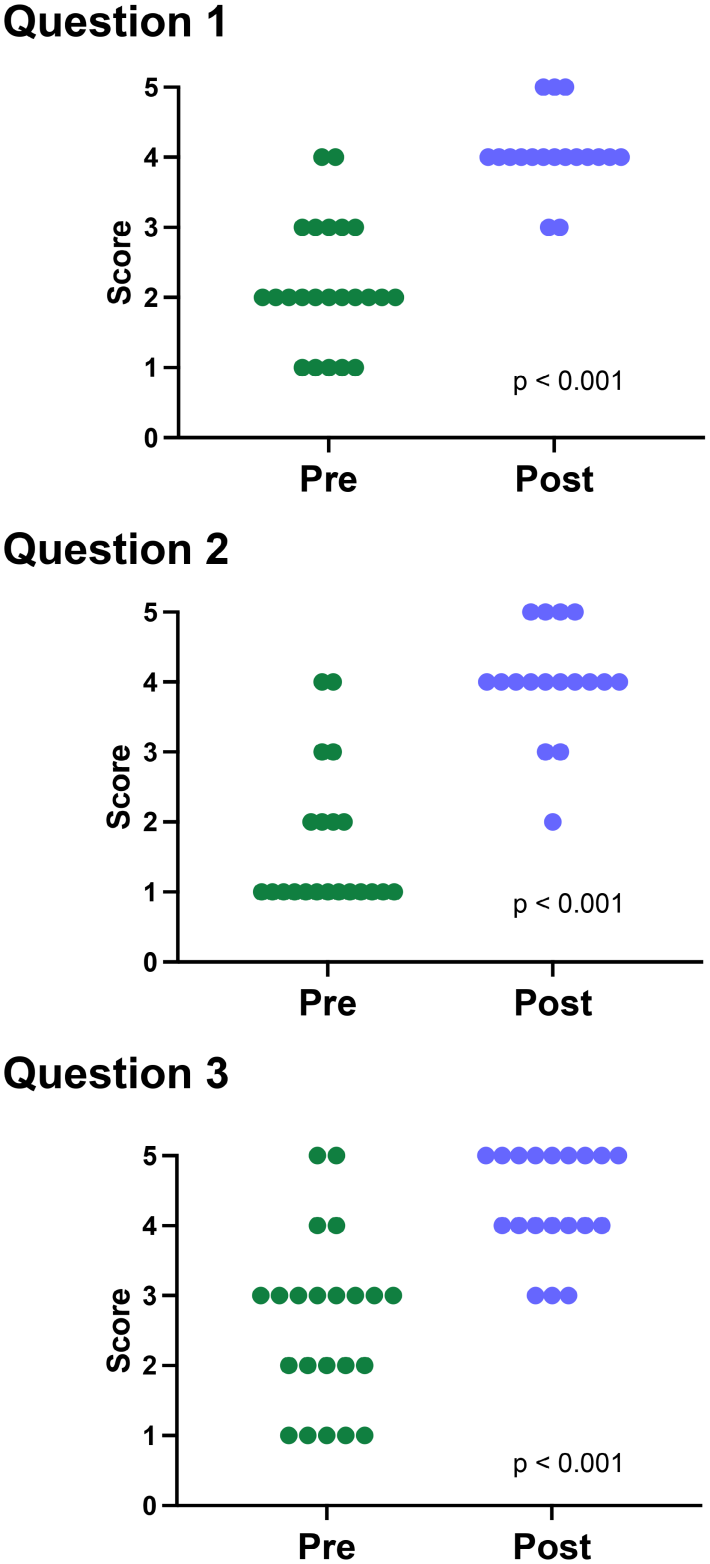
Pre‐ and post‐activity self‐assessment of participant knowledge. Students were asked three polling questions at the start and the end of the didactic and interactive sessions. Students had approximately 1 min to answer each question. Responses were converted to a 5‐point Likert scale: 1 none, 2 slight, 3 somewhat, 4 moderate, and 5 high. Each circle represents an individual respondent. Pre‐Activity: *N* = 21–23 respondents. Post‐Activity: *N* = 17–19 respondents.

We assessed student comprehension using three pre−/post‐activity multiple‐choice questions administered using PollEverywhere (Table 1). While the chemical weapon case activity was performed as a group, all pre−/post‐activity self‐assessments of understanding were completed individually. Polling questions provided students the ability to participate anonymously and assess their individual knowledge on various instructional topic.^16^ Student responses revealed that there were significant gains in all three knowledge areas (Figure 1). Prior to the activity, students largely rated their understanding of the long‐term toxicities of chemicals (Question 1), the repurposing of FDA drugs for new indications (Question 2), and the impact of route of administration and exposure scenarios on drug utility (Question 3) at “slight” which increased to “moderate” after completion of the lesson.

**FIGURE 2:**
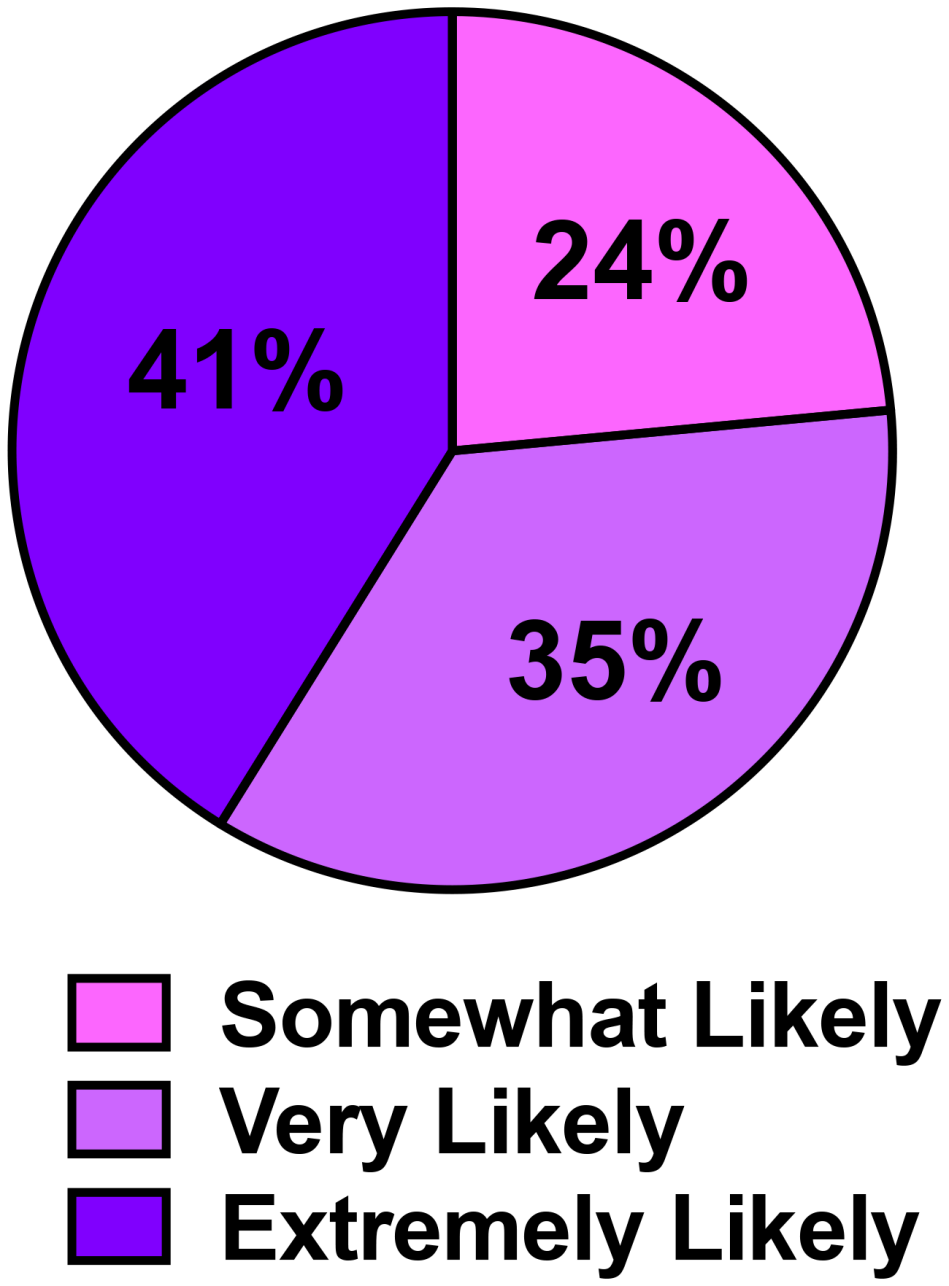
Post‐assessment of activity. Students were asked the likelihood they would recommend this activity to their colleagues (scale: extremely likely, very likely, somewhat likely, not very likely, not at all). No participants selected “not very likely” or “not at all.” *N* = 17 respondents.

**FIGURE 3:**
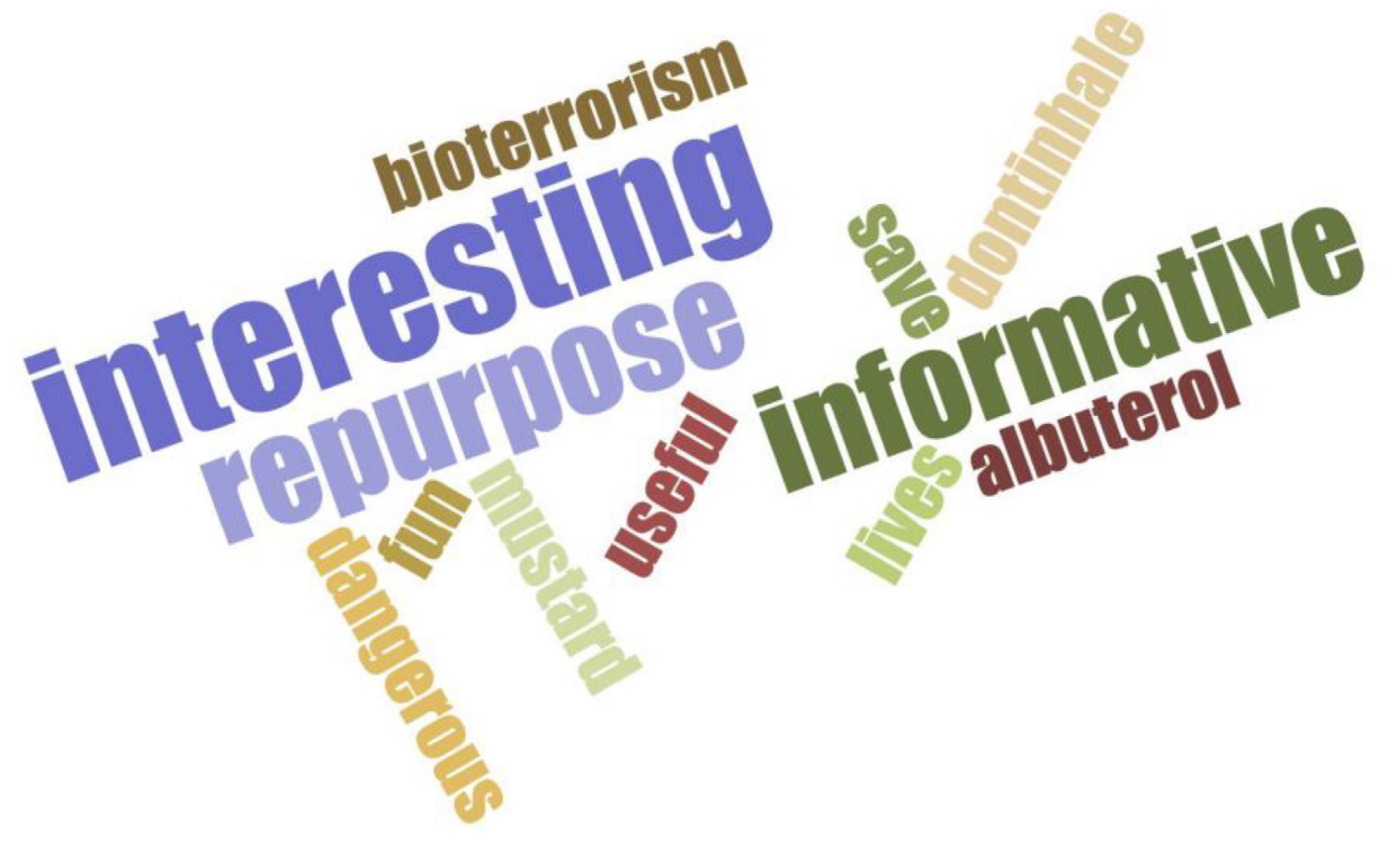
Collective student learning. Students were asked to use one word to describe what they had learned after the lesson. Answers were inputted into a word cloud generator from www.jasondavies.com. The text is a visual representation of the frequency of the words used by the participants. The more frequently a word is used, the larger the size of the text.

Following completion of the activity, students were asked their likelihood to recommend this activity to their colleagues (Figure 2). Over 75% were either “very likely” or “extremely likely” to recommend this activity. In addition, a word cloud was generated to summarize the students' reflection on the lesson and activity with one‐word answers (Figure 3). According to the word cloud, the most frequent answers were “interesting”, “repurpose” and “informative.” Other words that students reported included “bioterrorism” and “mustard.” Through the results of pre‐/post‐activity self‐assessments and the word cloud, students demonstrated an ability to advance their knowledge of chemical weapon toxicity and steps to repurpose existing drugs as novel countermeasures using hypothetical case studies.

There are multiple adaptations that can be used to customize this lesson for varying learning environments. The instructor can provide the student groups with the list of drugs and the completed tables that include clinical indication, route of administration, mechanism of action, and major side effects. Alternatively, the instructor can assign only the drug names and required student groups to look up and complete the tables prior to the start of the interactive session. The former approach can expedite the lesson; the latter approach encourages students to access and extract key information from drug references such as MicroMedex, the Physicians' Desk Reference, etc. For learners with limited backgrounds on an array of mechanisms of drug action, assigning the tables on slide 8 from each of the cases to populate the information in gray boxes can be an effective approach for self‐learning pharmacology basics before class. Additionally, if moderators or teaching assistants are available, they can assist groups within breakout rooms. Instructors or moderators may take the opportunity to assign students within the group a role while completing each step in the case. This can include assigning different students as readers or recorders within the breakout rooms or as the final presenters in the sales pitch. A set of instructions and talking points have been provided for moderators to assist each group (File S7—Moderator Instructions and Talking Points). These talking points can be used to ask questions of students and keep discussions moving forward.

Tables 2 and 3 provide an overview of the approximate time that was spent on the various lecture slides and activities to yield a 2‐h session. These serve only as a guide as there are opportunities for instructors to expand various topics that may need greater explanation based on prior student knowledge. This activity can be modified in multiple ways for use in a semester‐long project, workshop, or classroom setting. In 2022, this activity was repeated in‐person and was similarly effective as over Zoom. If there is limited time for the synchronous meetings, the didactic content can be delivered as a video using a “flipped” format. Or within a course, the didactic session can be given in one lecture period and the interactive activity performed in subsequent lecture periods. This approach can provide more free time for discussions. Likewise, the cases can be assigned to the groups to work on outside of class, researching the various drugs for potential repurposing, and presented at the next course meeting. As a semester long project, an entire session could be expanded to allow students to complete more than one case study as well as dedicate additional time and formal instruction in the development of an effective sales pitch. The sales pitch could also be expanded to the development of a NIH grant application.

Recognizing that this session was conducted as part of a summer internship, one limitation of this activity was that actual learning gains were not assessed. As a course‐based lesson, questions about the regulatory approval of new and repurposed medical countermeasures as well as principles of pharmacology and toxicology should be utilized to increase the rigor of evaluating student learning. Collectively, a combination of didactic instruction, a case study, and interactive group projects and presentations provides for a well‐rounded and novel approach to instruct students on the steps needed for the regulatory approval of medical countermeasures to chemical threats.

## AUTHOR CONTRIBUTIONS

Participated in research design: LA, JG, JM, JD, DL. Conducted analysis: LA, JM. Wrote or contributed to the writing of the manuscript: all authors.

## CONFLICT OF INTEREST STATEMENT

None of the authors have a financial, personal, or professional conflict of interest related to this work.

## ETHICS STATEMENT

Assessment of this activity was reviewed and exempted as a secondary data collection by the Rutgers Institutional Review Board.

## Supporting information


File S1.

File S2.

File S3.

File S7.

## Data Availability

The data that support the findings of this study are available from the corresponding author upon reasonable request.
