# Supplementary material for: Repurposing FDA‐approved drugs to treat chemical weapon toxicities: Interactive case studies for trainees
Source: Pharmacol Res Perspect. 2024 Jul 4;12(4):e1229. doi: 10.1002/prp2.1229 (PMC11223991; doi:10.1002/prp2.1229)
Supplement: Supplementary file 1 — File S1. [file PRP2-12-e1229-s003.pptx]

## Slide 1
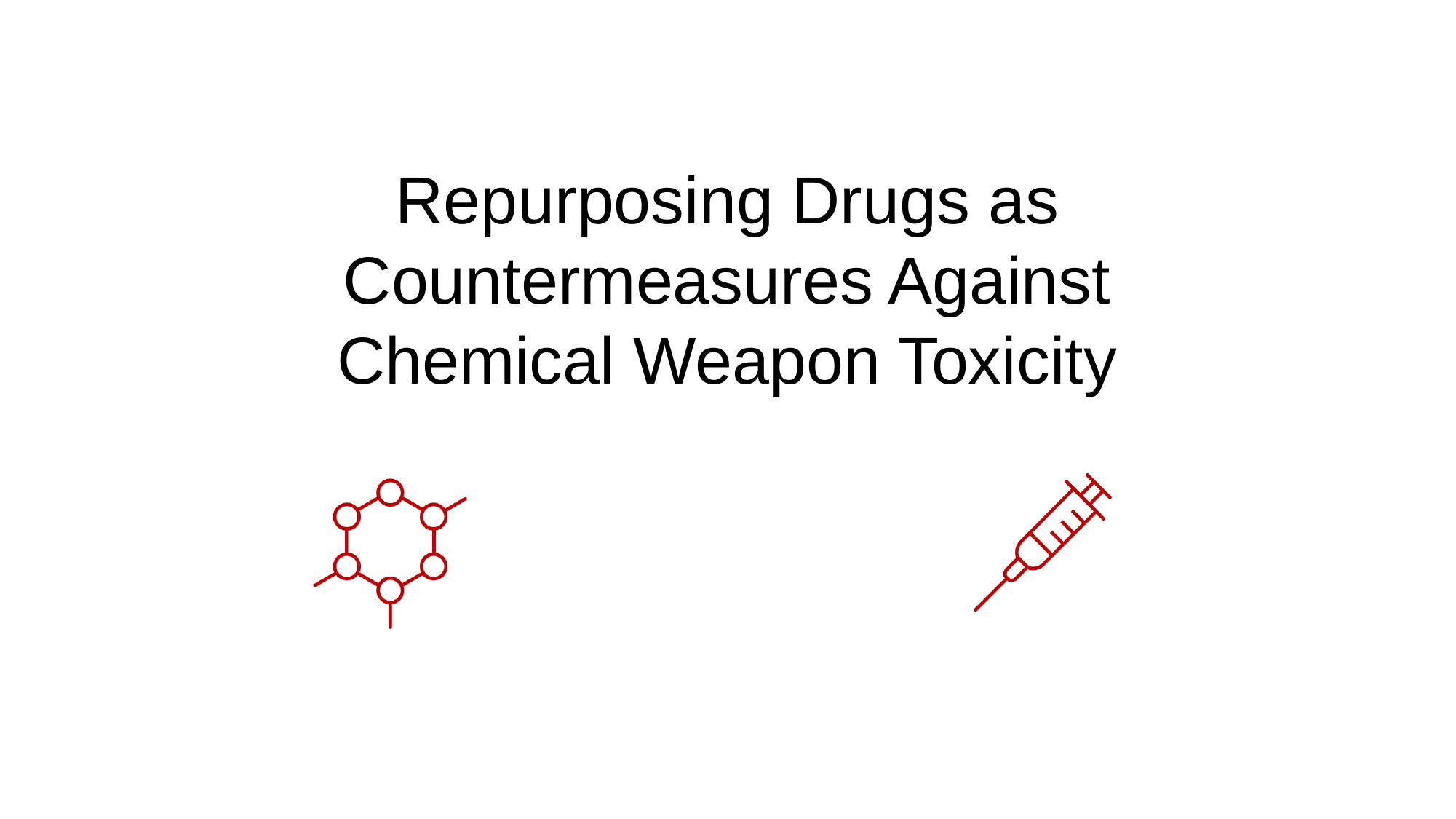

Repurposing Drugs as Countermeasures Against Chemical Weapon Toxicity

## Slide 2
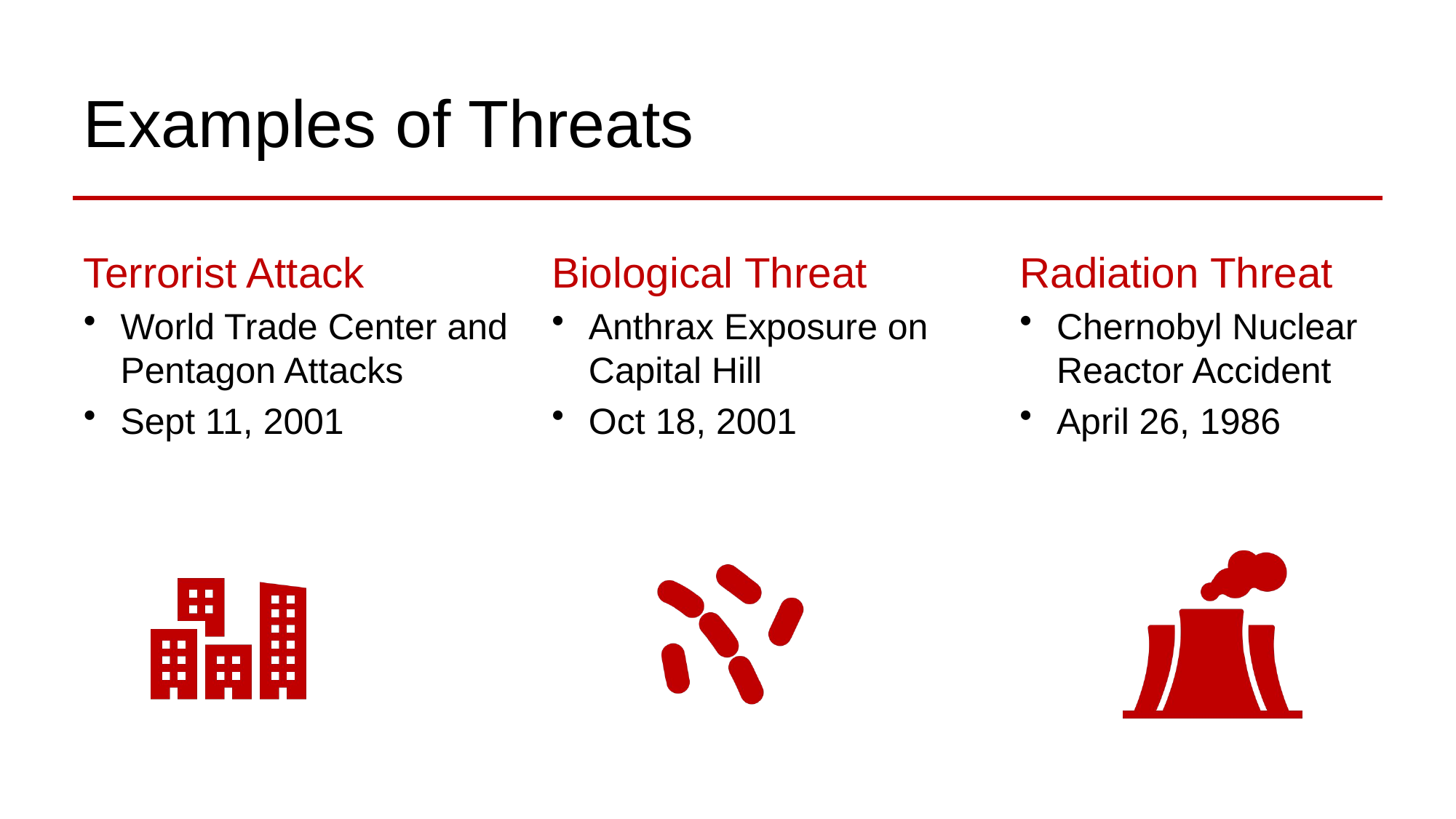

# Examples of Threats
Terrorist Attack
World Trade Center and Pentagon Attacks
Sept 11, 2001
Biological Threat
Anthrax Exposure on Capital Hill
Oct 18, 2001
Radiation Threat
Chernobyl Nuclear Reactor Accident
April 26, 1986

## Slide 3
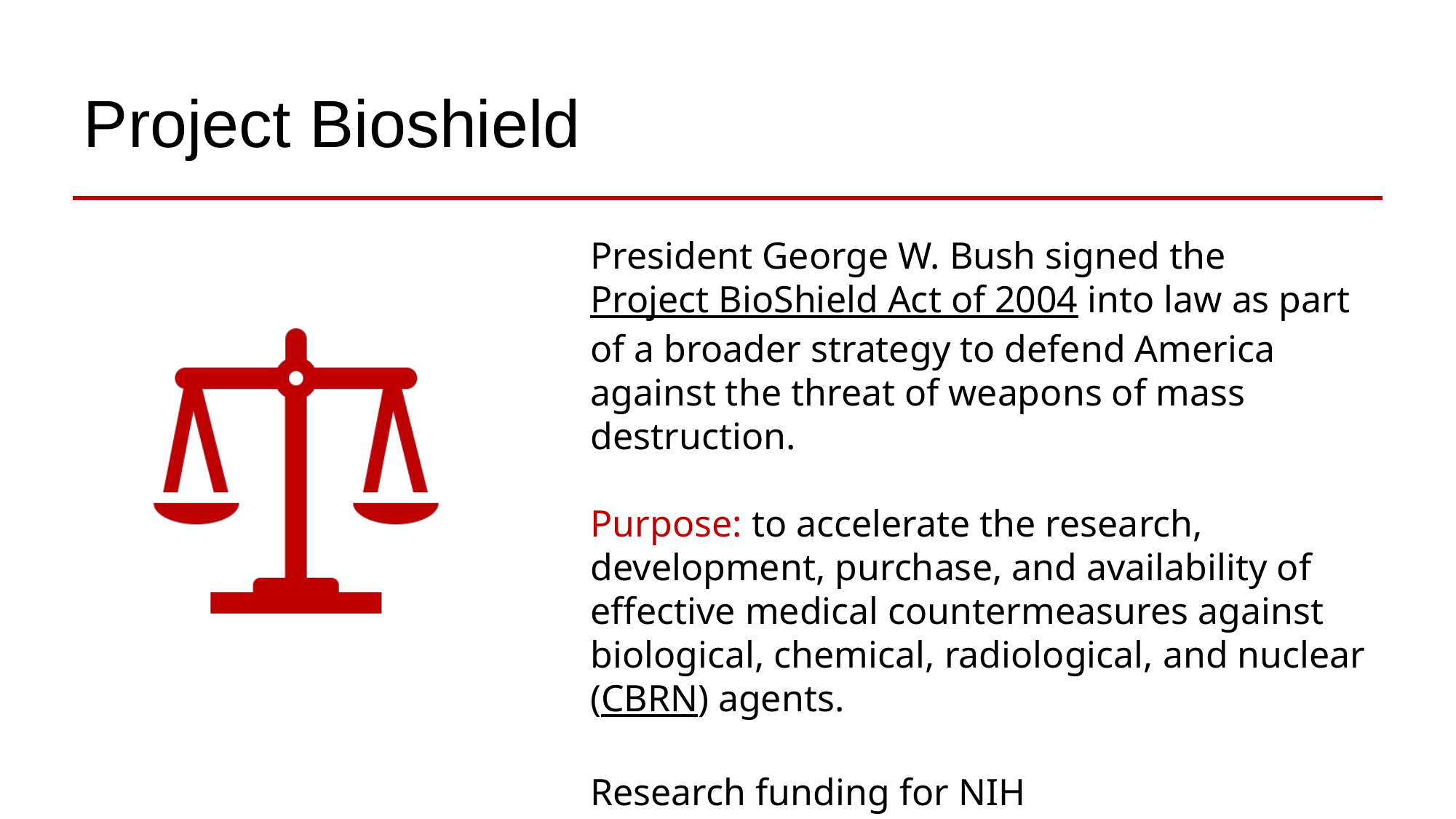

# Project Bioshield
President George W. Bush signed the Project BioShield Act of 2004 into law as part of a broader strategy to defend America against the threat of weapons of mass destruction.
Purpose: to accelerate the research, development, purchase, and availability of effective medical countermeasures against biological, chemical, radiological, and nuclear (CBRN) agents.
Research funding for NIH

## Slide 4
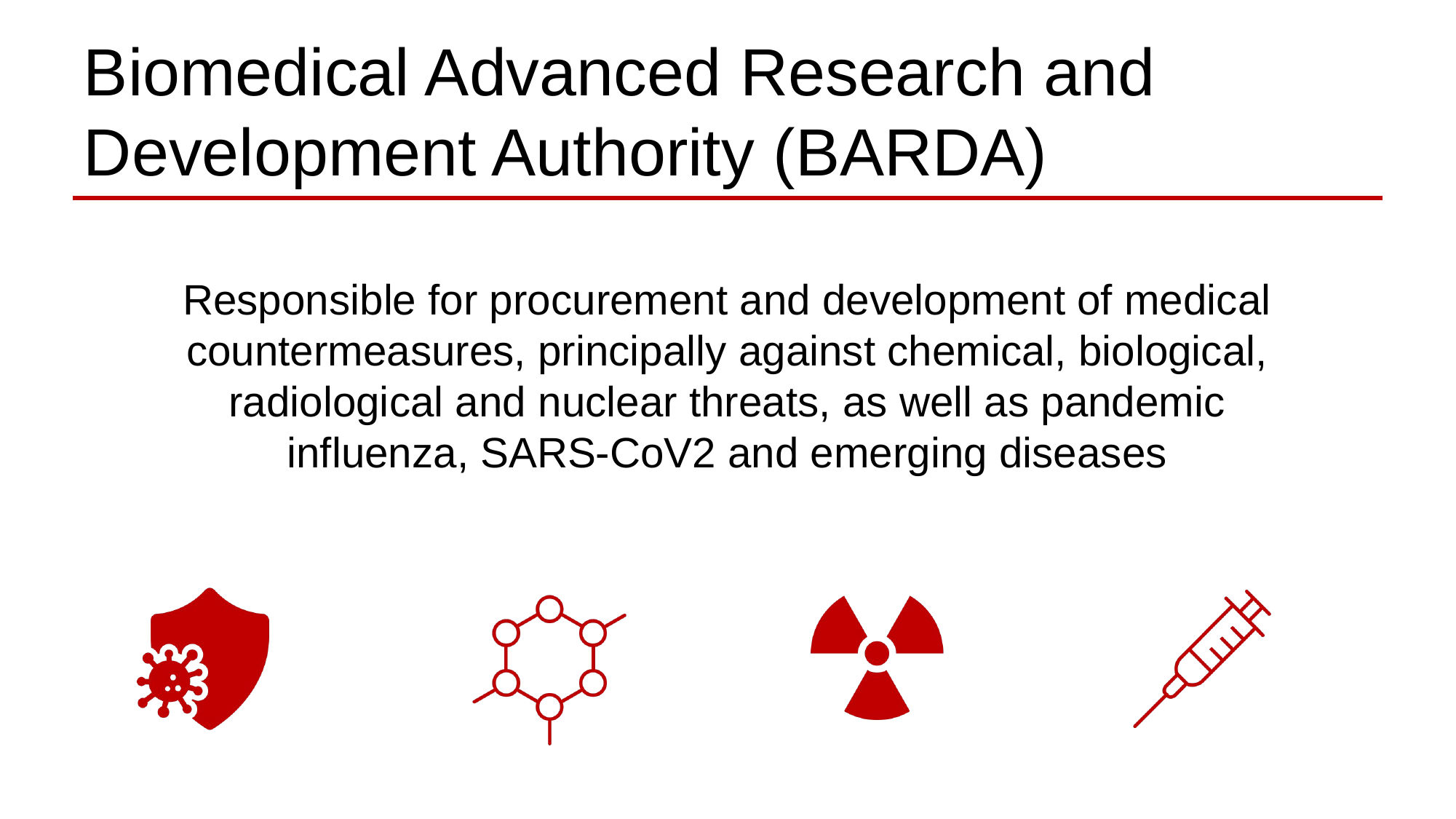

Biomedical Advanced Research and Development Authority (BARDA)
Responsible for procurement and development of medical countermeasures, principally against chemical, biological, radiological and nuclear threats, as well as pandemic influenza, SARS-CoV2 and emerging diseases

## Slide 5
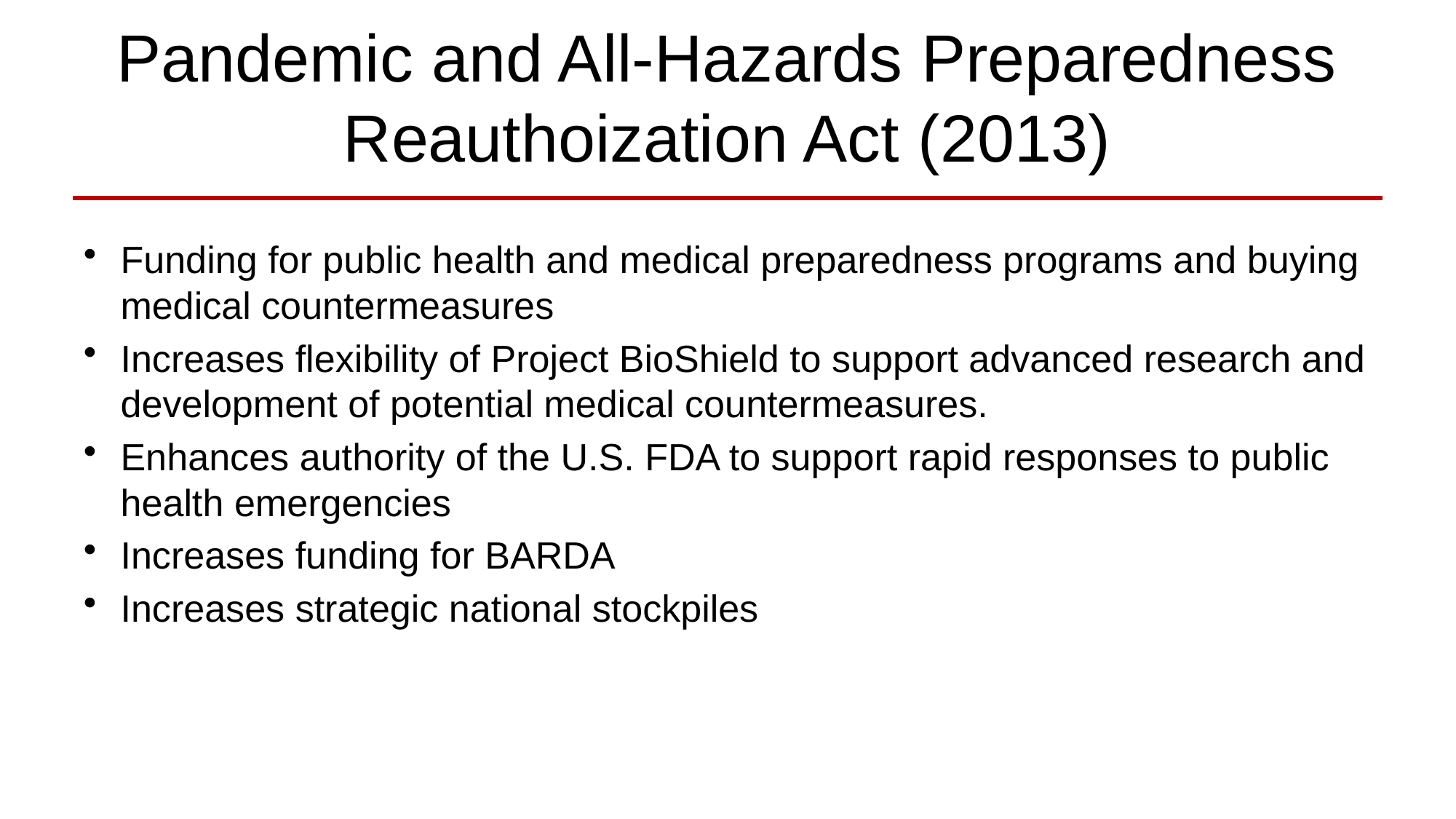

# Pandemic and All-Hazards Preparedness Reauthoization Act (2013)
Funding for public health and medical preparedness programs and buying medical countermeasures
Increases flexibility of Project BioShield to support advanced research and development of potential medical countermeasures.
Enhances authority of the U.S. FDA to support rapid responses to public health emergencies
Increases funding for BARDA
Increases strategic national stockpiles

## Slide 6
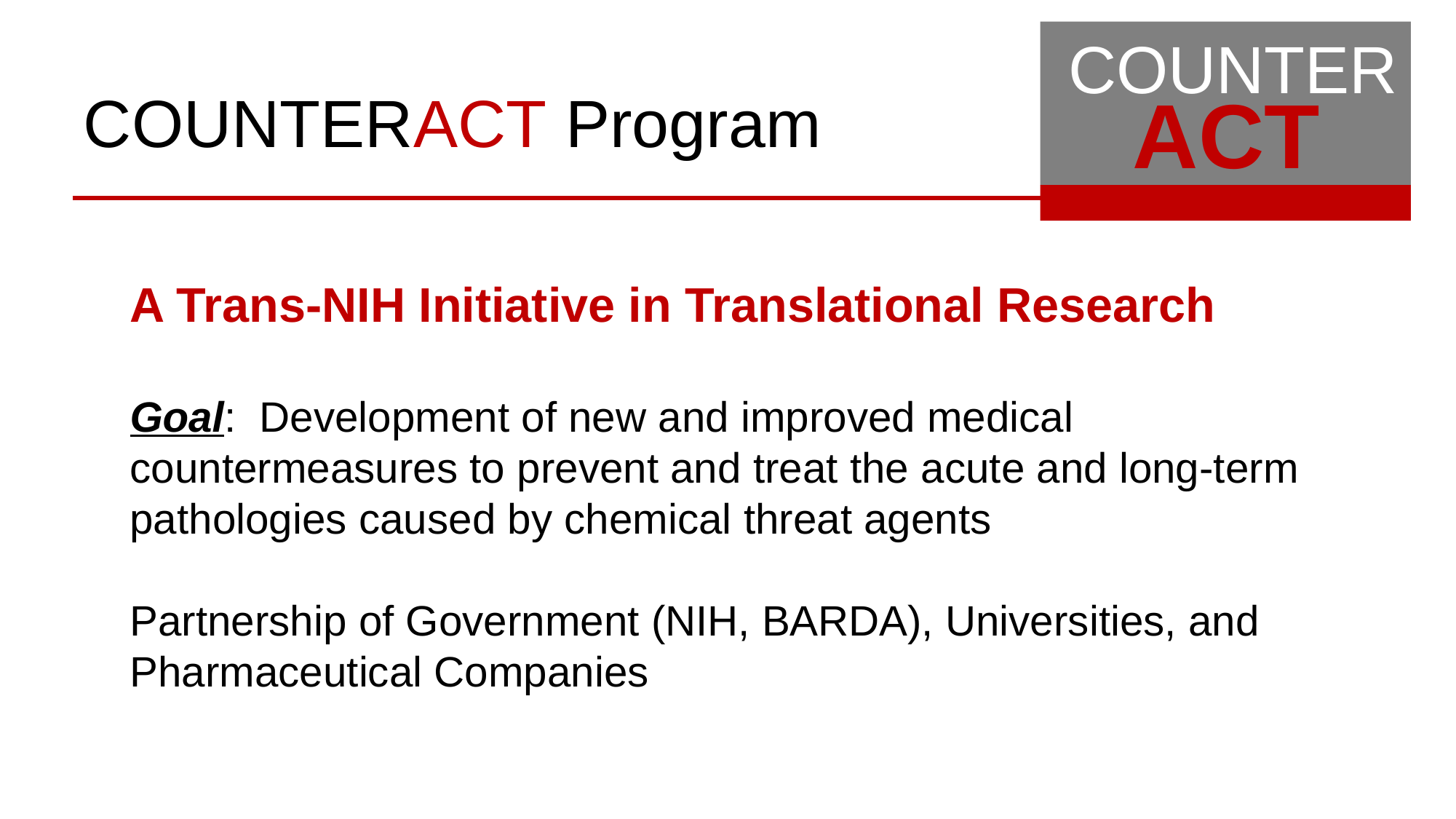

COUNTER
ACT
# COUNTERACT Program
A Trans-NIH Initiative in Translational Research
Goal: Development of new and improved medical countermeasures to prevent and treat the acute and long-term pathologies caused by chemical threat agents
Partnership of Government (NIH, BARDA), Universities, and Pharmaceutical Companies

## Slide 7
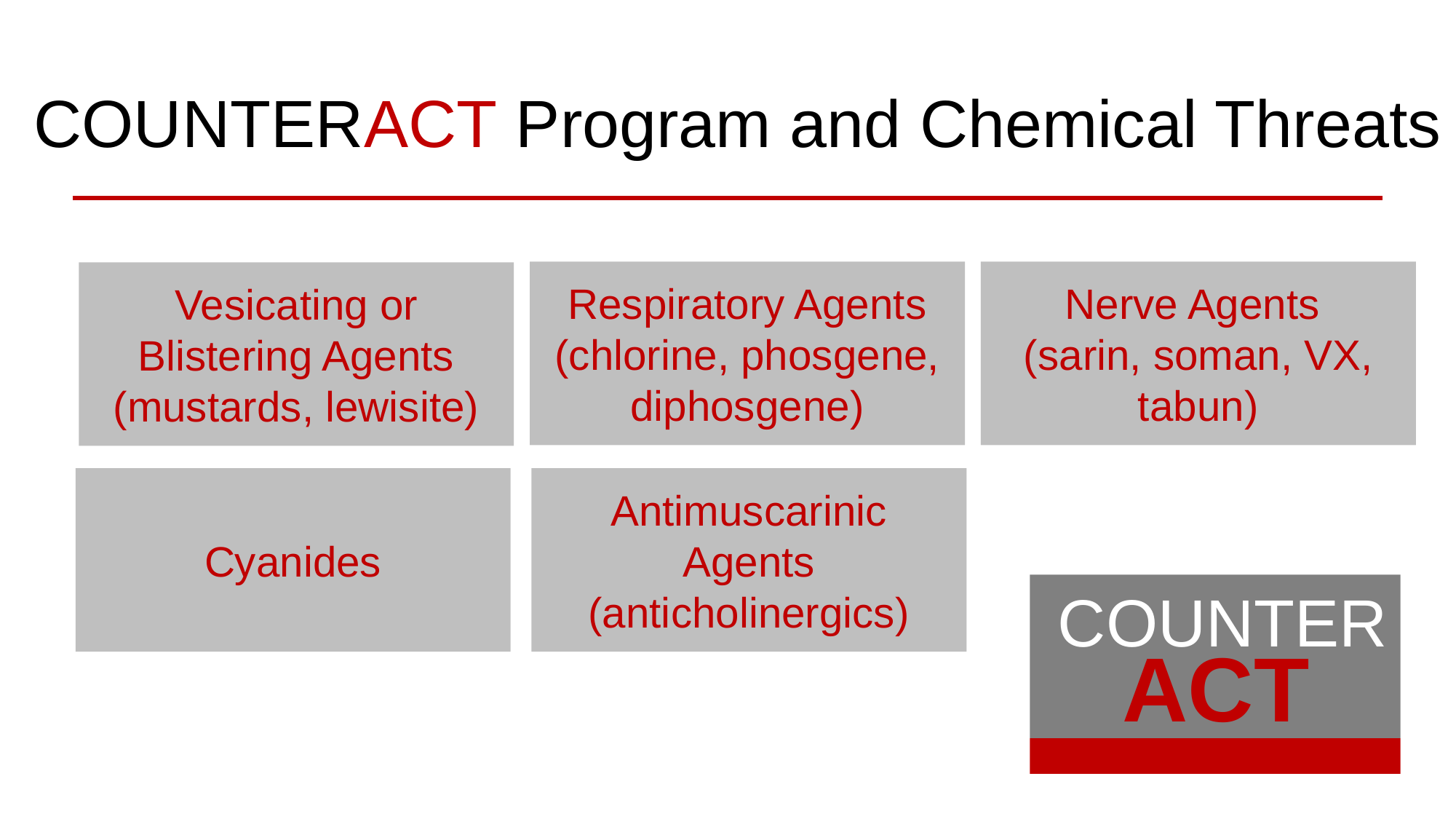

COUNTERACT Program and Chemical Threats
Respiratory Agents (chlorine, phosgene, diphosgene)
Nerve Agents
(sarin, soman, VX, tabun)
Vesicating or Blistering Agents (mustards, lewisite)
Cyanides
Antimuscarinic Agents (anticholinergics)
COUNTER
ACT

## Slide 8
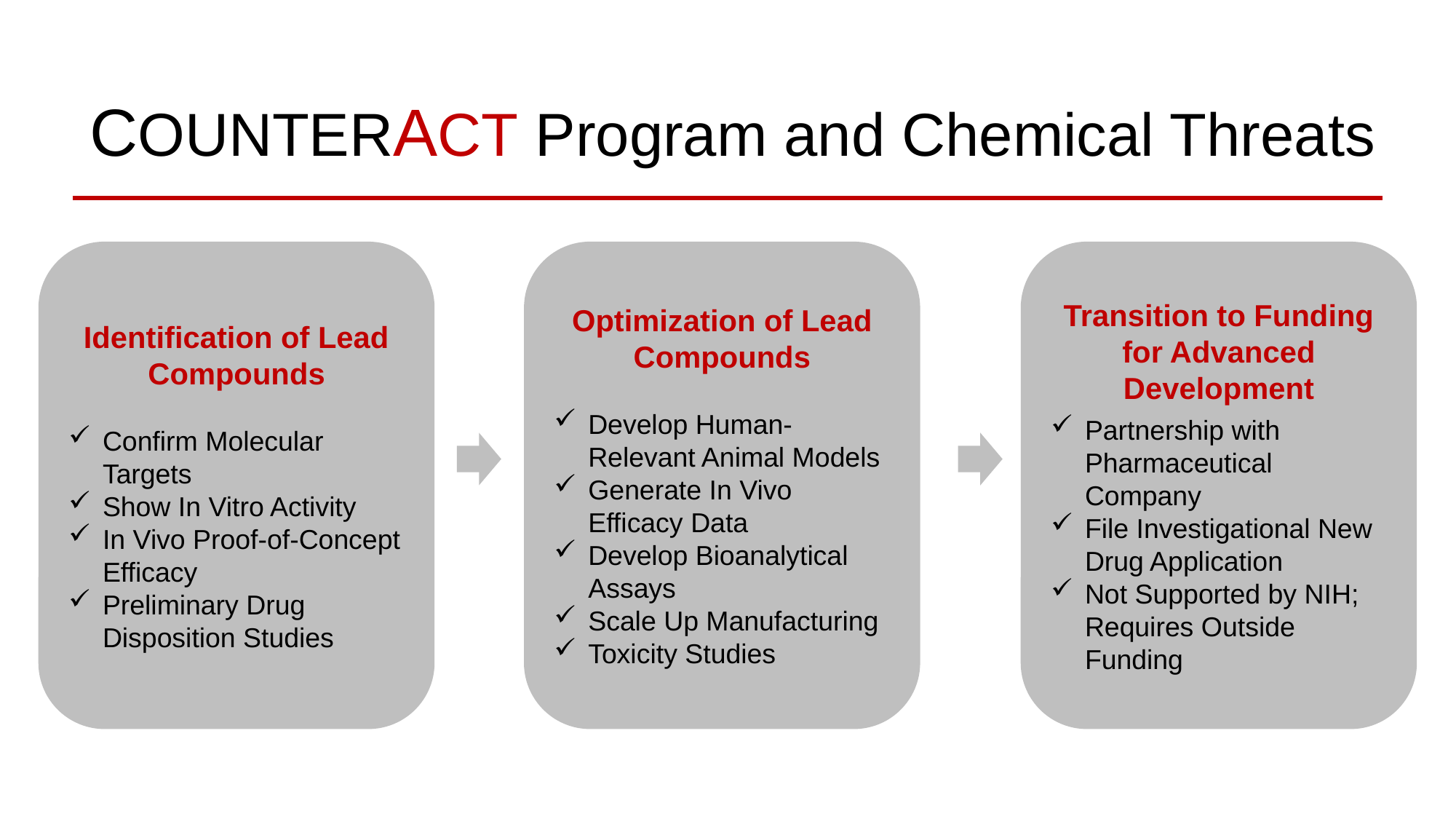

COUNTERACT Program and Chemical Threats
Identification of Lead Compounds
Confirm Molecular Targets
Show In Vitro Activity
In Vivo Proof-of-Concept Efficacy
Preliminary Drug Disposition Studies
Transition to Funding for Advanced Development
Partnership with Pharmaceutical Company
File Investigational New Drug Application
Not Supported by NIH; Requires Outside Funding
Optimization of Lead Compounds
Develop Human-Relevant Animal Models
Generate In Vivo Efficacy Data
Develop Bioanalytical Assays
Scale Up Manufacturing
Toxicity Studies

## Slide 9
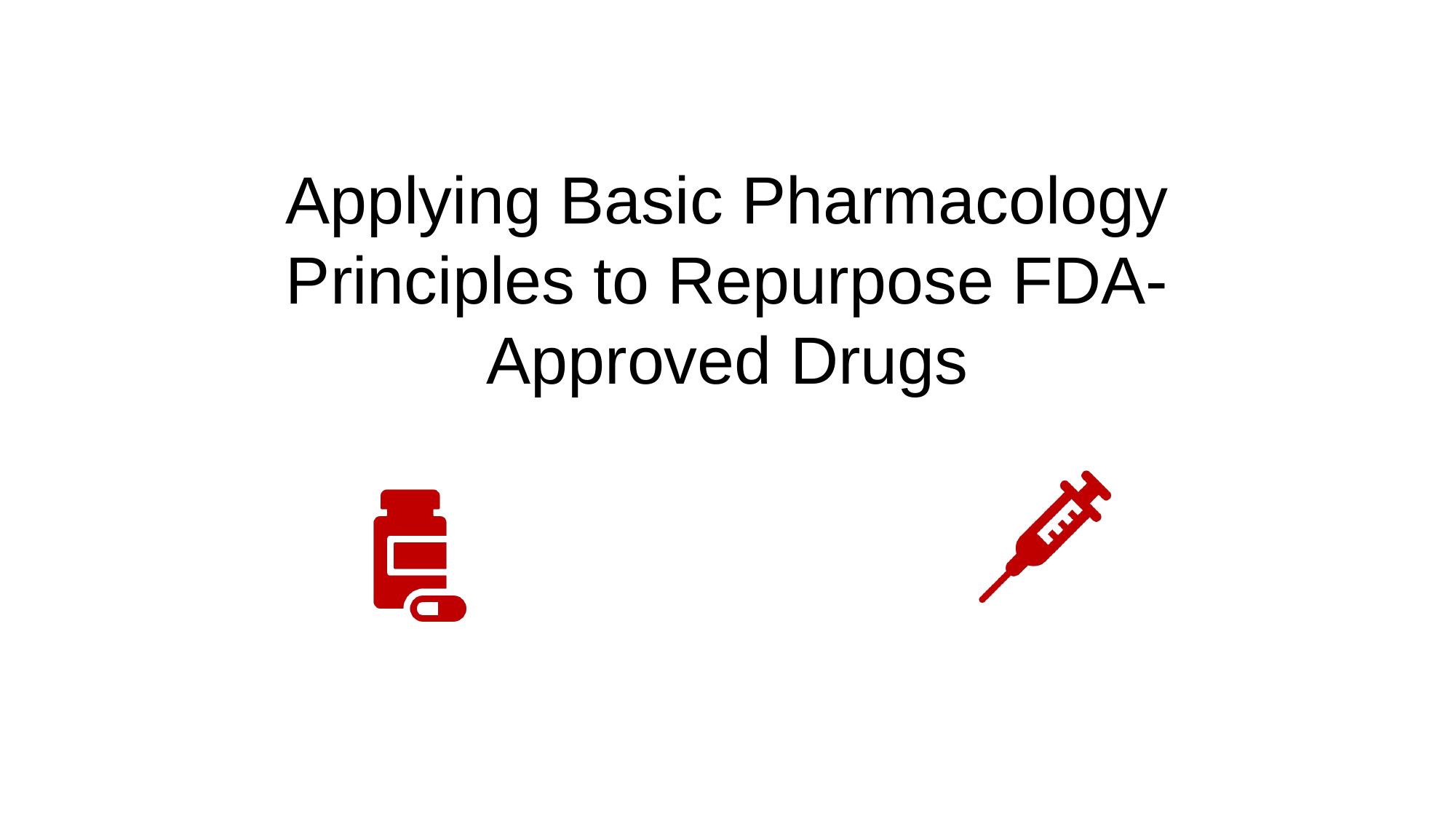

Applying Basic Pharmacology Principles to Repurpose FDA-Approved Drugs

## Slide 10
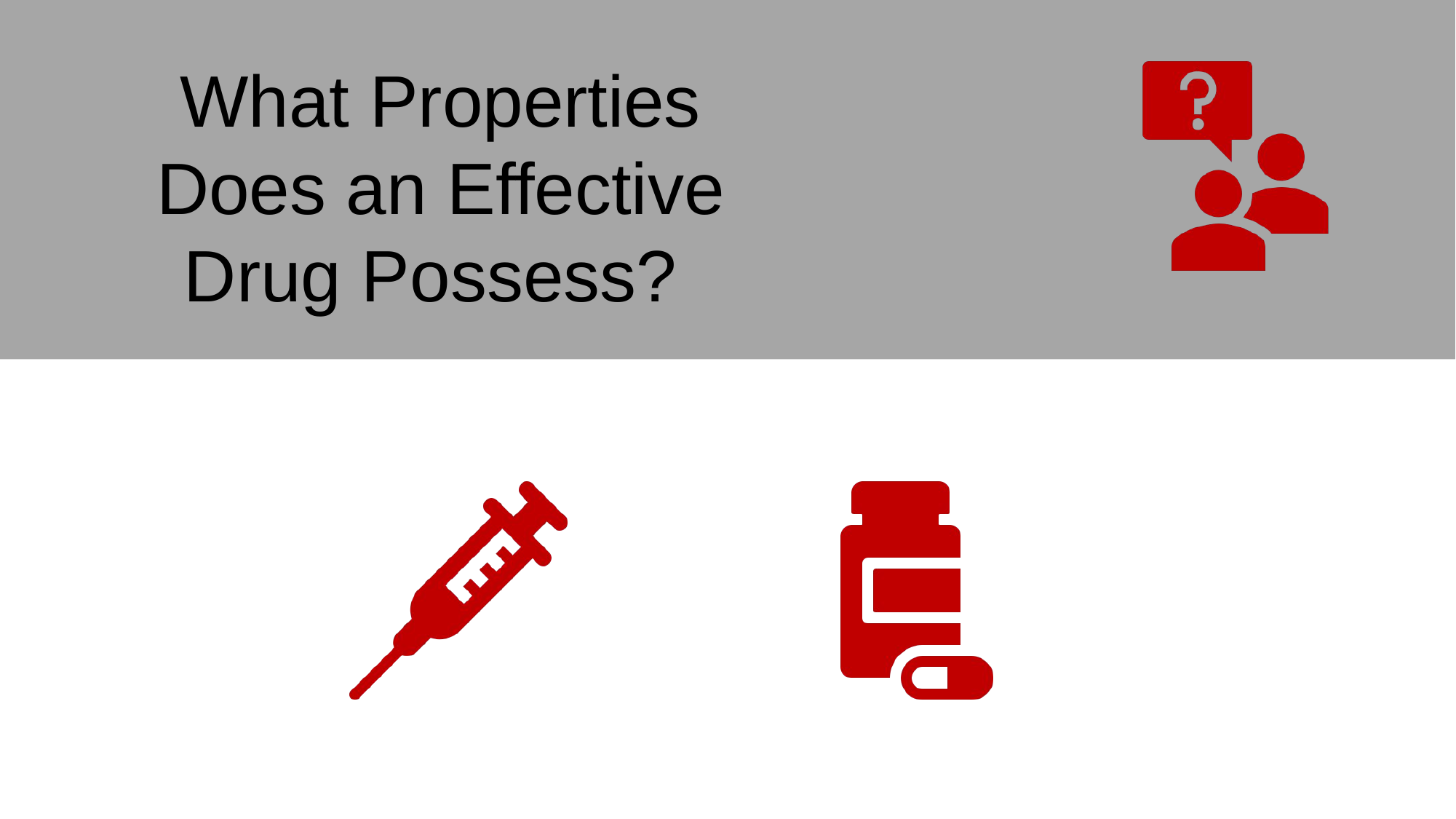

# What Properties Does an Effective Drug Possess?

## Slide 11
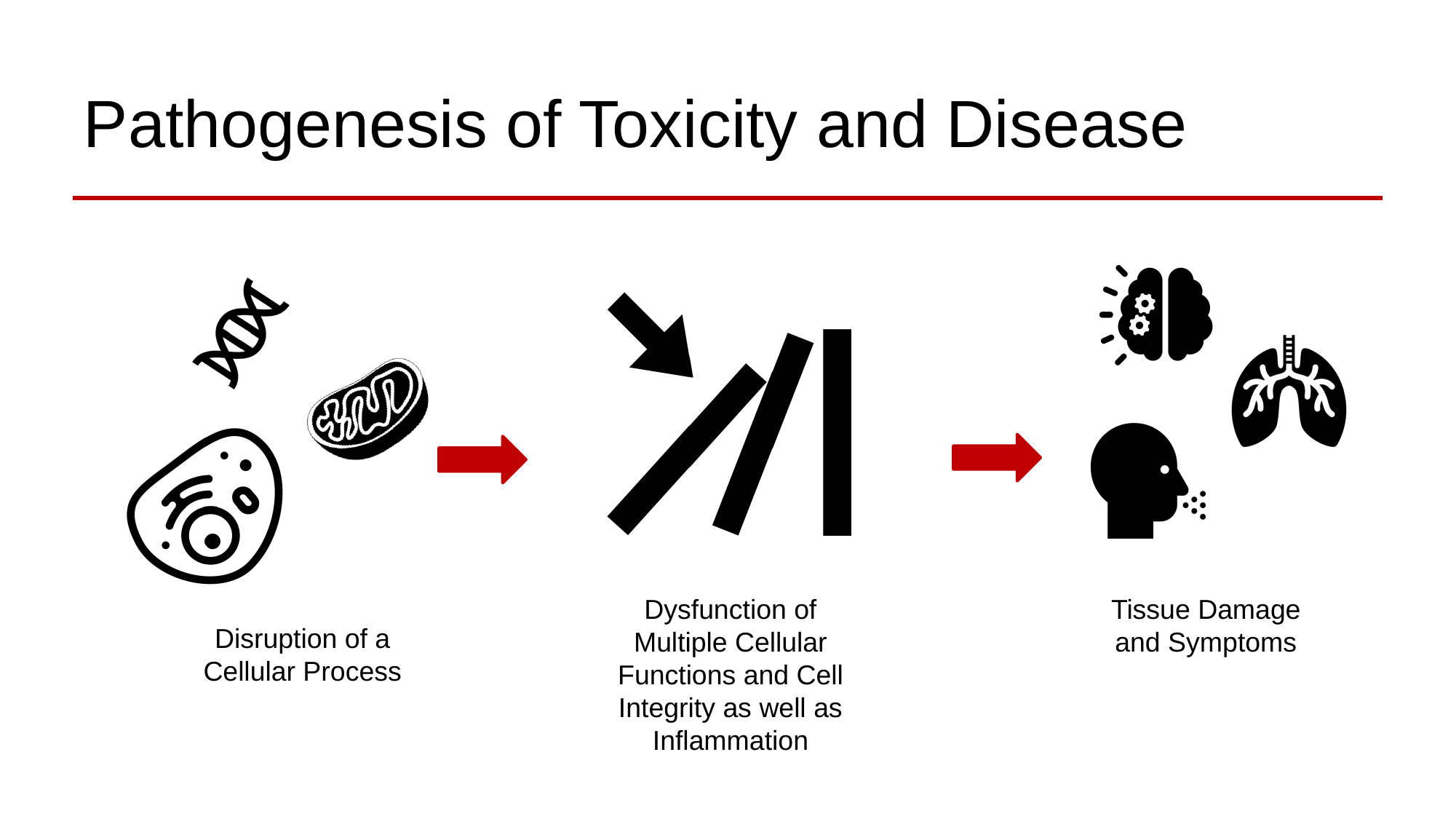

# Pathogenesis of Toxicity and Disease
Dysfunction of Multiple Cellular Functions and Cell Integrity as well as Inflammation
Tissue Damage and Symptoms
Disruption of a Cellular Process

## Slide 12
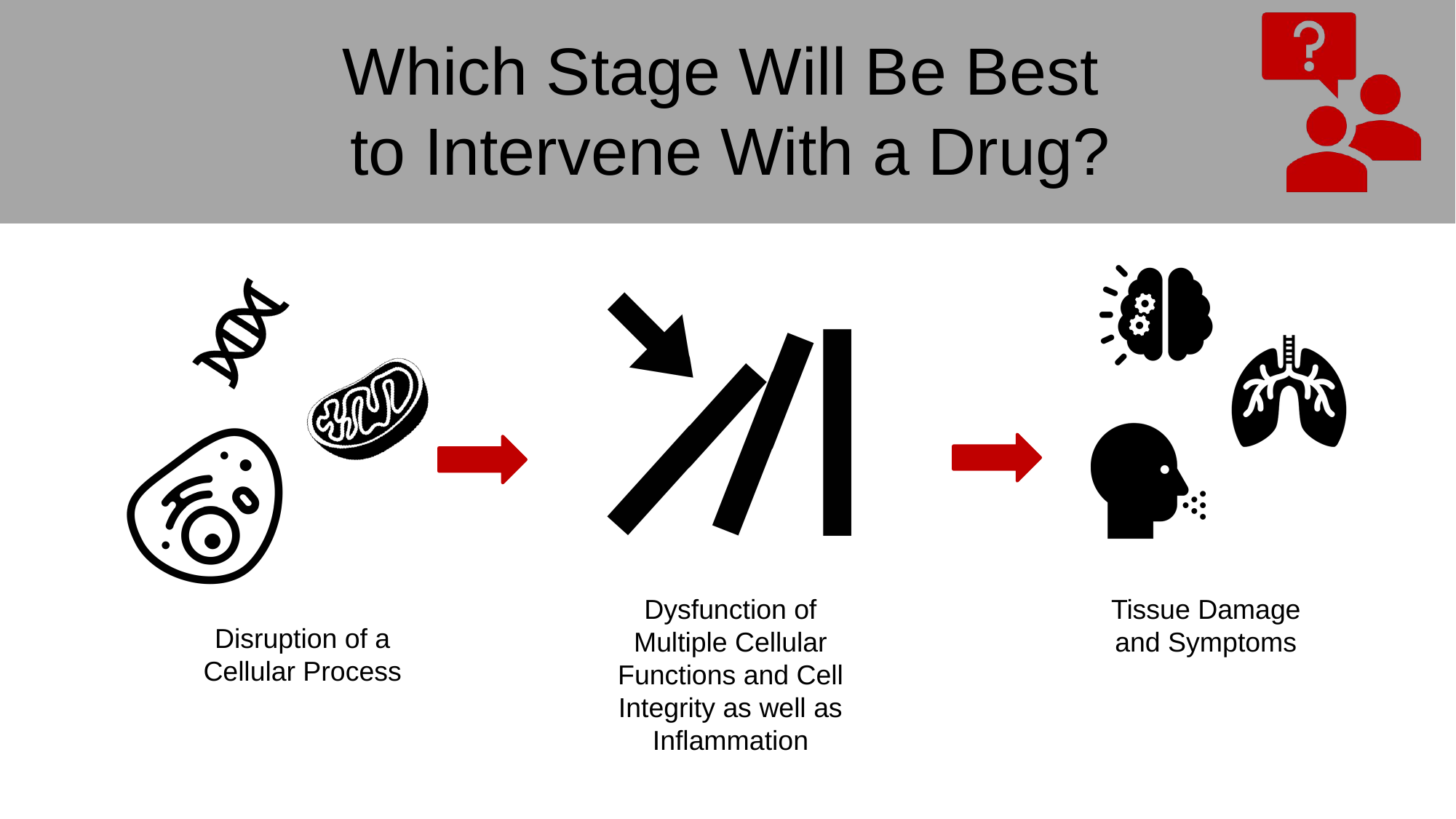

# Which Stage Will Be Best to Intervene With a Drug?
Dysfunction of Multiple Cellular Functions and Cell Integrity as well as Inflammation
Tissue Damage and Symptoms
Disruption of a Cellular Process

## Slide 13
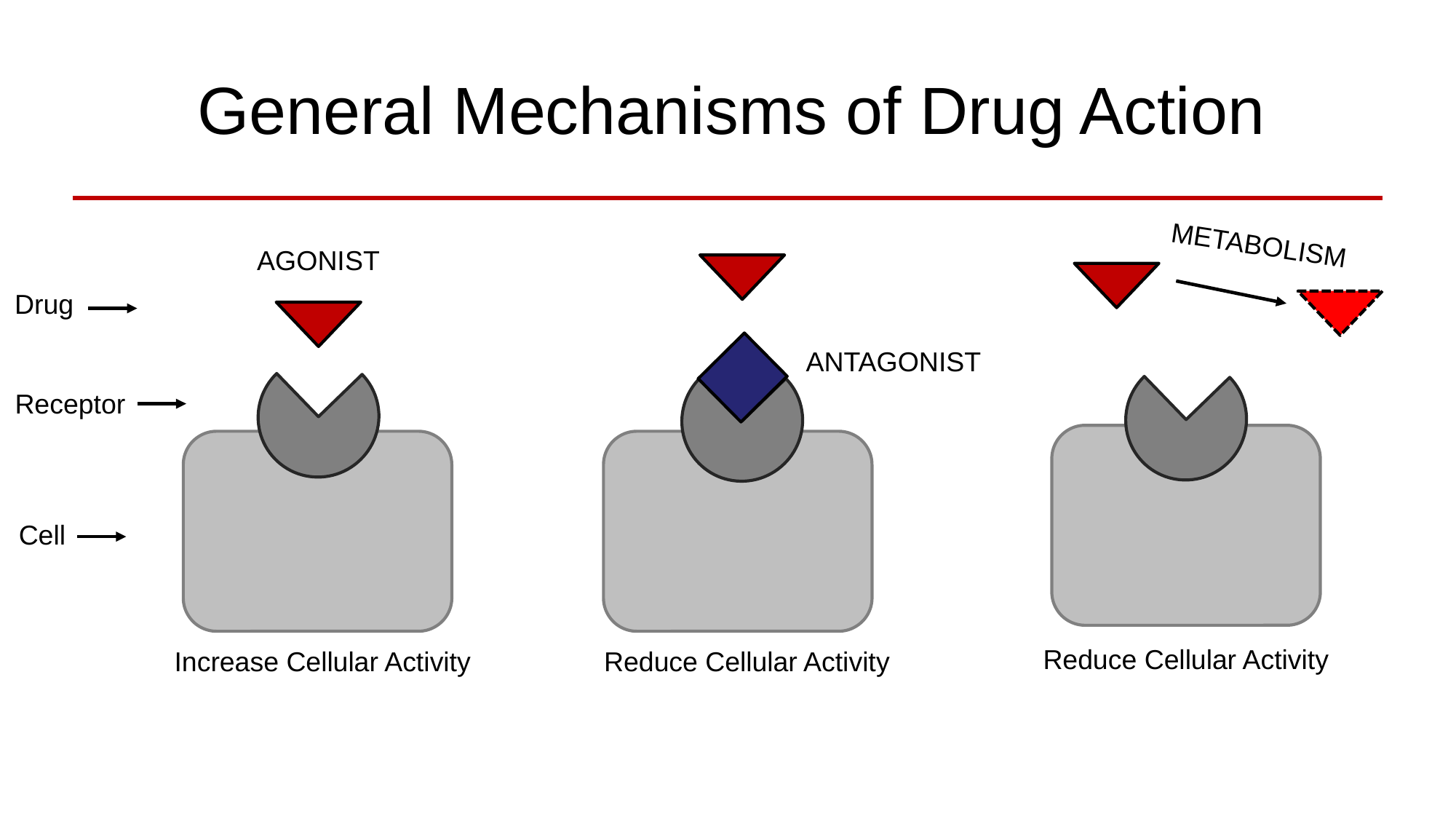

# General Mechanisms of Drug Action
METABOLISM
AGONIST
Drug
ANTAGONIST
Receptor
Cell
Reduce Cellular Activity
Increase Cellular Activity
Reduce Cellular Activity

## Slide 14
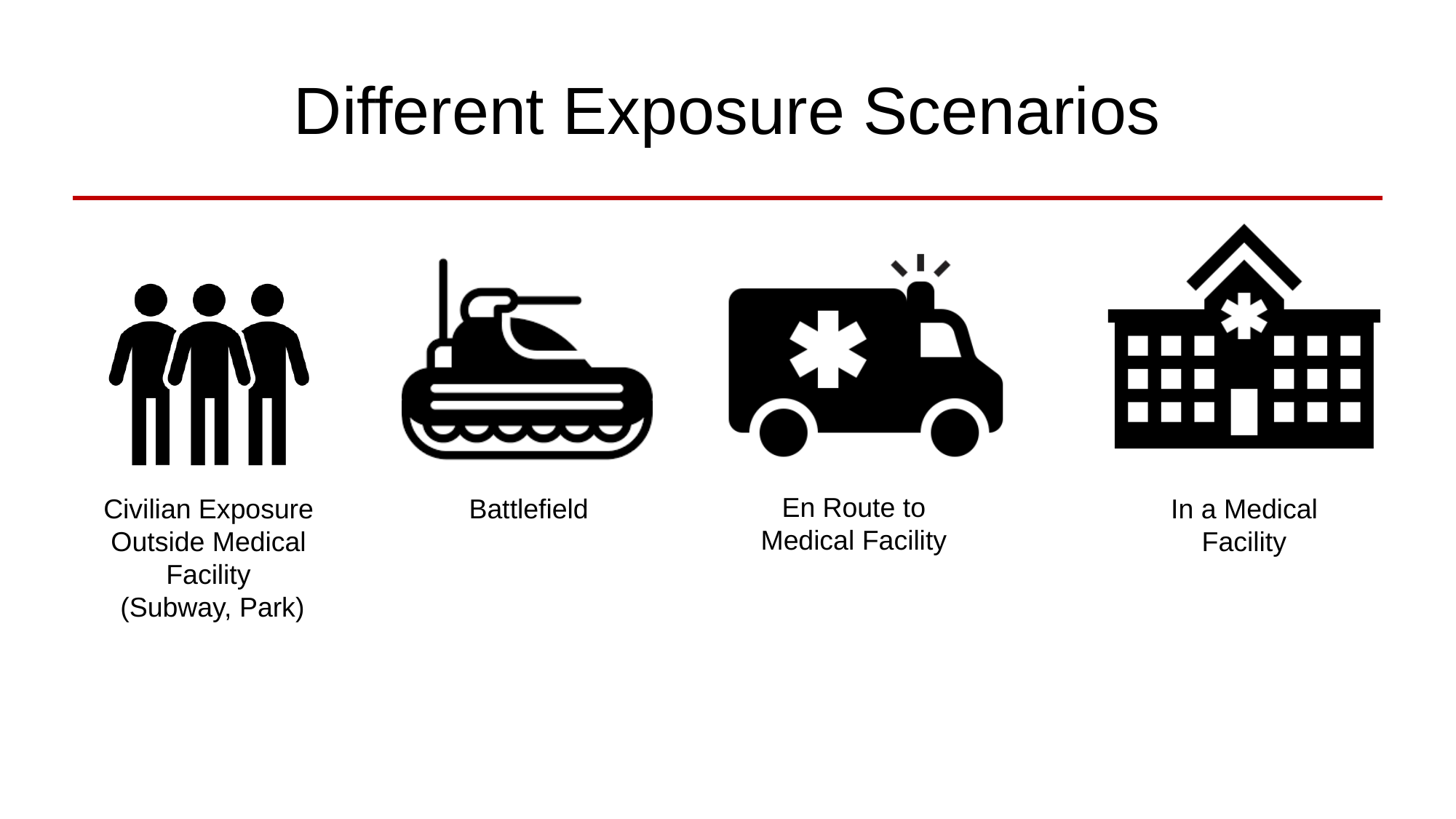

# Different Exposure Scenarios
En Route to Medical Facility
Civilian Exposure Outside Medical Facility
 (Subway, Park)
Battlefield
In a Medical Facility

## Slide 15
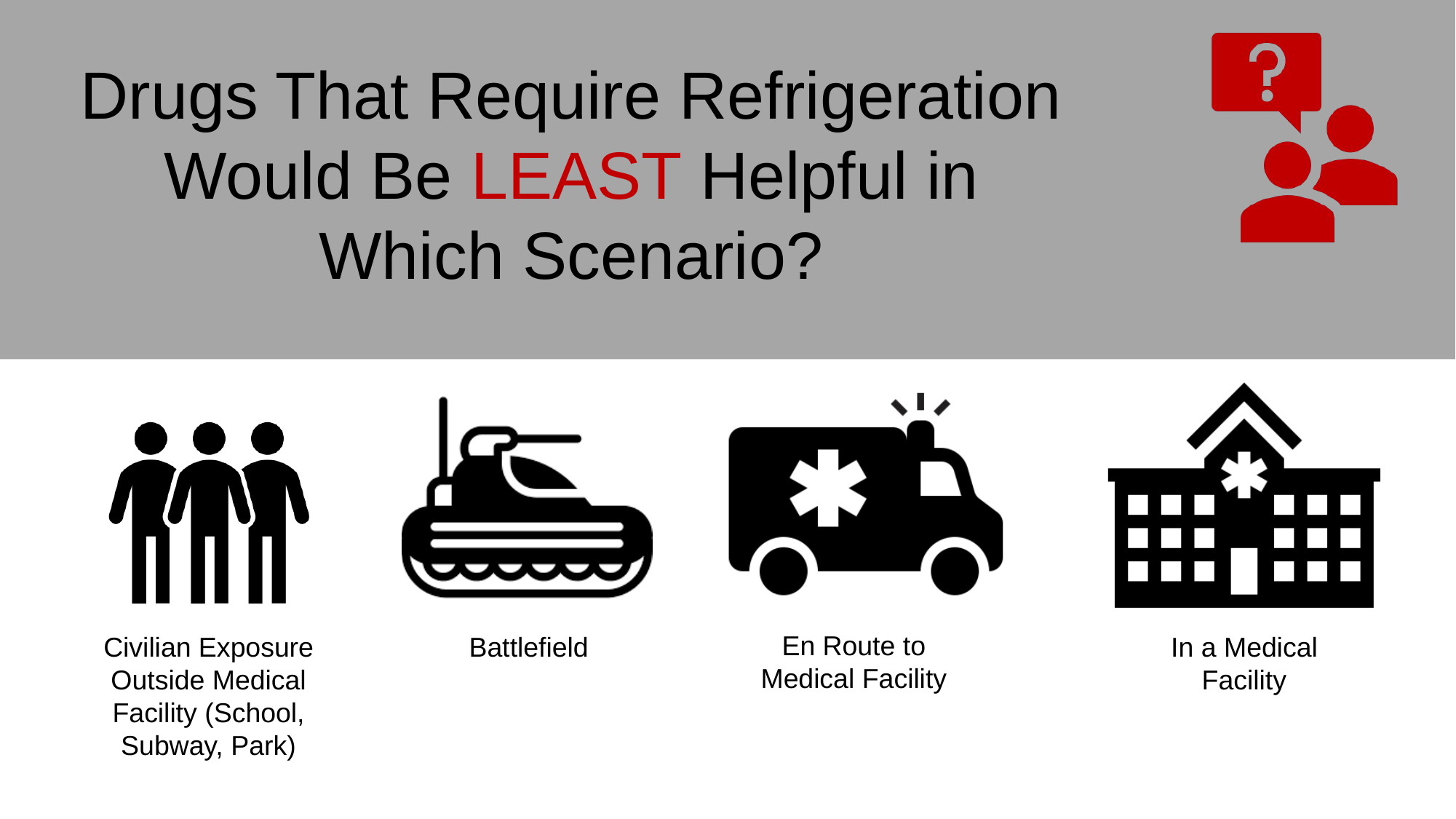

# Drugs That Require Refrigeration Would Be LEAST Helpful in Which Scenario?
En Route to Medical Facility
Civilian Exposure Outside Medical Facility (School, Subway, Park)
Battlefield
In a Medical Facility

## Slide 16
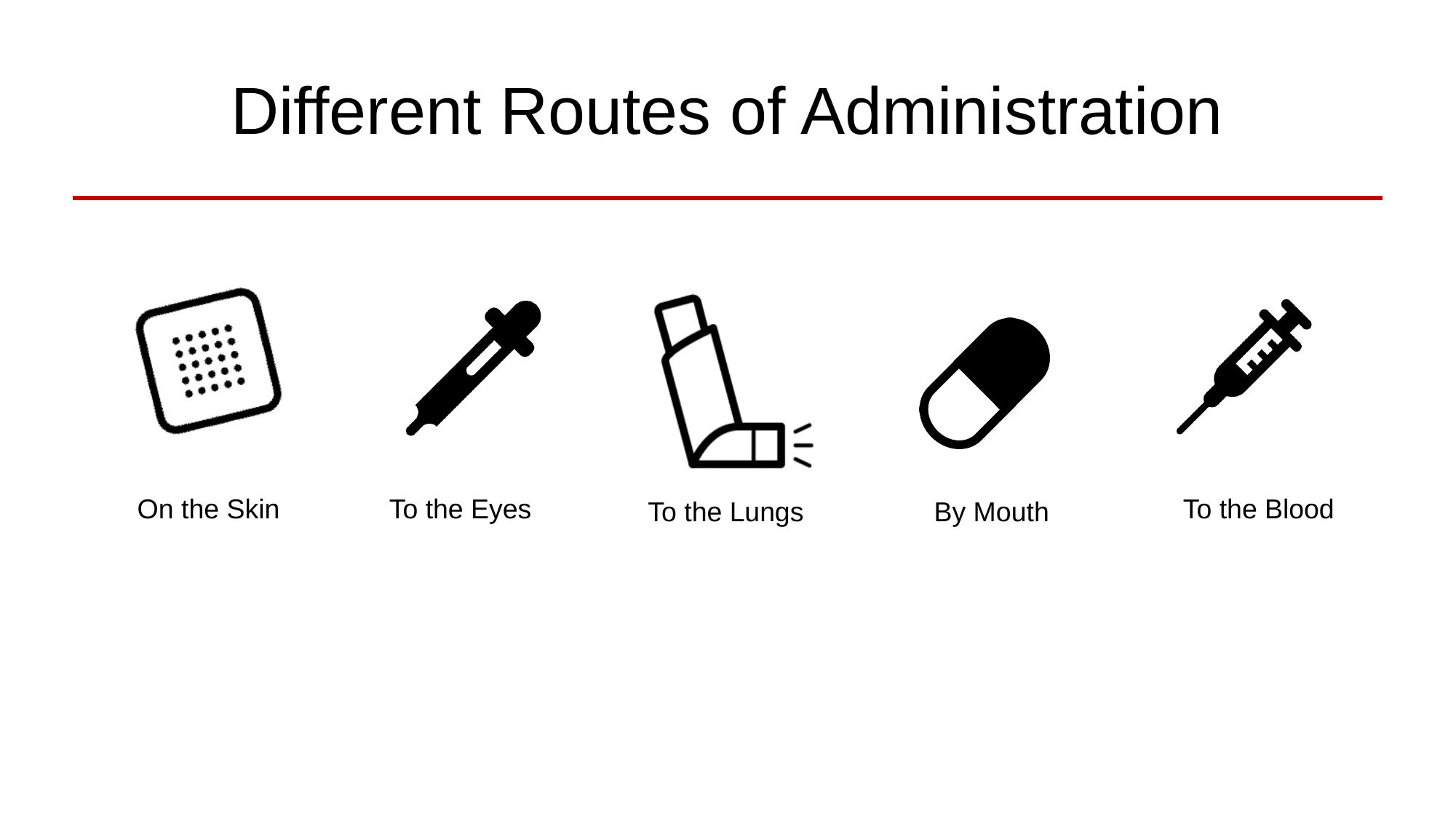

# Different Routes of Administration
On the Skin
To the Eyes
To the Blood
To the Lungs
By Mouth

## Slide 17
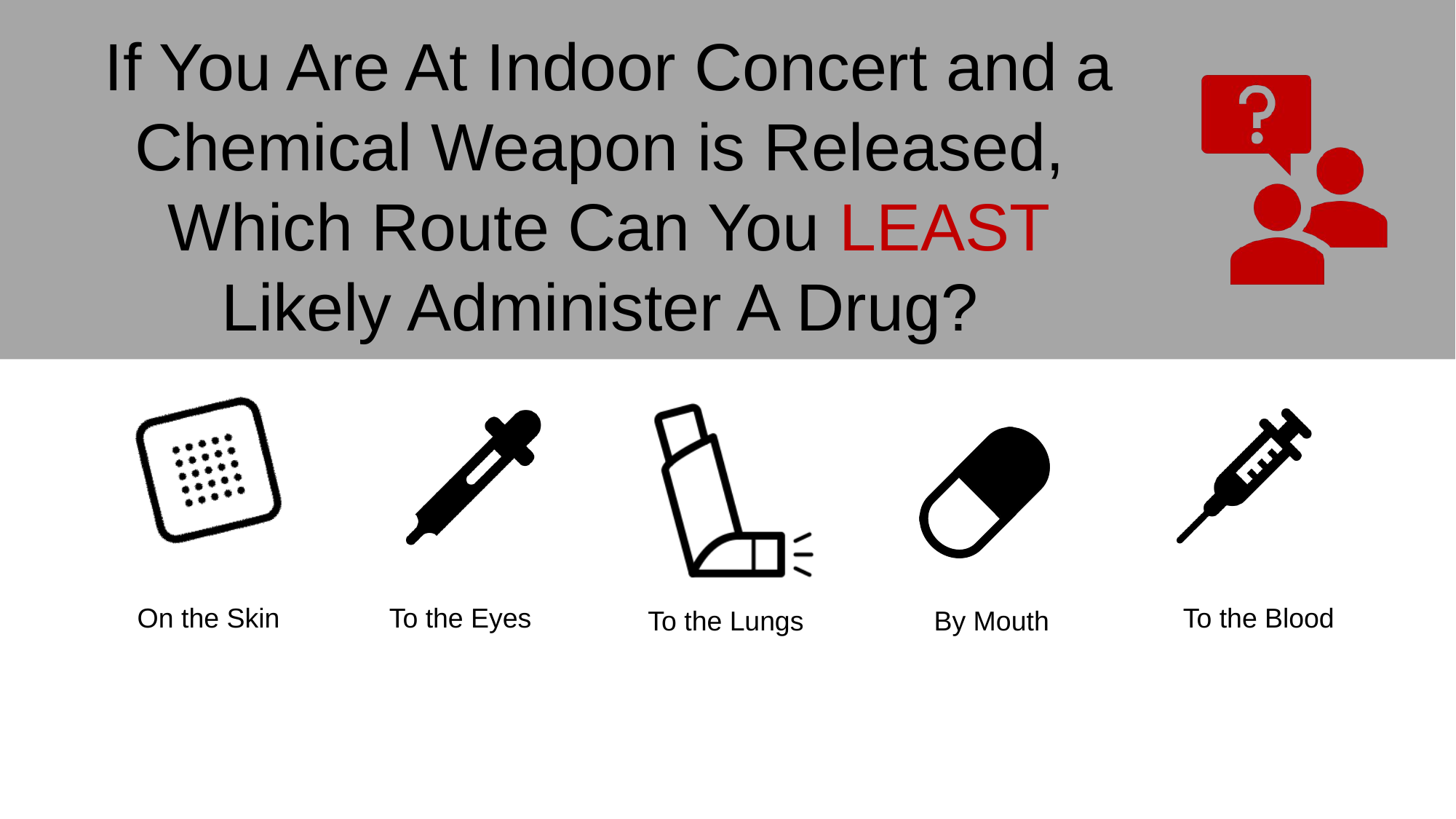

# If You Are At Indoor Concert and a Chemical Weapon is Released, Which Route Can You LEAST Likely Administer A Drug?
On the Skin
To the Eyes
To the Blood
To the Lungs
By Mouth
