# Supplementary material for: Repurposing FDA‐approved drugs to treat chemical weapon toxicities: Interactive case studies for trainees
Source: Pharmacol Res Perspect. 2024 Jul 4;12(4):e1229. doi: 10.1002/prp2.1229 (PMC11223991; doi:10.1002/prp2.1229)
Supplement: Supplementary file 2 — File S2. [file PRP2-12-e1229-s002.pptx]

## Slide 1
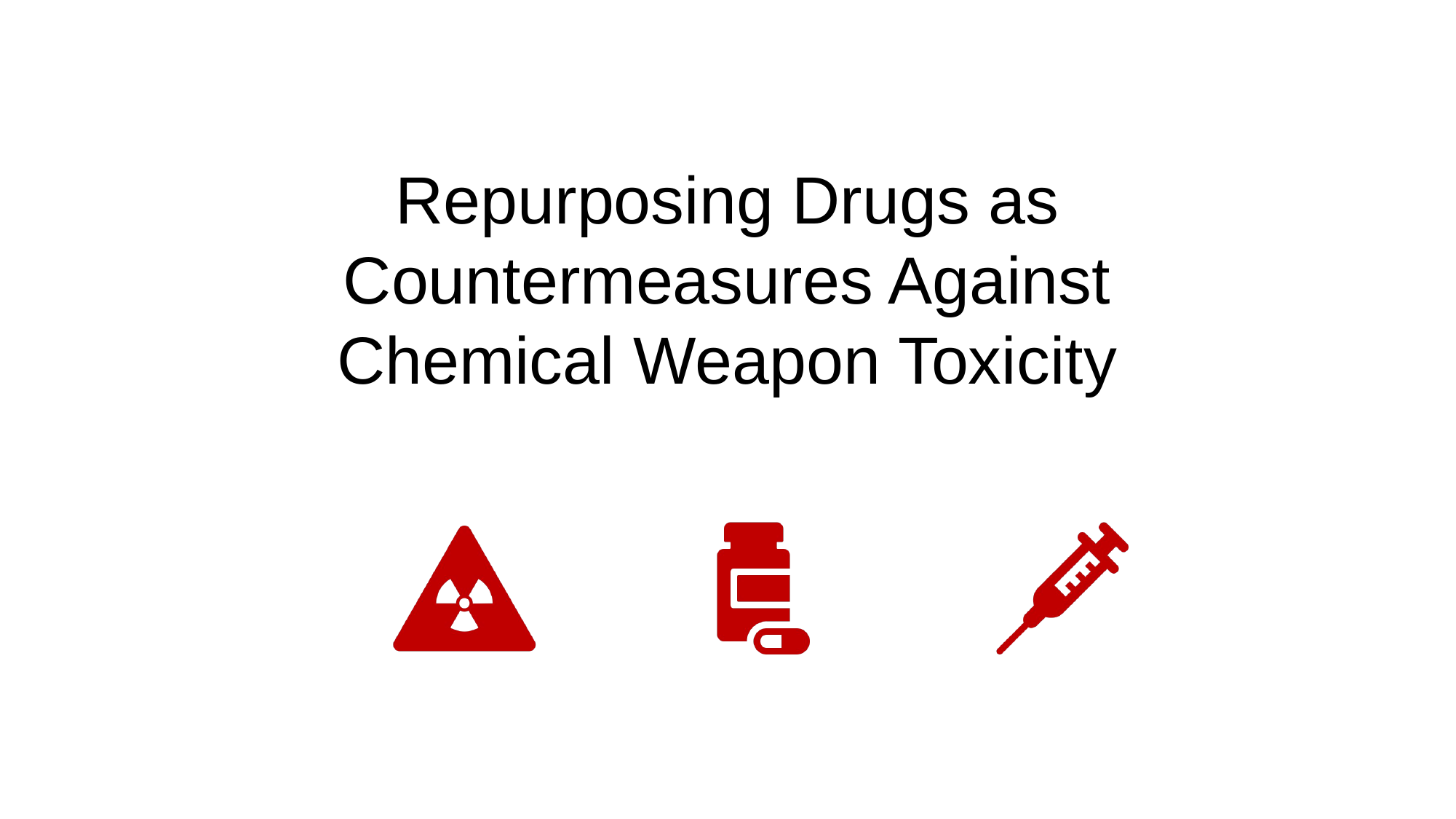

Repurposing Drugs as Countermeasures Against Chemical Weapon Toxicity

## Slide 2
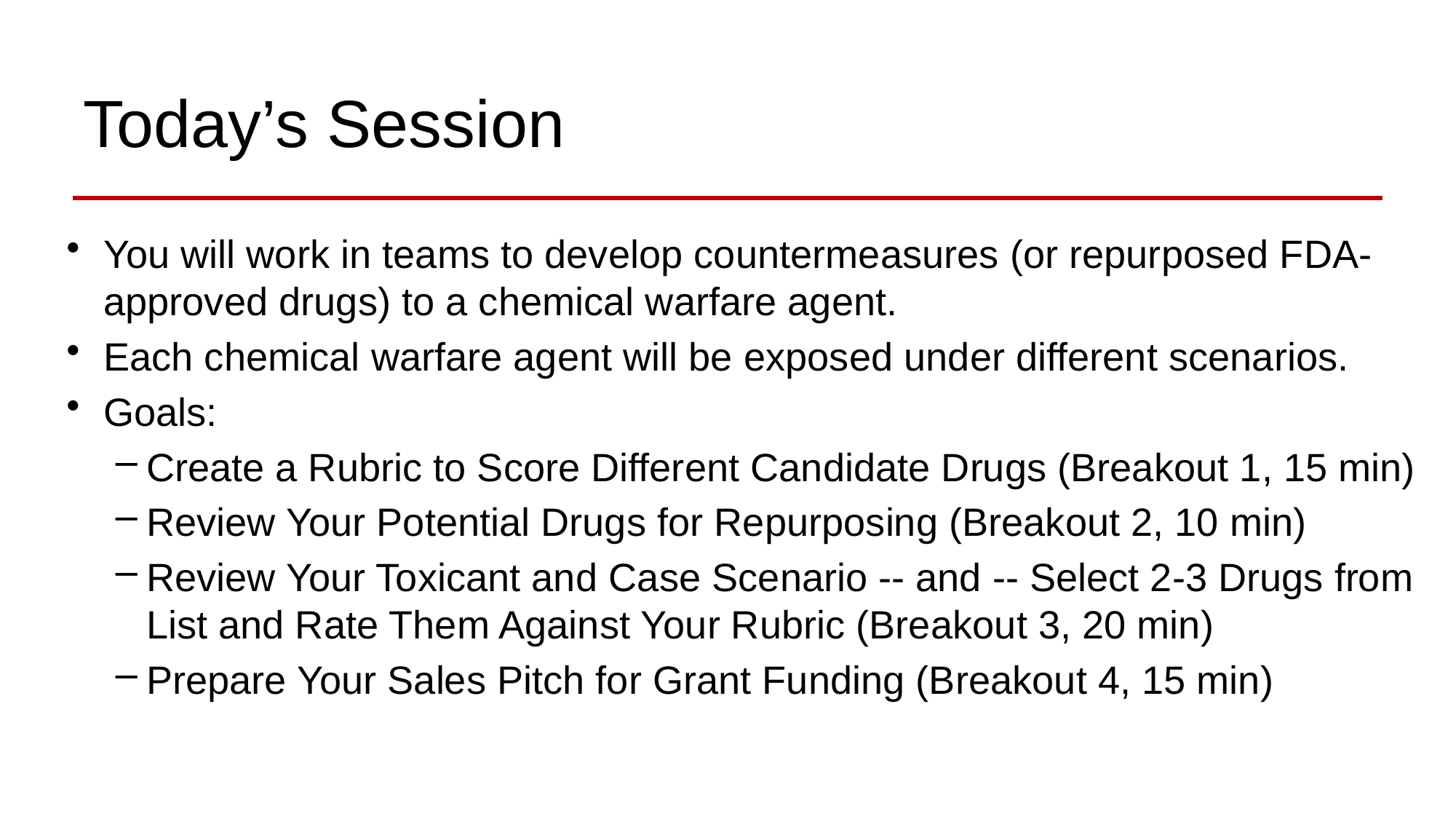

# Today’s Session
You will work in teams to develop countermeasures (or repurposed FDA-approved drugs) to a chemical warfare agent.
Each chemical warfare agent will be exposed under different scenarios.
Goals:
Create a Rubric to Score Different Candidate Drugs (Breakout 1, 15 min)
Review Your Potential Drugs for Repurposing (Breakout 2, 10 min)
Review Your Toxicant and Case Scenario -- and -- Select 2-3 Drugs from List and Rate Them Against Your Rubric (Breakout 3, 20 min)
Prepare Your Sales Pitch for Grant Funding (Breakout 4, 15 min)

## Slide 3
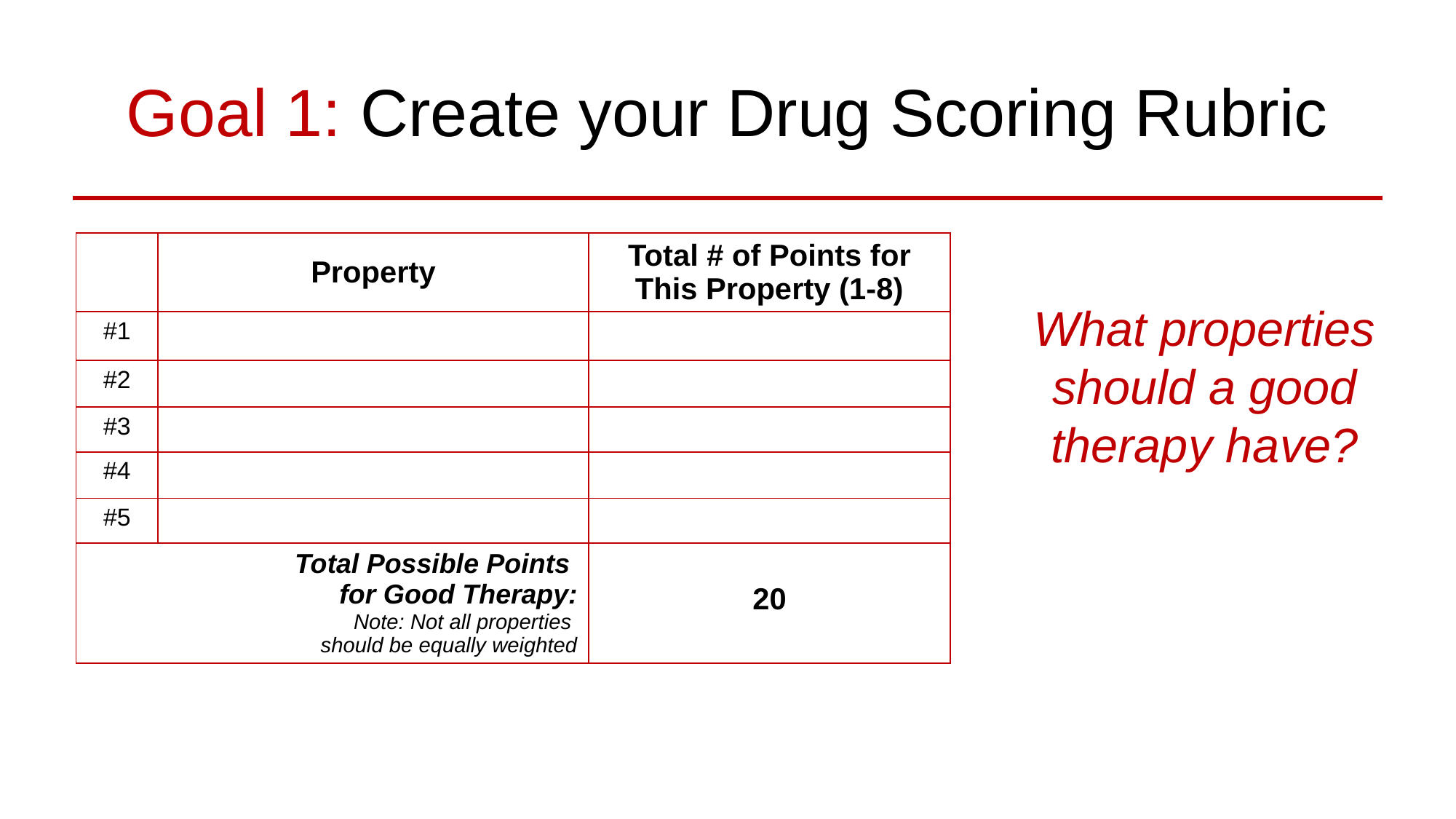

# Goal 1: Create your Drug Scoring Rubric
| | Property | Total # of Points for This Property (1-8) |
| --- | --- | --- |
| #1 | | |
| #2 | | |
| #3 | | |
| #4 | | |
| #5 | | |
| Total Possible Points for Good Therapy: Note: Not all properties should be equally weighted | | 20 |
What properties should a good therapy have?

## Slide 4
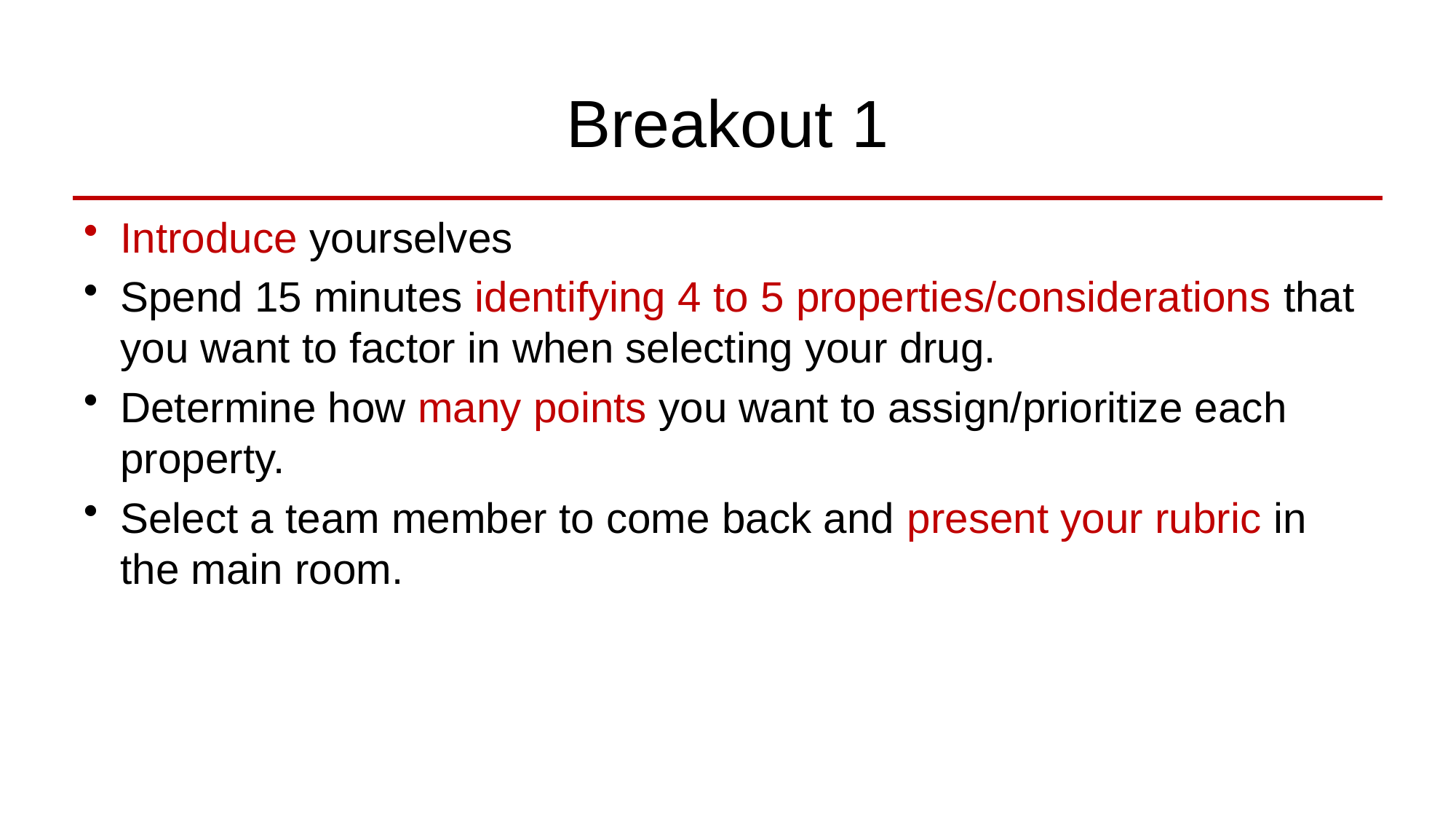

# Breakout 1
Introduce yourselves
Spend 15 minutes identifying 4 to 5 properties/considerations that you want to factor in when selecting your drug.
Determine how many points you want to assign/prioritize each property.
Select a team member to come back and present your rubric in the main room.

## Slide 5
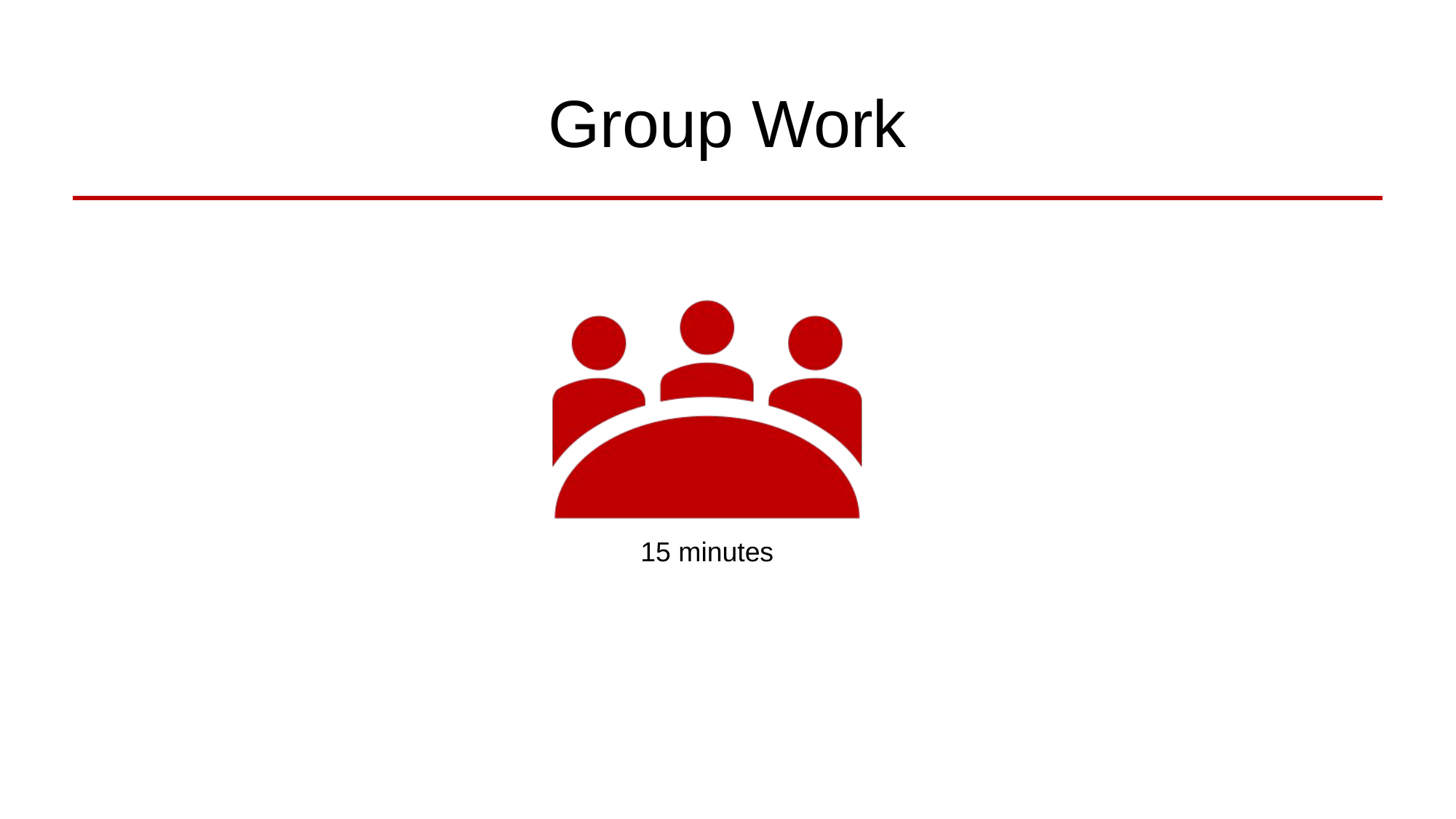

# Group Work
15 minutes

## Slide 6
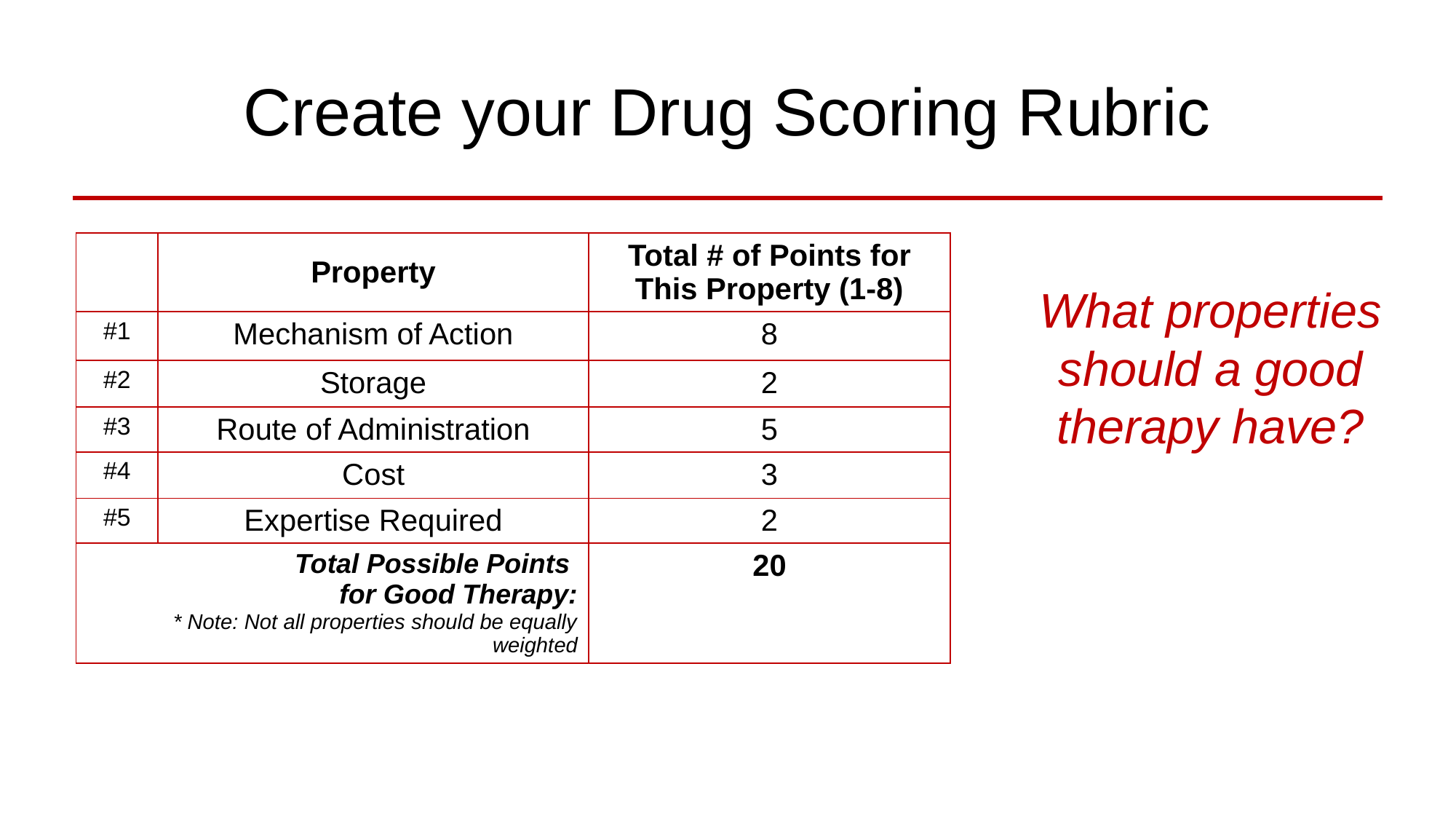

# Create your Drug Scoring Rubric
| | Property | Total # of Points for This Property (1-8) |
| --- | --- | --- |
| #1 | Mechanism of Action | 8 |
| #2 | Storage | 2 |
| #3 | Route of Administration | 5 |
| #4 | Cost | 3 |
| #5 | Expertise Required | 2 |
| Total Possible Points for Good Therapy: \* Note: Not all properties should be equally weighted | | 20 |
What properties should a good therapy have?

## Slide 7
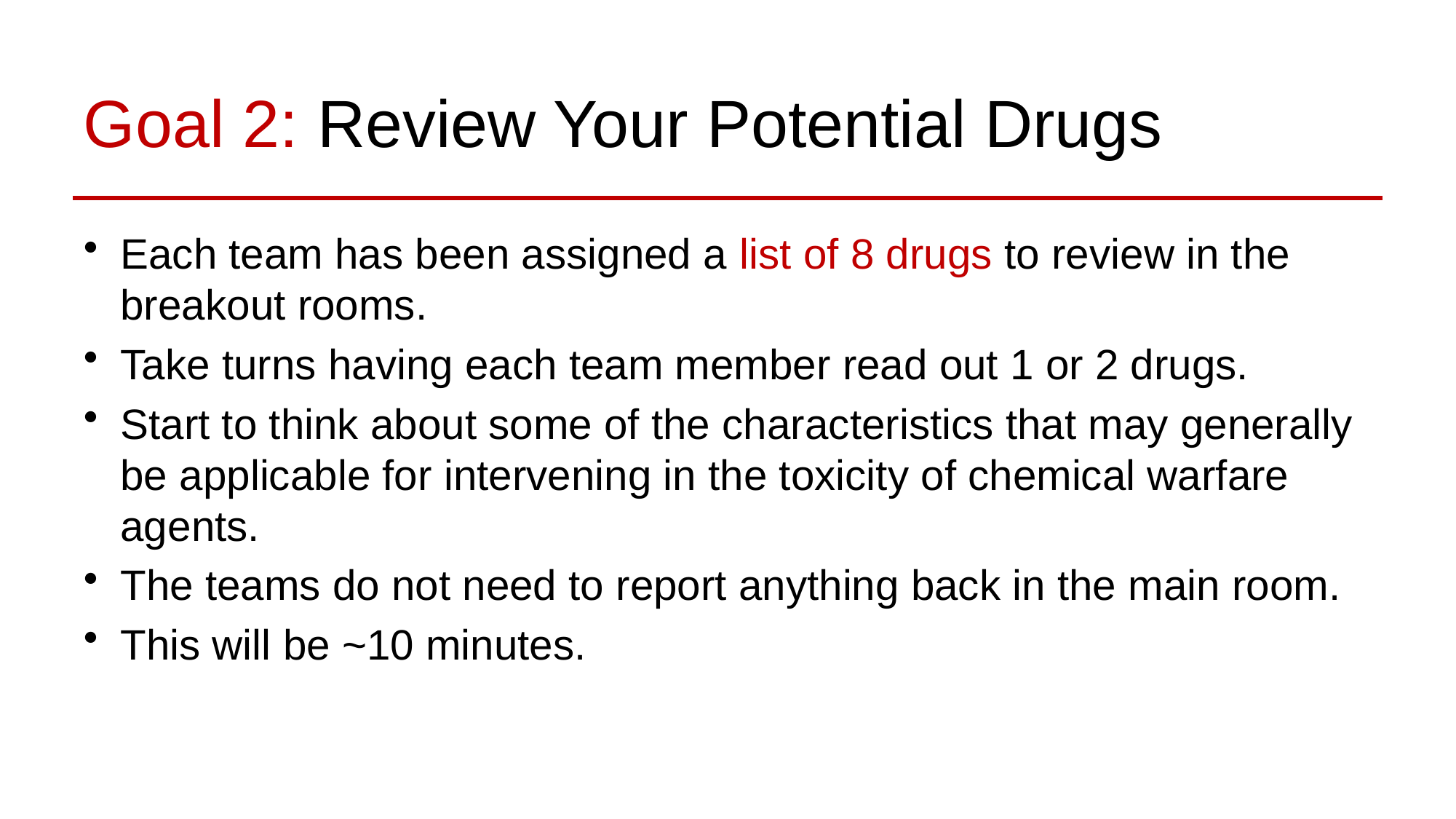

# Goal 2: Review Your Potential Drugs
Each team has been assigned a list of 8 drugs to review in the breakout rooms.
Take turns having each team member read out 1 or 2 drugs.
Start to think about some of the characteristics that may generally be applicable for intervening in the toxicity of chemical warfare agents.
The teams do not need to report anything back in the main room.
This will be ~10 minutes.

## Slide 8
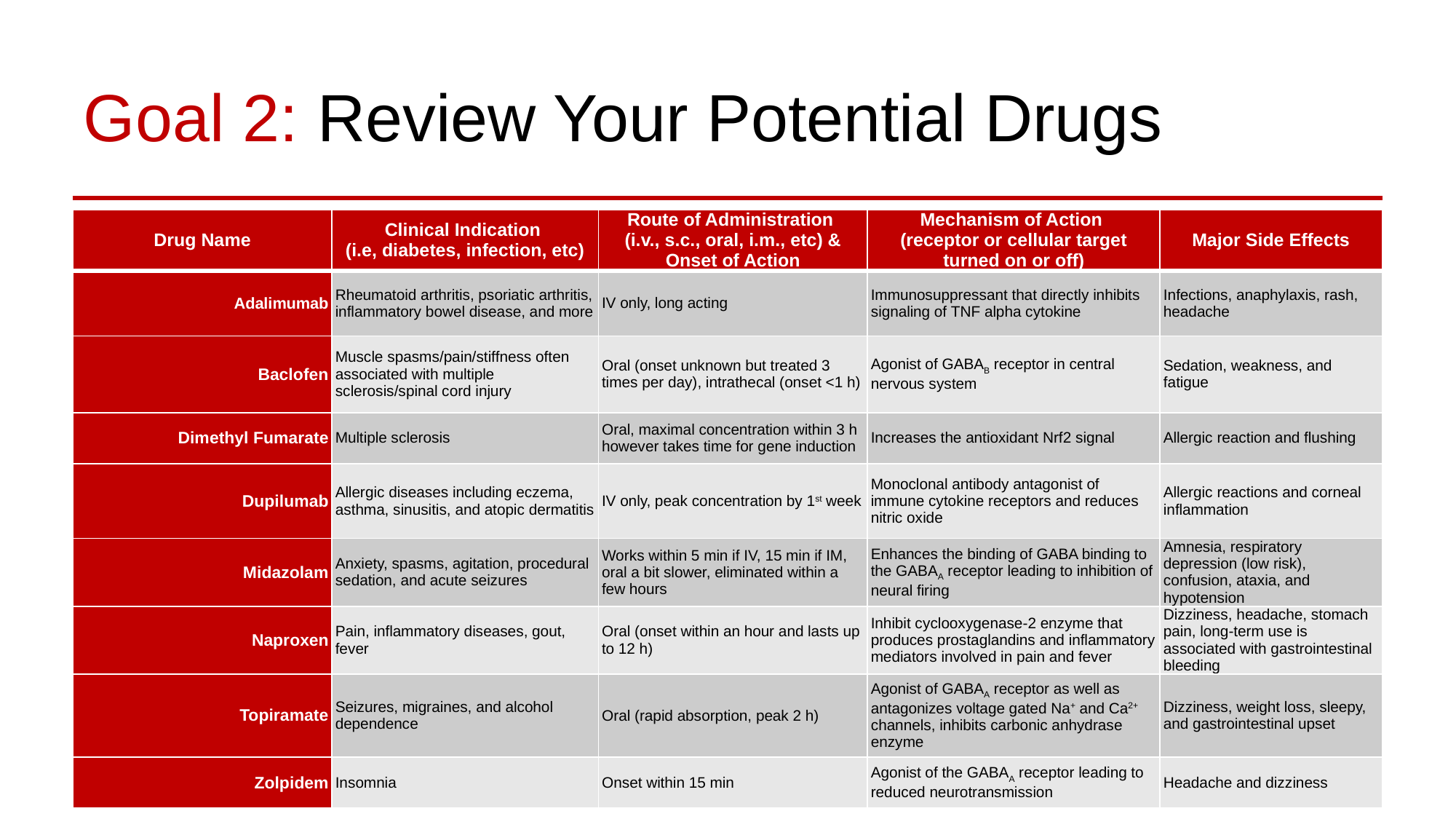

# Goal 2: Review Your Potential Drugs
| Drug Name | Clinical Indication (i.e, diabetes, infection, etc) | Route of Administration (i.v., s.c., oral, i.m., etc) & Onset of Action | Mechanism of Action (receptor or cellular target turned on or off) | Major Side Effects |
| --- | --- | --- | --- | --- |
| Adalimumab | Rheumatoid arthritis, psoriatic arthritis, inflammatory bowel disease, and more | IV only, long acting | Immunosuppressant that directly inhibits signaling of TNF alpha cytokine | Infections, anaphylaxis, rash, headache |
| Baclofen | Muscle spasms/pain/stiffness often associated with multiple sclerosis/spinal cord injury | Oral (onset unknown but treated 3 times per day), intrathecal (onset <1 h) | Agonist of GABAB receptor in central nervous system | Sedation, weakness, and fatigue |
| Dimethyl Fumarate | Multiple sclerosis | Oral, maximal concentration within 3 h however takes time for gene induction | Increases the antioxidant Nrf2 signal | Allergic reaction and flushing |
| Dupilumab | Allergic diseases including eczema, asthma, sinusitis, and atopic dermatitis | IV only, peak concentration by 1st week | Monoclonal antibody antagonist of immune cytokine receptors and reduces nitric oxide | Allergic reactions and corneal inflammation |
| Midazolam | Anxiety, spasms, agitation, procedural sedation, and acute seizures | Works within 5 min if IV, 15 min if IM, oral a bit slower, eliminated within a few hours | Enhances the binding of GABA binding to the GABAA receptor leading to inhibition of neural firing | Amnesia, respiratory depression (low risk), confusion, ataxia, and hypotension |
| Naproxen | Pain, inflammatory diseases, gout, fever | Oral (onset within an hour and lasts up to 12 h) | Inhibit cyclooxygenase-2 enzyme that produces prostaglandins and inflammatory mediators involved in pain and fever | Dizziness, headache, stomach pain, long-term use is associated with gastrointestinal bleeding |
| Topiramate | Seizures, migraines, and alcohol dependence | Oral (rapid absorption, peak 2 h) | Agonist of GABAA receptor as well as antagonizes voltage gated Na+ and Ca2+ channels, inhibits carbonic anhydrase enzyme | Dizziness, weight loss, sleepy, and gastrointestinal upset |
| Zolpidem | Insomnia | Onset within 15 min | Agonist of the GABAA receptor leading to reduced neurotransmission | Headache and dizziness |

## Slide 9
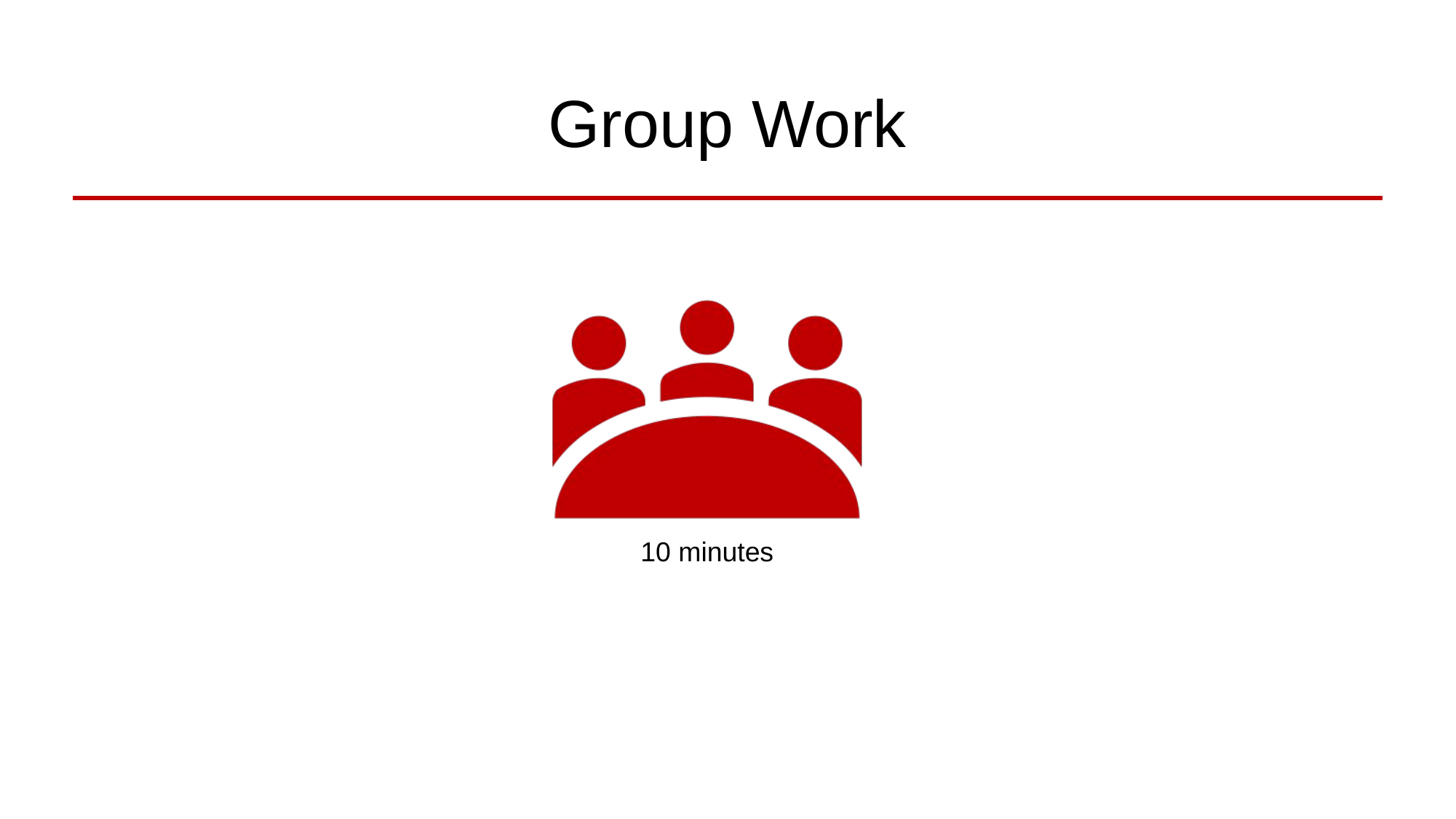

# Group Work
10 minutes

## Slide 10
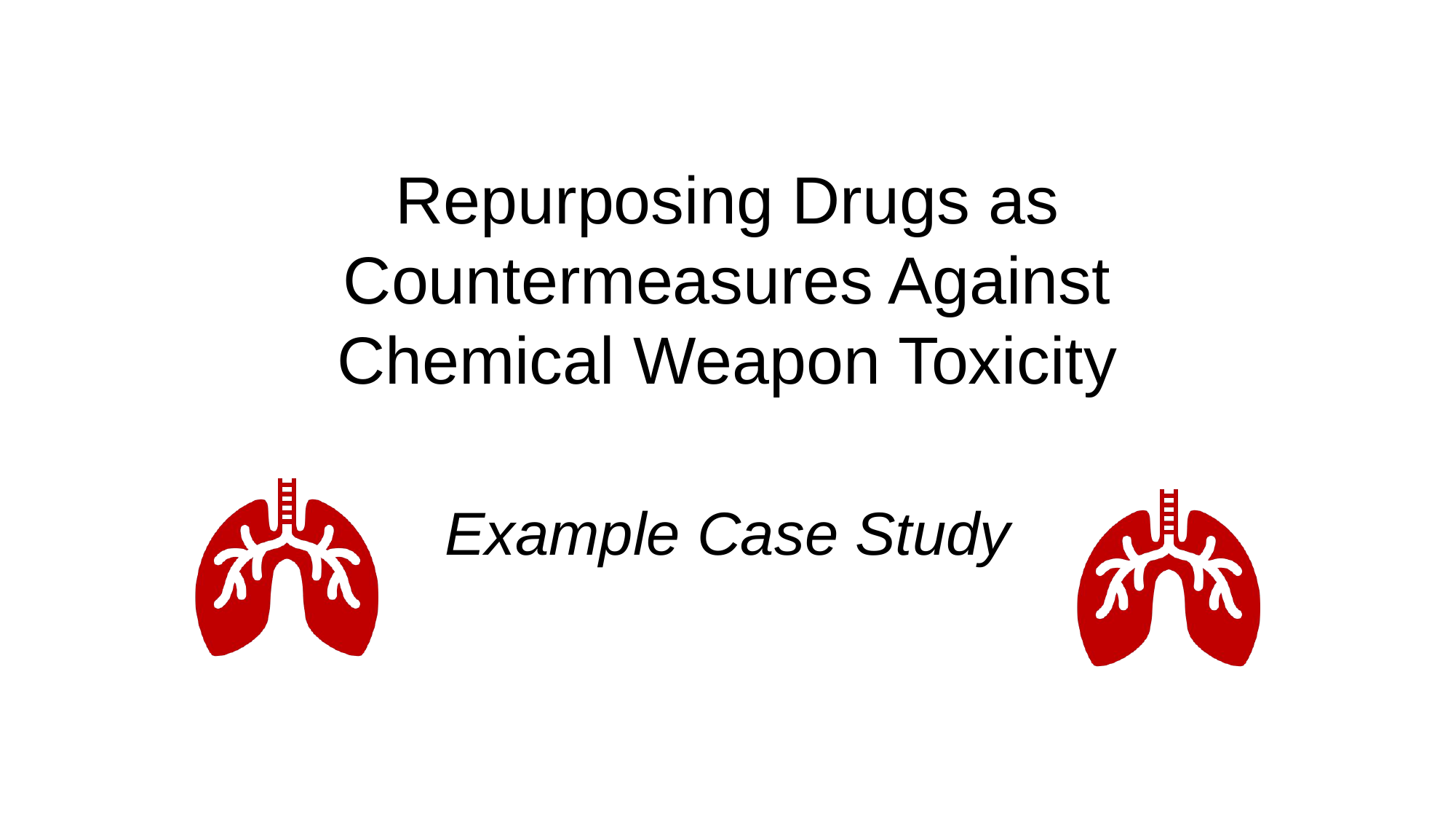

Repurposing Drugs as Countermeasures Against Chemical Weapon Toxicity
Example Case Study

## Slide 11
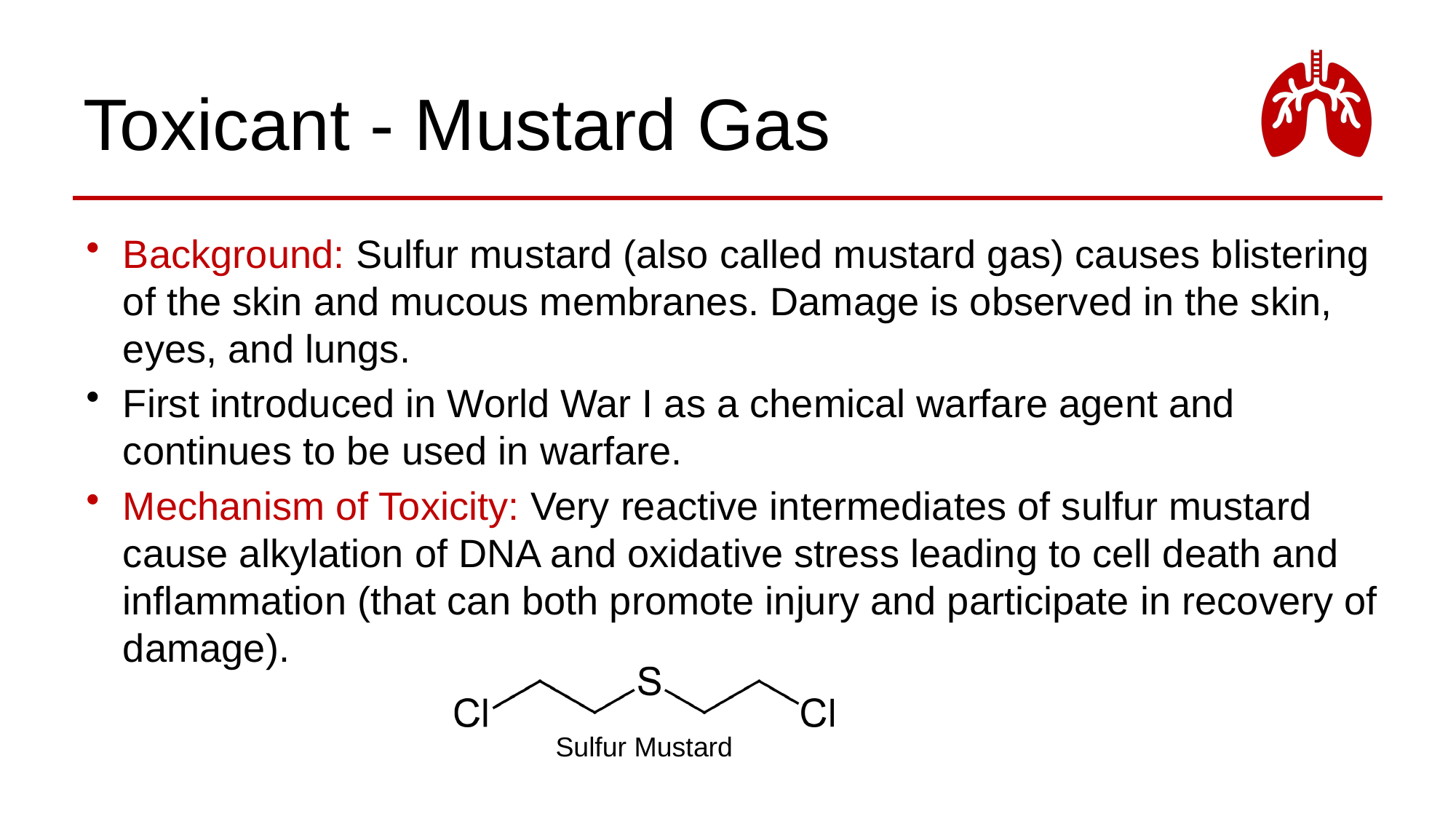

# Toxicant - Mustard Gas
Background: Sulfur mustard (also called mustard gas) causes blistering of the skin and mucous membranes. Damage is observed in the skin, eyes, and lungs.
First introduced in World War I as a chemical warfare agent and continues to be used in warfare.
Mechanism of Toxicity: Very reactive intermediates of sulfur mustard cause alkylation of DNA and oxidative stress leading to cell death and inflammation (that can both promote injury and participate in recovery of damage).
Sulfur Mustard

## Slide 12
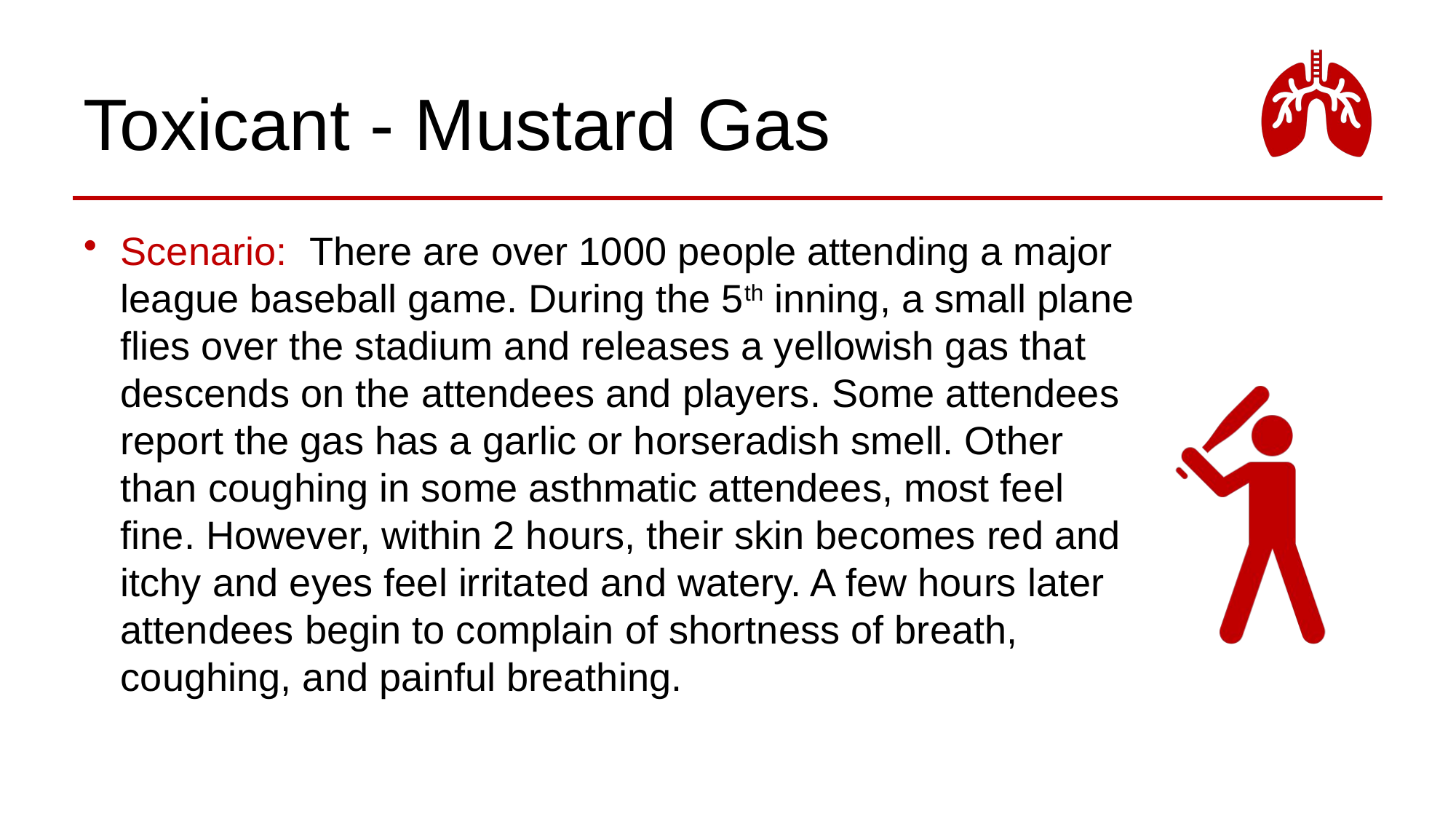

# Toxicant - Mustard Gas
Scenario: There are over 1000 people attending a major league baseball game. During the 5th inning, a small plane flies over the stadium and releases a yellowish gas that descends on the attendees and players. Some attendees report the gas has a garlic or horseradish smell. Other than coughing in some asthmatic attendees, most feel fine. However, within 2 hours, their skin becomes red and itchy and eyes feel irritated and watery. A few hours later attendees begin to complain of shortness of breath, coughing, and painful breathing.

## Slide 13
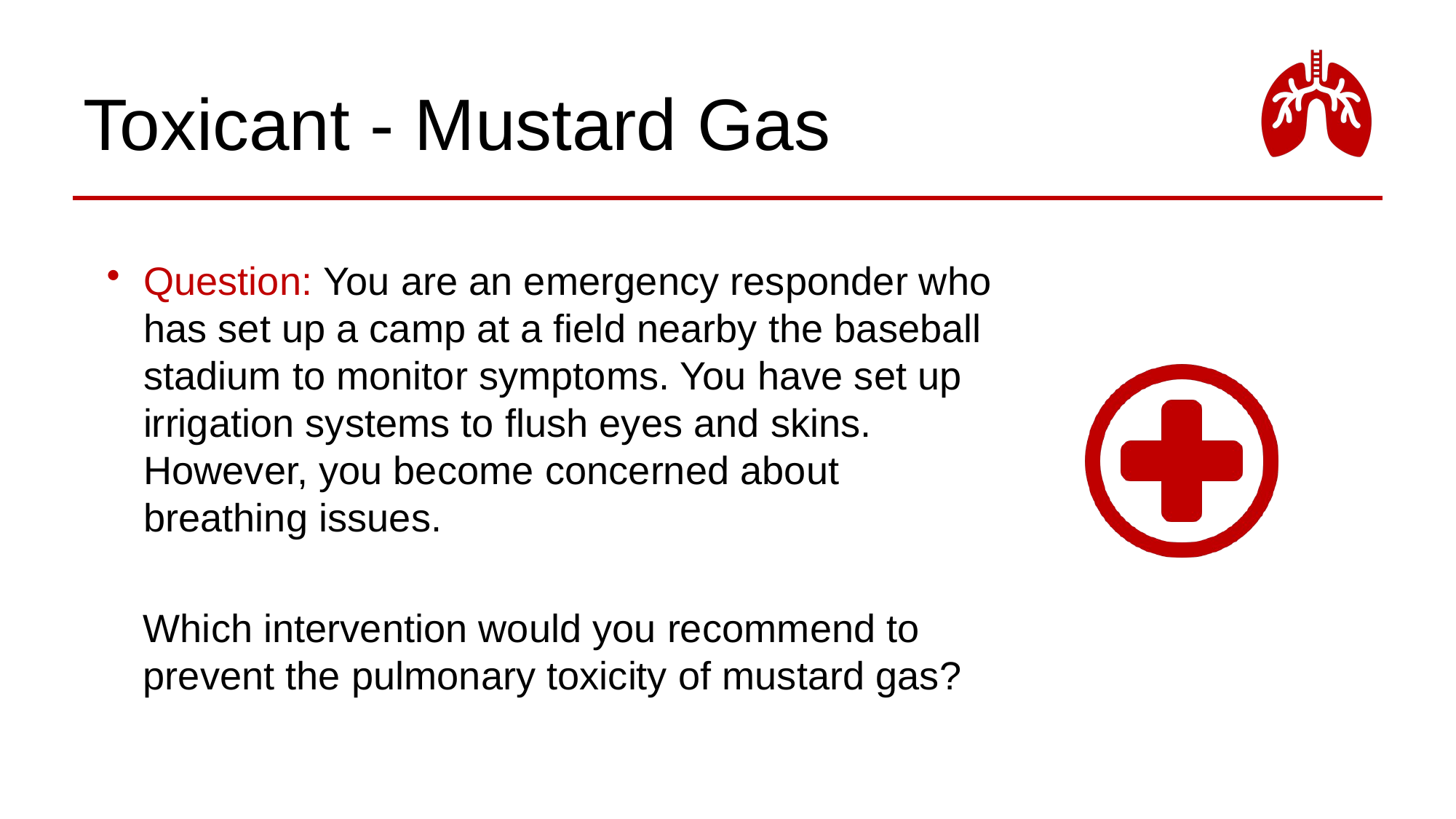

# Toxicant - Mustard Gas
Question: You are an emergency responder who has set up a camp at a field nearby the baseball stadium to monitor symptoms. You have set up irrigation systems to flush eyes and skins. However, you become concerned about breathing issues.
Which intervention would you recommend to prevent the pulmonary toxicity of mustard gas?

## Slide 14
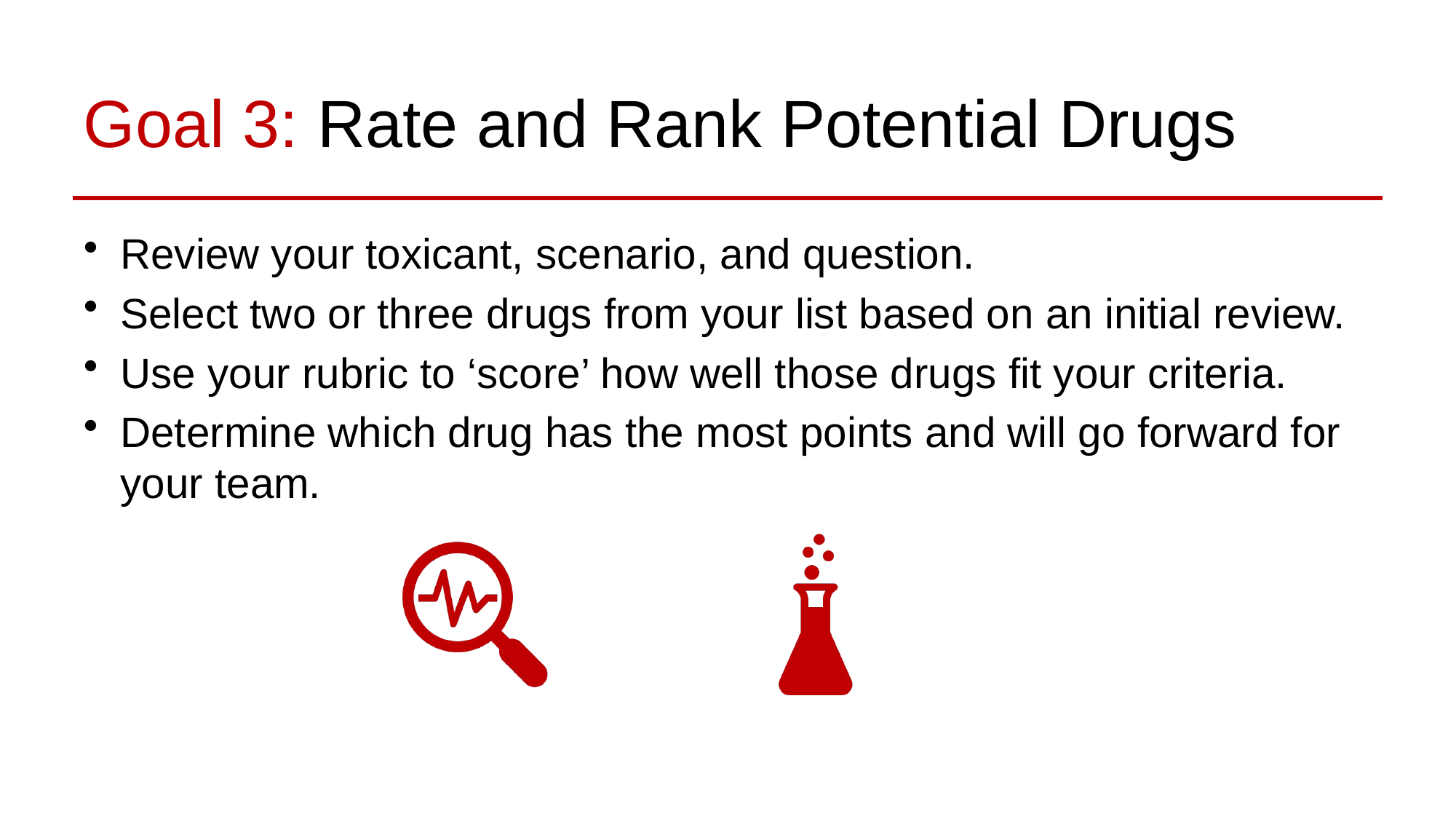

# Goal 3: Rate and Rank Potential Drugs
Review your toxicant, scenario, and question.
Select two or three drugs from your list based on an initial review.
Use your rubric to ‘score’ how well those drugs fit your criteria.
Determine which drug has the most points and will go forward for your team.

## Slide 15
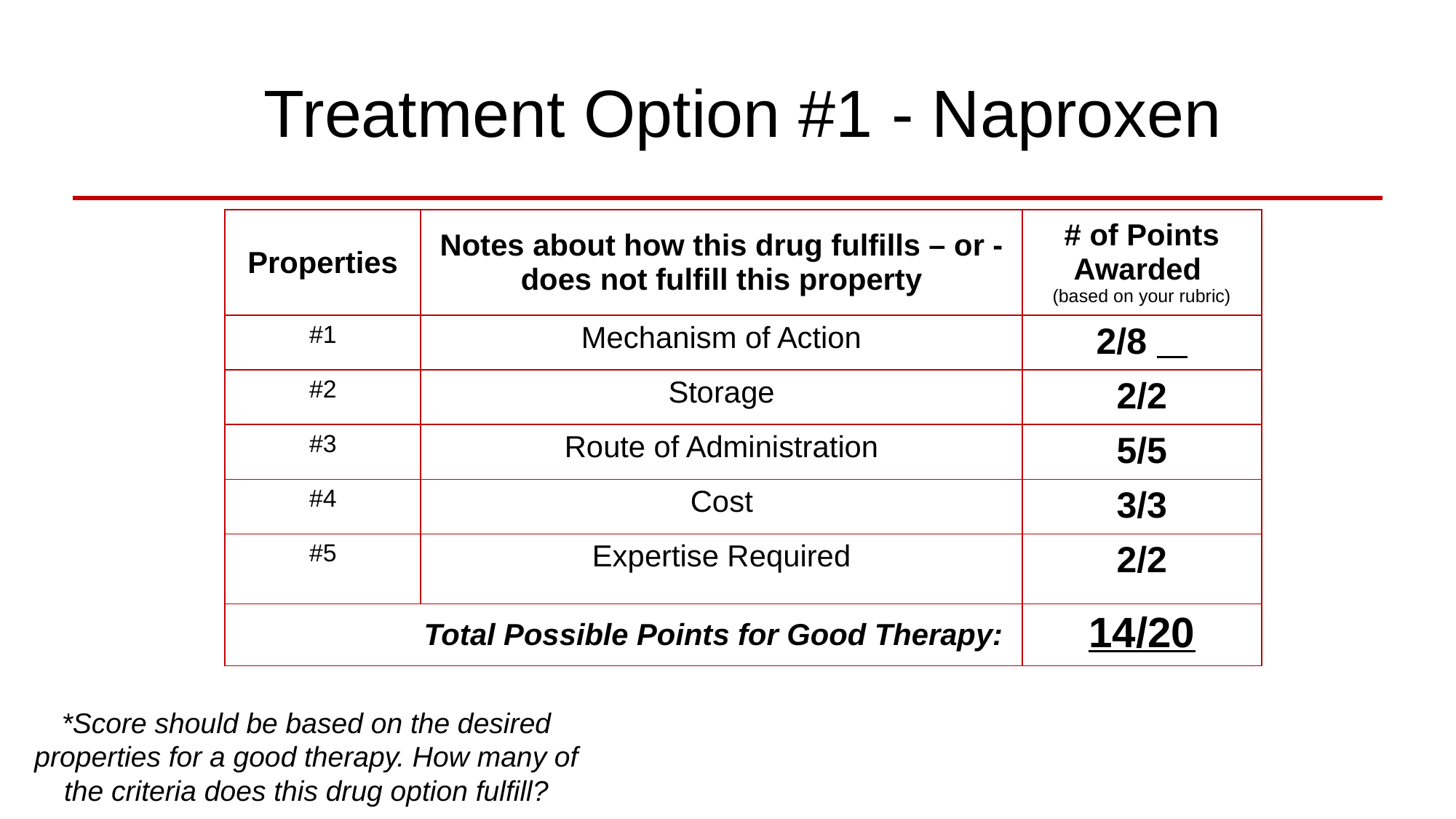

# Treatment Option #1 - Naproxen
| Properties | Notes about how this drug fulfills – or -does not fulfill this property | # of Points Awarded (based on your rubric) |
| --- | --- | --- |
| #1 | Mechanism of Action | 2/8 |
| #2 | Storage | 2/2 |
| #3 | Route of Administration | 5/5 |
| #4 | Cost | 3/3 |
| #5 | Expertise Required | 2/2 |
| Total Possible Points for Good Therapy: | | 14/20 |
*Score should be based on the desired properties for a good therapy. How many of the criteria does this drug option fulfill?

## Slide 16
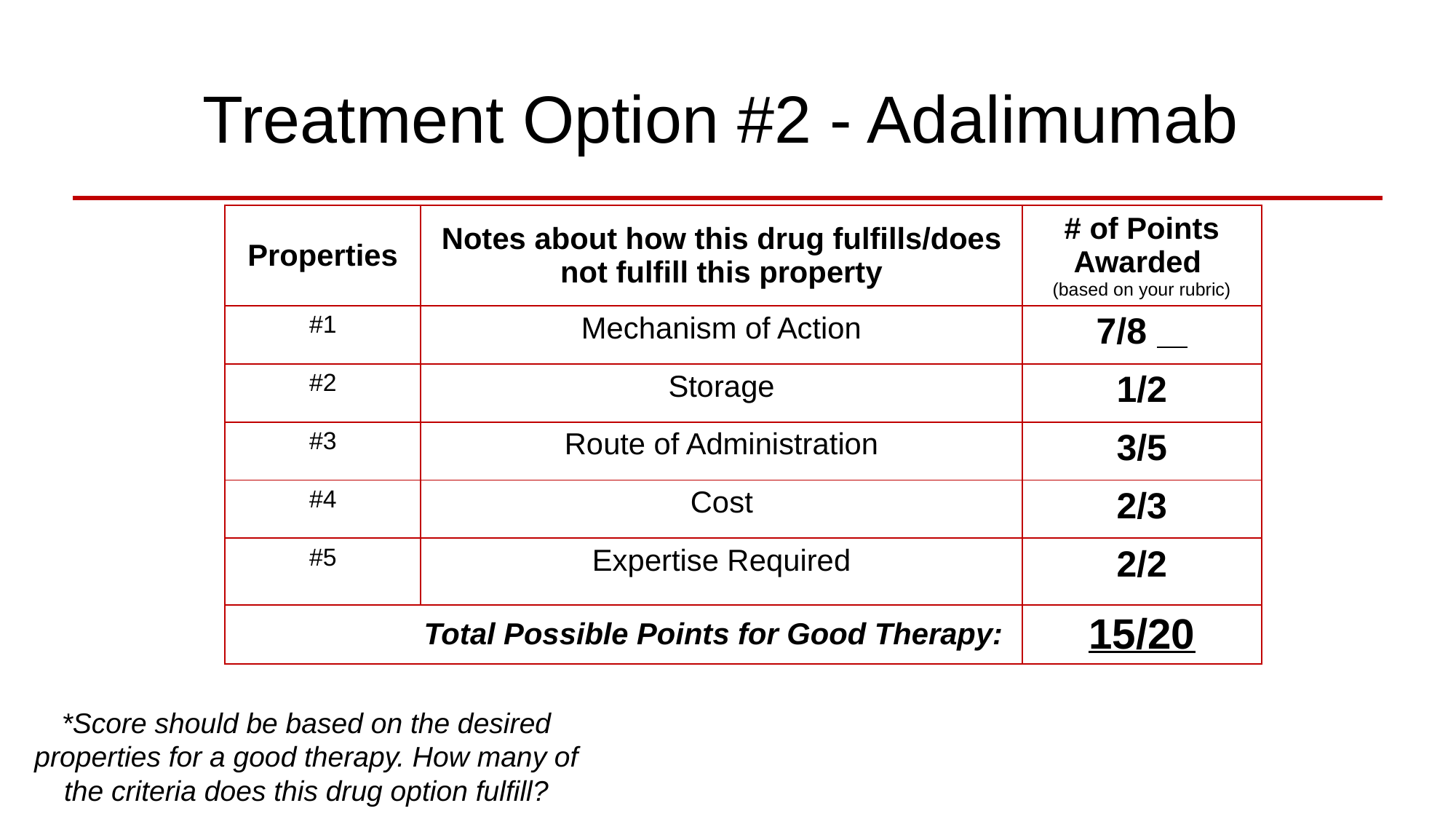

# Treatment Option #2 - Adalimumab
| Properties | Notes about how this drug fulfills/does not fulfill this property | # of Points Awarded (based on your rubric) |
| --- | --- | --- |
| #1 | Mechanism of Action | 7/8 |
| #2 | Storage | 1/2 |
| #3 | Route of Administration | 3/5 |
| #4 | Cost | 2/3 |
| #5 | Expertise Required | 2/2 |
| Total Possible Points for Good Therapy: | | 15/20 |
*Score should be based on the desired properties for a good therapy. How many of the criteria does this drug option fulfill?

## Slide 17
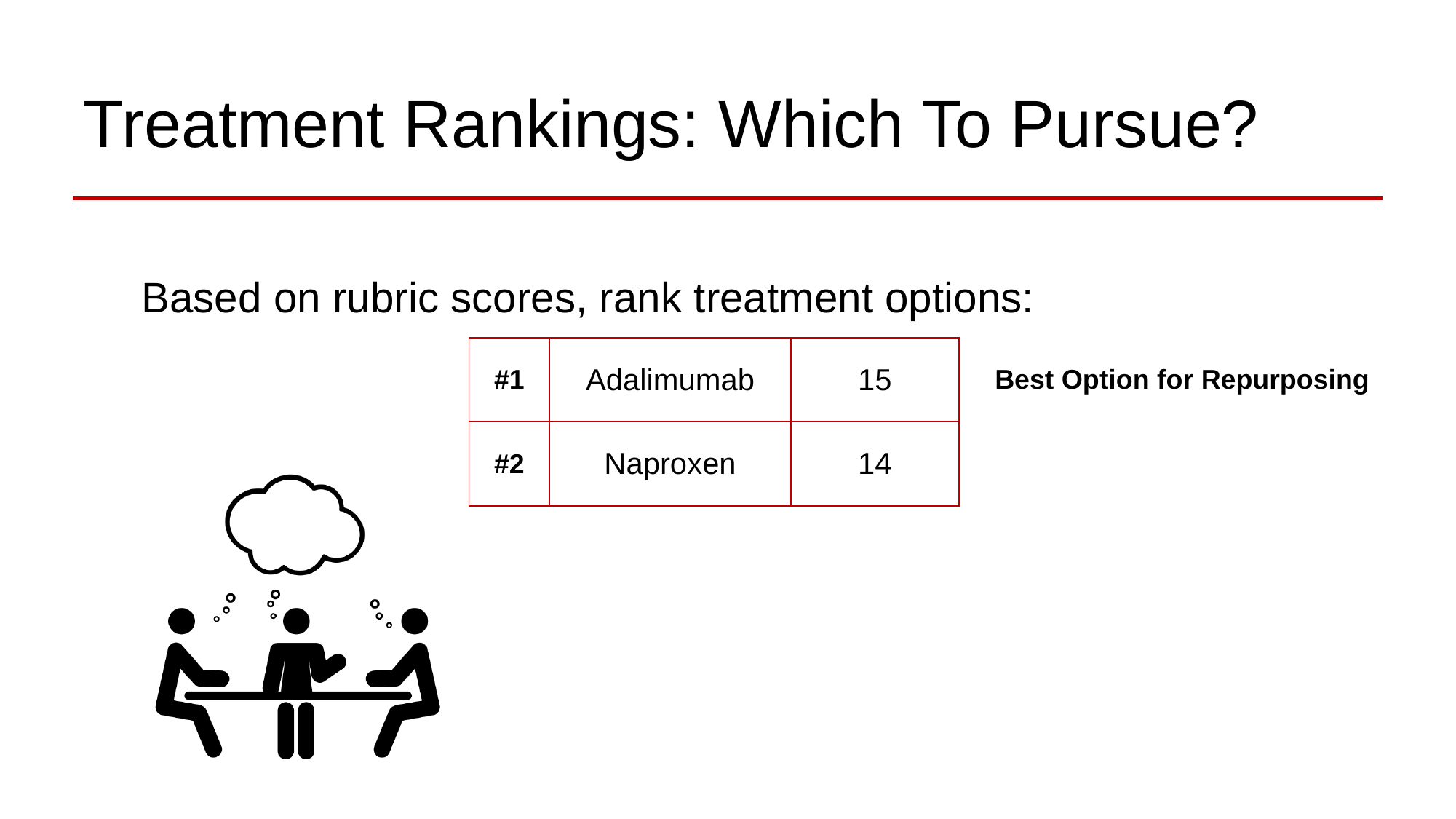

# Treatment Rankings: Which To Pursue?
Based on rubric scores, rank treatment options:
| #1 | Adalimumab | 15 |
| --- | --- | --- |
| #2 | Naproxen | 14 |
Best Option for Repurposing

## Slide 18
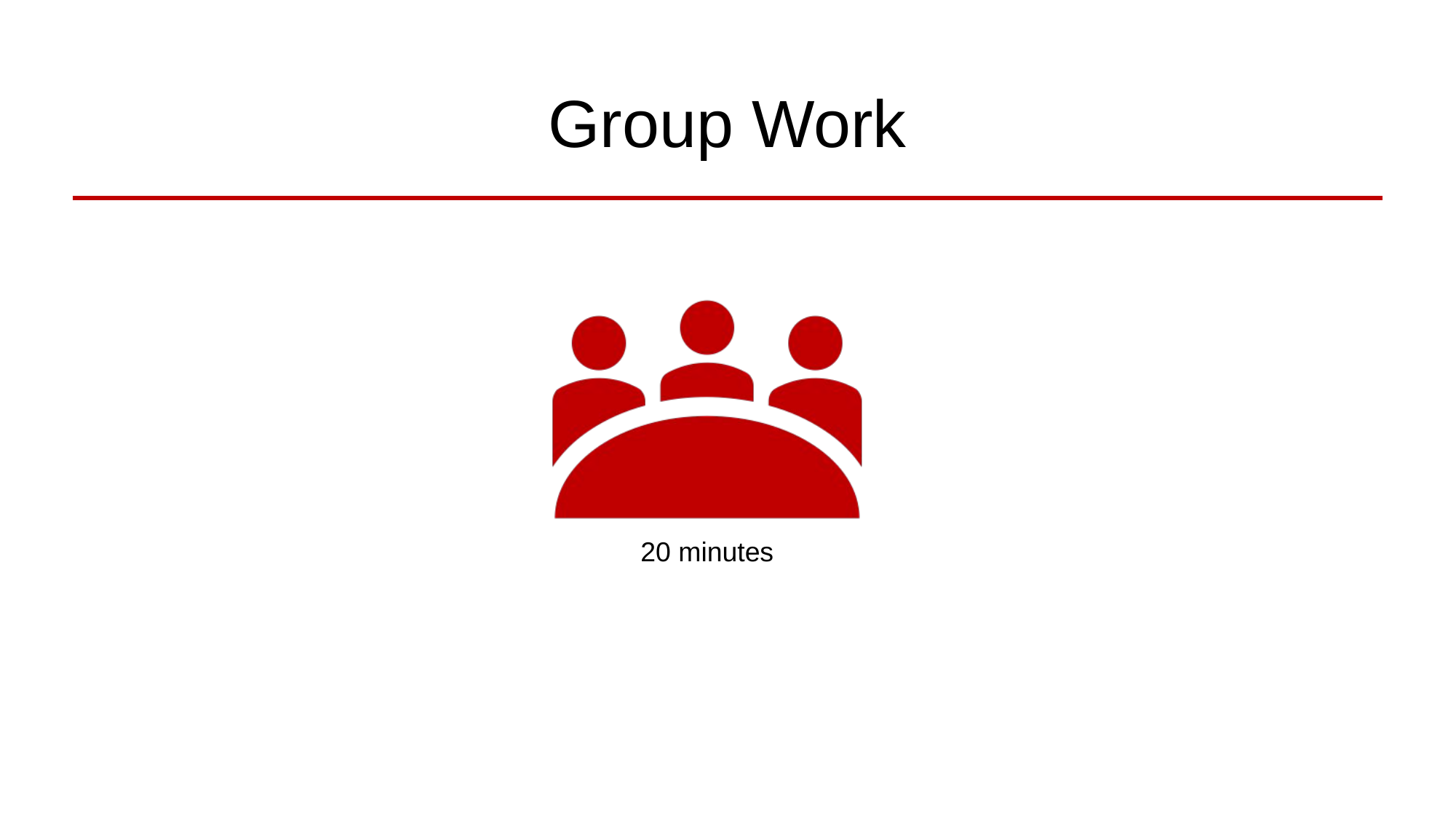

# Group Work
20 minutes

## Slide 19
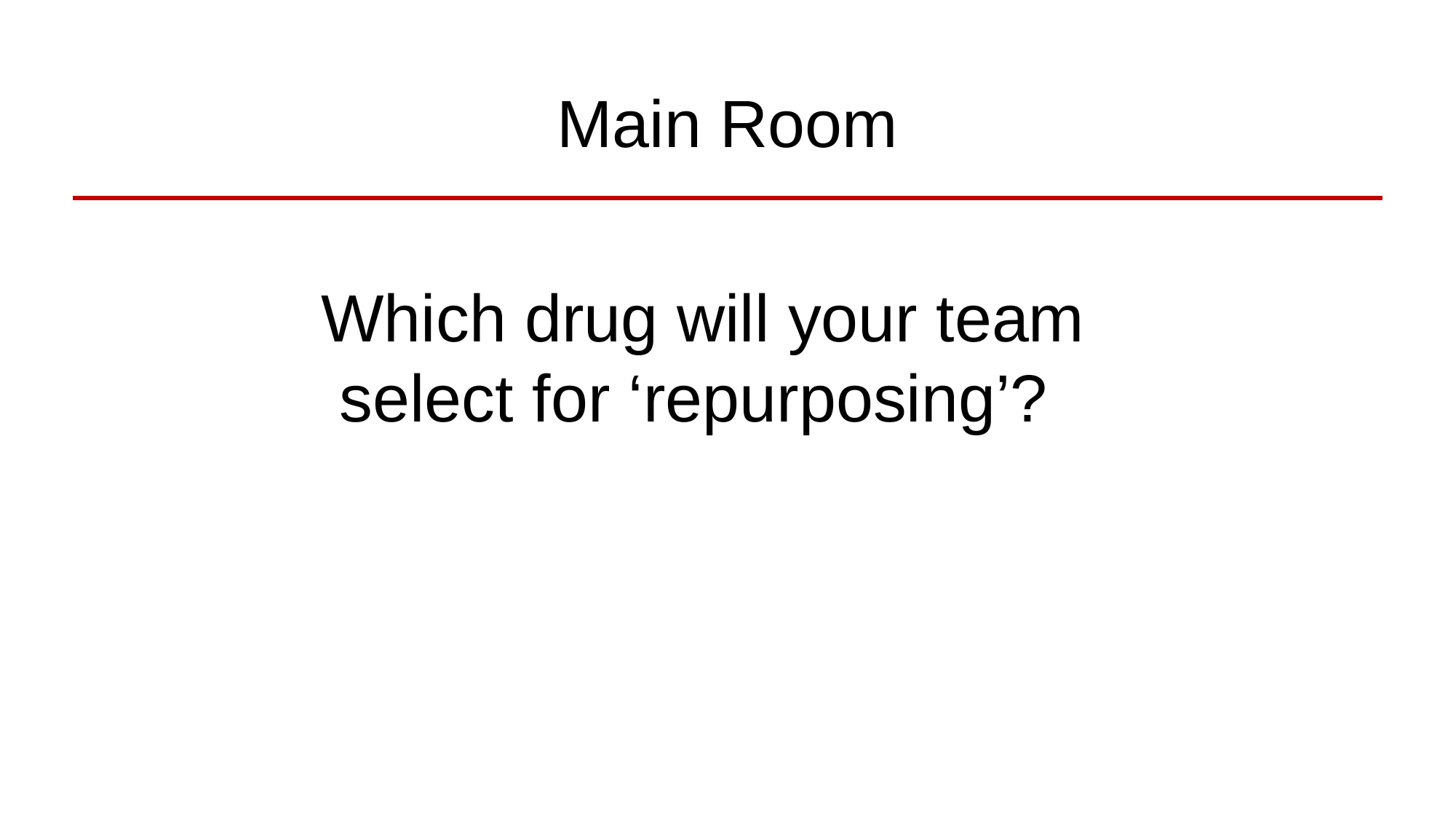

# Main Room
Which drug will your team select for ‘repurposing’?

## Slide 20
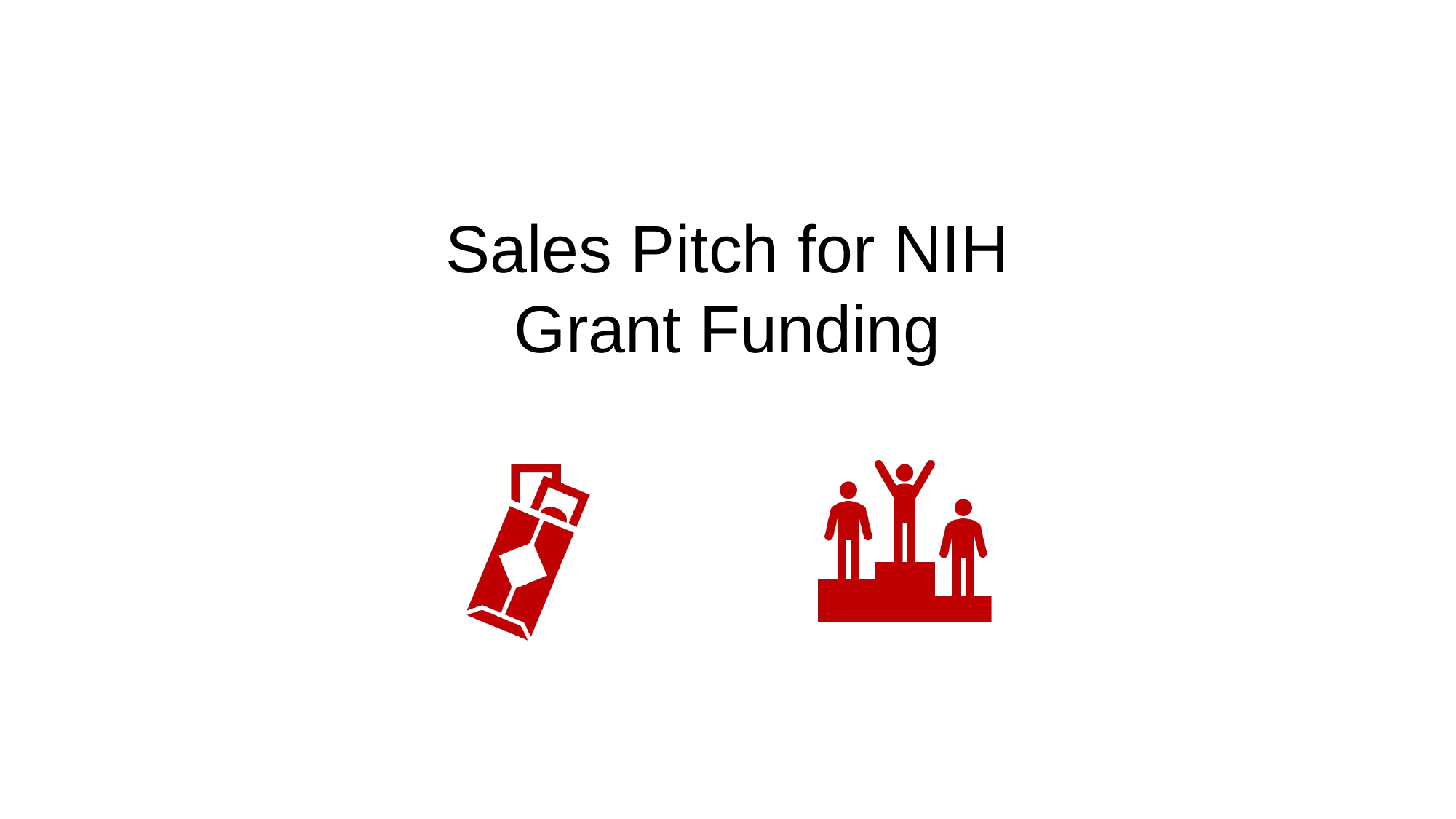

Sales Pitch for NIH Grant Funding

## Slide 21
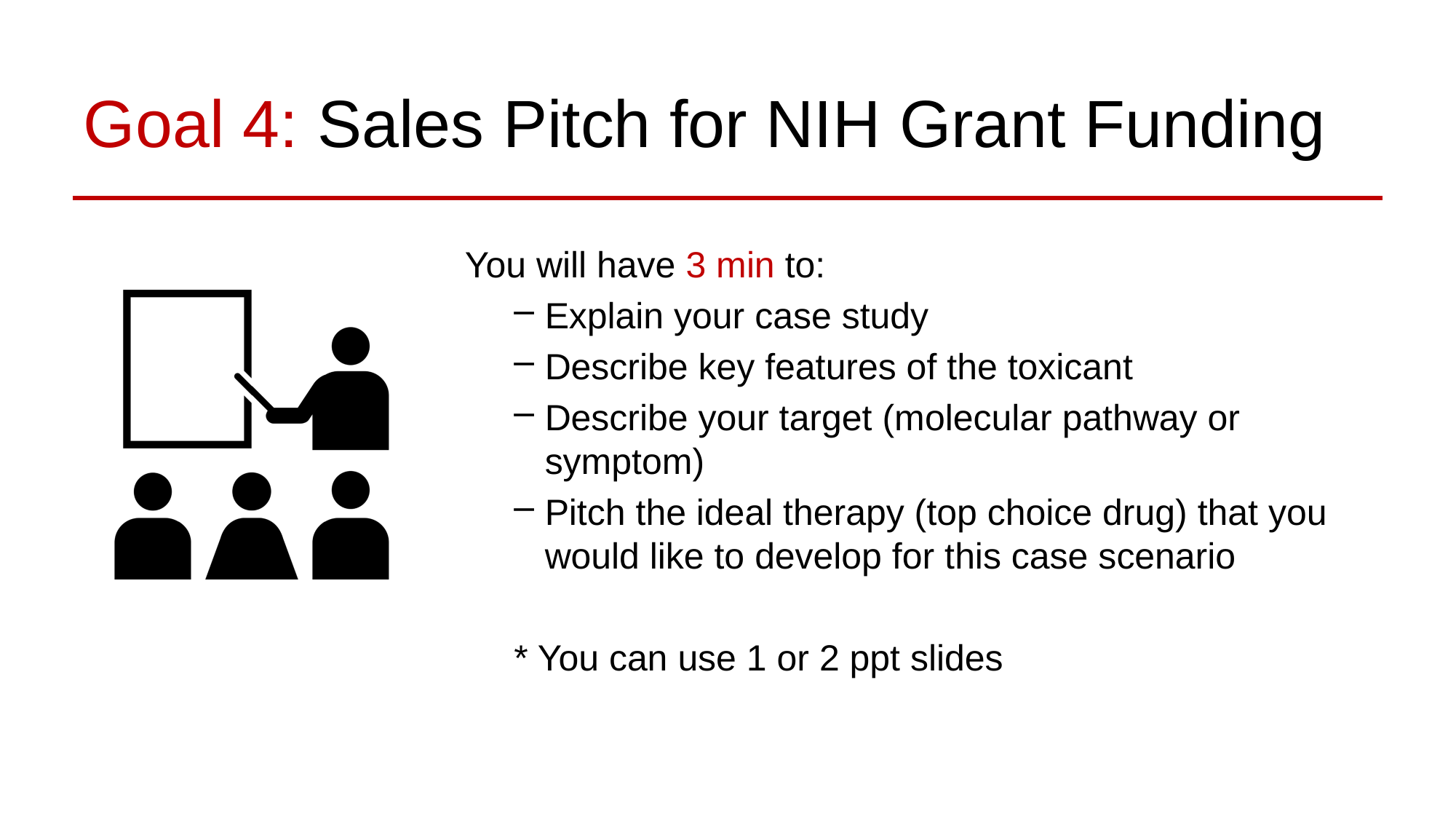

# Goal 4: Sales Pitch for NIH Grant Funding
You will have 3 min to:
Explain your case study
Describe key features of the toxicant
Describe your target (molecular pathway or symptom)
Pitch the ideal therapy (top choice drug) that you would like to develop for this case scenario
* You can use 1 or 2 ppt slides

## Slide 22
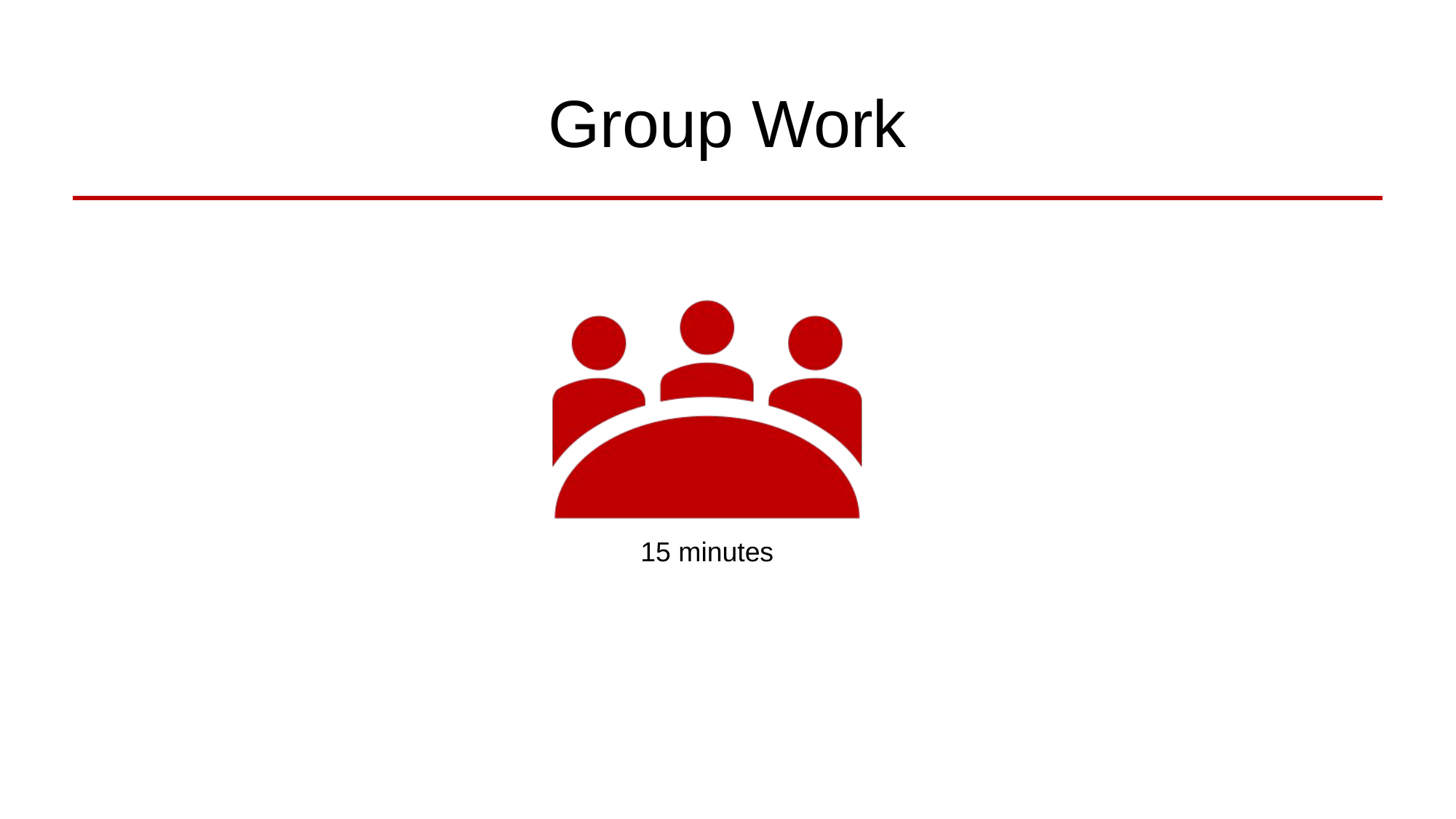

# Group Work
15 minutes

## Slide 23
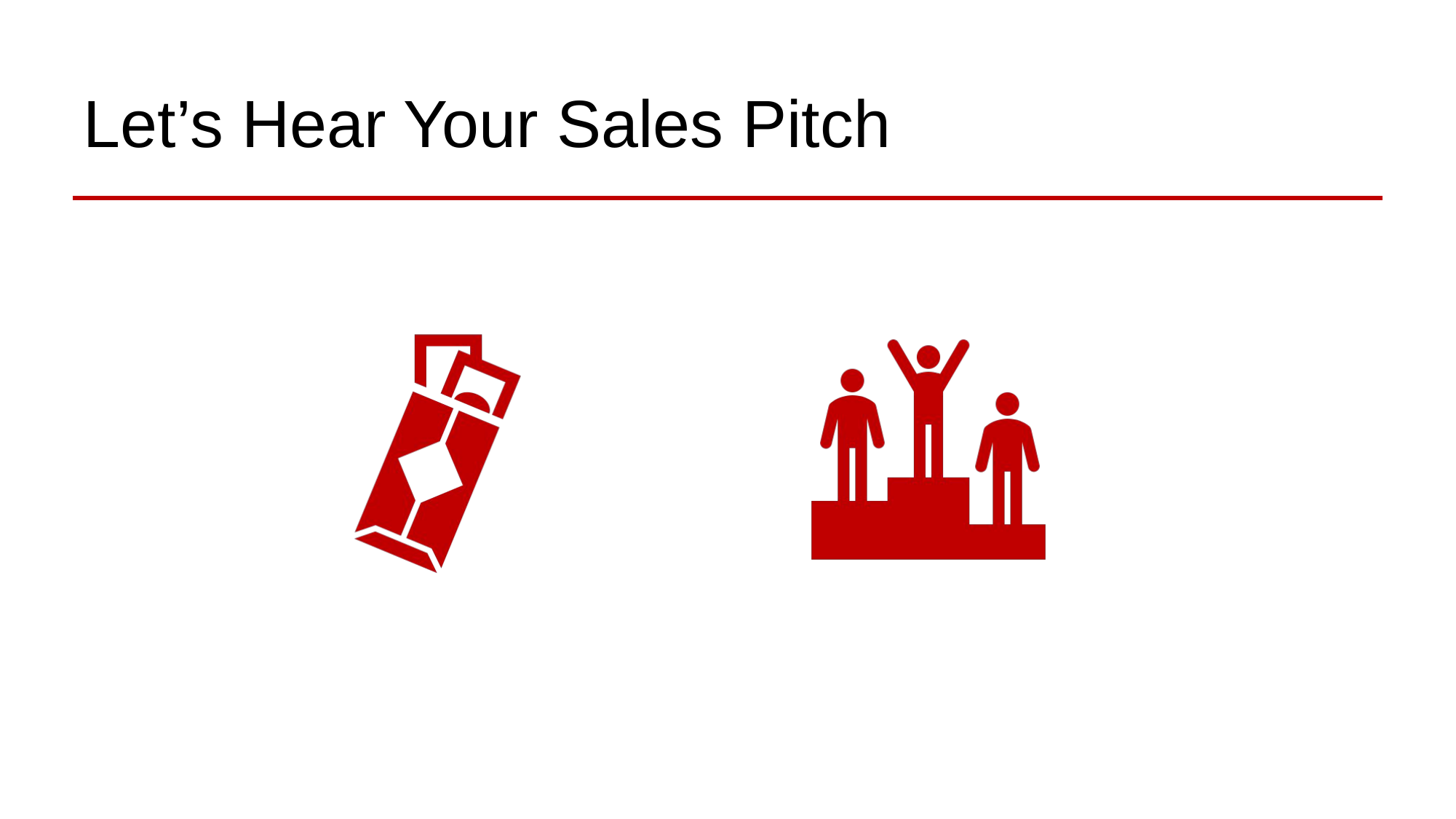

# Let’s Hear Your Sales Pitch
