# Supplementary material for: Repurposing FDA‐approved drugs to treat chemical weapon toxicities: Interactive case studies for trainees
Source: Pharmacol Res Perspect. 2024 Jul 4;12(4):e1229. doi: 10.1002/prp2.1229 (PMC11223991; doi:10.1002/prp2.1229)
Supplement: Supplementary file 3 — File S3. [file PRP2-12-e1229-s004.zip › Supporting File S3 - Case 1.pptx]

## Slide 1
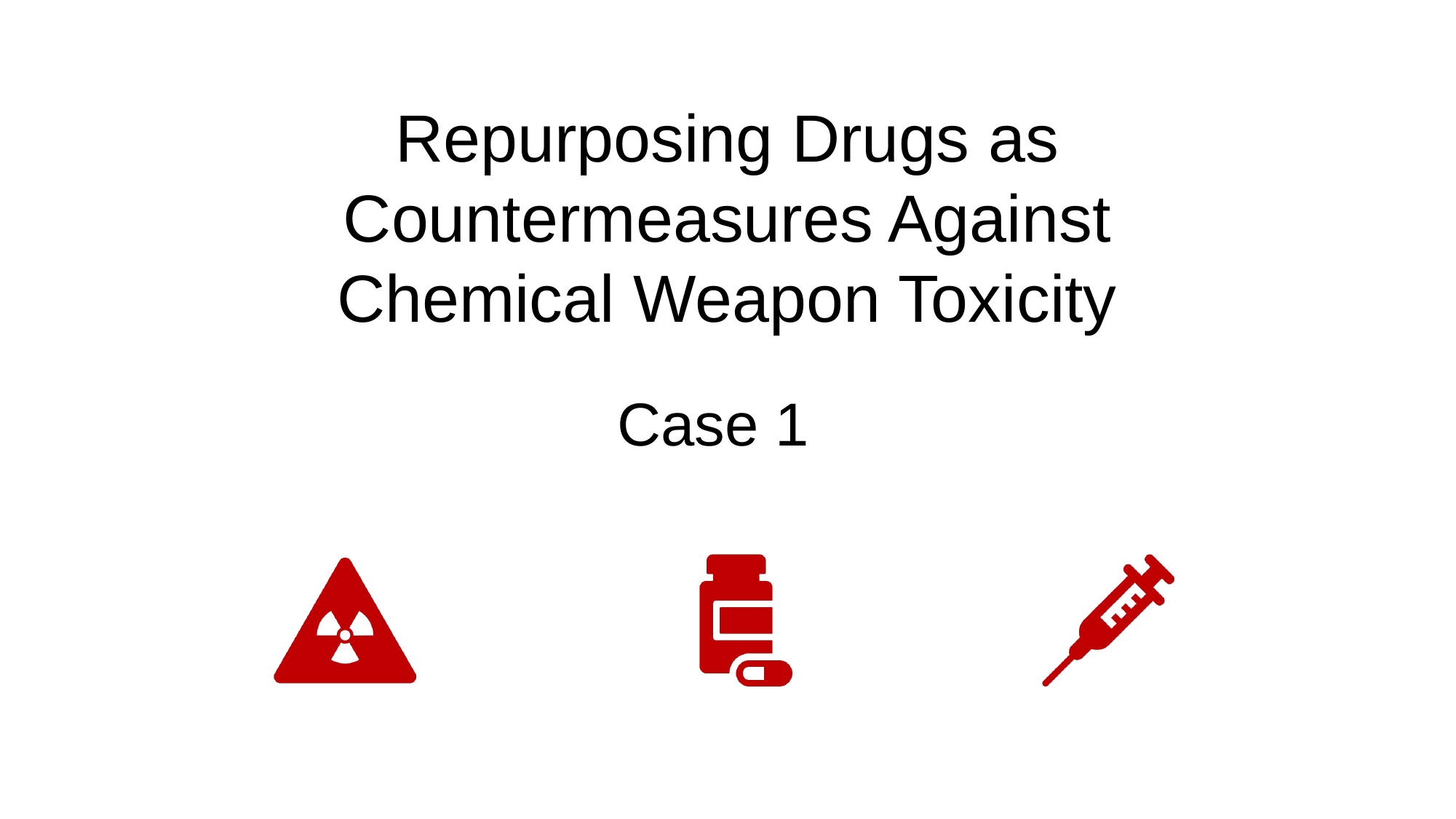

Repurposing Drugs as Countermeasures Against Chemical Weapon Toxicity
Case 1

## Slide 2
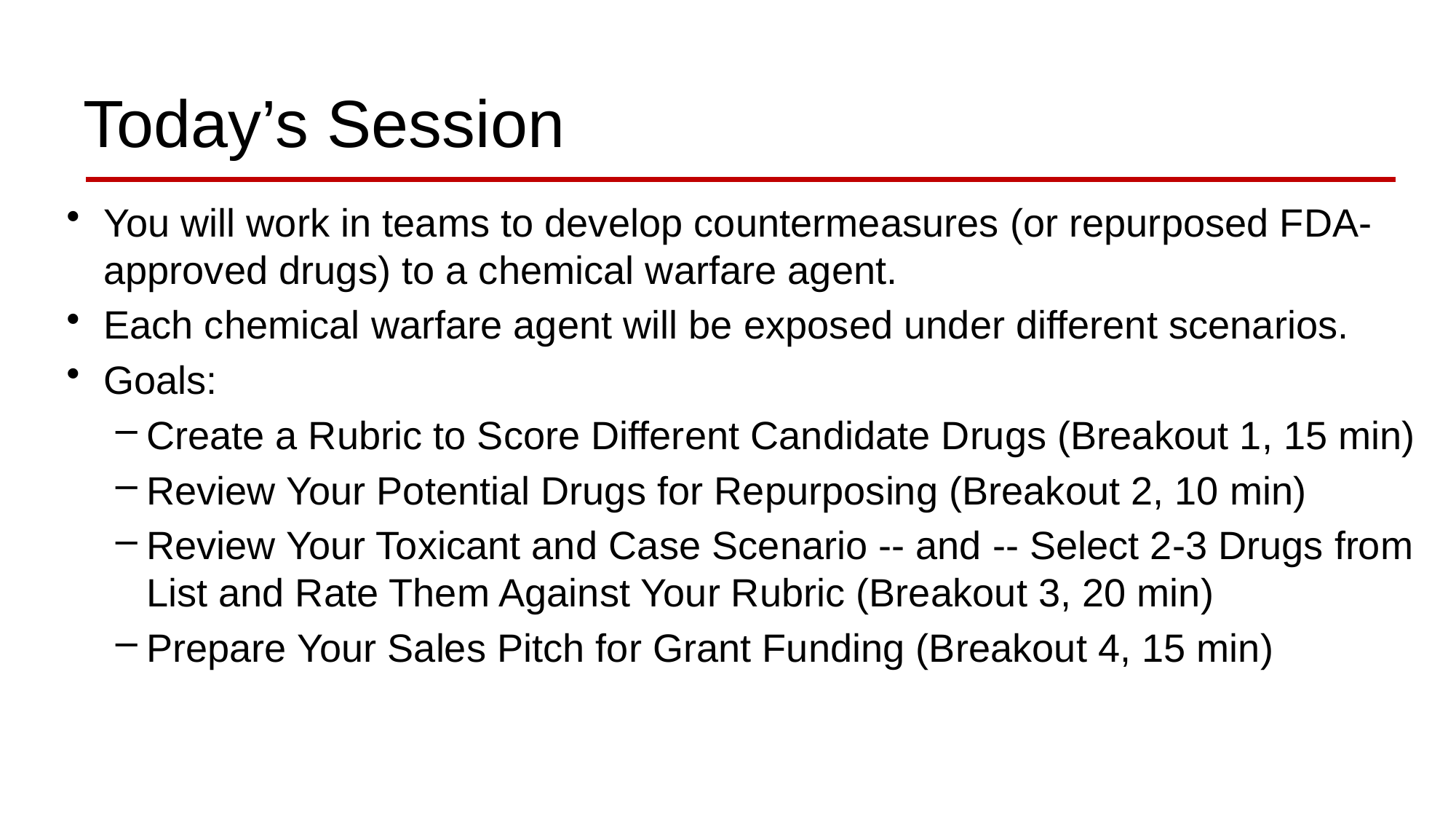

# Today’s Session
You will work in teams to develop countermeasures (or repurposed FDA-approved drugs) to a chemical warfare agent.
Each chemical warfare agent will be exposed under different scenarios.
Goals:
Create a Rubric to Score Different Candidate Drugs (Breakout 1, 15 min)
Review Your Potential Drugs for Repurposing (Breakout 2, 10 min)
Review Your Toxicant and Case Scenario -- and -- Select 2-3 Drugs from List and Rate Them Against Your Rubric (Breakout 3, 20 min)
Prepare Your Sales Pitch for Grant Funding (Breakout 4, 15 min)

## Slide 3
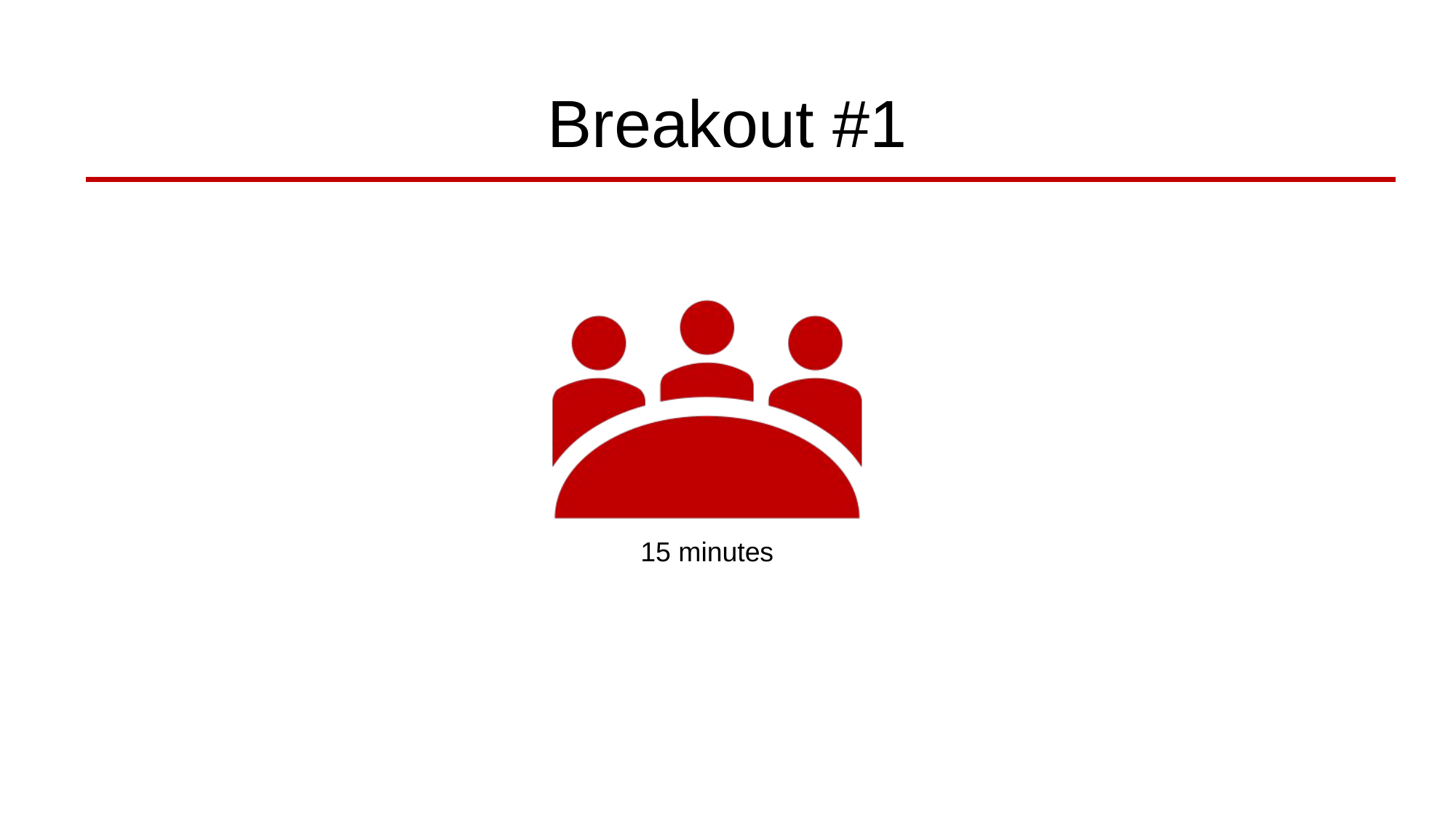

# Breakout #1
15 minutes

## Slide 4
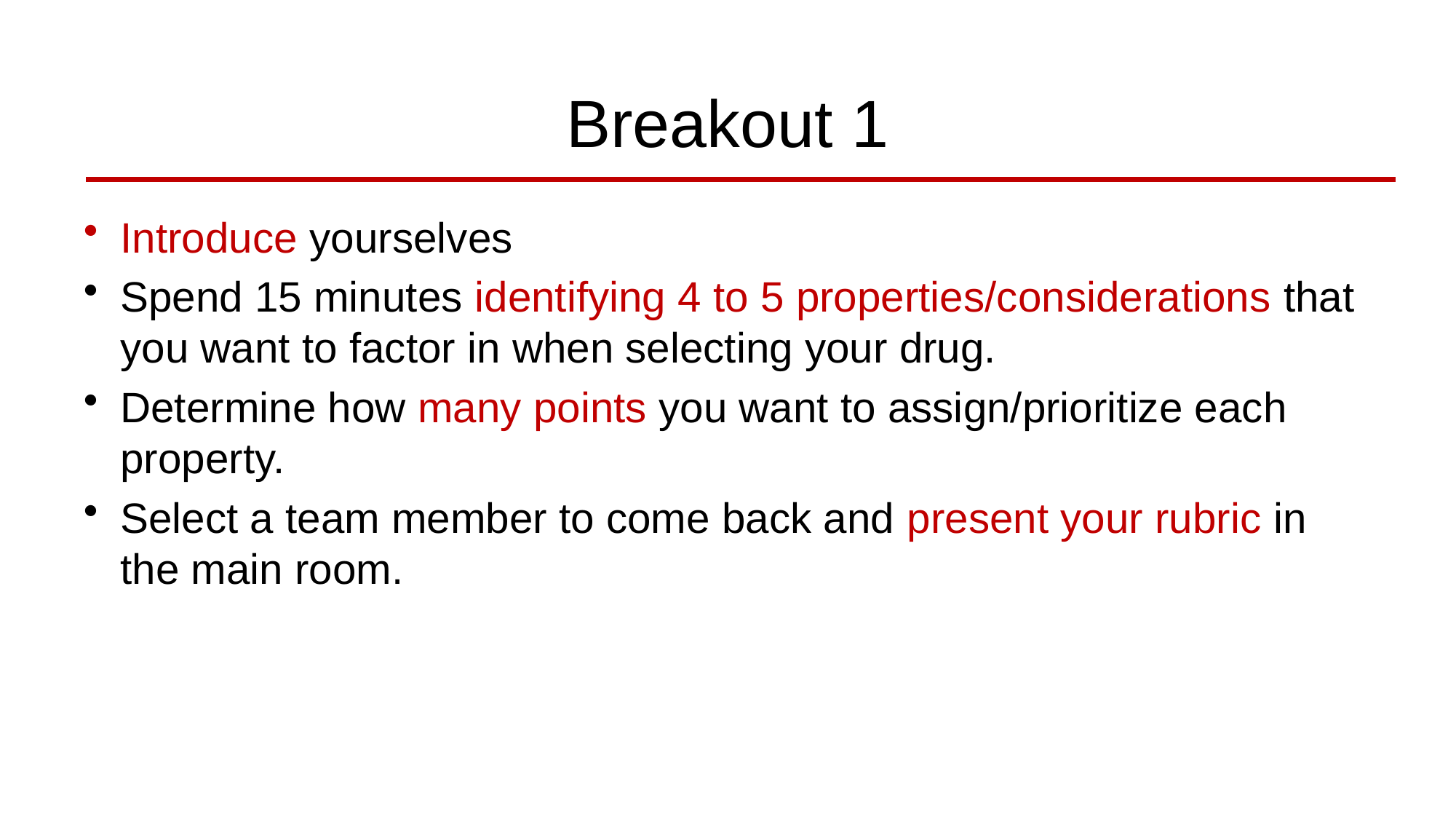

# Breakout 1
Introduce yourselves
Spend 15 minutes identifying 4 to 5 properties/considerations that you want to factor in when selecting your drug.
Determine how many points you want to assign/prioritize each property.
Select a team member to come back and present your rubric in the main room.

## Slide 5
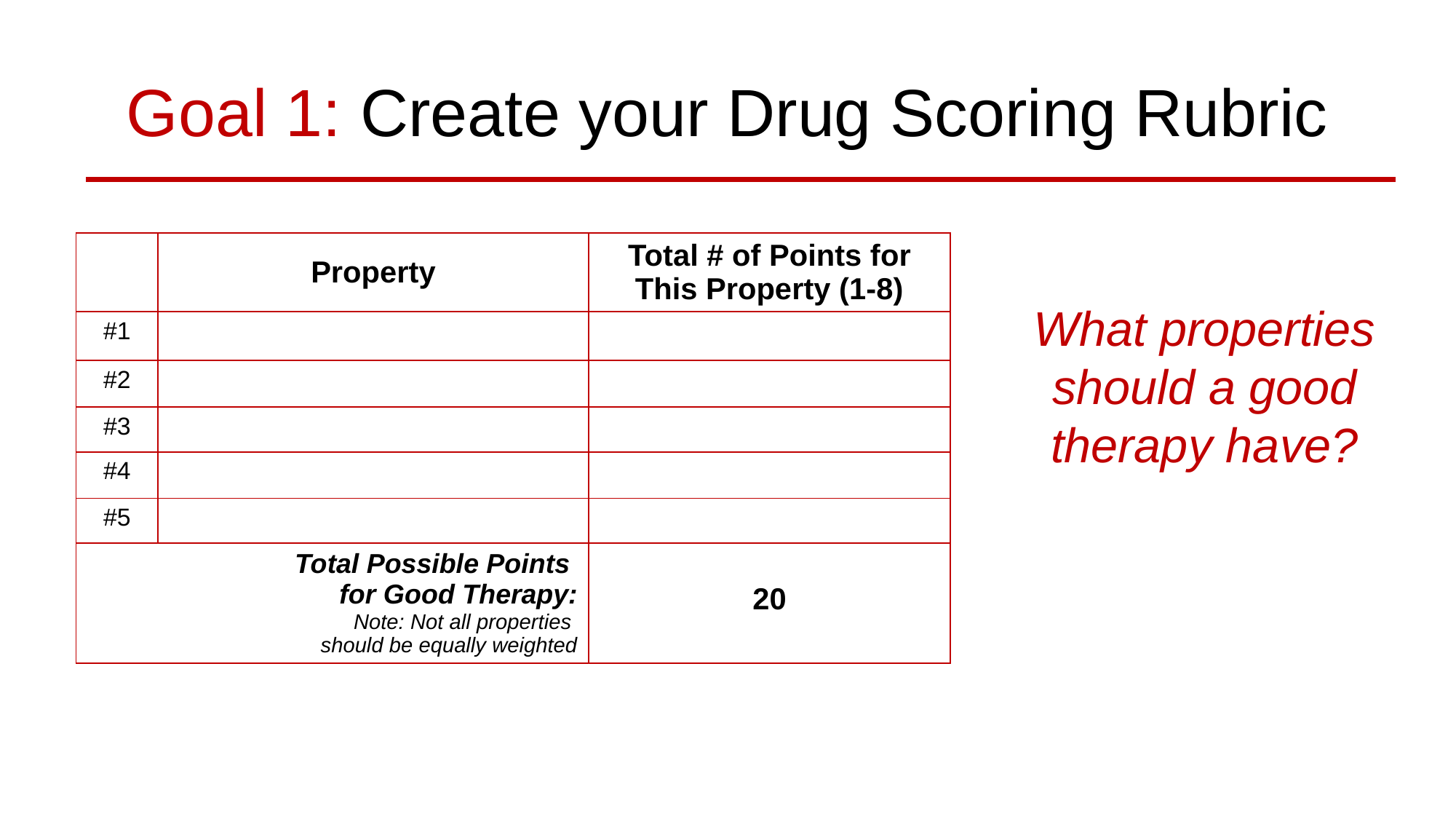

# Goal 1: Create your Drug Scoring Rubric
| | Property | Total # of Points for This Property (1-8) |
| --- | --- | --- |
| #1 | | |
| #2 | | |
| #3 | | |
| #4 | | |
| #5 | | |
| Total Possible Points for Good Therapy: Note: Not all properties should be equally weighted | | 20 |
What properties should a good therapy have?

## Slide 6
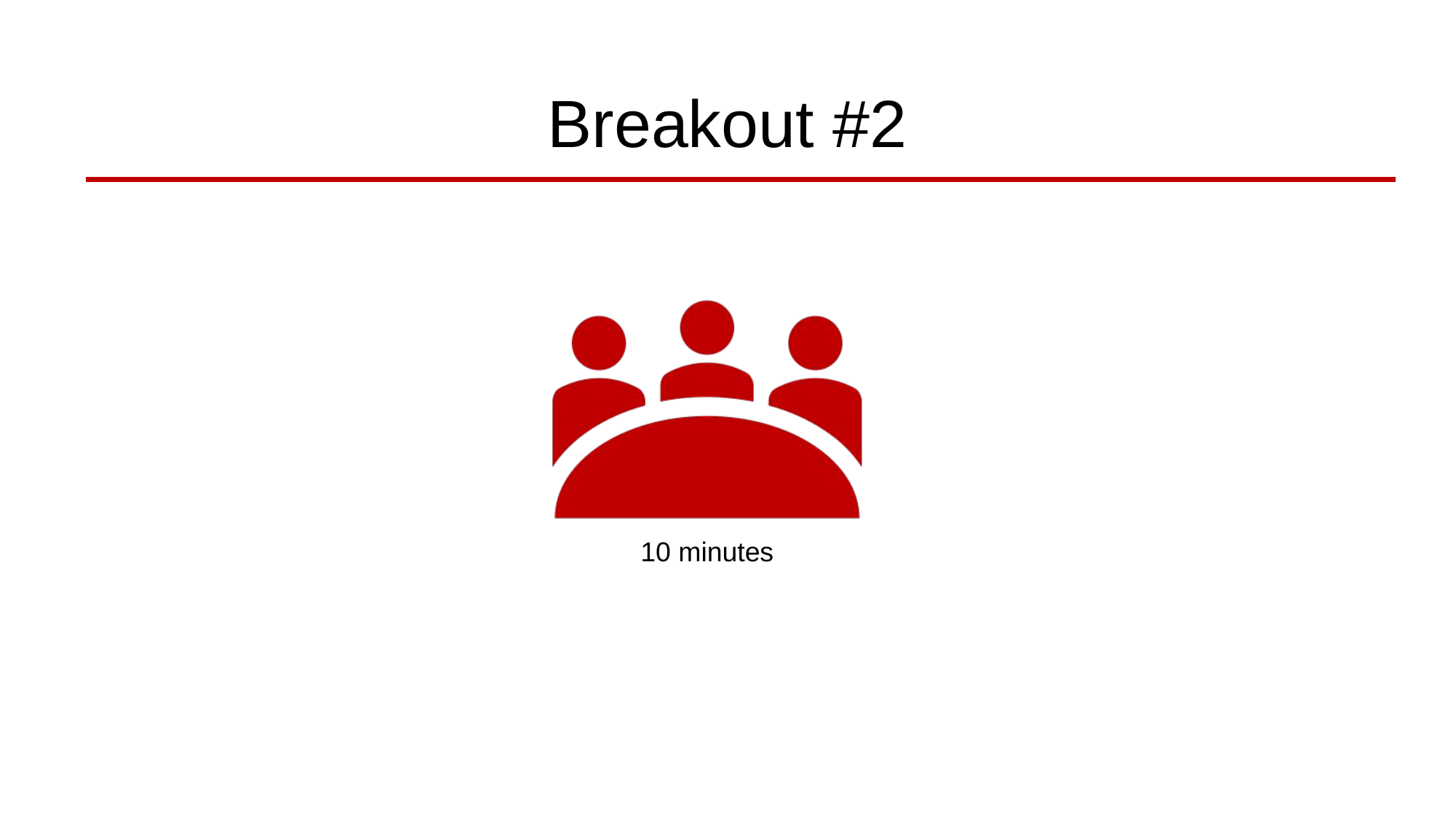

# Breakout #2
10 minutes

## Slide 7
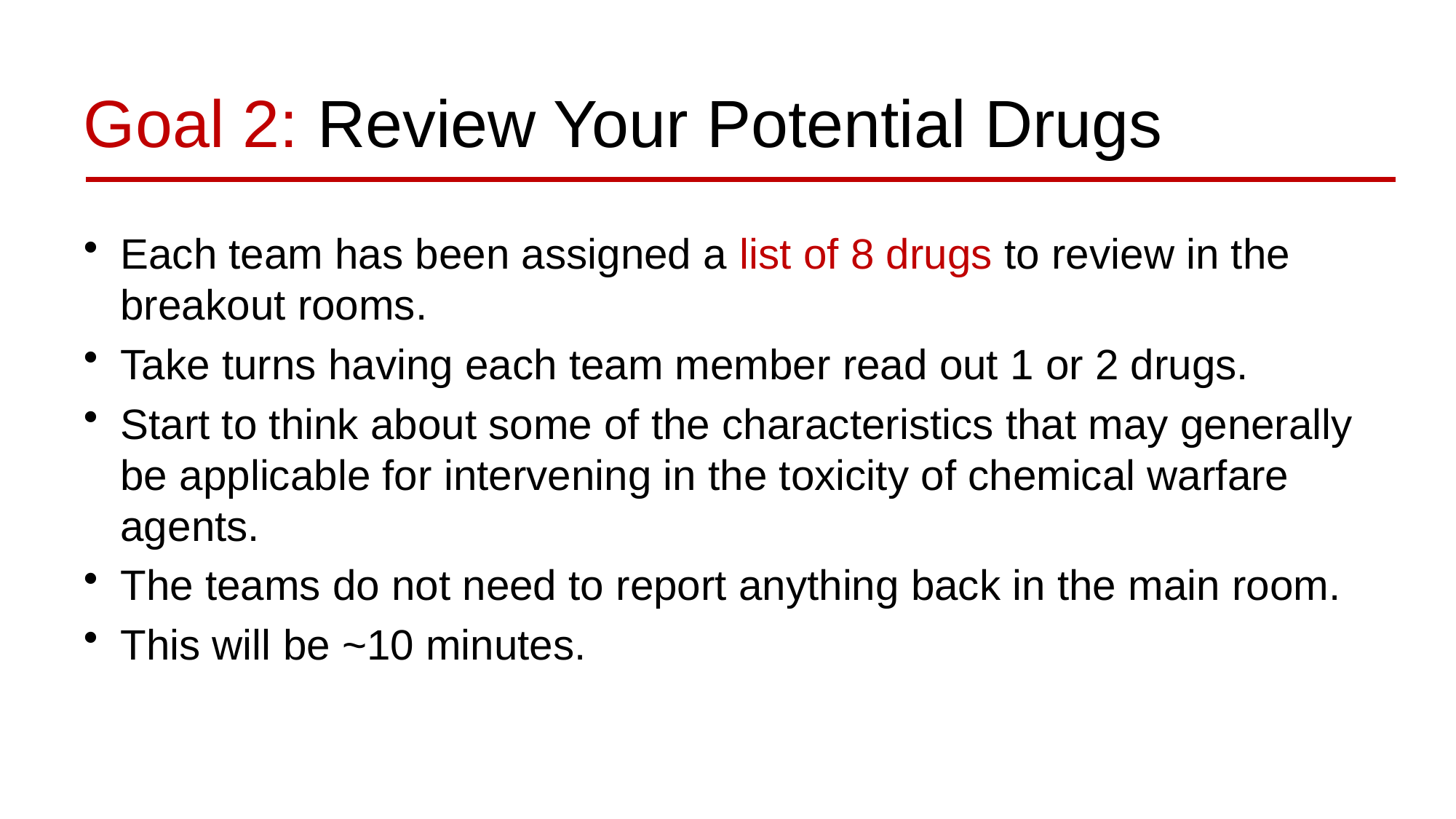

# Goal 2: Review Your Potential Drugs
Each team has been assigned a list of 8 drugs to review in the breakout rooms.
Take turns having each team member read out 1 or 2 drugs.
Start to think about some of the characteristics that may generally be applicable for intervening in the toxicity of chemical warfare agents.
The teams do not need to report anything back in the main room.
This will be ~10 minutes.

## Slide 8
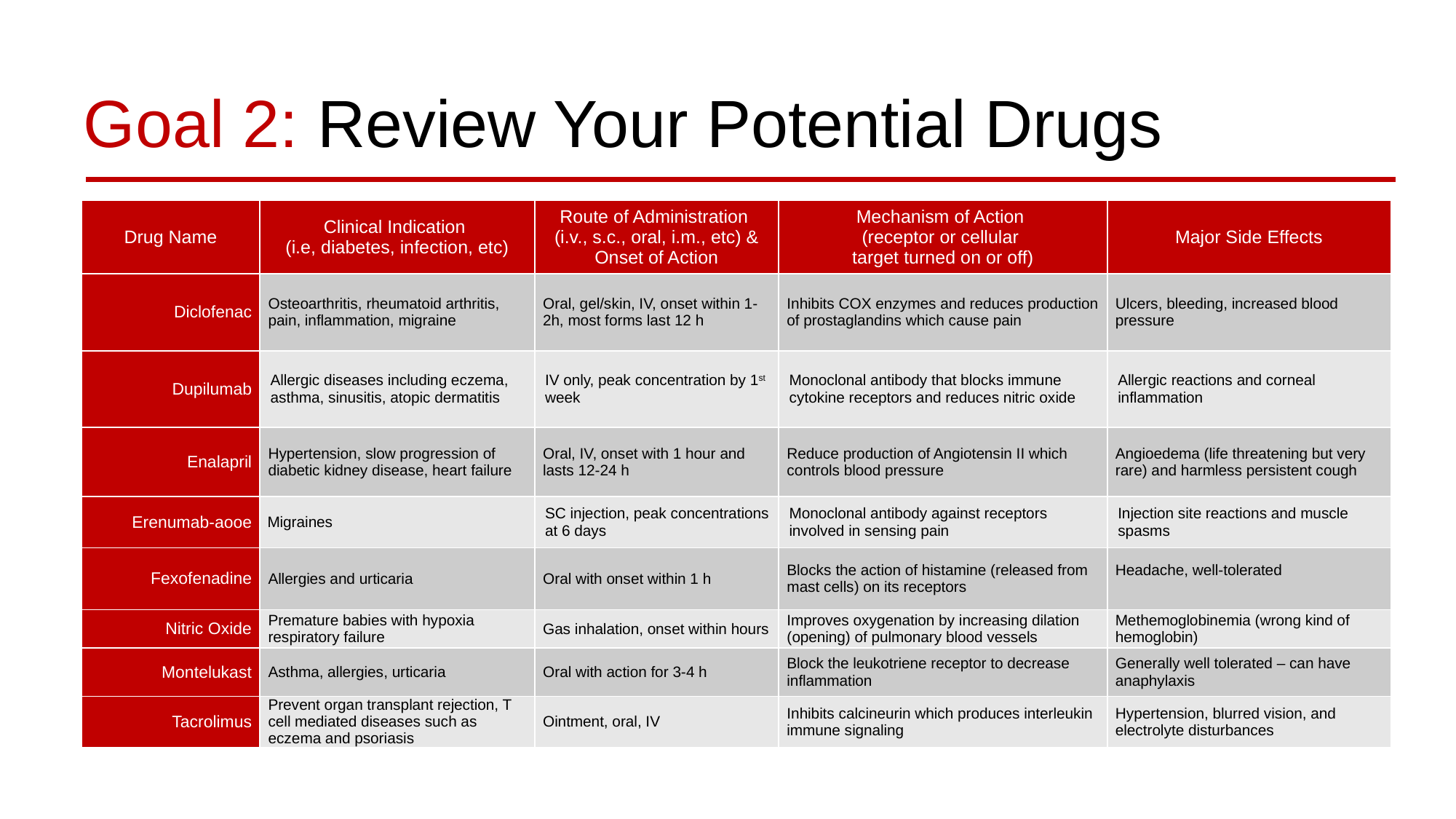

# Goal 2: Review Your Potential Drugs
| Drug Name | Clinical Indication (i.e, diabetes, infection, etc) | Route of Administration (i.v., s.c., oral, i.m., etc) & Onset of Action | Mechanism of Action (receptor or cellular target turned on or off) | Major Side Effects |
| --- | --- | --- | --- | --- |
| Diclofenac | Osteoarthritis, rheumatoid arthritis, pain, inflammation, migraine | Oral, gel/skin, IV, onset within 1-2h, most forms last 12 h | Inhibits COX enzymes and reduces production of prostaglandins which cause pain | Ulcers, bleeding, increased blood pressure |
| Dupilumab | Allergic diseases including eczema, asthma, sinusitis, atopic dermatitis | IV only, peak concentration by 1st week | Monoclonal antibody that blocks immune cytokine receptors and reduces nitric oxide | Allergic reactions and corneal inflammation |
| Enalapril | Hypertension, slow progression of diabetic kidney disease, heart failure | Oral, IV, onset with 1 hour and lasts 12-24 h | Reduce production of Angiotensin II which controls blood pressure | Angioedema (life threatening but very rare) and harmless persistent cough |
| Erenumab-aooe | Migraines | SC injection, peak concentrations at 6 days | Monoclonal antibody against receptors involved in sensing pain | Injection site reactions and muscle spasms |
| Fexofenadine | Allergies and urticaria | Oral with onset within 1 h | Blocks the action of histamine (released from mast cells) on its receptors | Headache, well-tolerated |
| Nitric Oxide | Premature babies with hypoxia respiratory failure | Gas inhalation, onset within hours | Improves oxygenation by increasing dilation (opening) of pulmonary blood vessels | Methemoglobinemia (wrong kind of hemoglobin) |
| Montelukast | Asthma, allergies, urticaria | Oral with action for 3-4 h | Block the leukotriene receptor to decrease inflammation | Generally well tolerated – can have anaphylaxis |
| Tacrolimus | Prevent organ transplant rejection, T cell mediated diseases such as eczema and psoriasis | Ointment, oral, IV | Inhibits calcineurin which produces interleukin immune signaling | Hypertension, blurred vision, and electrolyte disturbances |

## Slide 9
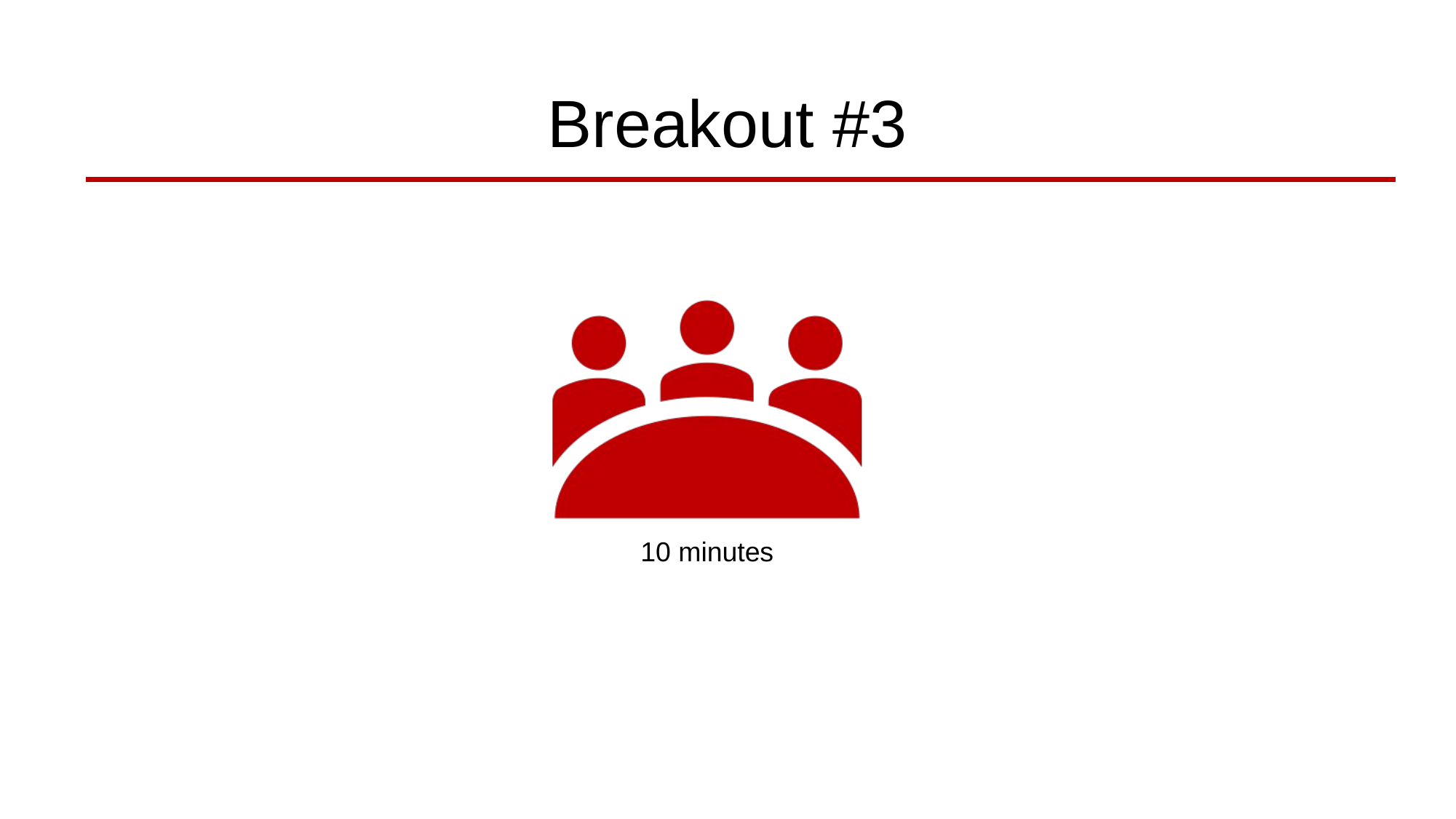

# Breakout #3
10 minutes

## Slide 10
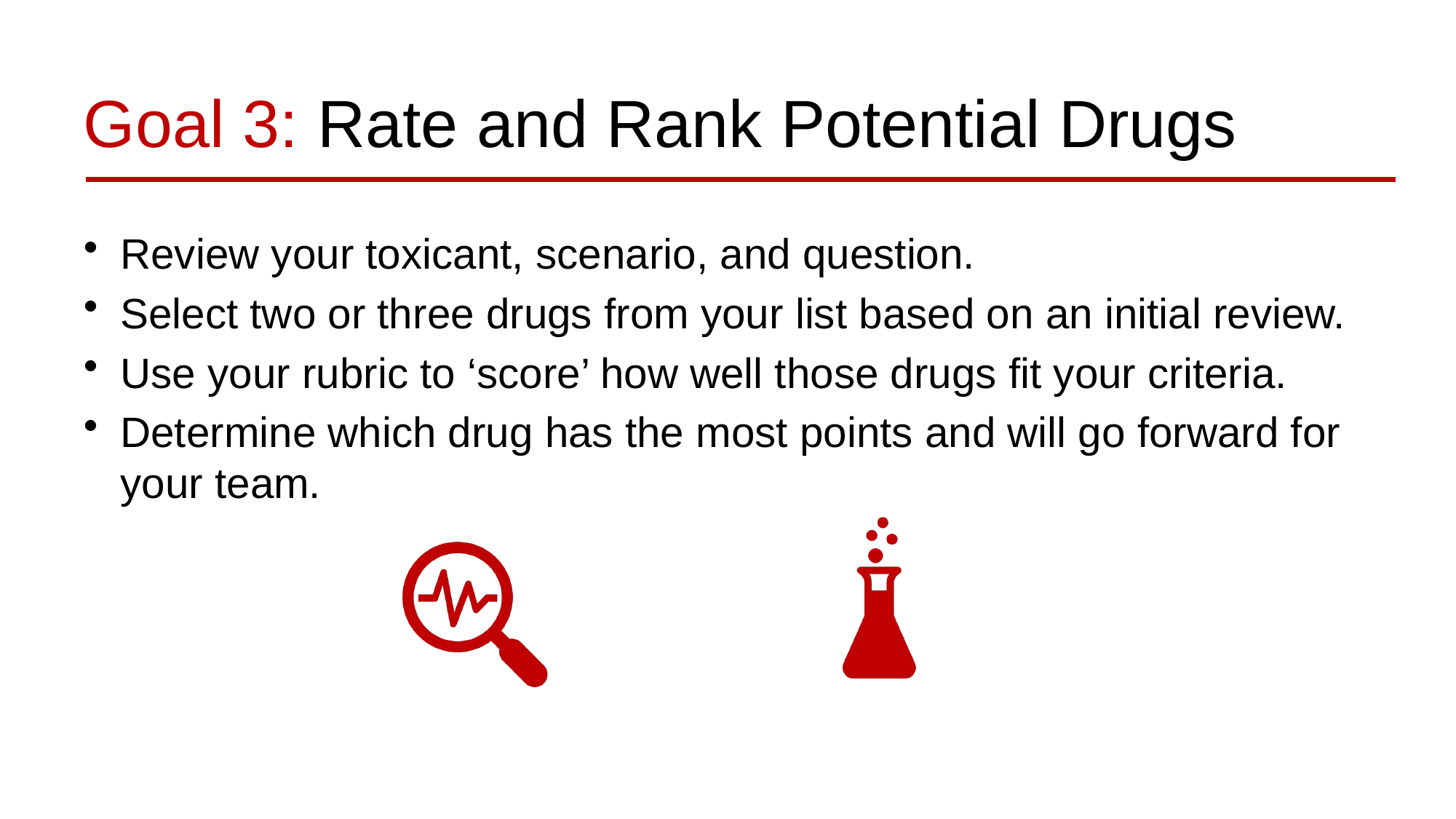

# Goal 3: Rate and Rank Potential Drugs
Review your toxicant, scenario, and question.
Select two or three drugs from your list based on an initial review.
Use your rubric to ‘score’ how well those drugs fit your criteria.
Determine which drug has the most points and will go forward for your team.

## Slide 11
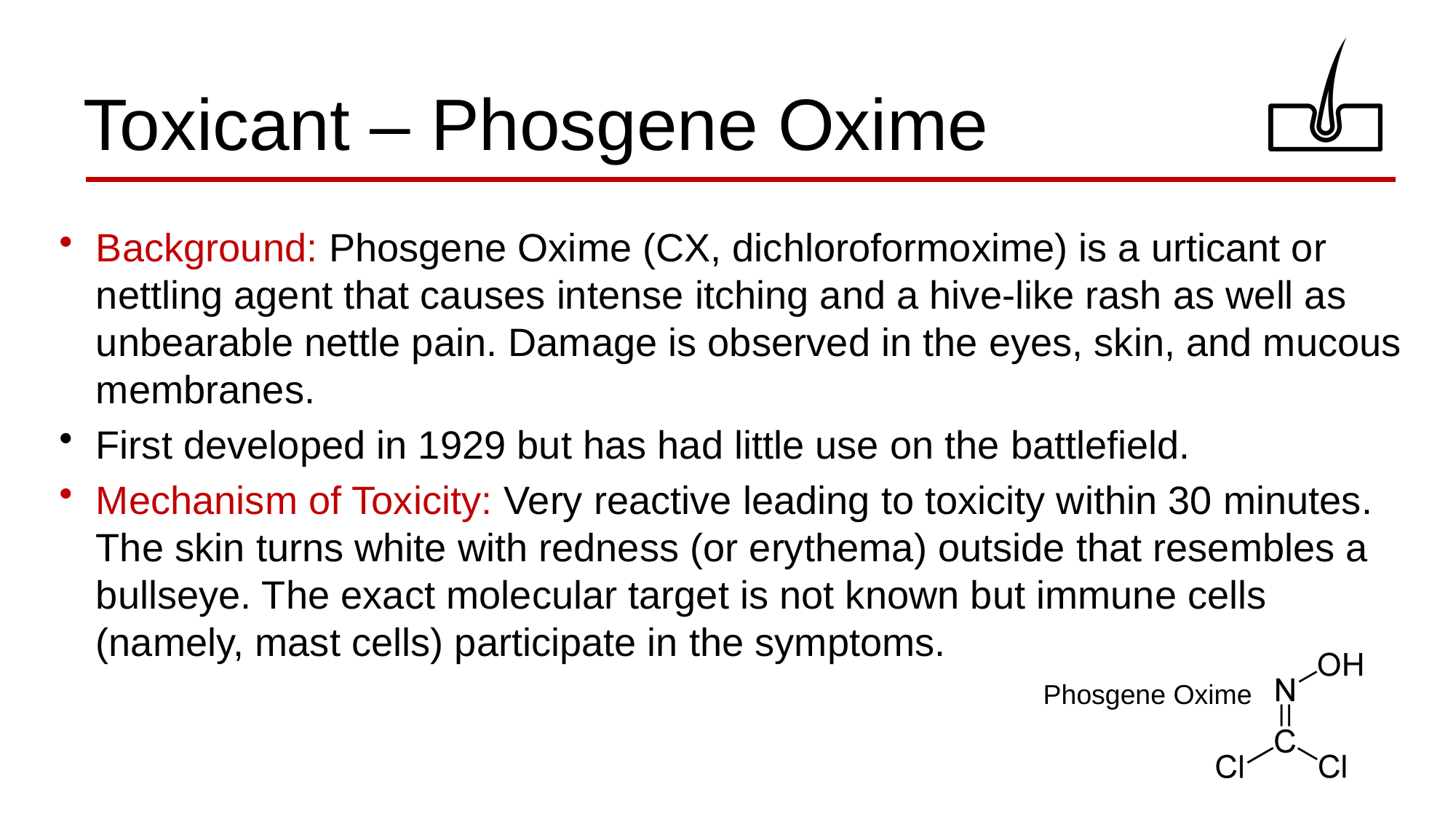

# Toxicant – Phosgene Oxime
Background: Phosgene Oxime (CX, dichloroformoxime) is a urticant or nettling agent that causes intense itching and a hive-like rash as well as unbearable nettle pain. Damage is observed in the eyes, skin, and mucous membranes.
First developed in 1929 but has had little use on the battlefield.
Mechanism of Toxicity: Very reactive leading to toxicity within 30 minutes. The skin turns white with redness (or erythema) outside that resembles a bullseye. The exact molecular target is not known but immune cells (namely, mast cells) participate in the symptoms.
Phosgene Oxime

## Slide 12
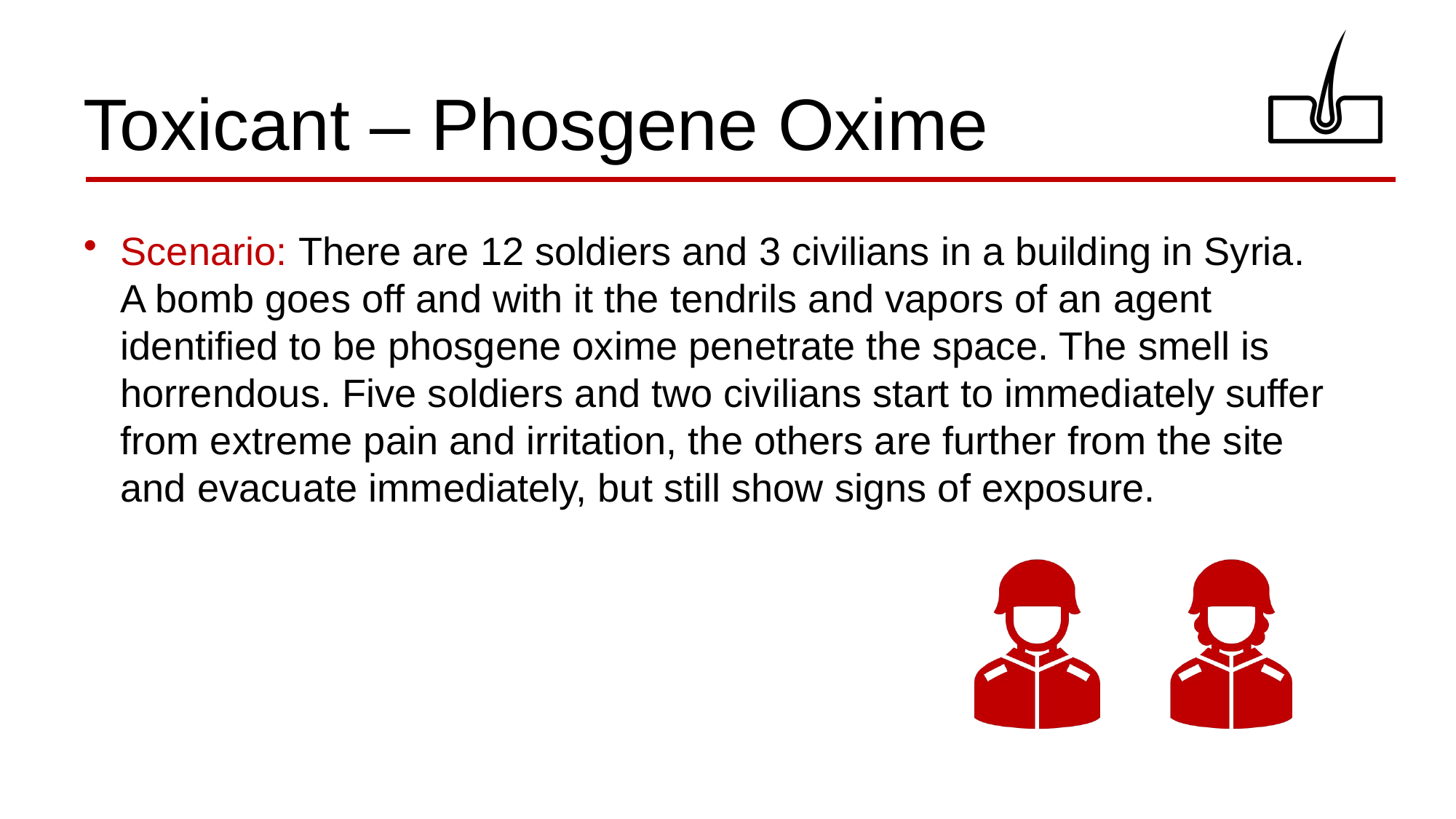

# Toxicant – Phosgene Oxime
Scenario: There are 12 soldiers and 3 civilians in a building in Syria. A bomb goes off and with it the tendrils and vapors of an agent identified to be phosgene oxime penetrate the space. The smell is horrendous. Five soldiers and two civilians start to immediately suffer from extreme pain and irritation, the others are further from the site and evacuate immediately, but still show signs of exposure.

## Slide 13
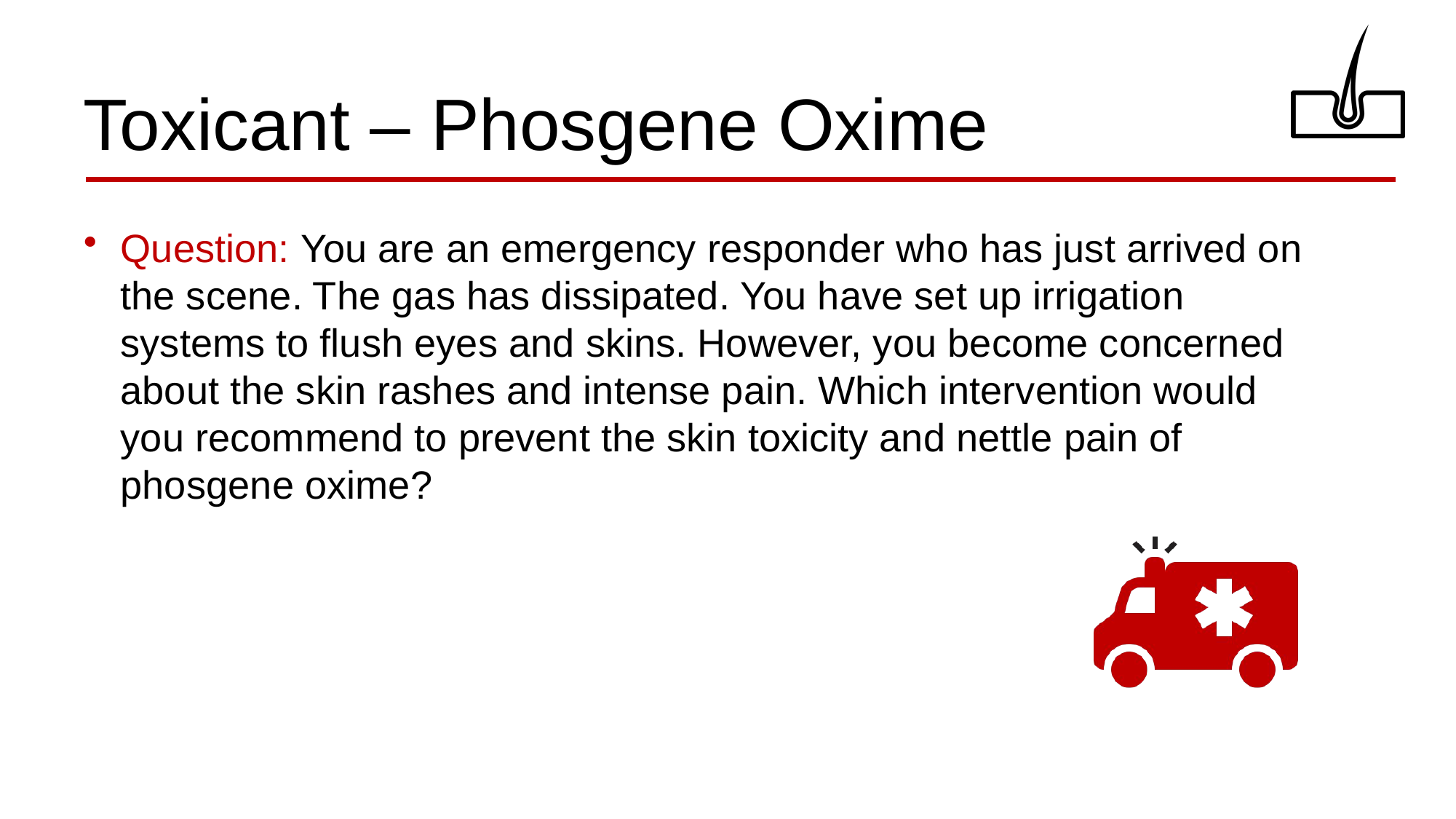

# Toxicant – Phosgene Oxime
Question: You are an emergency responder who has just arrived on the scene. The gas has dissipated. You have set up irrigation systems to flush eyes and skins. However, you become concerned about the skin rashes and intense pain. Which intervention would you recommend to prevent the skin toxicity and nettle pain of phosgene oxime?

## Slide 14
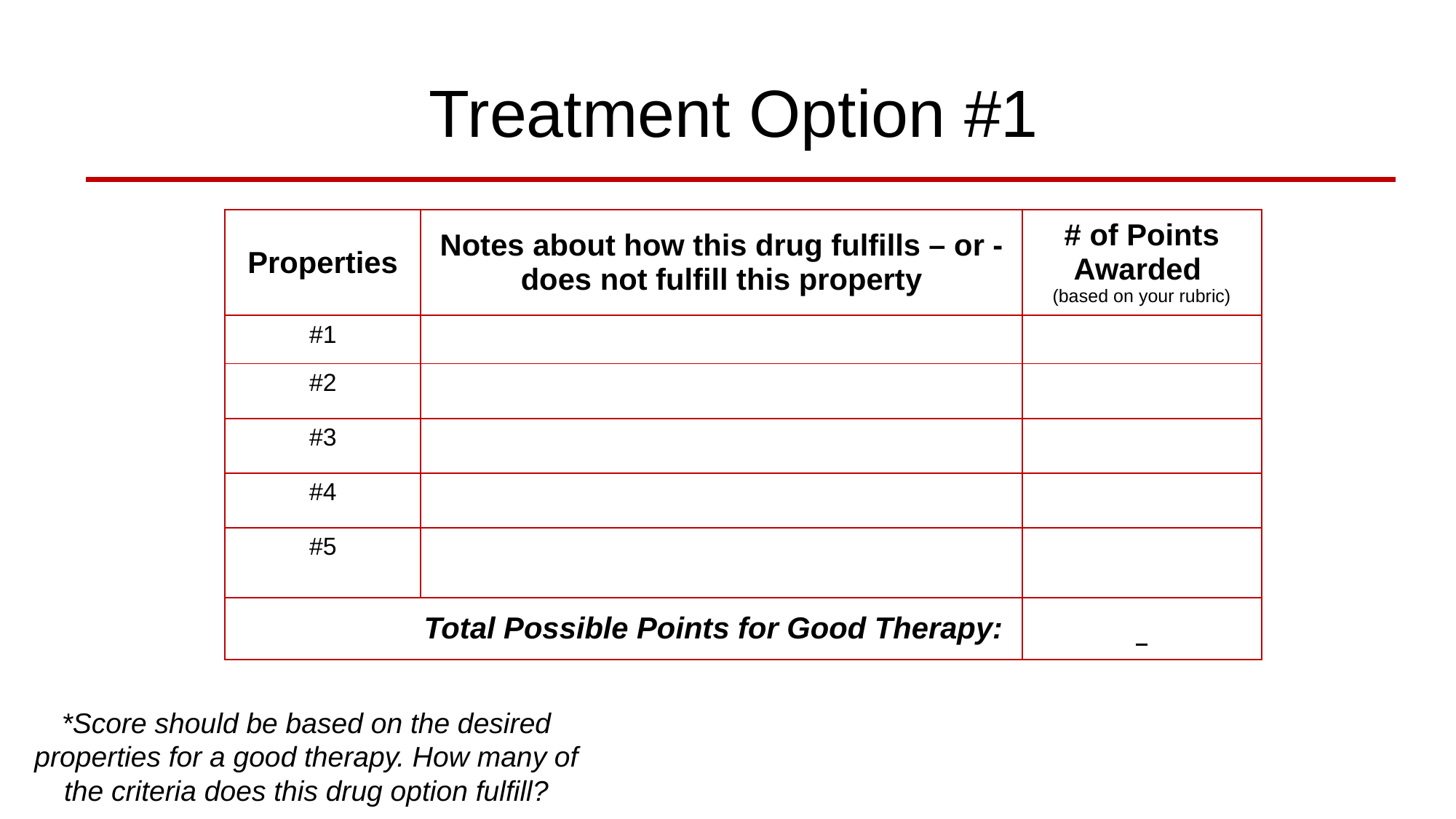

# Treatment Option #1
| Properties | Notes about how this drug fulfills – or -does not fulfill this property | # of Points Awarded (based on your rubric) |
| --- | --- | --- |
| #1 | | |
| #2 | | |
| #3 | | |
| #4 | | |
| #5 | | |
| Total Possible Points for Good Therapy: | | |
*Score should be based on the desired properties for a good therapy. How many of the criteria does this drug option fulfill?

## Slide 15
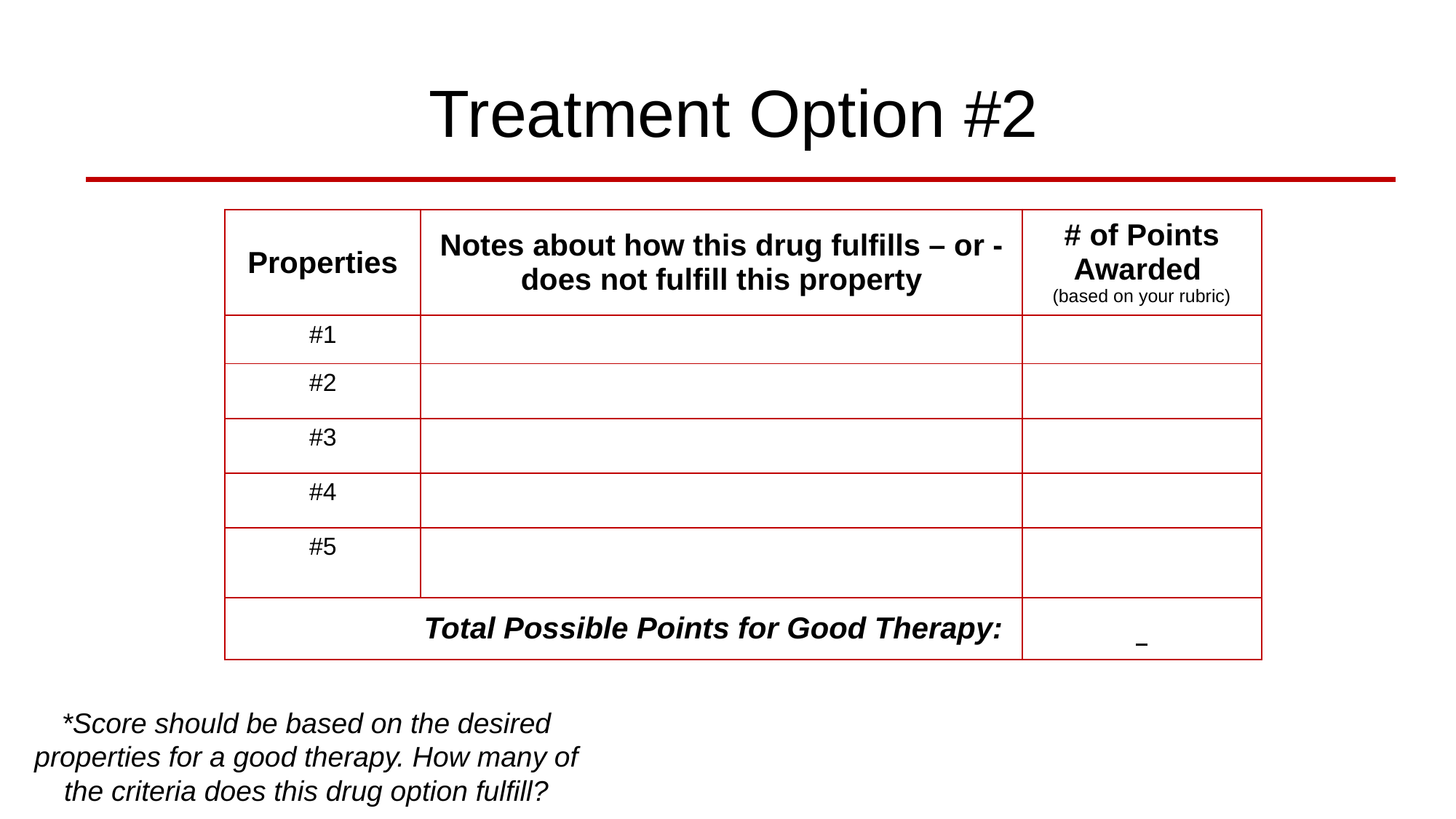

# Treatment Option #2
| Properties | Notes about how this drug fulfills – or -does not fulfill this property | # of Points Awarded (based on your rubric) |
| --- | --- | --- |
| #1 | | |
| #2 | | |
| #3 | | |
| #4 | | |
| #5 | | |
| Total Possible Points for Good Therapy: | | |
*Score should be based on the desired properties for a good therapy. How many of the criteria does this drug option fulfill?

## Slide 16
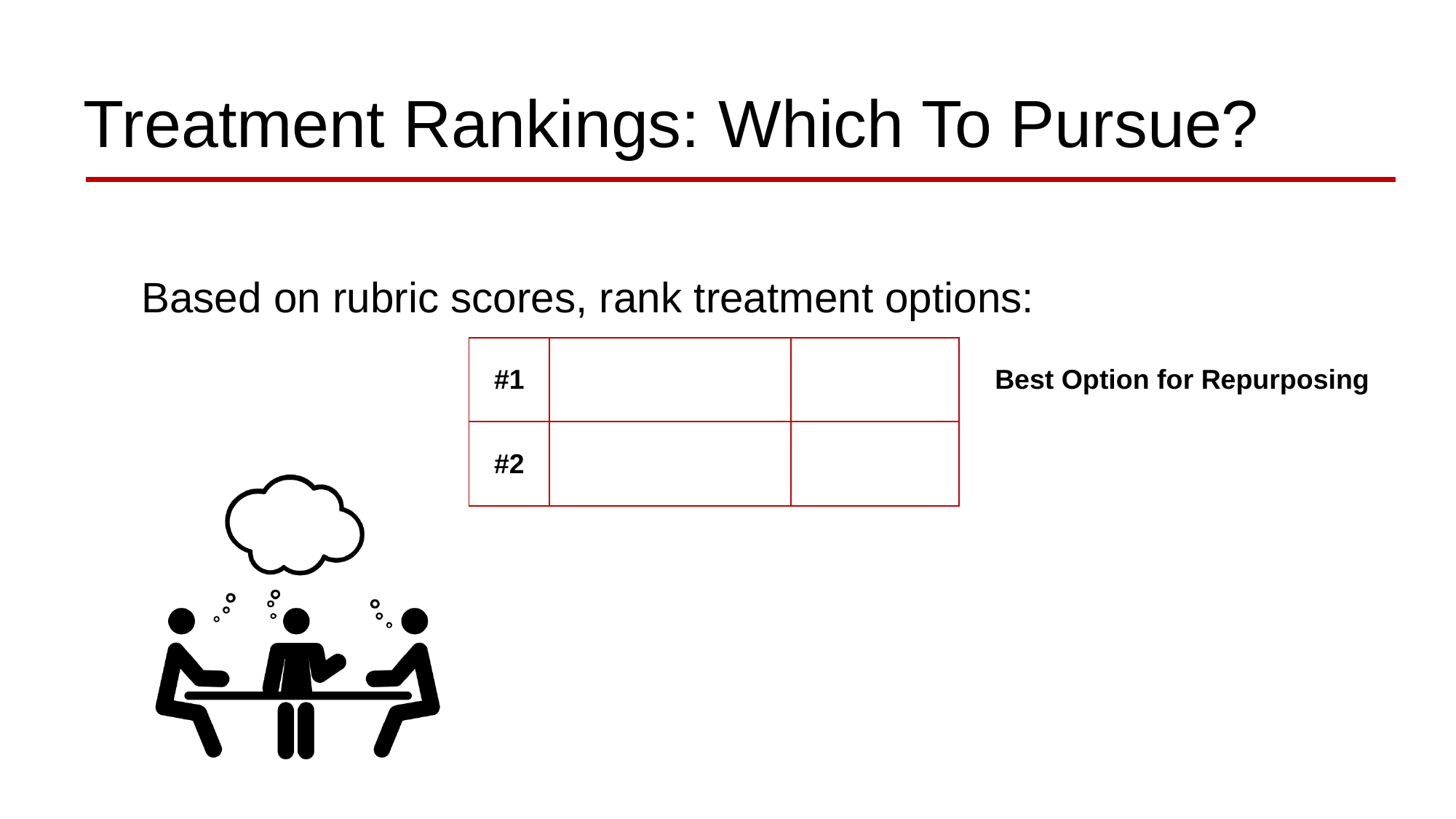

# Treatment Rankings: Which To Pursue?
Based on rubric scores, rank treatment options:
| #1 | | |
| --- | --- | --- |
| #2 | | |
Best Option for Repurposing

## Slide 17
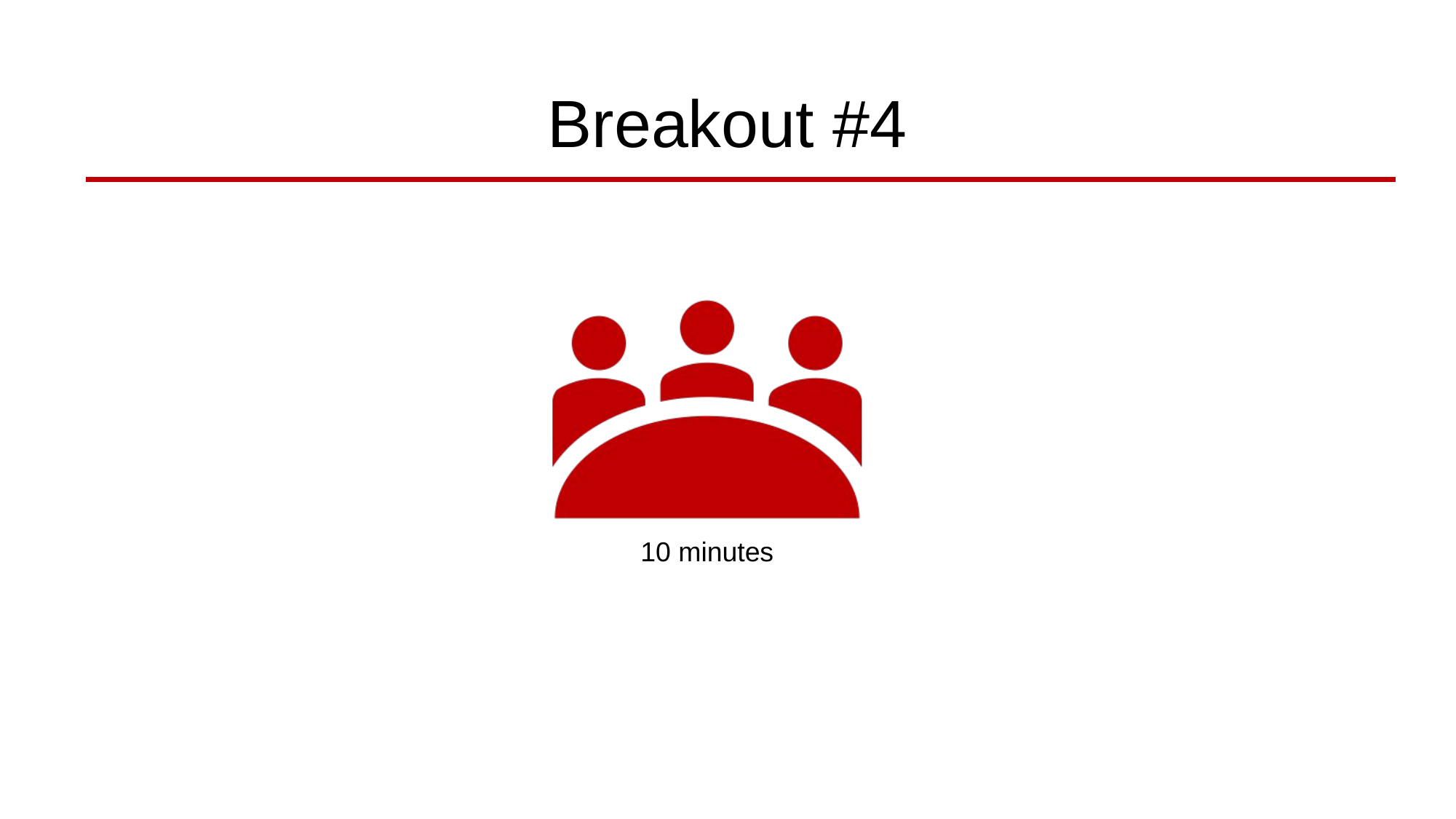

# Breakout #4
10 minutes

## Slide 18
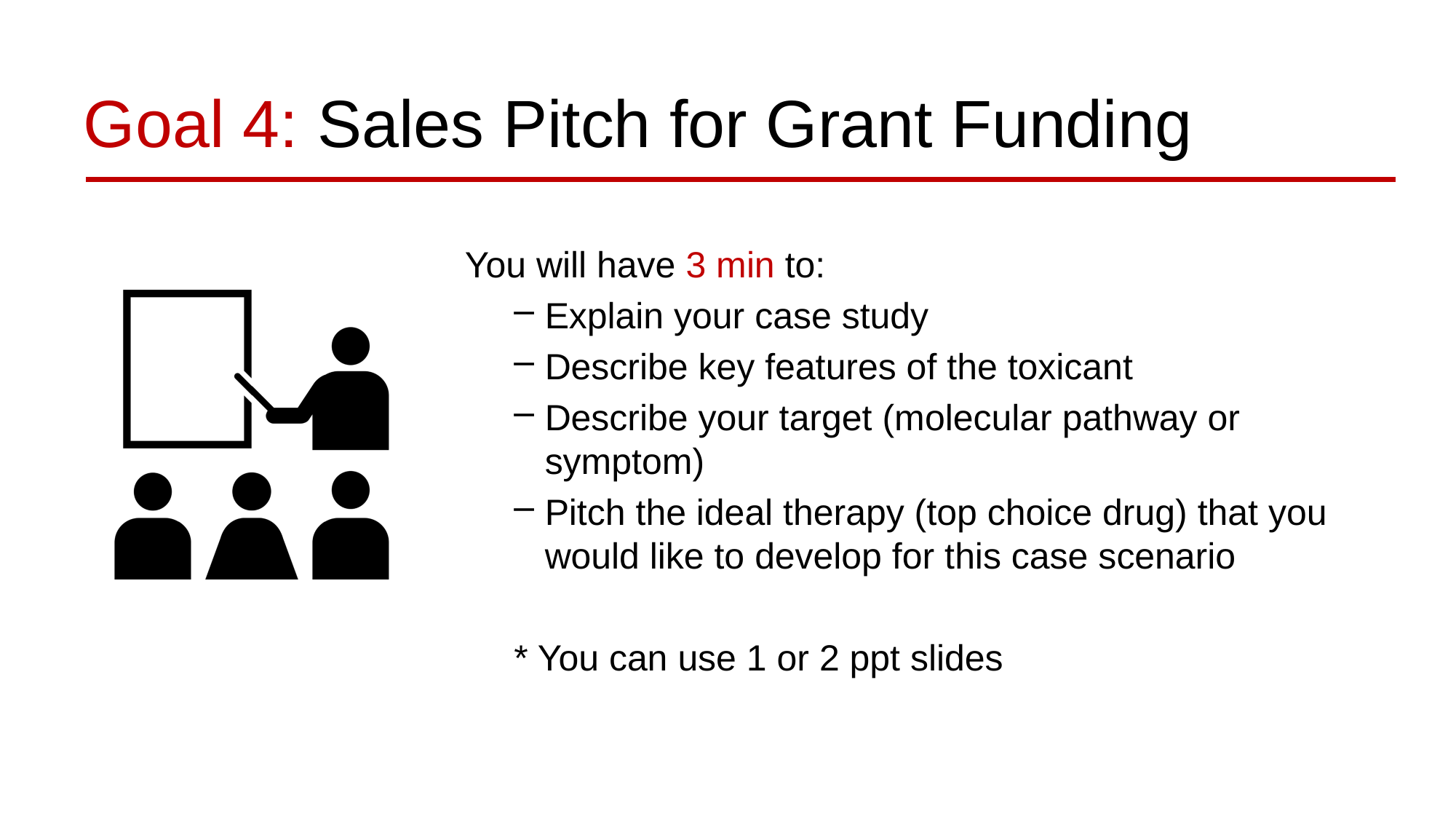

# Goal 4: Sales Pitch for Grant Funding
You will have 3 min to:
Explain your case study
Describe key features of the toxicant
Describe your target (molecular pathway or symptom)
Pitch the ideal therapy (top choice drug) that you would like to develop for this case scenario
* You can use 1 or 2 ppt slides
