# Supplementary material for: Repurposing FDA‐approved drugs to treat chemical weapon toxicities: Interactive case studies for trainees
Source: Pharmacol Res Perspect. 2024 Jul 4;12(4):e1229. doi: 10.1002/prp2.1229 (PMC11223991; doi:10.1002/prp2.1229)
Supplement: Supplementary file 3 — File S3. [file PRP2-12-e1229-s004.zip › Supporting File S4 - Case 2.pptx]

## Slide 1
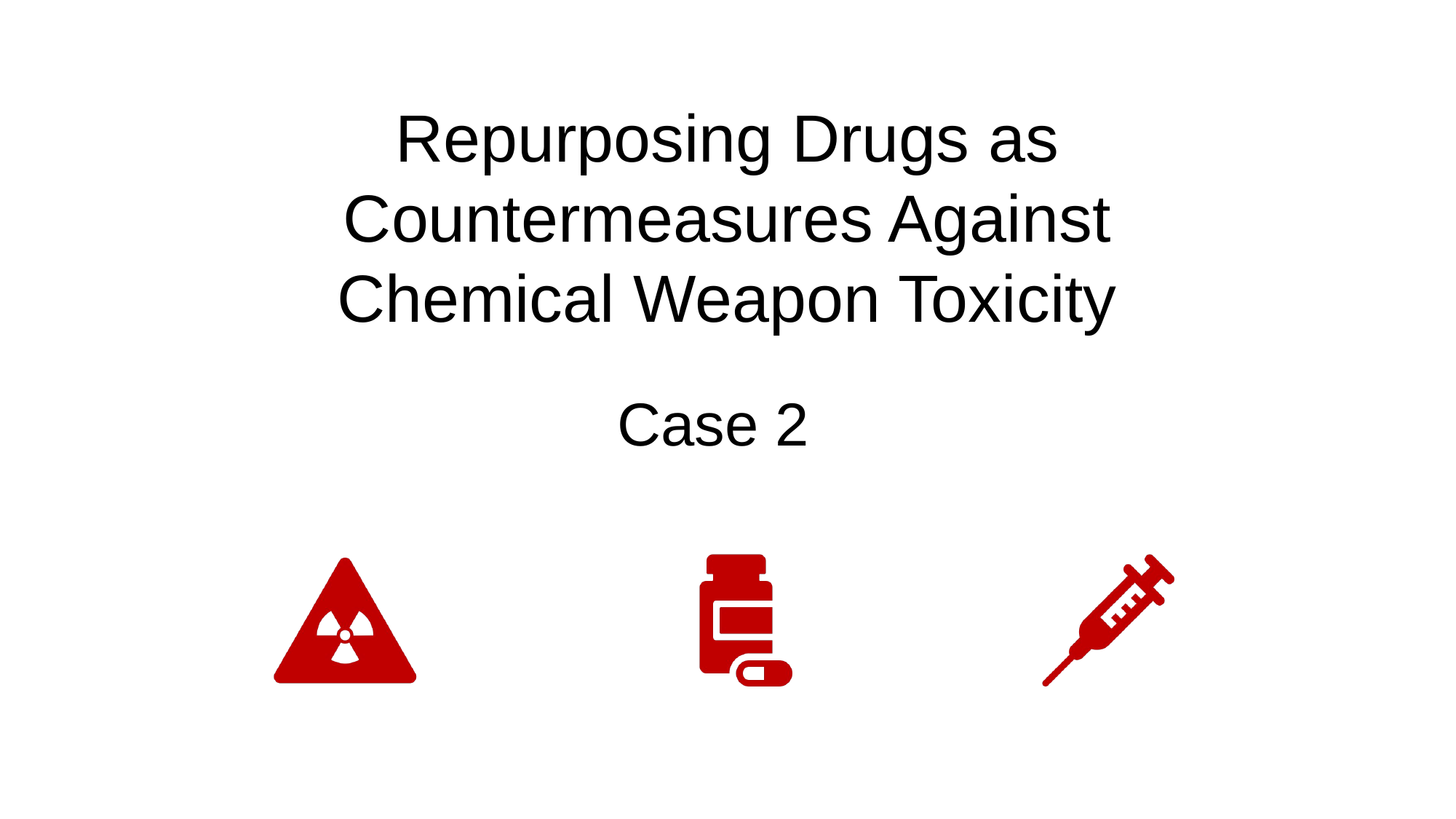

Repurposing Drugs as Countermeasures Against Chemical Weapon Toxicity
Case 2

## Slide 2
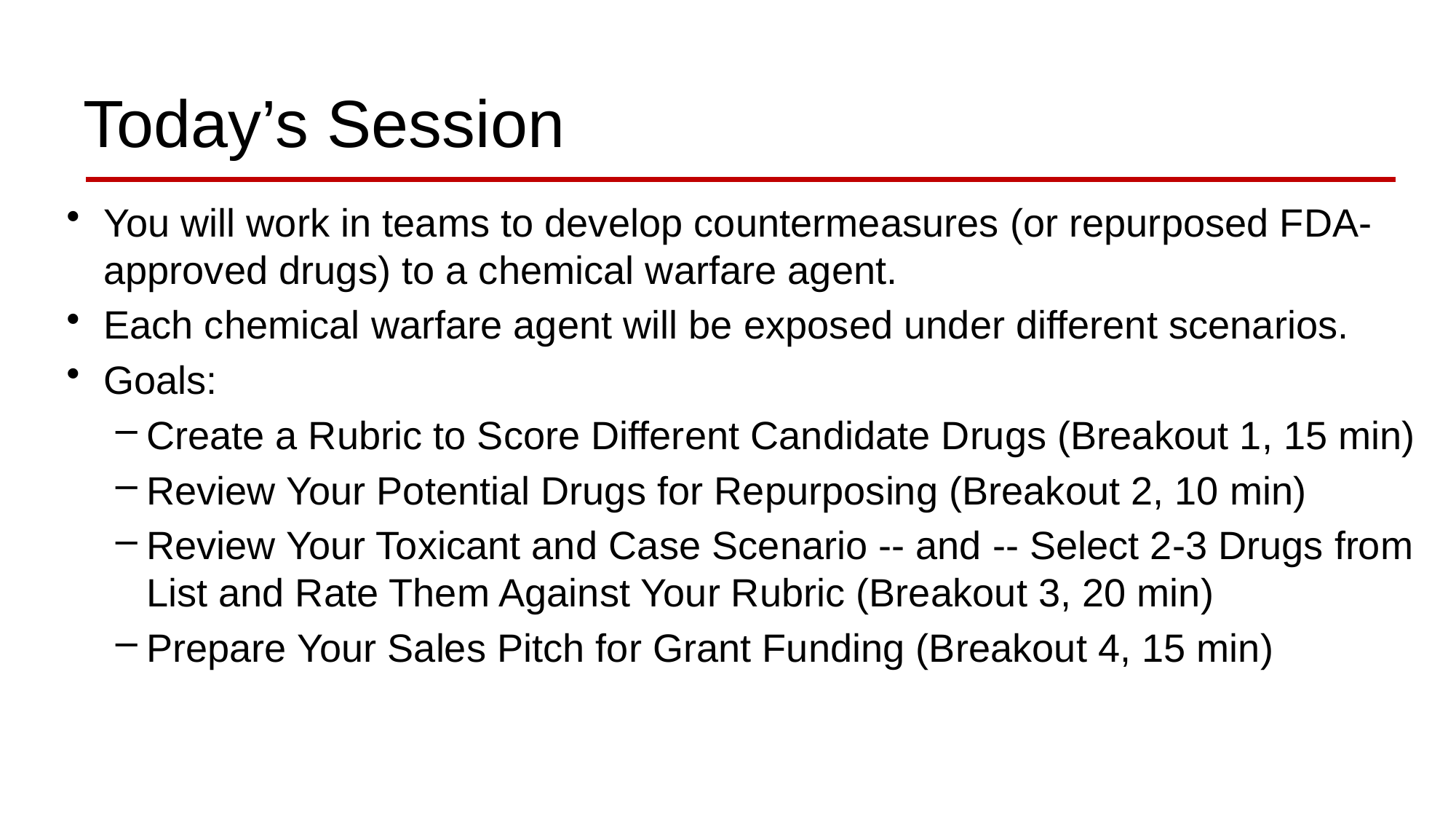

# Today’s Session
You will work in teams to develop countermeasures (or repurposed FDA-approved drugs) to a chemical warfare agent.
Each chemical warfare agent will be exposed under different scenarios.
Goals:
Create a Rubric to Score Different Candidate Drugs (Breakout 1, 15 min)
Review Your Potential Drugs for Repurposing (Breakout 2, 10 min)
Review Your Toxicant and Case Scenario -- and -- Select 2-3 Drugs from List and Rate Them Against Your Rubric (Breakout 3, 20 min)
Prepare Your Sales Pitch for Grant Funding (Breakout 4, 15 min)

## Slide 3
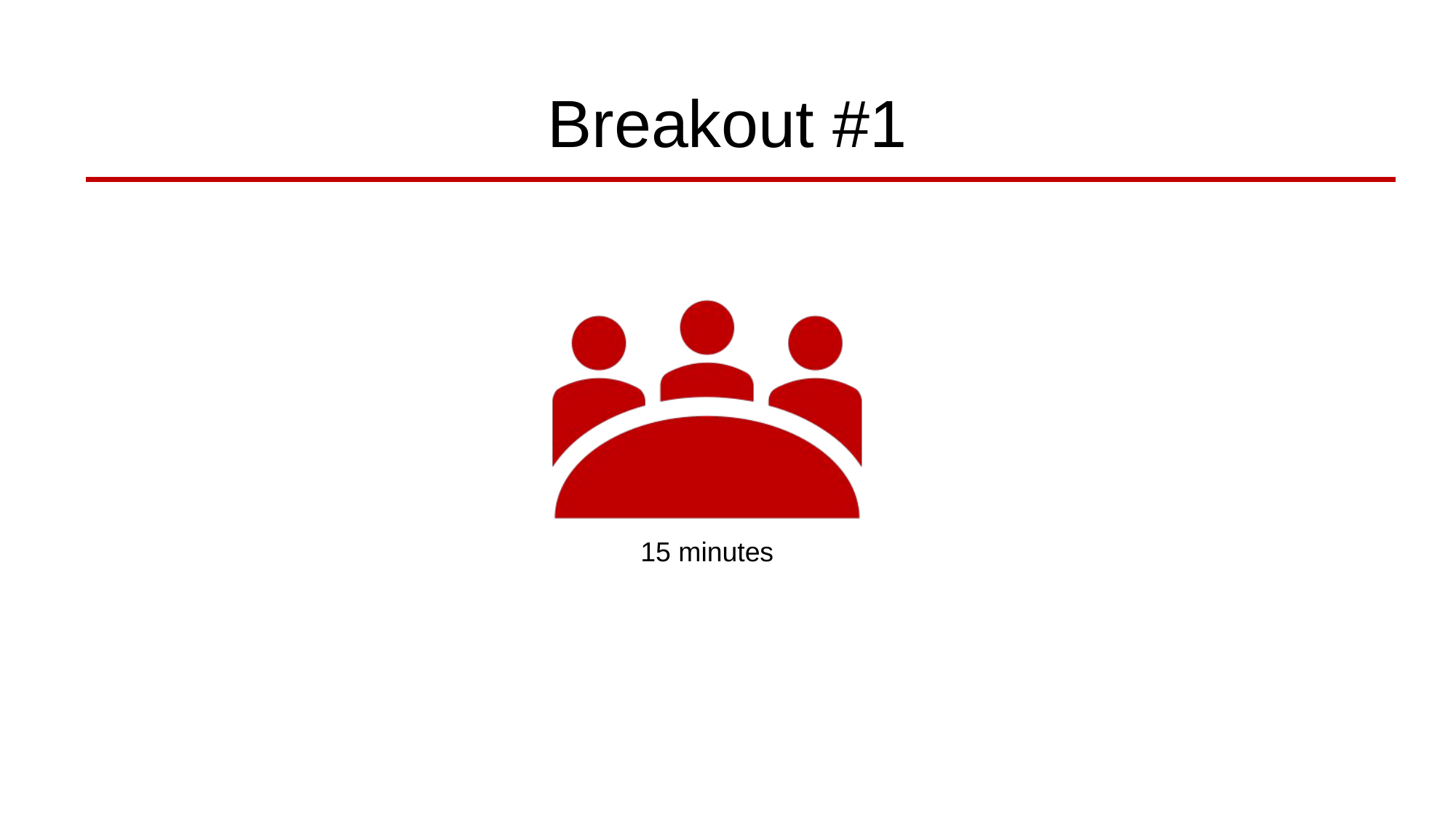

# Breakout #1
15 minutes

## Slide 4
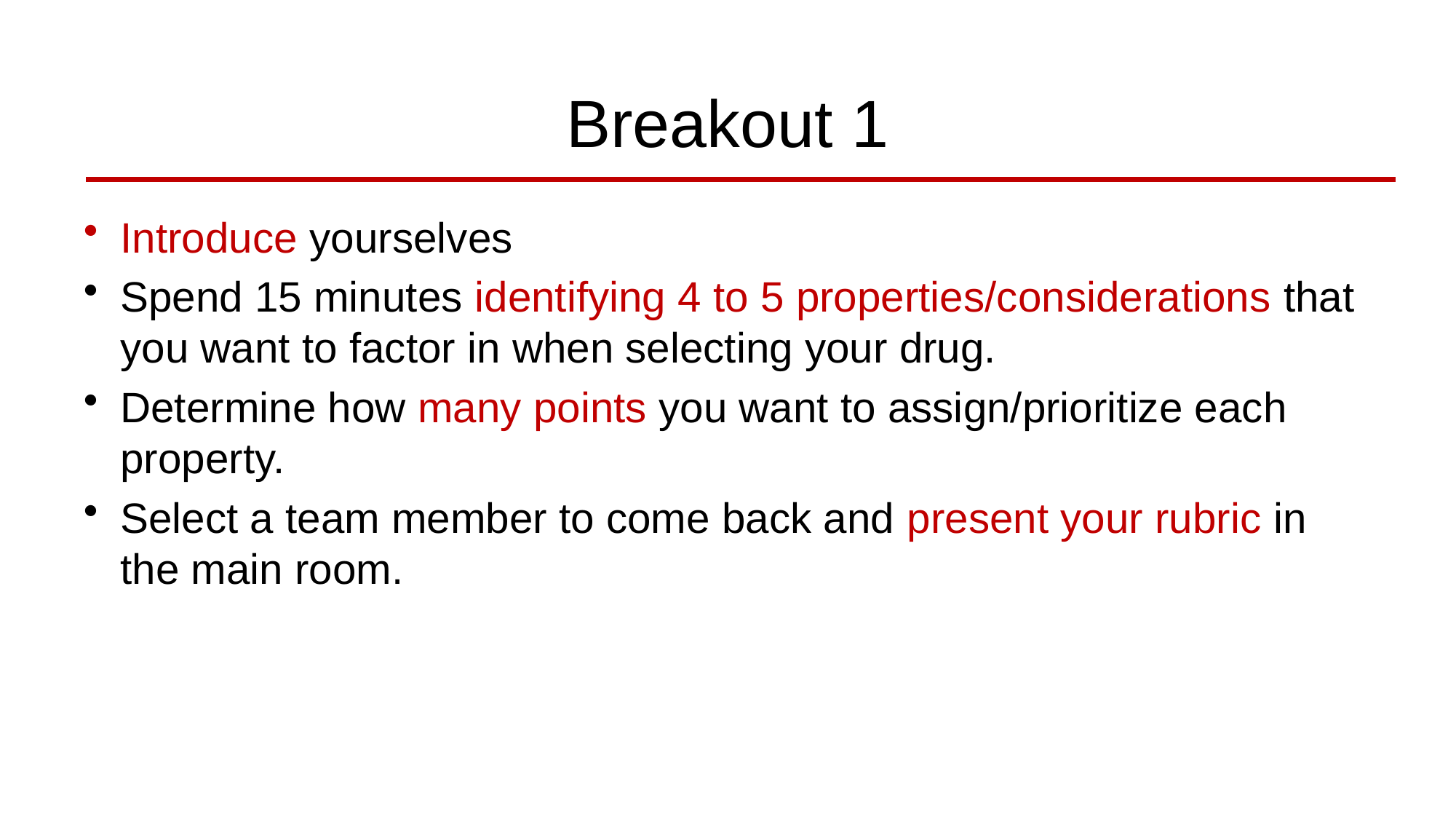

# Breakout 1
Introduce yourselves
Spend 15 minutes identifying 4 to 5 properties/considerations that you want to factor in when selecting your drug.
Determine how many points you want to assign/prioritize each property.
Select a team member to come back and present your rubric in the main room.

## Slide 5
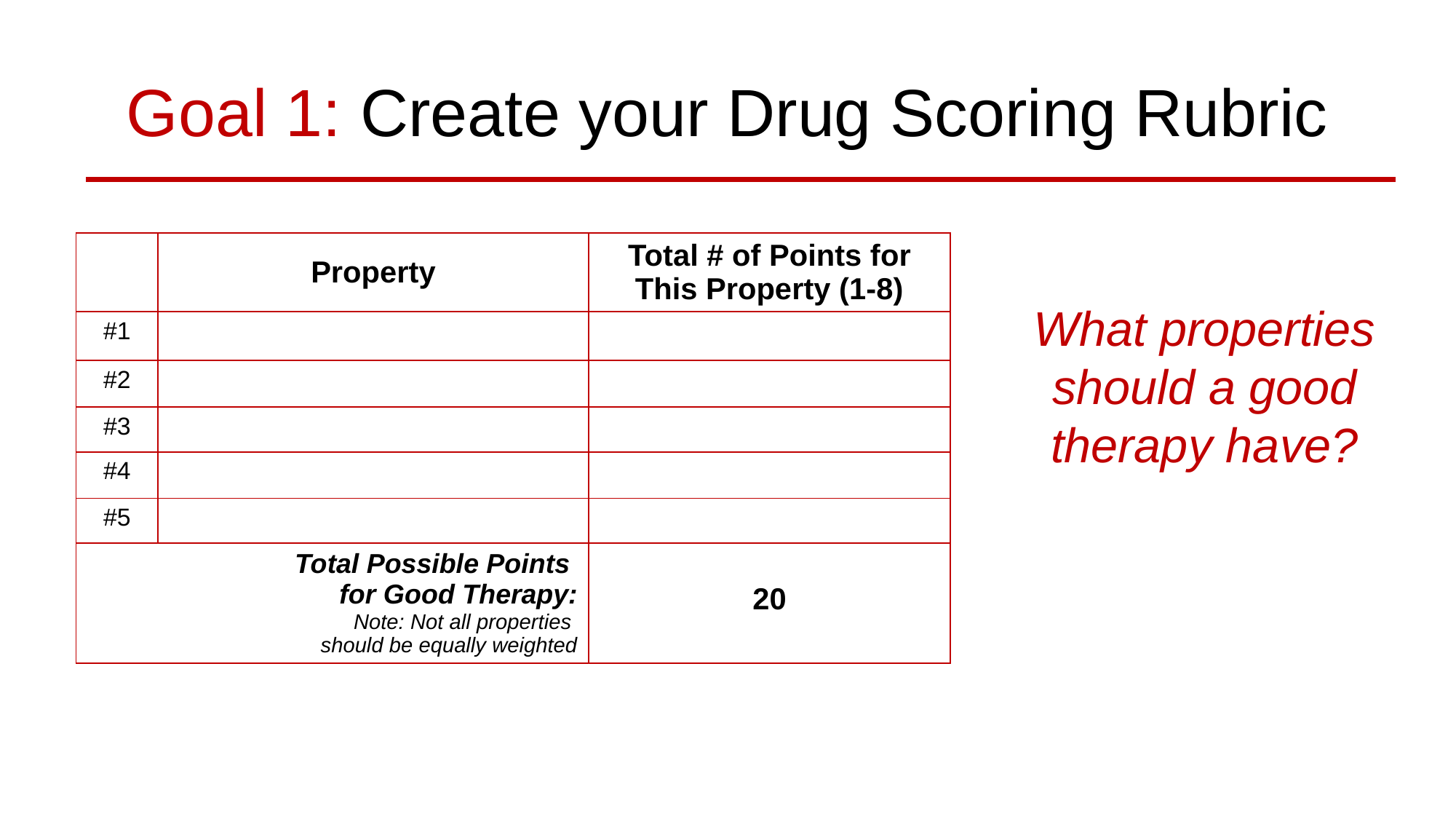

# Goal 1: Create your Drug Scoring Rubric
| | Property | Total # of Points for This Property (1-8) |
| --- | --- | --- |
| #1 | | |
| #2 | | |
| #3 | | |
| #4 | | |
| #5 | | |
| Total Possible Points for Good Therapy: Note: Not all properties should be equally weighted | | 20 |
What properties should a good therapy have?

## Slide 6
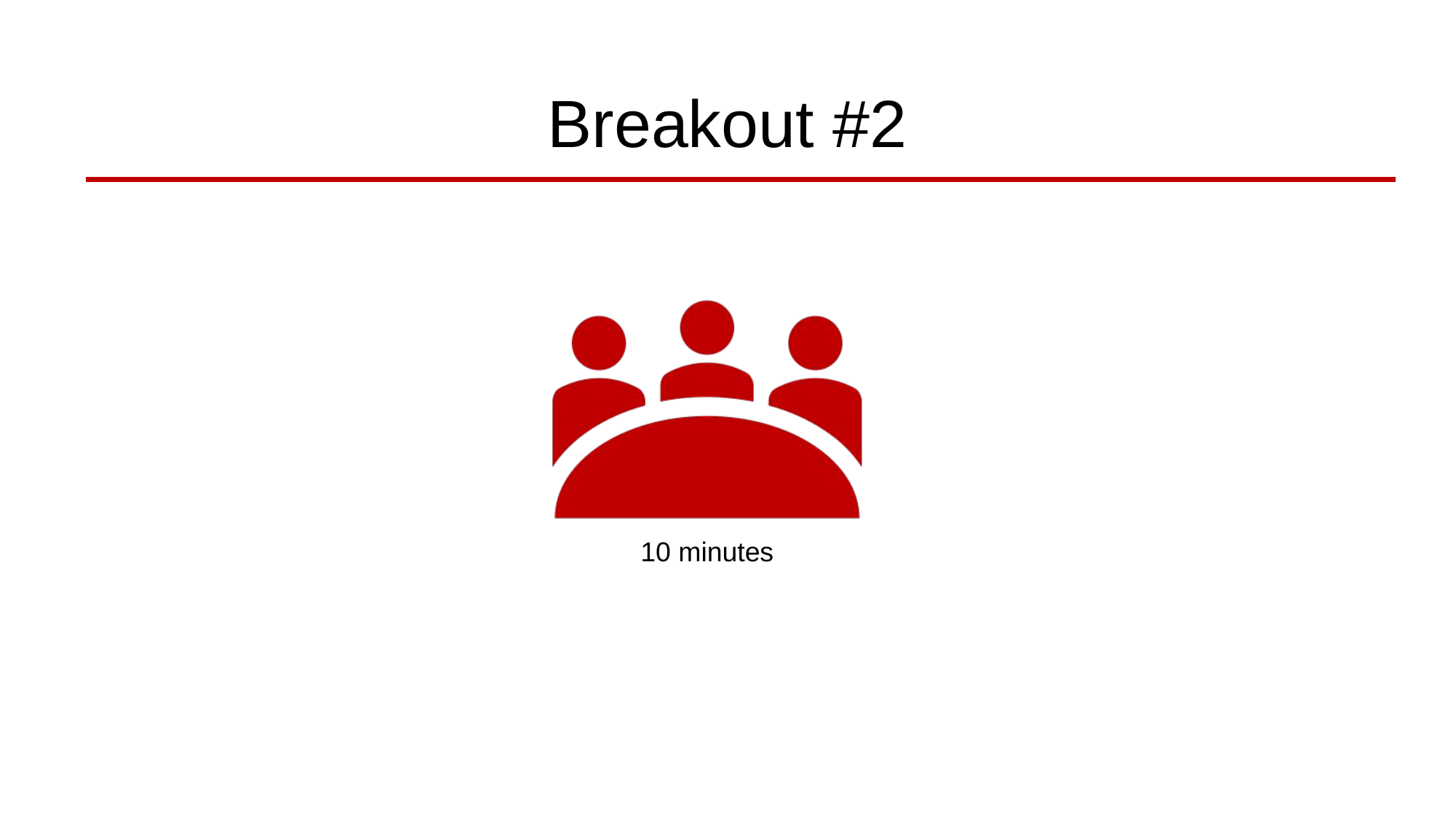

# Breakout #2
10 minutes

## Slide 7
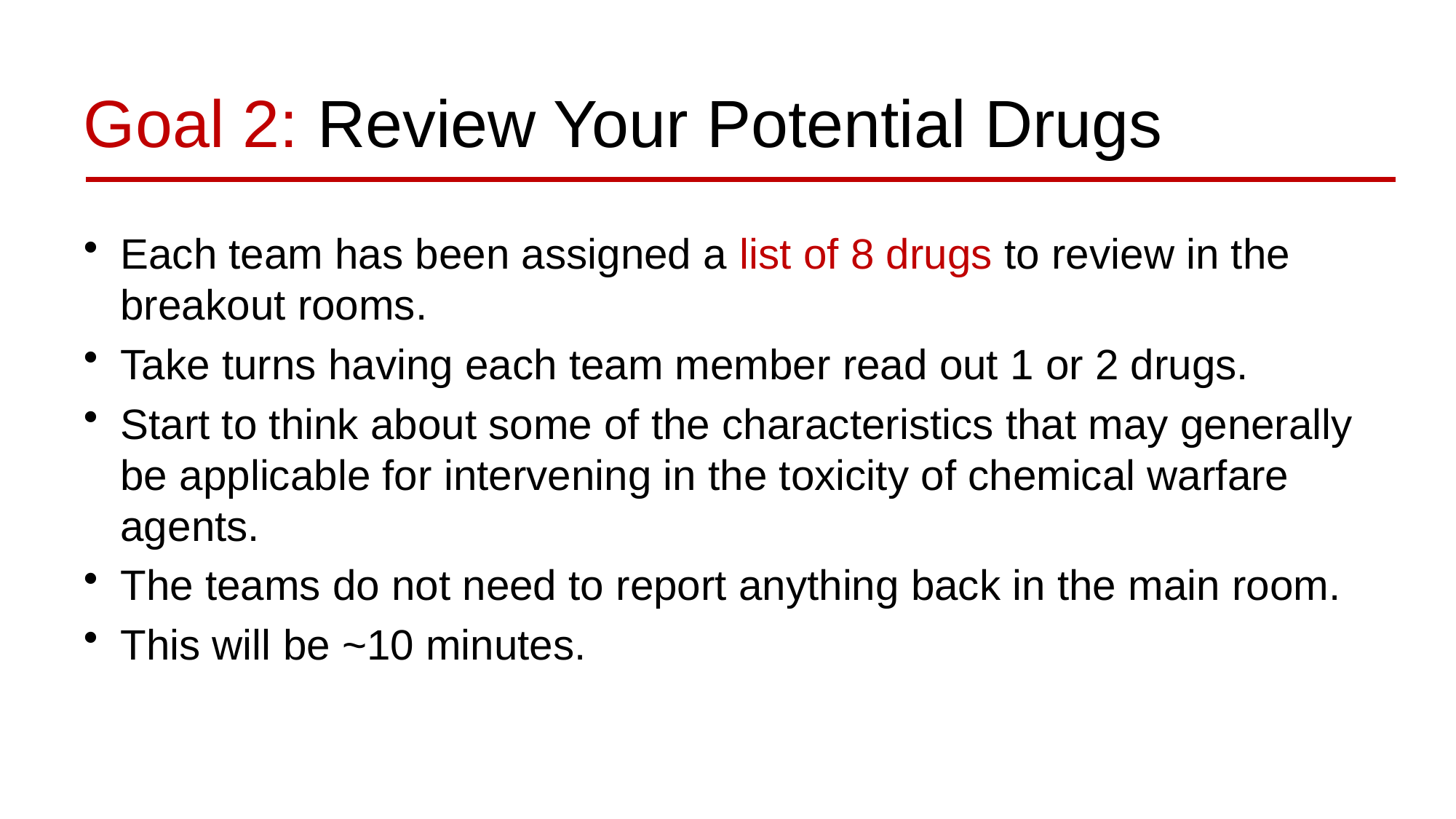

# Goal 2: Review Your Potential Drugs
Each team has been assigned a list of 8 drugs to review in the breakout rooms.
Take turns having each team member read out 1 or 2 drugs.
Start to think about some of the characteristics that may generally be applicable for intervening in the toxicity of chemical warfare agents.
The teams do not need to report anything back in the main room.
This will be ~10 minutes.

## Slide 8
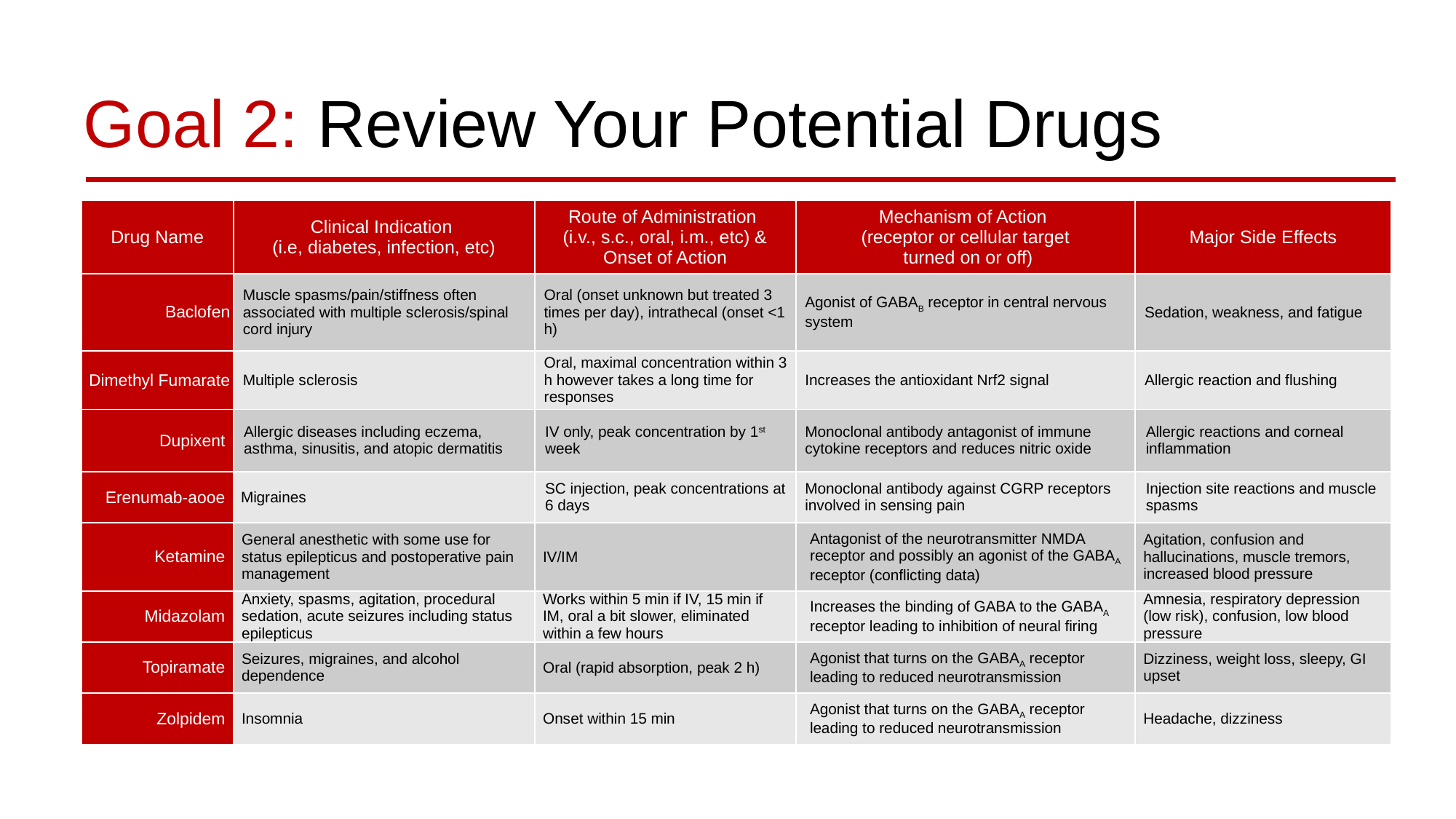

# Goal 2: Review Your Potential Drugs
| Drug Name | Clinical Indication (i.e, diabetes, infection, etc) | Route of Administration (i.v., s.c., oral, i.m., etc) & Onset of Action | Mechanism of Action (receptor or cellular target turned on or off) | Major Side Effects |
| --- | --- | --- | --- | --- |
| Baclofen | Muscle spasms/pain/stiffness often associated with multiple sclerosis/spinal cord injury | Oral (onset unknown but treated 3 times per day), intrathecal (onset <1 h) | Agonist of GABAB receptor in central nervous system | Sedation, weakness, and fatigue |
| Dimethyl Fumarate | Multiple sclerosis | Oral, maximal concentration within 3 h however takes a long time for responses | Increases the antioxidant Nrf2 signal | Allergic reaction and flushing |
| Dupixent | Allergic diseases including eczema, asthma, sinusitis, and atopic dermatitis | IV only, peak concentration by 1st week | Monoclonal antibody antagonist of immune cytokine receptors and reduces nitric oxide | Allergic reactions and corneal inflammation |
| Erenumab-aooe | Migraines | SC injection, peak concentrations at 6 days | Monoclonal antibody against CGRP receptors involved in sensing pain | Injection site reactions and muscle spasms |
| Ketamine | General anesthetic with some use for status epilepticus and postoperative pain management | IV/IM | Antagonist of the neurotransmitter NMDA receptor and possibly an agonist of the GABAA receptor (conflicting data) | Agitation, confusion and hallucinations, muscle tremors, increased blood pressure |
| Midazolam | Anxiety, spasms, agitation, procedural sedation, acute seizures including status epilepticus | Works within 5 min if IV, 15 min if IM, oral a bit slower, eliminated within a few hours | Increases the binding of GABA to the GABAA receptor leading to inhibition of neural firing | Amnesia, respiratory depression (low risk), confusion, low blood pressure |
| Topiramate | Seizures, migraines, and alcohol dependence | Oral (rapid absorption, peak 2 h) | Agonist that turns on the GABAA receptor leading to reduced neurotransmission | Dizziness, weight loss, sleepy, GI upset |
| Zolpidem | Insomnia | Onset within 15 min | Agonist that turns on the GABAA receptor leading to reduced neurotransmission | Headache, dizziness |

## Slide 9
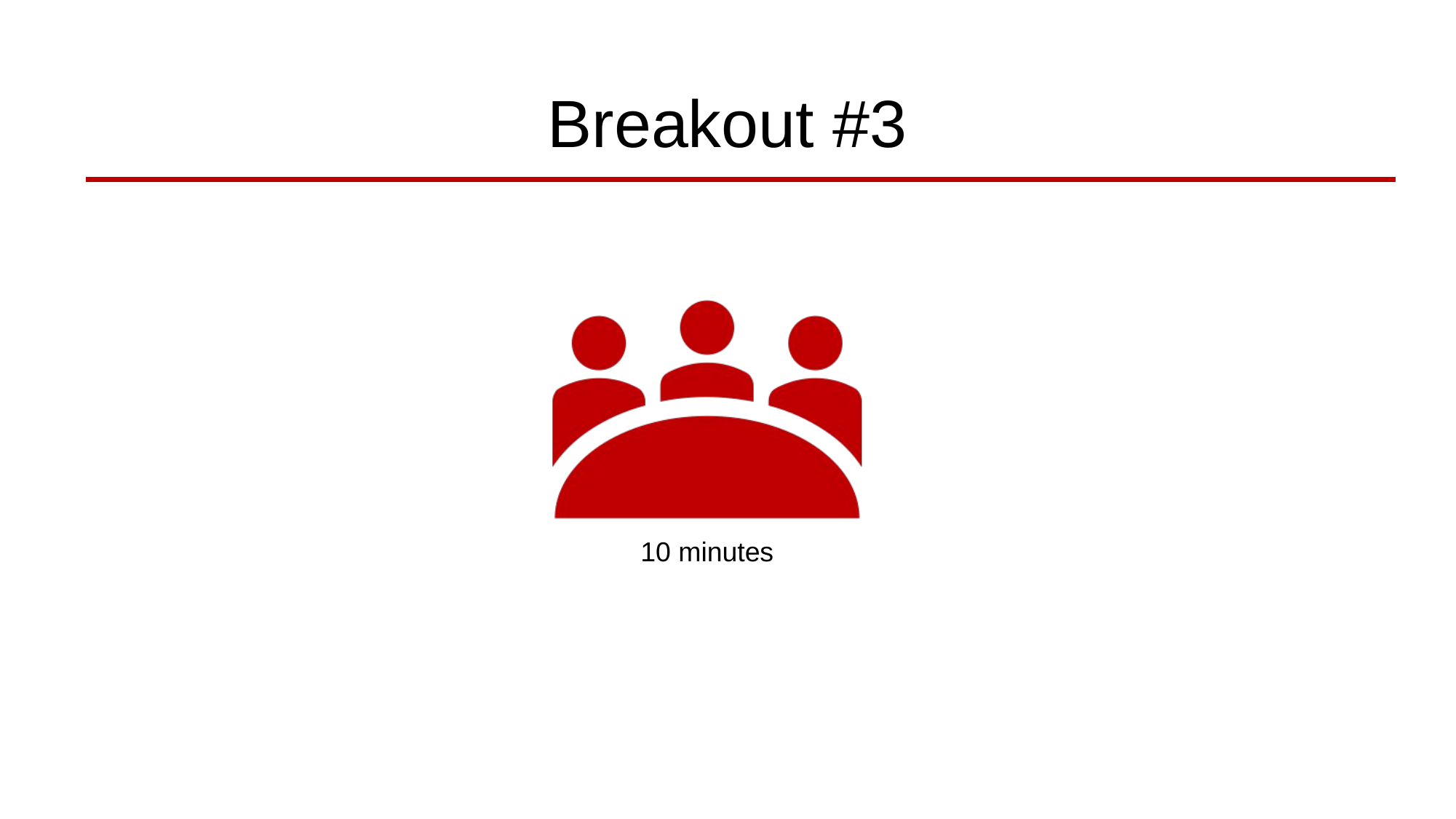

# Breakout #3
10 minutes

## Slide 10
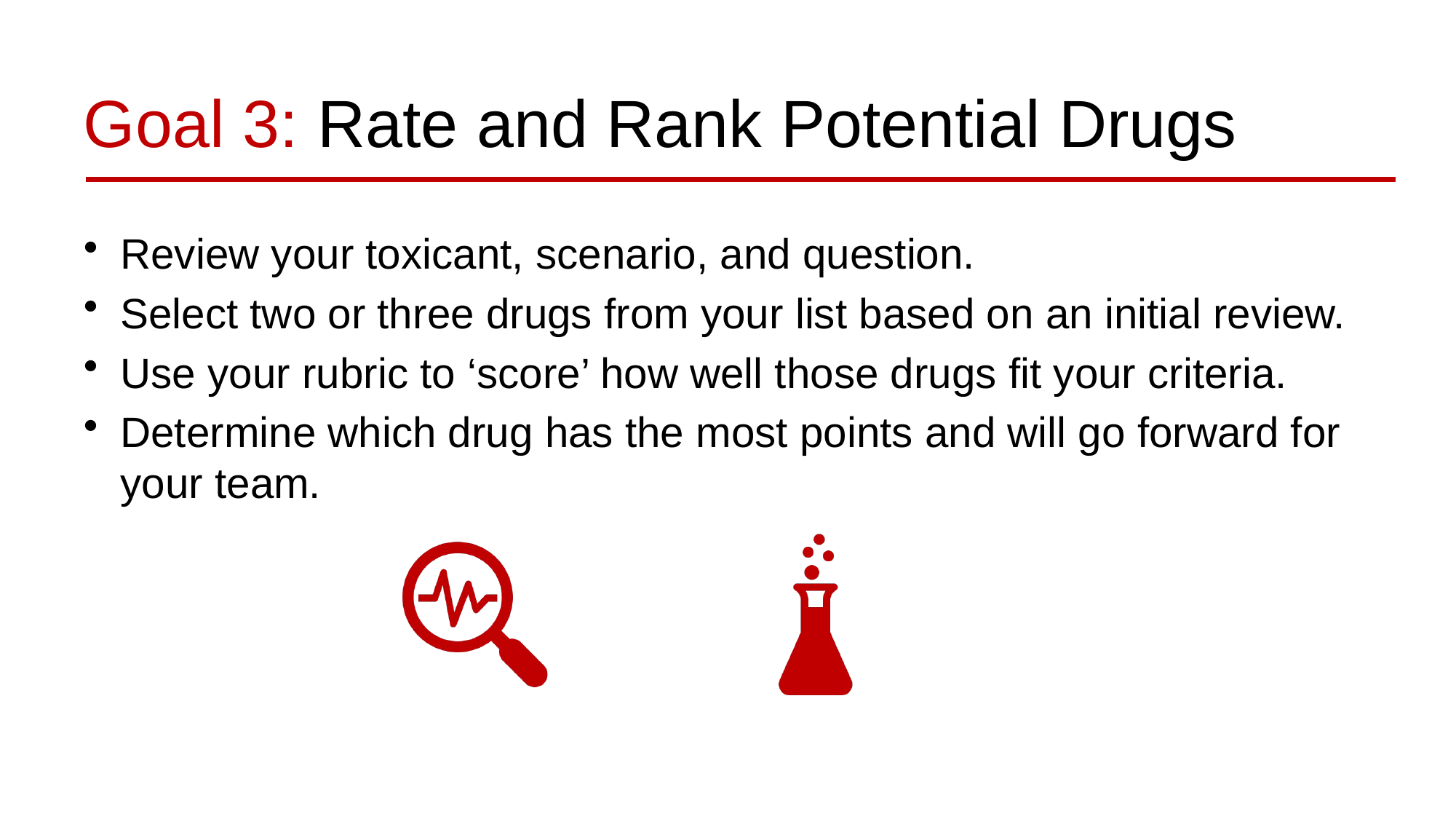

# Goal 3: Rate and Rank Potential Drugs
Review your toxicant, scenario, and question.
Select two or three drugs from your list based on an initial review.
Use your rubric to ‘score’ how well those drugs fit your criteria.
Determine which drug has the most points and will go forward for your team.

## Slide 11
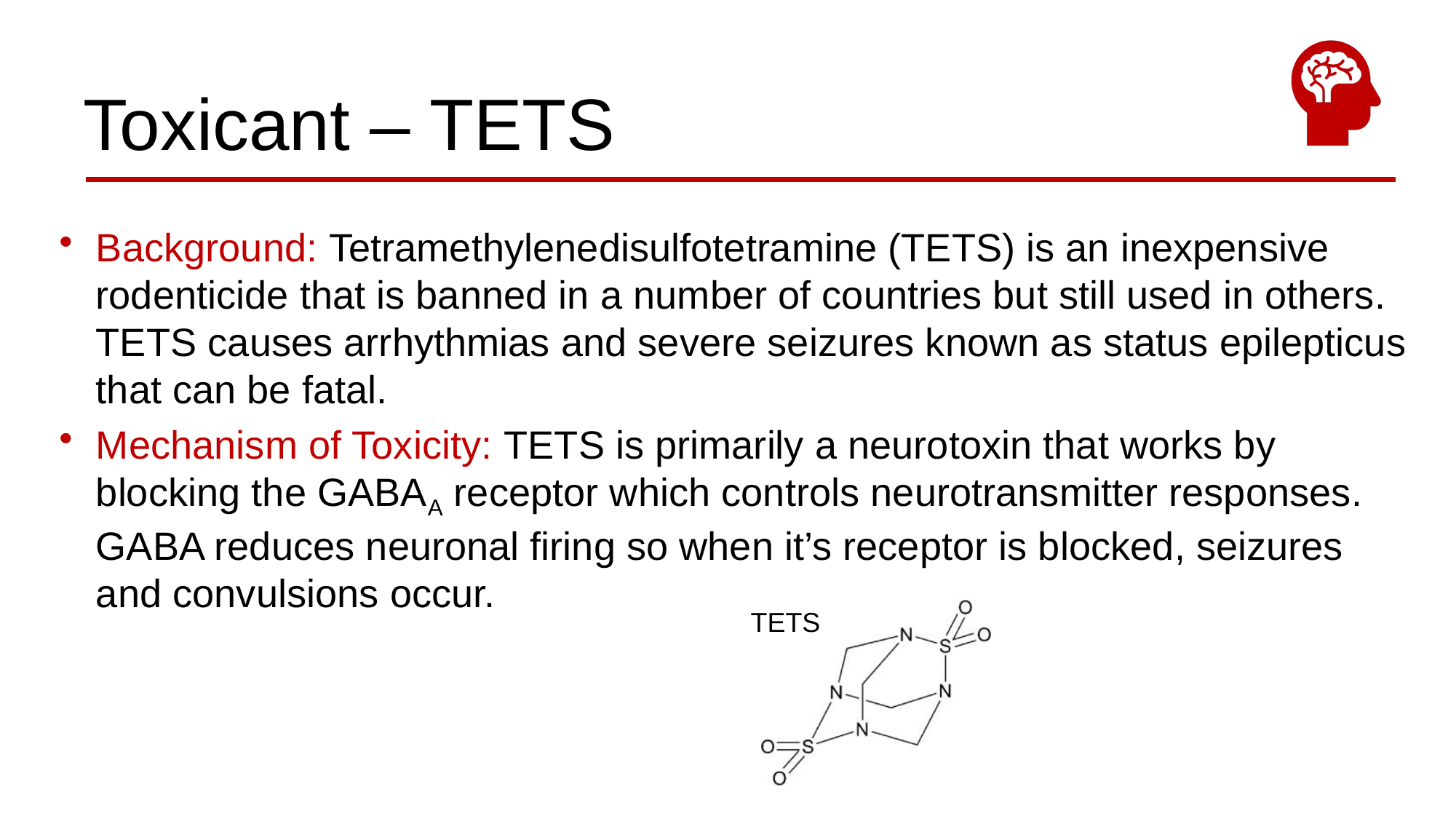

# Toxicant – TETS
Background: Tetramethylenedisulfotetramine (TETS) is an inexpensive rodenticide that is banned in a number of countries but still used in others. TETS causes arrhythmias and severe seizures known as status epilepticus that can be fatal.
Mechanism of Toxicity: TETS is primarily a neurotoxin that works by blocking the GABAA receptor which controls neurotransmitter responses. GABA reduces neuronal firing so when it’s receptor is blocked, seizures and convulsions occur.
TETS

## Slide 12
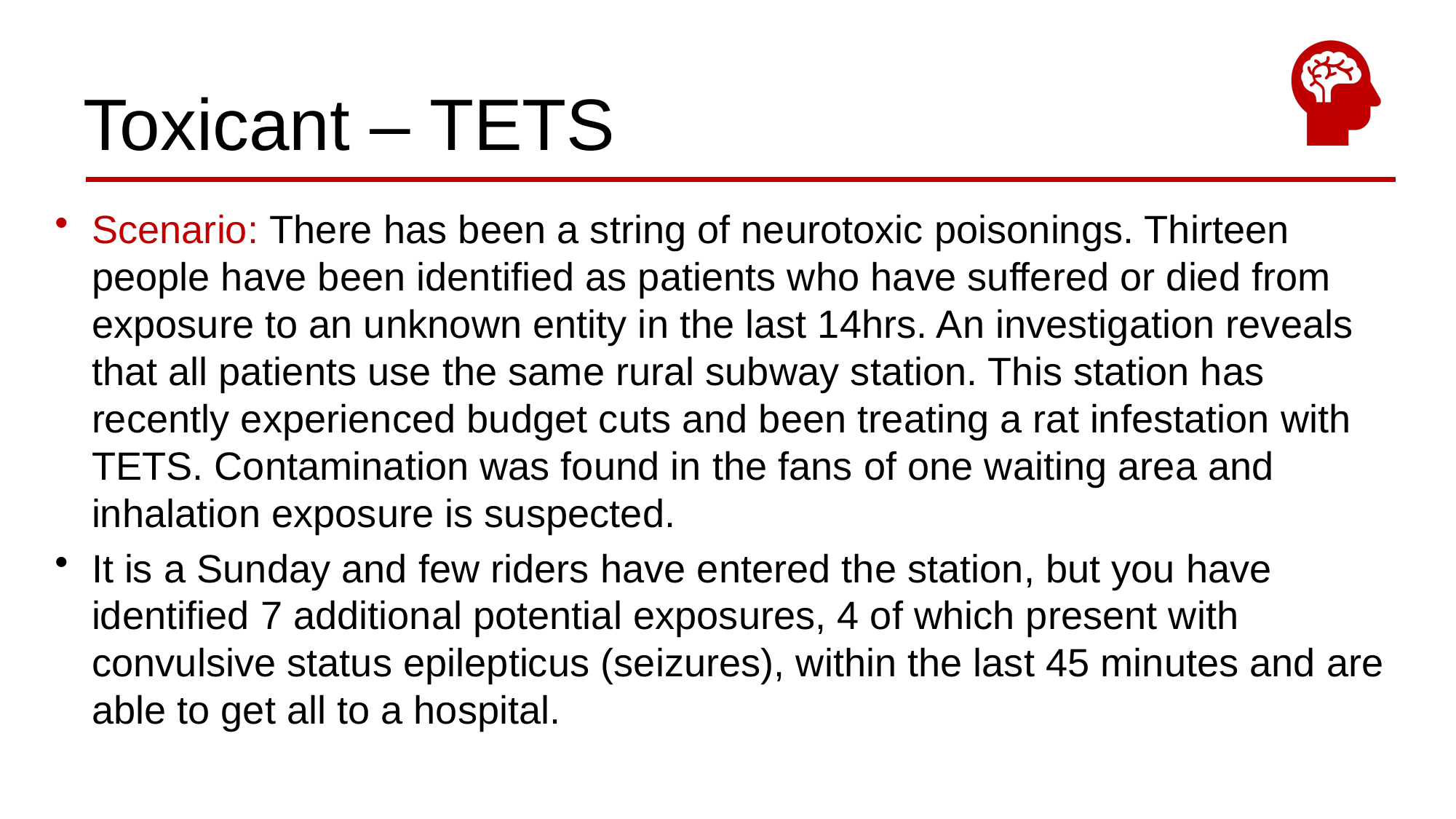

# Toxicant – TETS
Scenario: There has been a string of neurotoxic poisonings. Thirteen people have been identified as patients who have suffered or died from exposure to an unknown entity in the last 14hrs. An investigation reveals that all patients use the same rural subway station. This station has recently experienced budget cuts and been treating a rat infestation with TETS. Contamination was found in the fans of one waiting area and inhalation exposure is suspected.
It is a Sunday and few riders have entered the station, but you have identified 7 additional potential exposures, 4 of which present with convulsive status epilepticus (seizures), within the last 45 minutes and are able to get all to a hospital.

## Slide 13
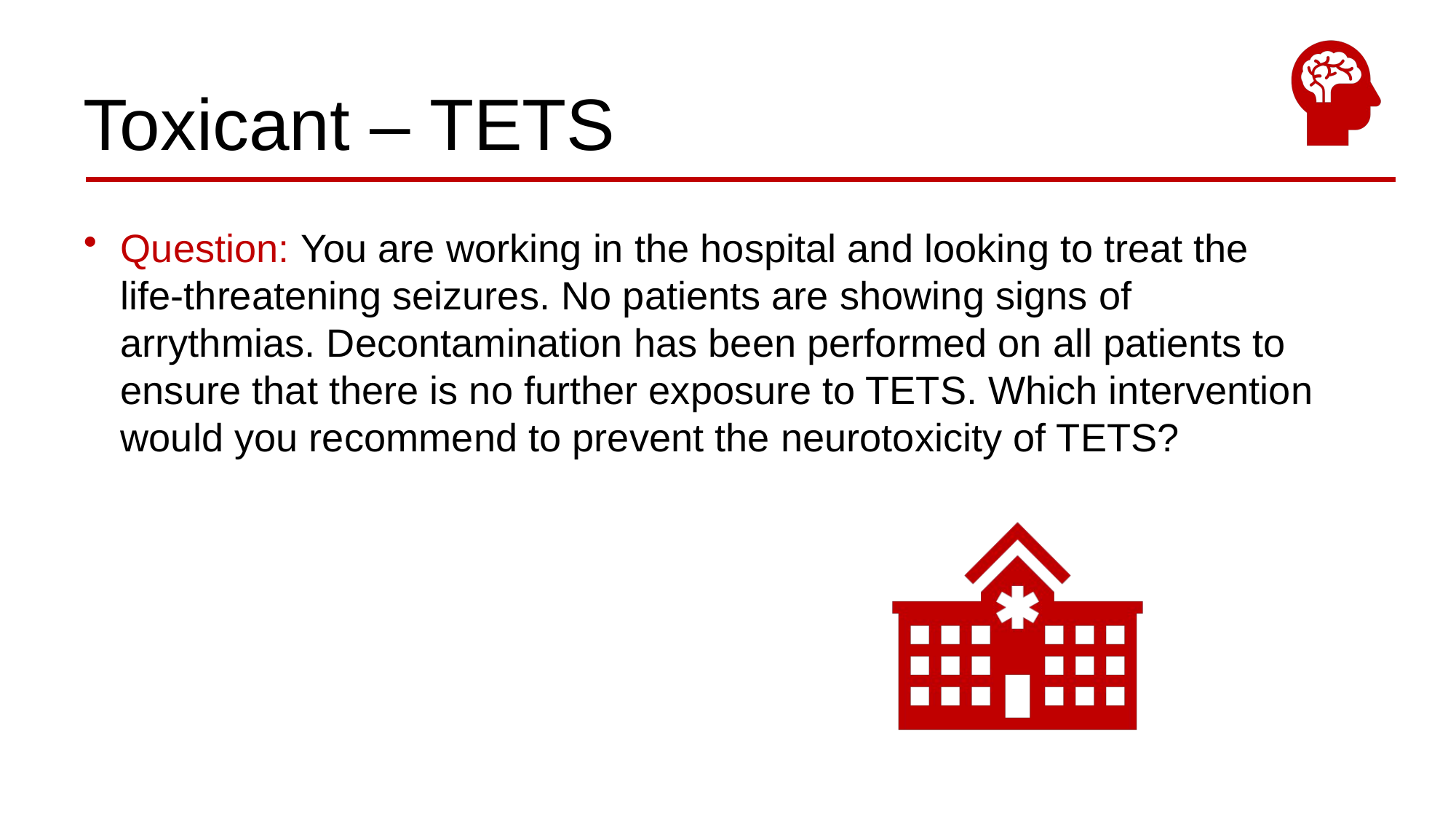

# Toxicant – TETS
Question: You are working in the hospital and looking to treat the life-threatening seizures. No patients are showing signs of arrythmias. Decontamination has been performed on all patients to ensure that there is no further exposure to TETS. Which intervention would you recommend to prevent the neurotoxicity of TETS?

## Slide 14
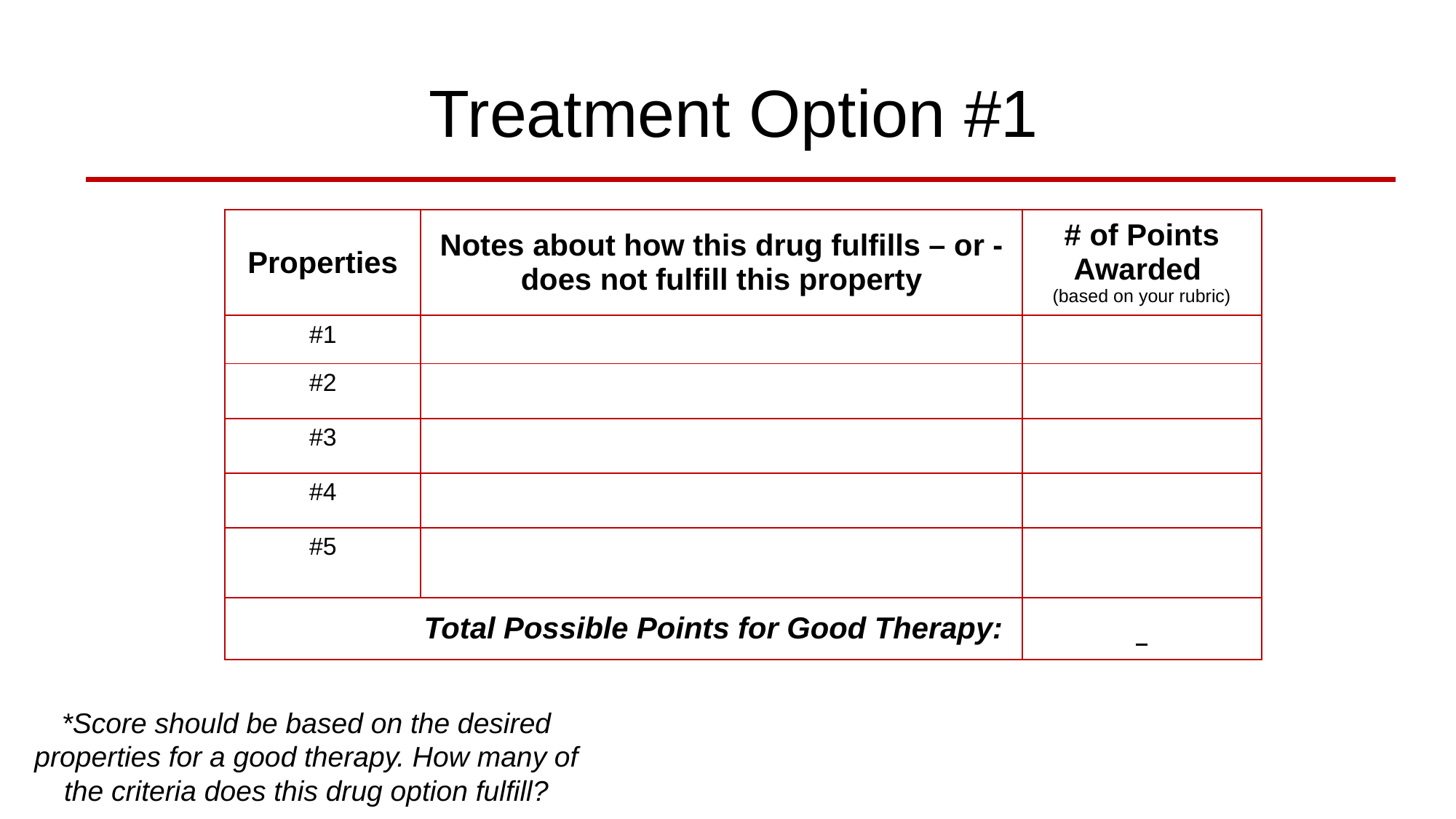

# Treatment Option #1
| Properties | Notes about how this drug fulfills – or -does not fulfill this property | # of Points Awarded (based on your rubric) |
| --- | --- | --- |
| #1 | | |
| #2 | | |
| #3 | | |
| #4 | | |
| #5 | | |
| Total Possible Points for Good Therapy: | | |
*Score should be based on the desired properties for a good therapy. How many of the criteria does this drug option fulfill?

## Slide 15
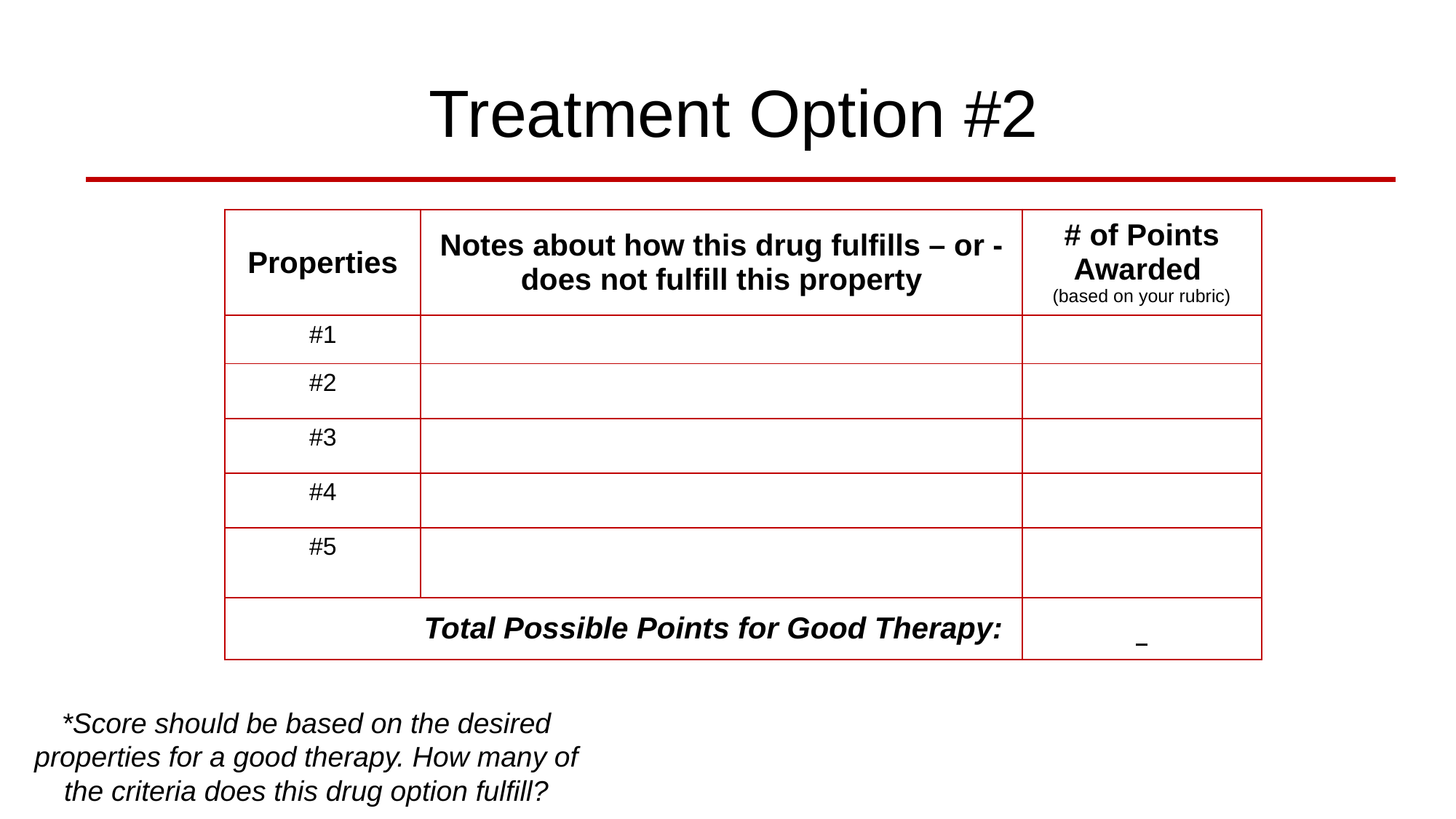

# Treatment Option #2
| Properties | Notes about how this drug fulfills – or -does not fulfill this property | # of Points Awarded (based on your rubric) |
| --- | --- | --- |
| #1 | | |
| #2 | | |
| #3 | | |
| #4 | | |
| #5 | | |
| Total Possible Points for Good Therapy: | | |
*Score should be based on the desired properties for a good therapy. How many of the criteria does this drug option fulfill?

## Slide 16
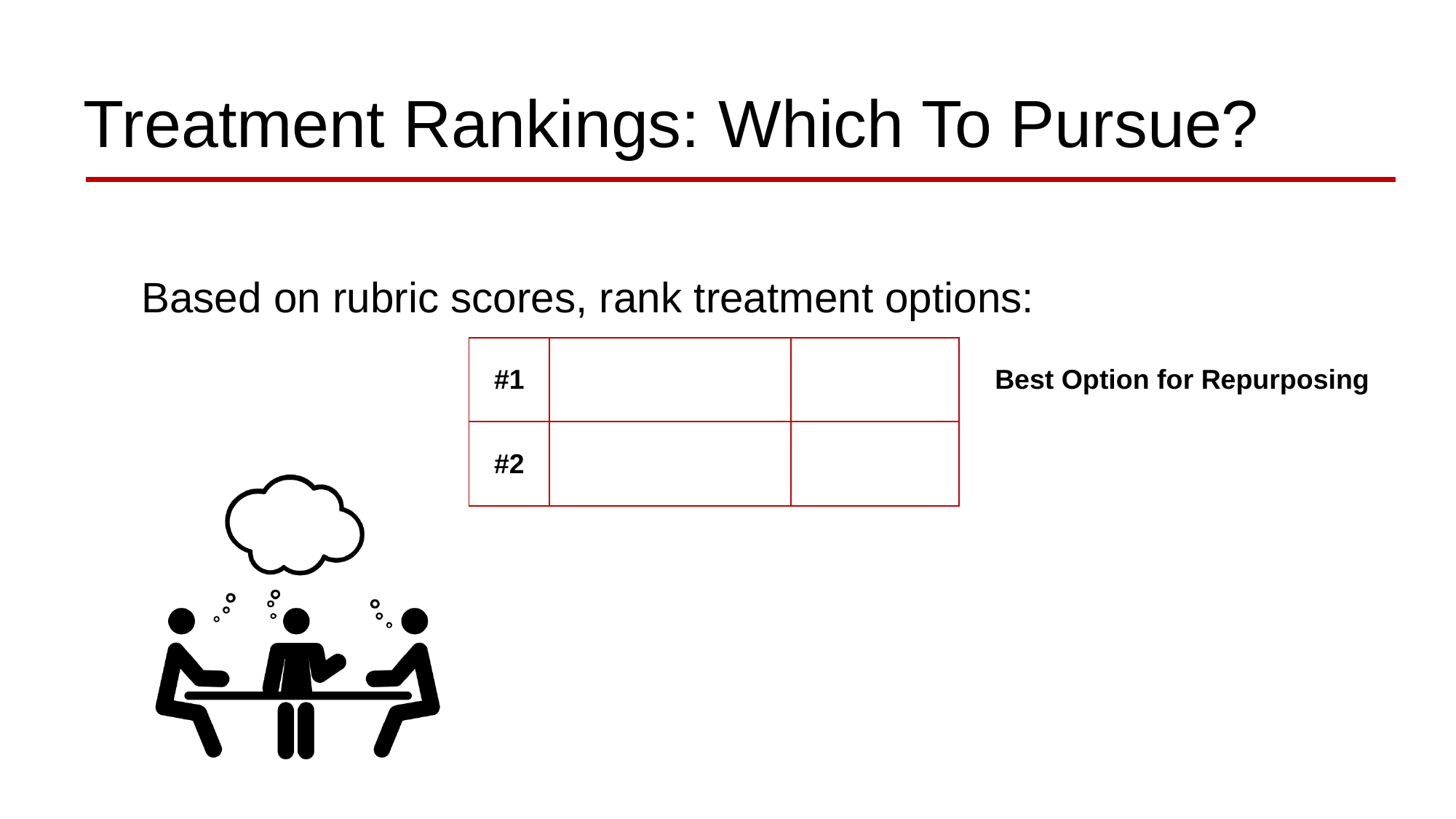

# Treatment Rankings: Which To Pursue?
Based on rubric scores, rank treatment options:
| #1 | | |
| --- | --- | --- |
| #2 | | |
Best Option for Repurposing

## Slide 17
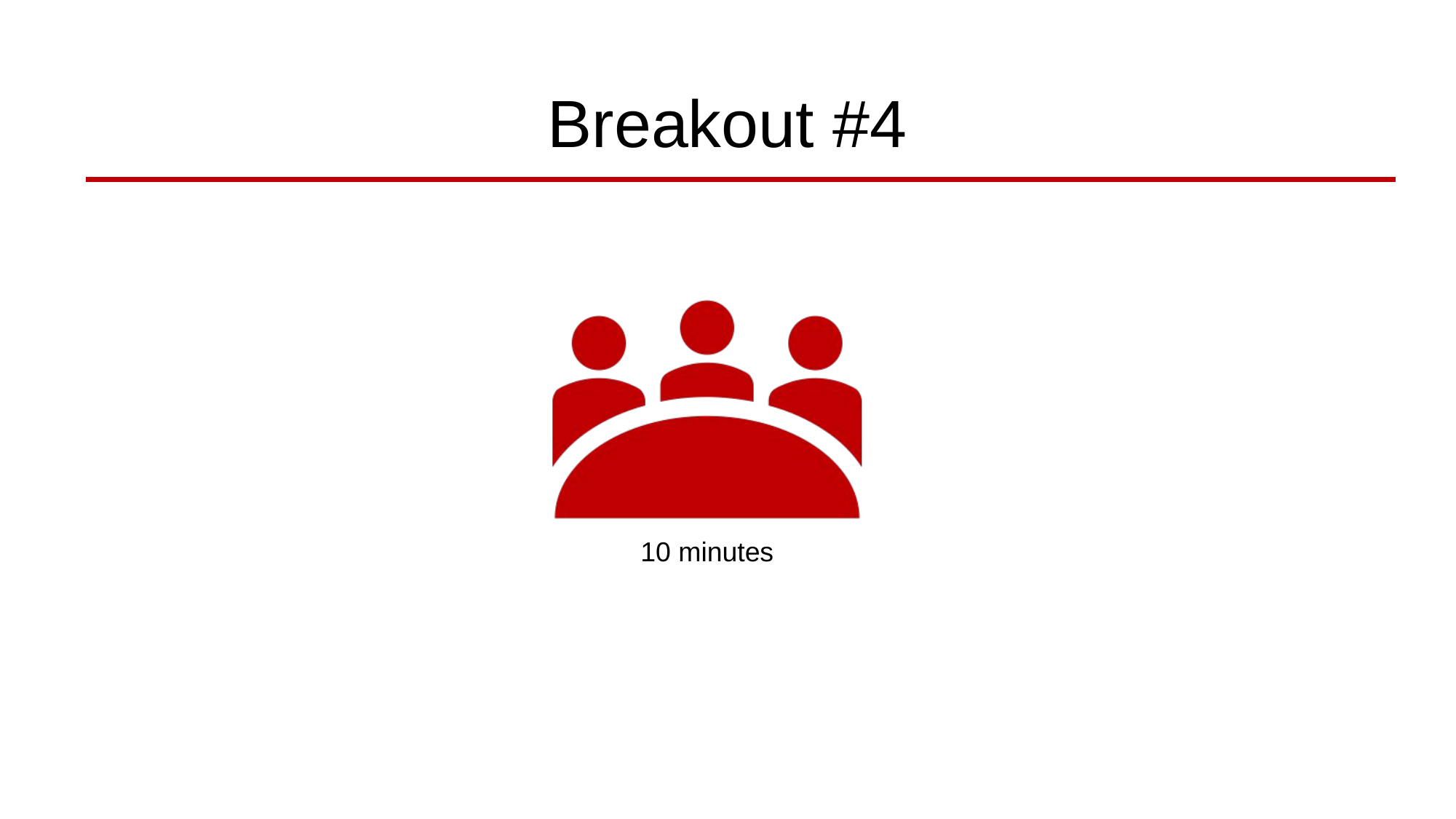

# Breakout #4
10 minutes

## Slide 18
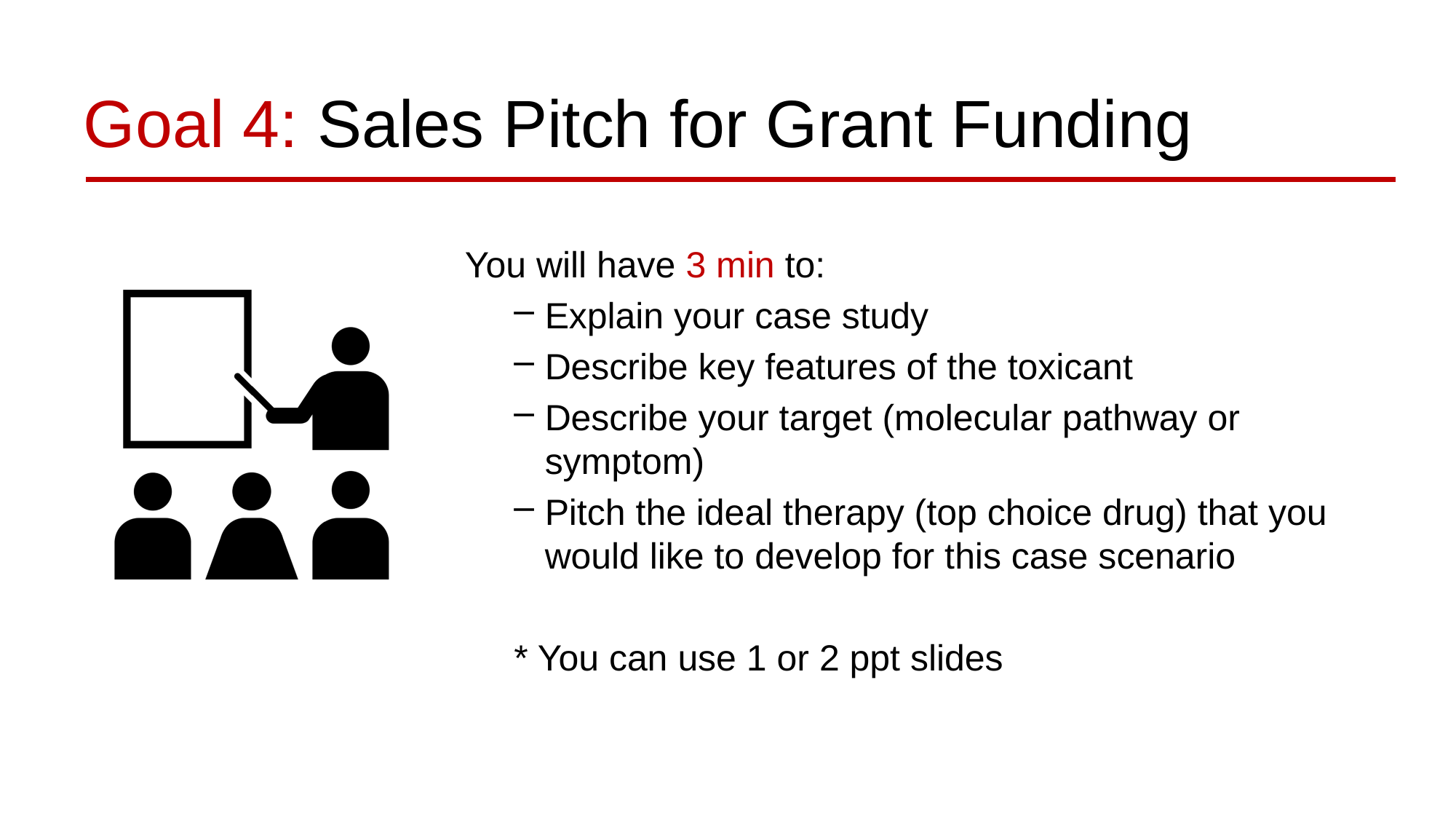

# Goal 4: Sales Pitch for Grant Funding
You will have 3 min to:
Explain your case study
Describe key features of the toxicant
Describe your target (molecular pathway or symptom)
Pitch the ideal therapy (top choice drug) that you would like to develop for this case scenario
* You can use 1 or 2 ppt slides
