# Supplementary material for: Repurposing FDA‐approved drugs to treat chemical weapon toxicities: Interactive case studies for trainees
Source: Pharmacol Res Perspect. 2024 Jul 4;12(4):e1229. doi: 10.1002/prp2.1229 (PMC11223991; doi:10.1002/prp2.1229)
Supplement: Supplementary file 3 — File S3. [file PRP2-12-e1229-s004.zip › Supporting File S5 - Case 3.pptx]

## Slide 1
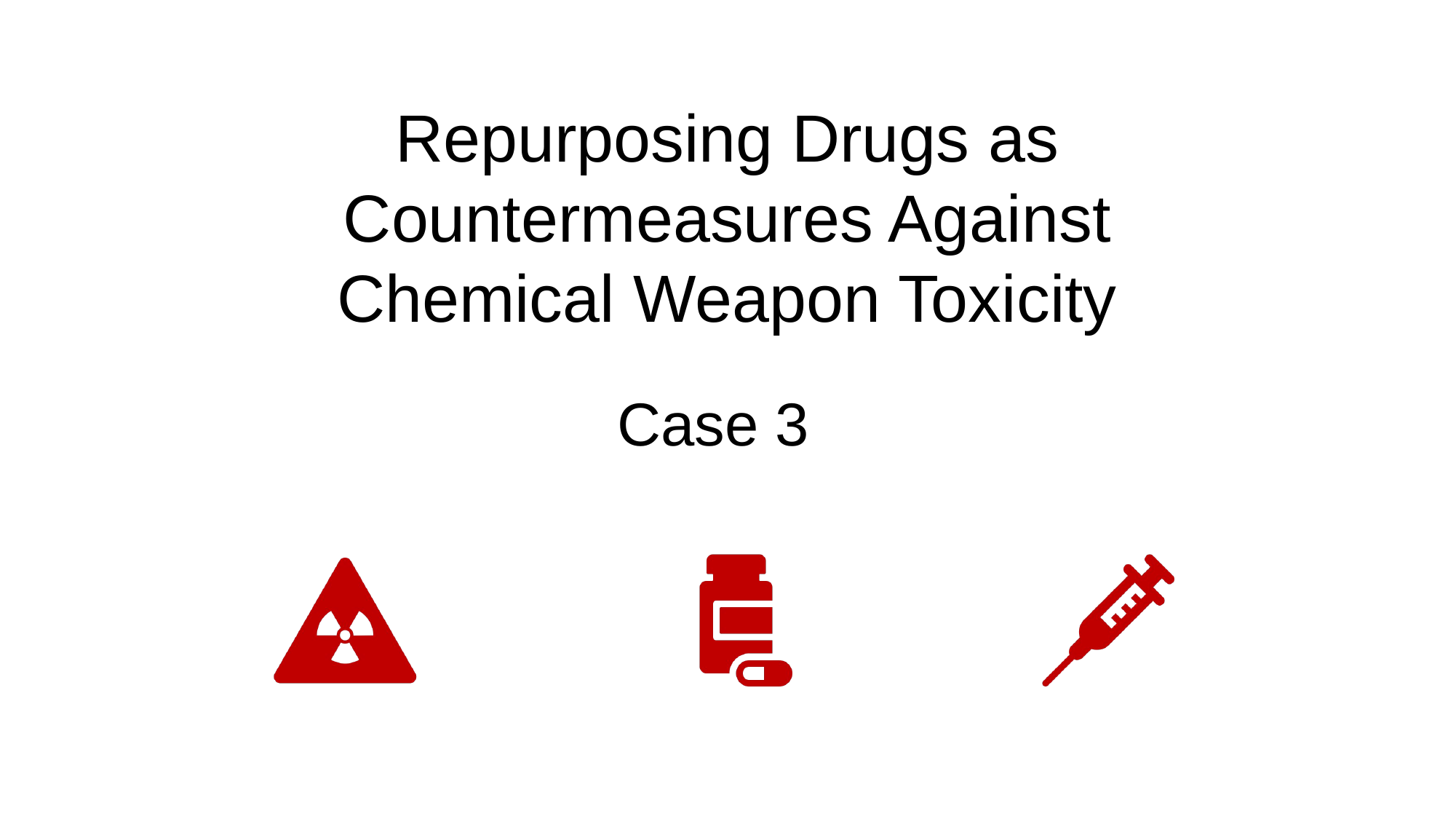

Repurposing Drugs as Countermeasures Against Chemical Weapon Toxicity
Case 3

## Slide 2
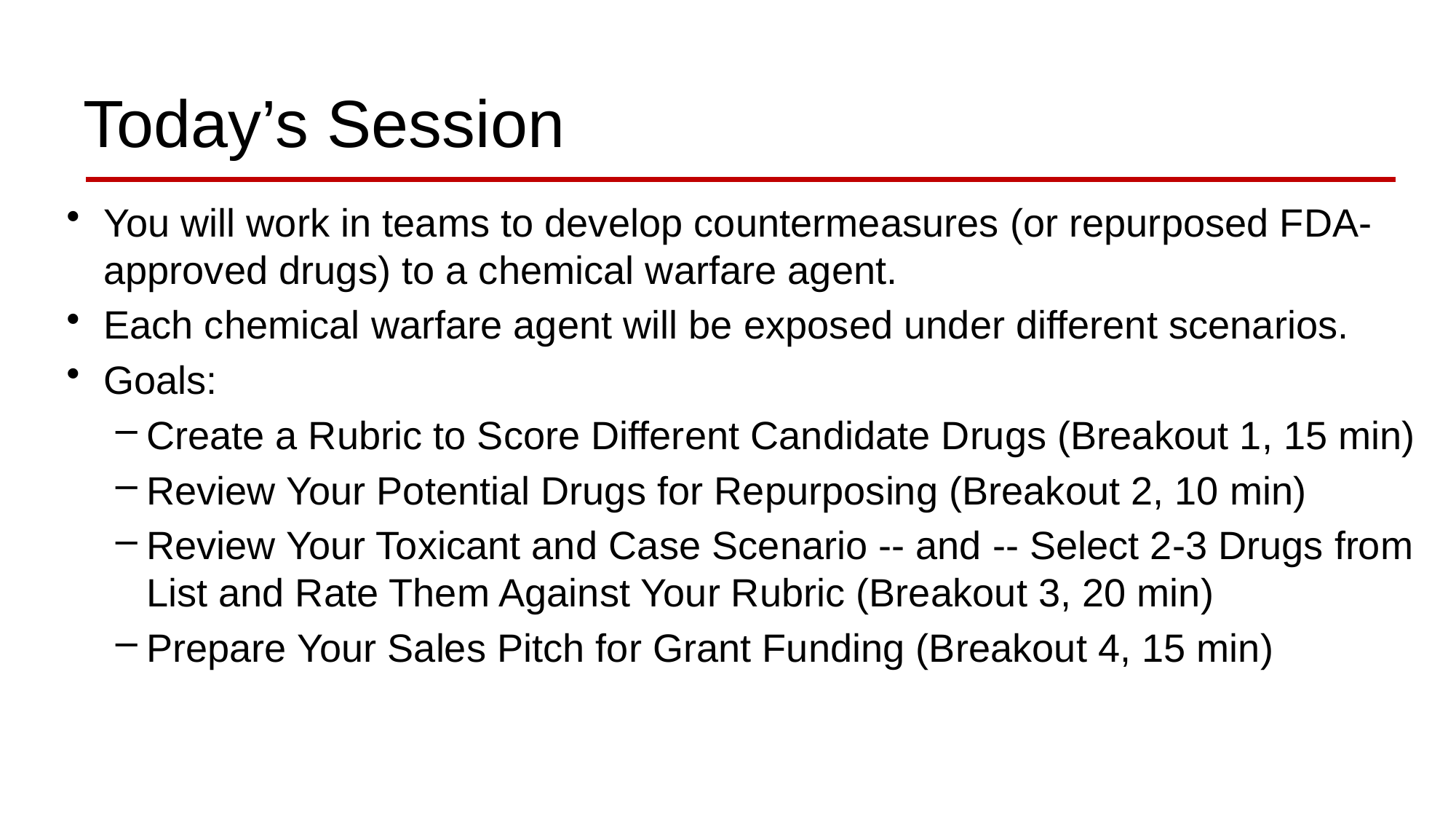

# Today’s Session
You will work in teams to develop countermeasures (or repurposed FDA-approved drugs) to a chemical warfare agent.
Each chemical warfare agent will be exposed under different scenarios.
Goals:
Create a Rubric to Score Different Candidate Drugs (Breakout 1, 15 min)
Review Your Potential Drugs for Repurposing (Breakout 2, 10 min)
Review Your Toxicant and Case Scenario -- and -- Select 2-3 Drugs from List and Rate Them Against Your Rubric (Breakout 3, 20 min)
Prepare Your Sales Pitch for Grant Funding (Breakout 4, 15 min)

## Slide 3
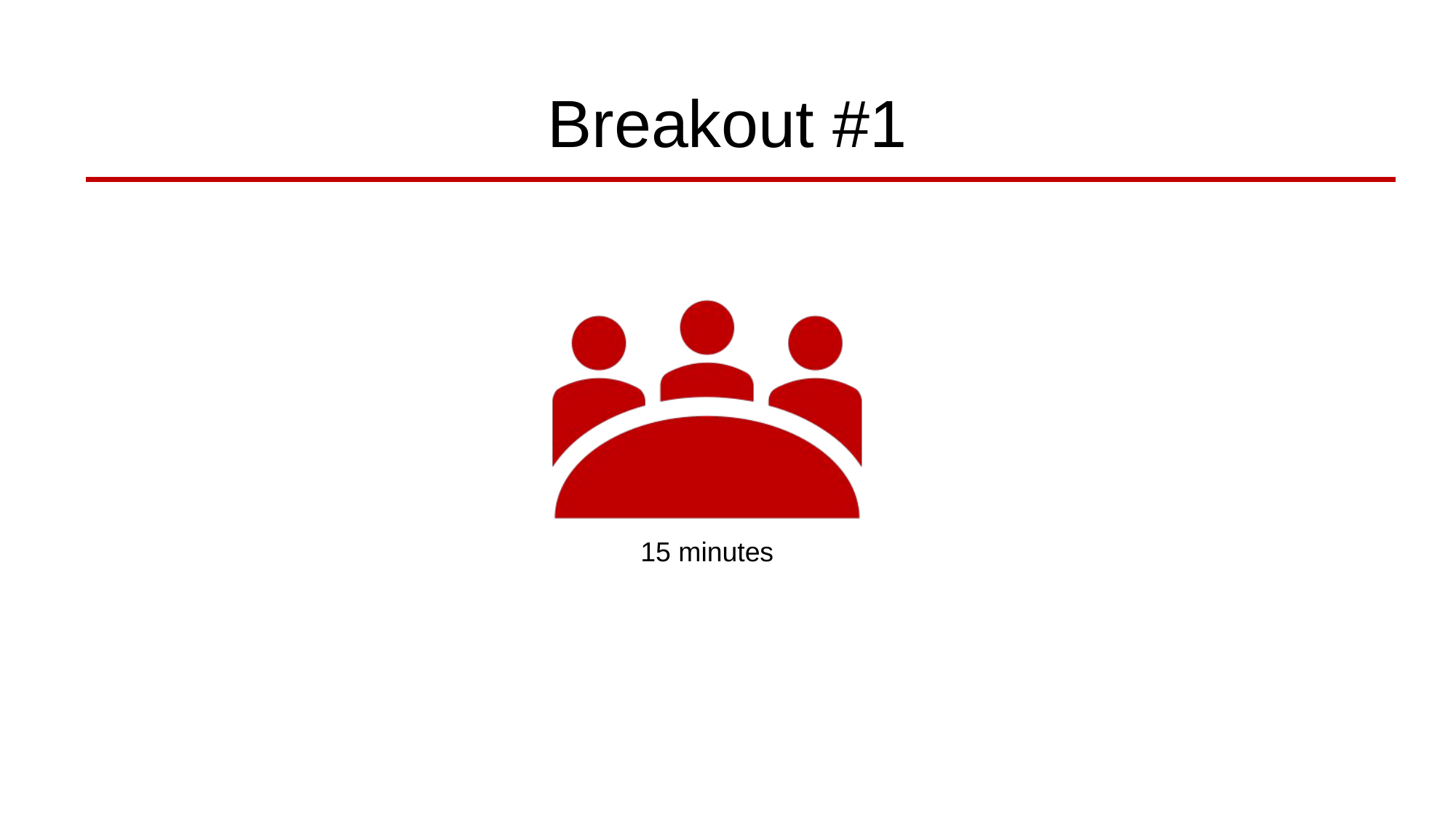

# Breakout #1
15 minutes

## Slide 4
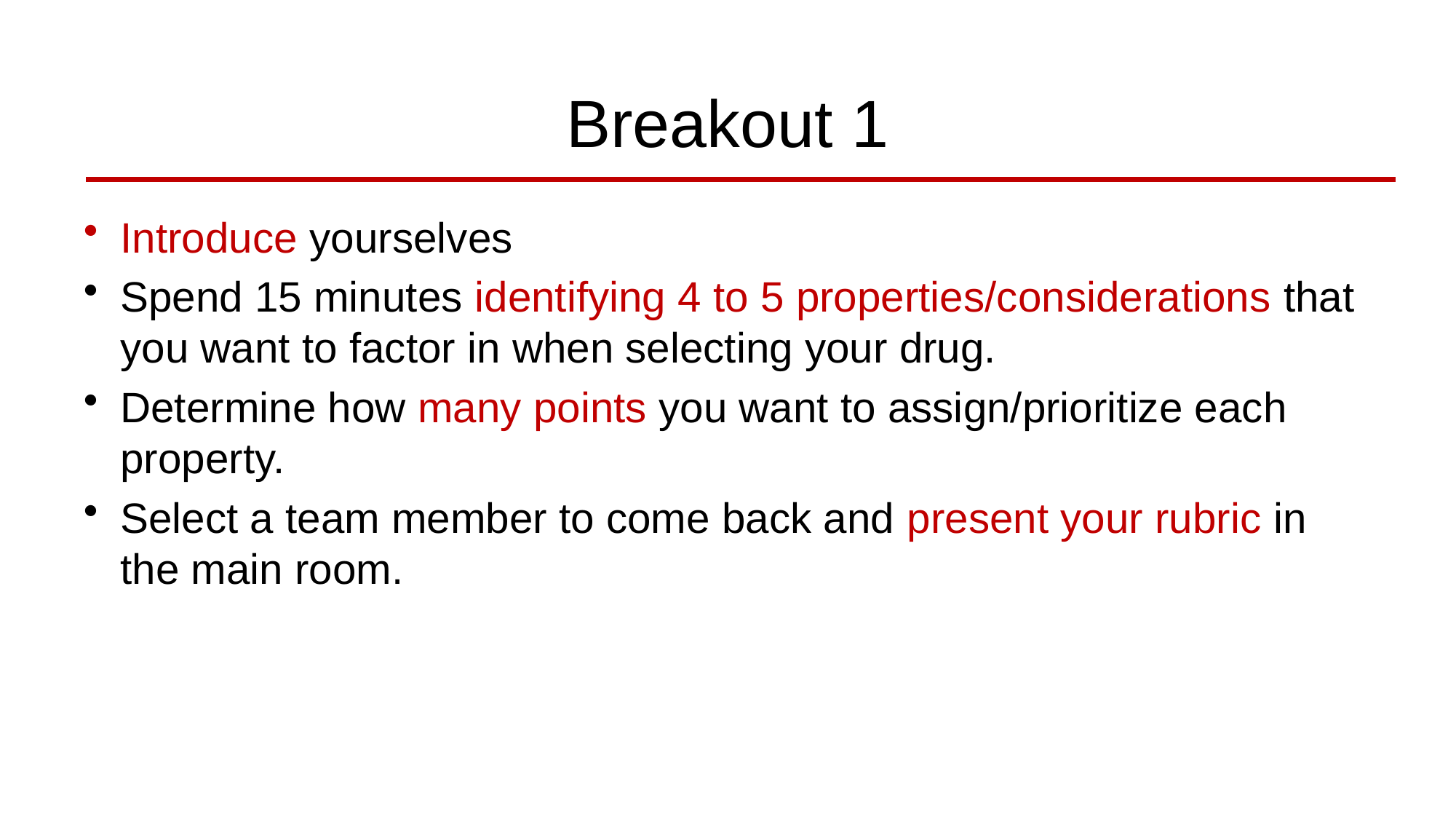

# Breakout 1
Introduce yourselves
Spend 15 minutes identifying 4 to 5 properties/considerations that you want to factor in when selecting your drug.
Determine how many points you want to assign/prioritize each property.
Select a team member to come back and present your rubric in the main room.

## Slide 5
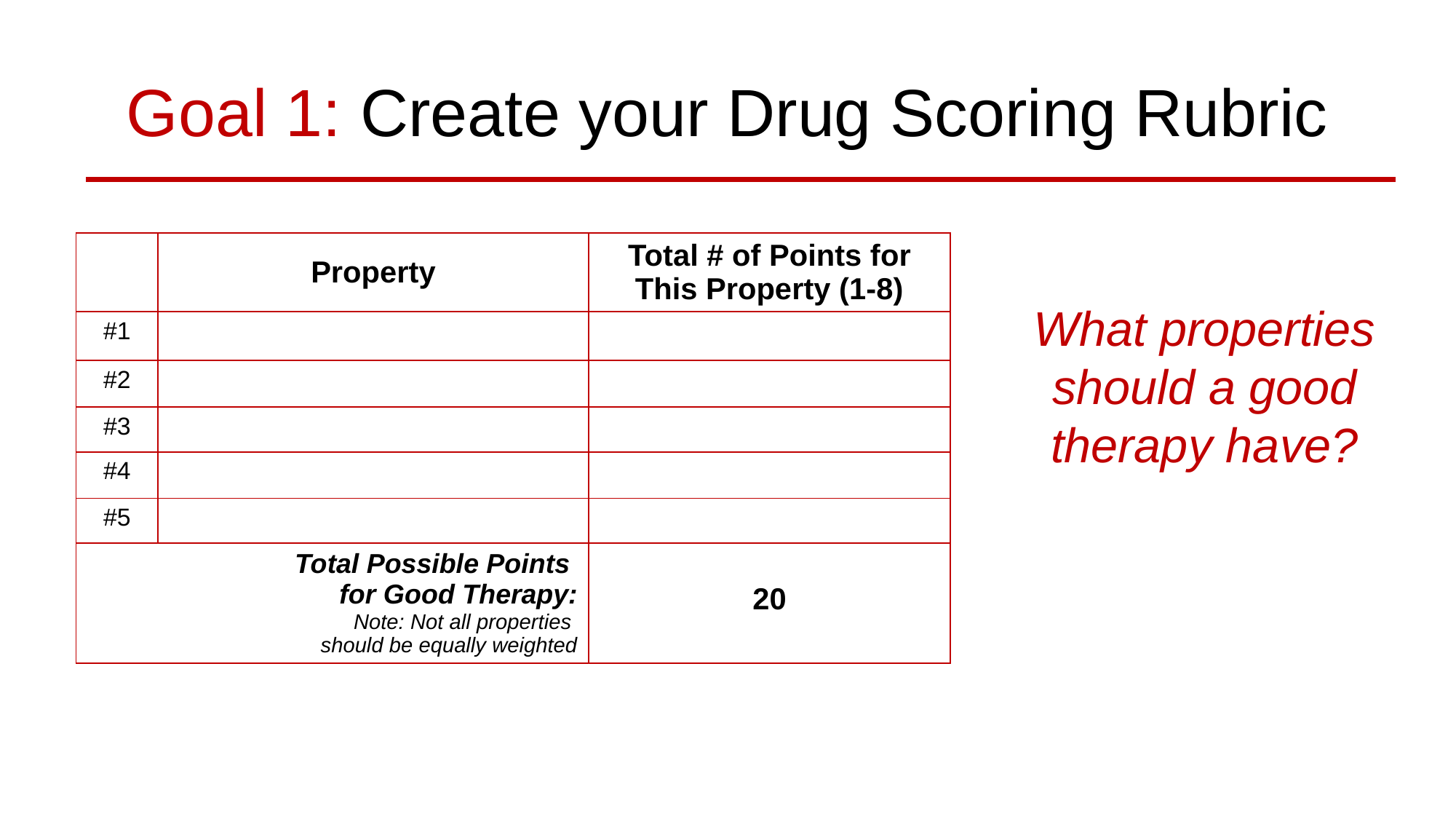

# Goal 1: Create your Drug Scoring Rubric
| | Property | Total # of Points for This Property (1-8) |
| --- | --- | --- |
| #1 | | |
| #2 | | |
| #3 | | |
| #4 | | |
| #5 | | |
| Total Possible Points for Good Therapy: Note: Not all properties should be equally weighted | | 20 |
What properties should a good therapy have?

## Slide 6
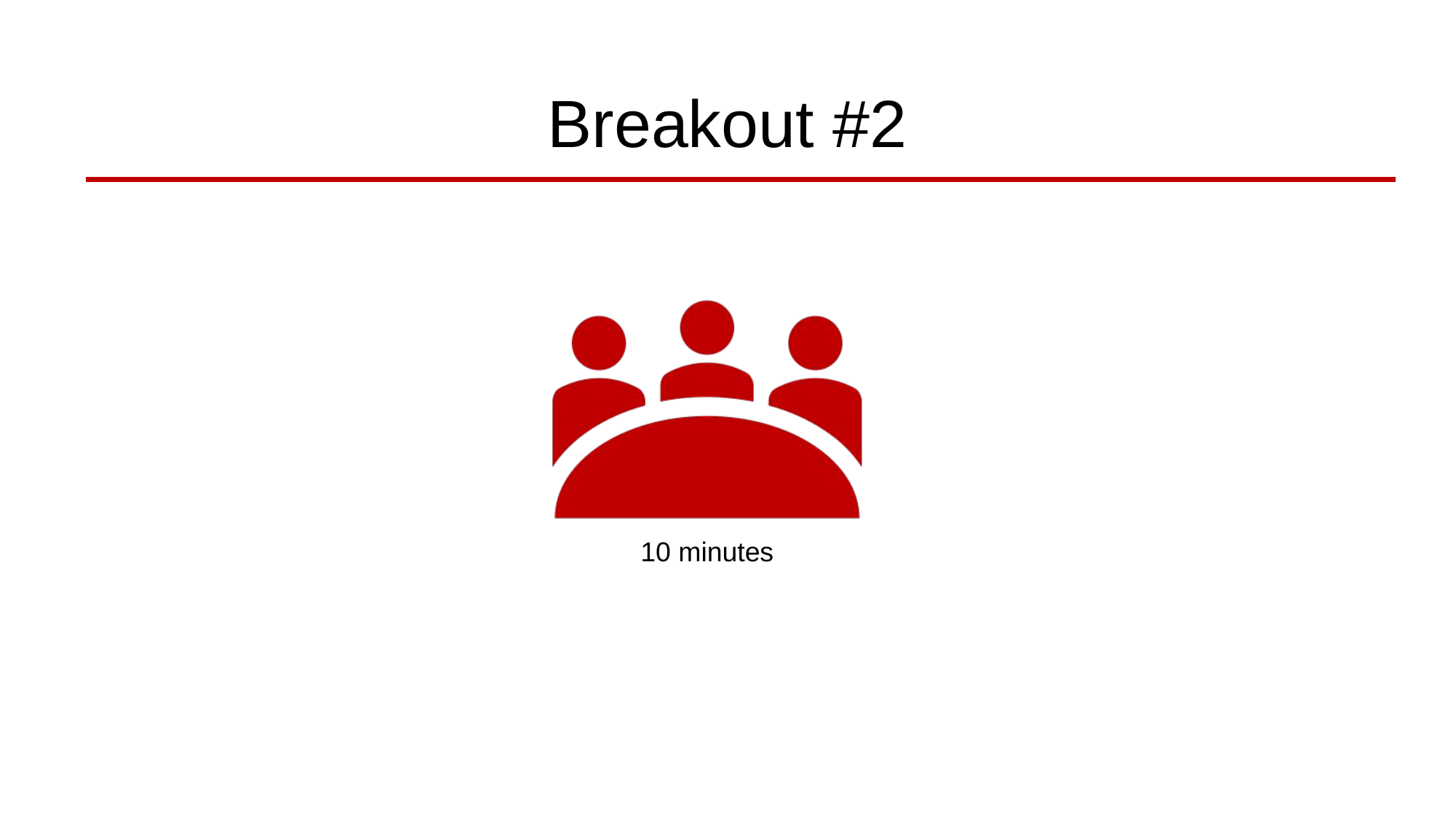

# Breakout #2
10 minutes

## Slide 7
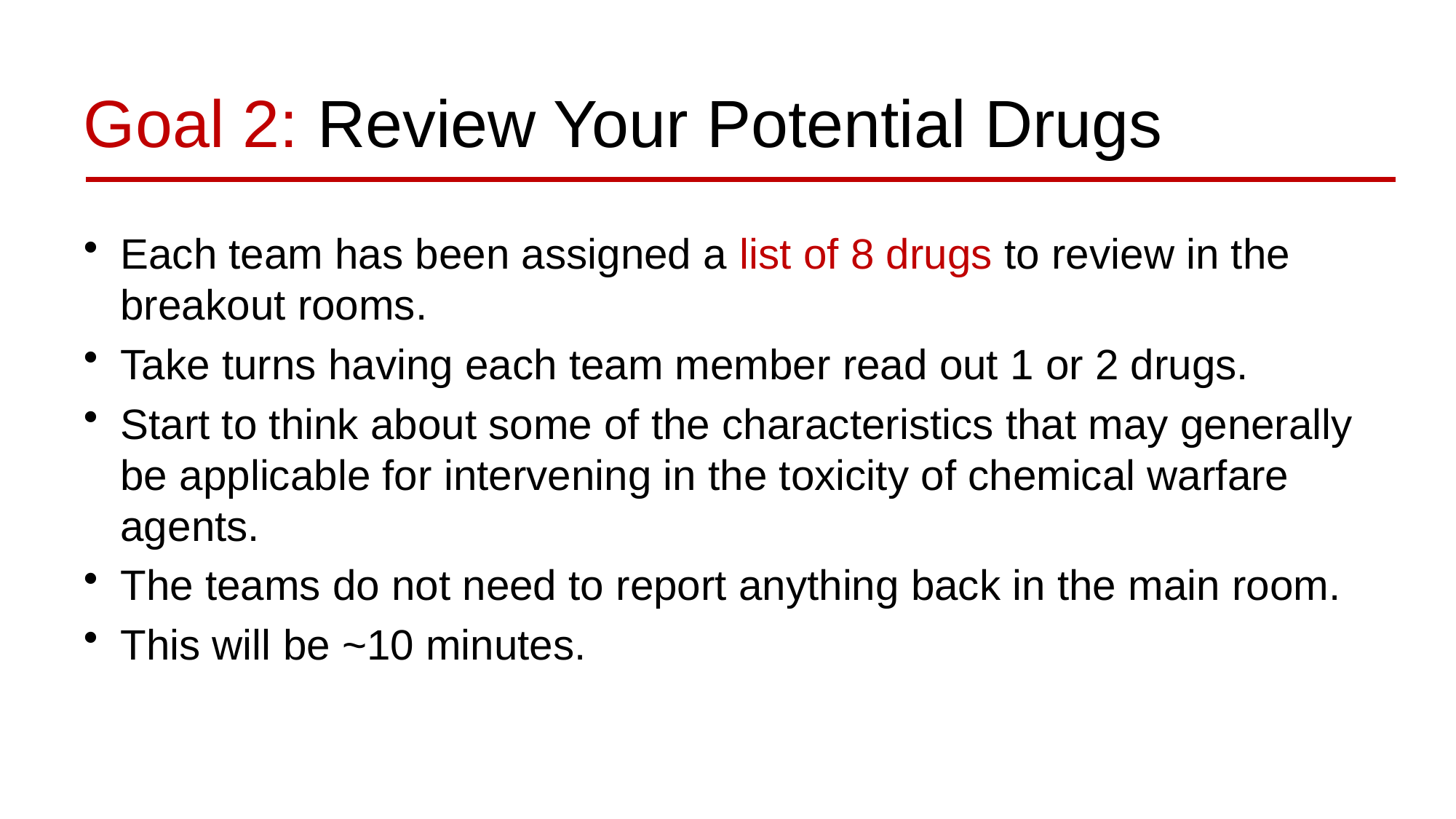

# Goal 2: Review Your Potential Drugs
Each team has been assigned a list of 8 drugs to review in the breakout rooms.
Take turns having each team member read out 1 or 2 drugs.
Start to think about some of the characteristics that may generally be applicable for intervening in the toxicity of chemical warfare agents.
The teams do not need to report anything back in the main room.
This will be ~10 minutes.

## Slide 8
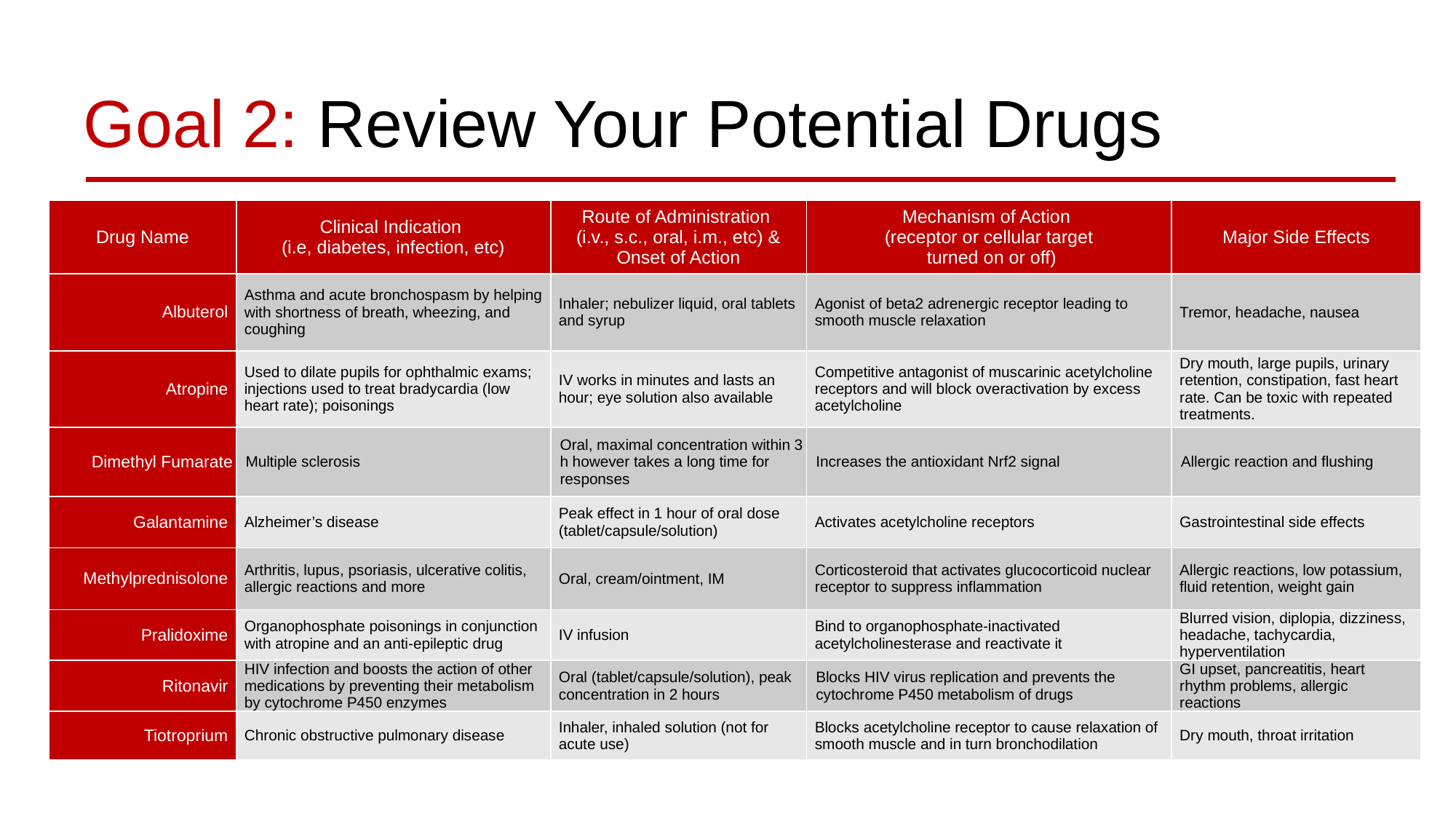

# Goal 2: Review Your Potential Drugs
| Drug Name | Clinical Indication (i.e, diabetes, infection, etc) | Route of Administration (i.v., s.c., oral, i.m., etc) & Onset of Action | Mechanism of Action (receptor or cellular target turned on or off) | Major Side Effects |
| --- | --- | --- | --- | --- |
| Albuterol | Asthma and acute bronchospasm by helping with shortness of breath, wheezing, and coughing | Inhaler; nebulizer liquid, oral tablets and syrup | Agonist of beta2 adrenergic receptor leading to smooth muscle relaxation | Tremor, headache, nausea |
| Atropine | Used to dilate pupils for ophthalmic exams; injections used to treat bradycardia (low heart rate); poisonings | IV works in minutes and lasts an hour; eye solution also available | Competitive antagonist of muscarinic acetylcholine receptors and will block overactivation by excess acetylcholine | Dry mouth, large pupils, urinary retention, constipation, fast heart rate. Can be toxic with repeated treatments. |
| Dimethyl Fumarate | Multiple sclerosis | Oral, maximal concentration within 3 h however takes a long time for responses | Increases the antioxidant Nrf2 signal | Allergic reaction and flushing |
| Galantamine | Alzheimer’s disease | Peak effect in 1 hour of oral dose (tablet/capsule/solution) | Activates acetylcholine receptors | Gastrointestinal side effects |
| Methylprednisolone | Arthritis, lupus, psoriasis, ulcerative colitis, allergic reactions and more | Oral, cream/ointment, IM | Corticosteroid that activates glucocorticoid nuclear receptor to suppress inflammation | Allergic reactions, low potassium, fluid retention, weight gain |
| Pralidoxime | Organophosphate poisonings in conjunction with atropine and an anti-epileptic drug | IV infusion | Bind to organophosphate-inactivated acetylcholinesterase and reactivate it | Blurred vision, diplopia, dizziness, headache, tachycardia, hyperventilation |
| Ritonavir | HIV infection and boosts the action of other medications by preventing their metabolism by cytochrome P450 enzymes | Oral (tablet/capsule/solution), peak concentration in 2 hours | Blocks HIV virus replication and prevents the cytochrome P450 metabolism of drugs | GI upset, pancreatitis, heart rhythm problems, allergic reactions |
| Tiotroprium | Chronic obstructive pulmonary disease | Inhaler, inhaled solution (not for acute use) | Blocks acetylcholine receptor to cause relaxation of smooth muscle and in turn bronchodilation | Dry mouth, throat irritation |

## Slide 9
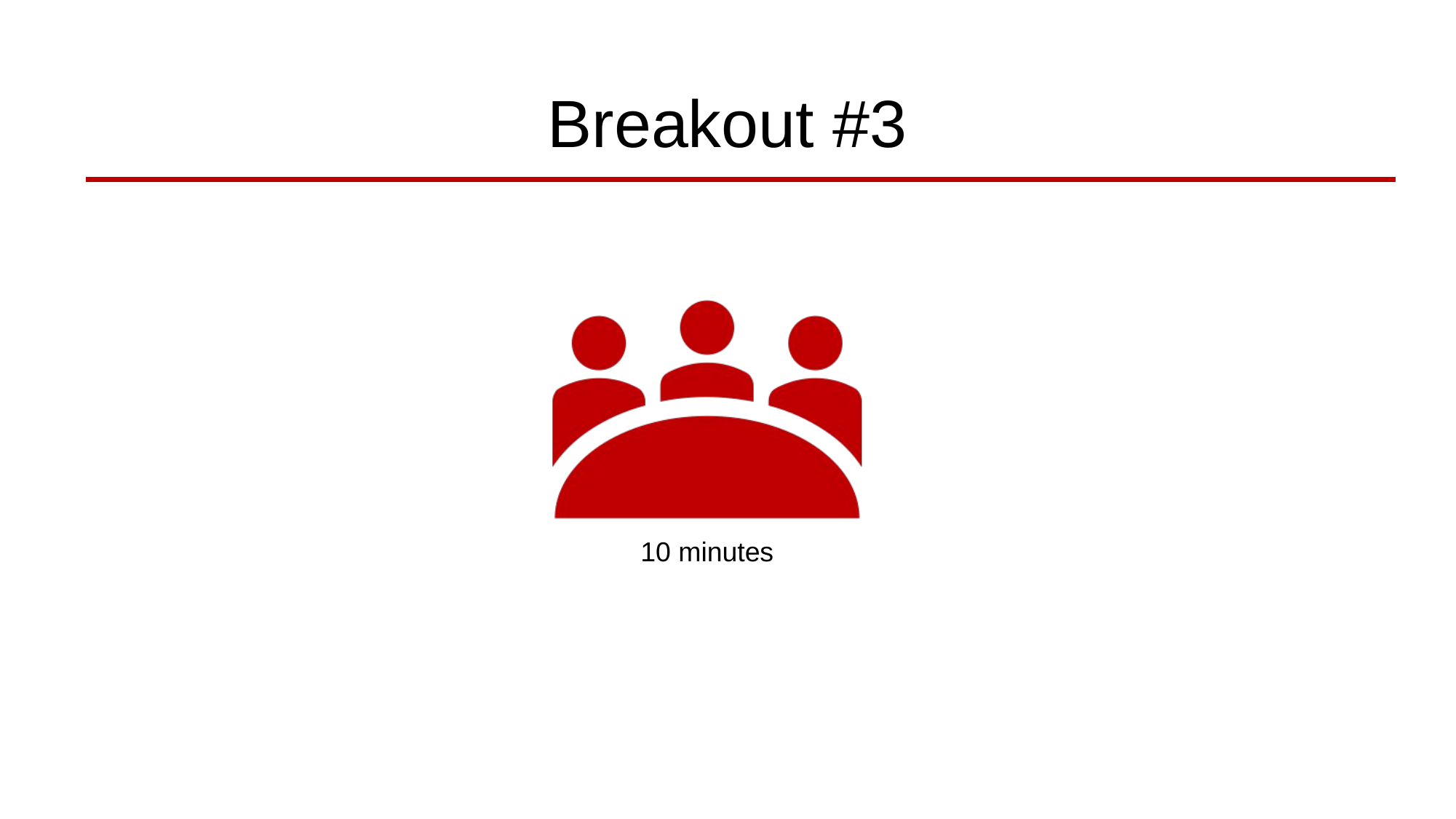

# Breakout #3
10 minutes

## Slide 10
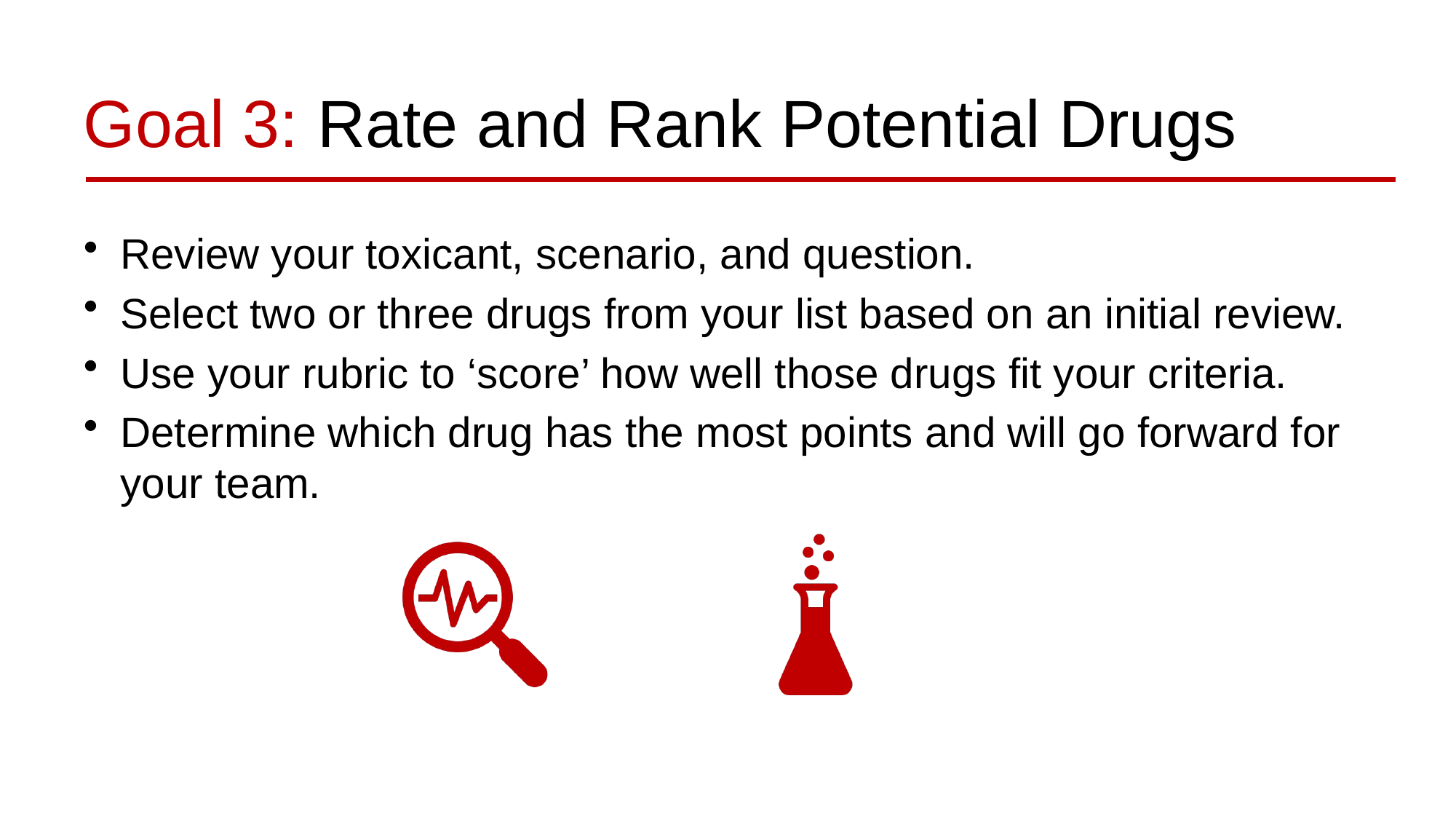

# Goal 3: Rate and Rank Potential Drugs
Review your toxicant, scenario, and question.
Select two or three drugs from your list based on an initial review.
Use your rubric to ‘score’ how well those drugs fit your criteria.
Determine which drug has the most points and will go forward for your team.

## Slide 11
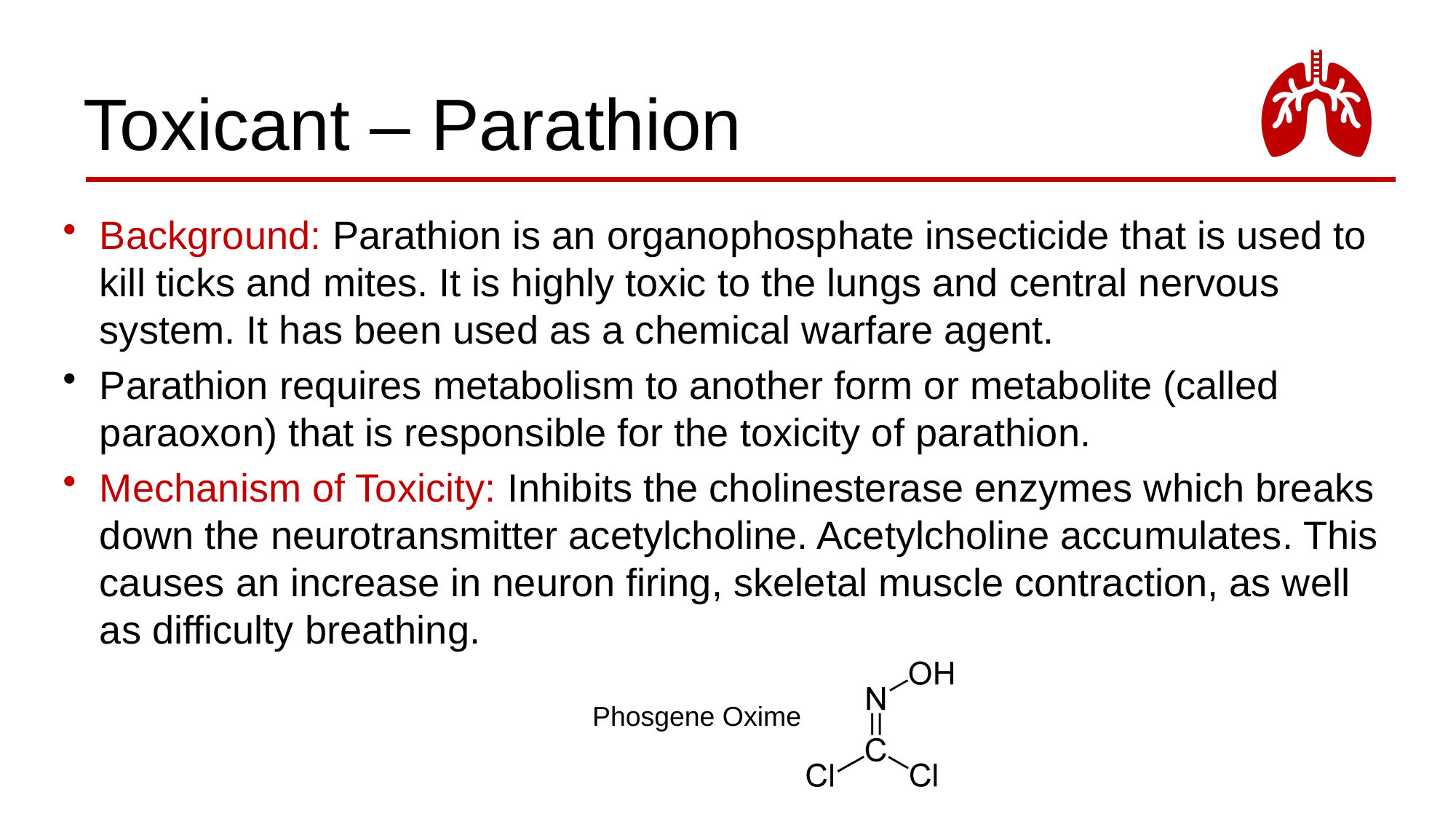

# Toxicant – Parathion
Background: Parathion is an organophosphate insecticide that is used to kill ticks and mites. It is highly toxic to the lungs and central nervous system. It has been used as a chemical warfare agent.
Parathion requires metabolism to another form or metabolite (called paraoxon) that is responsible for the toxicity of parathion.
Mechanism of Toxicity: Inhibits the cholinesterase enzymes which breaks down the neurotransmitter acetylcholine. Acetylcholine accumulates. This causes an increase in neuron firing, skeletal muscle contraction, as well as difficulty breathing.
Phosgene Oxime

## Slide 12
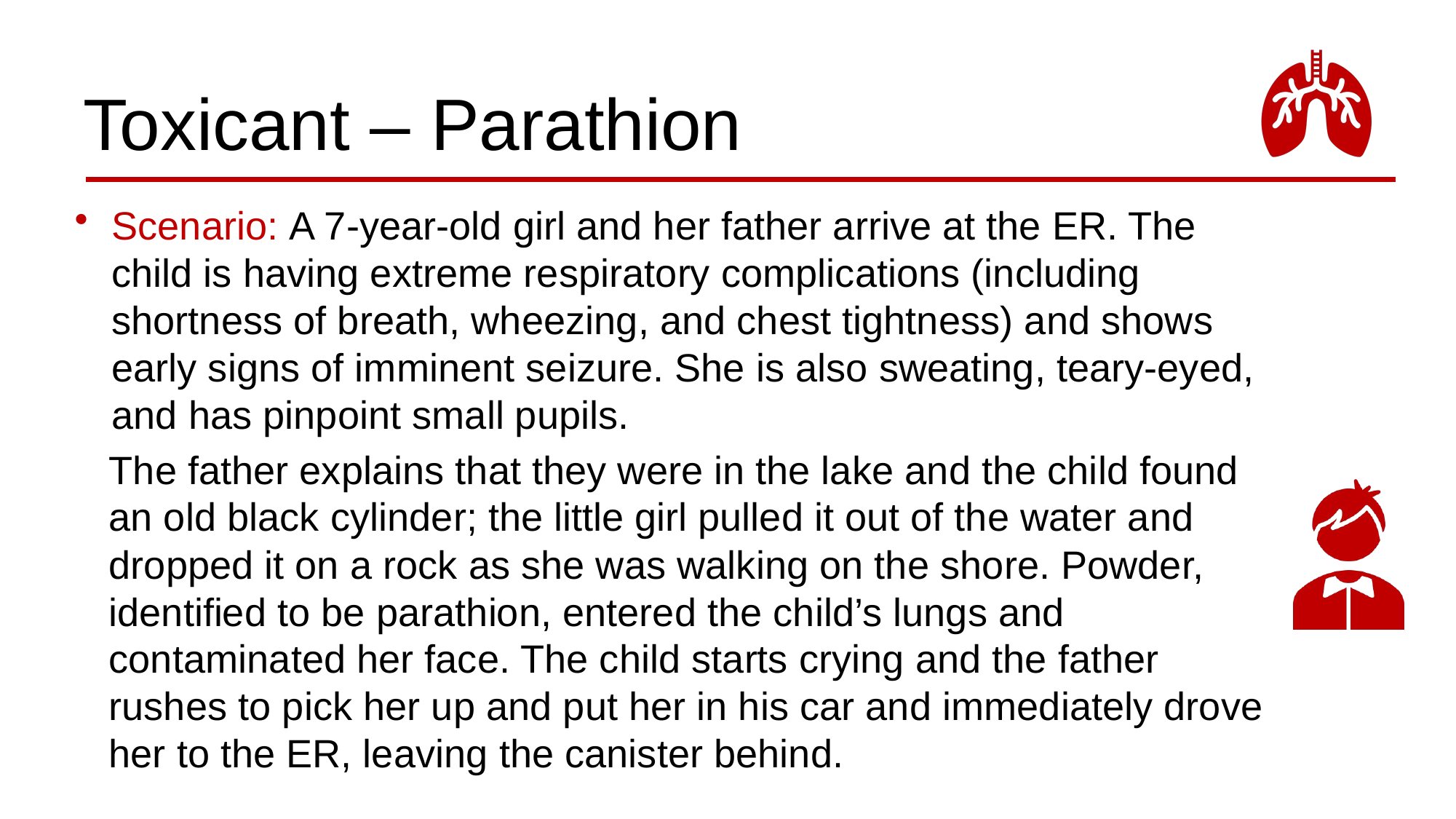

# Toxicant – Parathion
Scenario: A 7-year-old girl and her father arrive at the ER. The child is having extreme respiratory complications (including shortness of breath, wheezing, and chest tightness) and shows early signs of imminent seizure. She is also sweating, teary-eyed, and has pinpoint small pupils.
The father explains that they were in the lake and the child found an old black cylinder; the little girl pulled it out of the water and dropped it on a rock as she was walking on the shore. Powder, identified to be parathion, entered the child’s lungs and contaminated her face. The child starts crying and the father rushes to pick her up and put her in his car and immediately drove her to the ER, leaving the canister behind.

## Slide 13
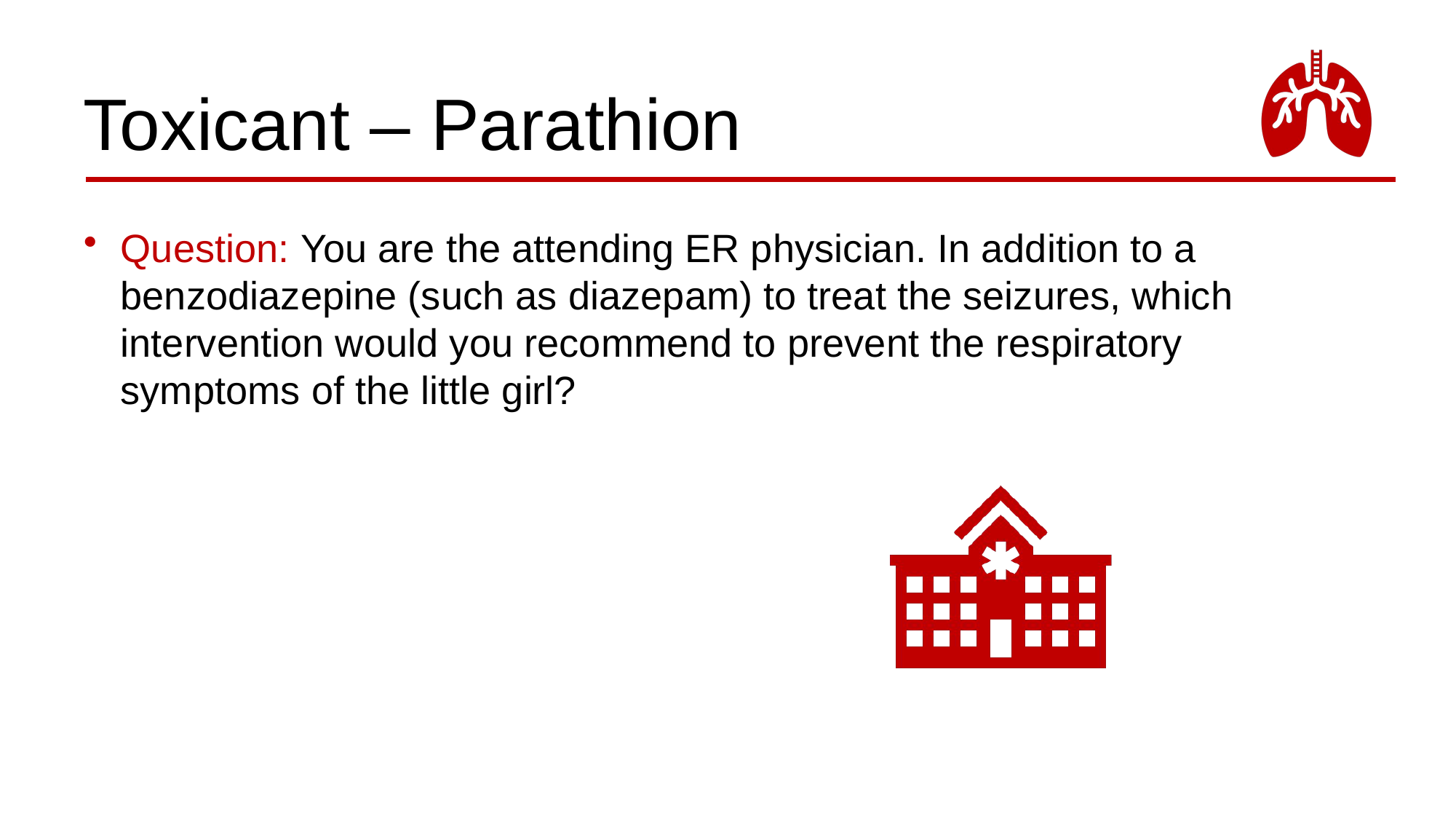

# Toxicant – Parathion
Question: You are the attending ER physician. In addition to a benzodiazepine (such as diazepam) to treat the seizures, which intervention would you recommend to prevent the respiratory symptoms of the little girl?

## Slide 14
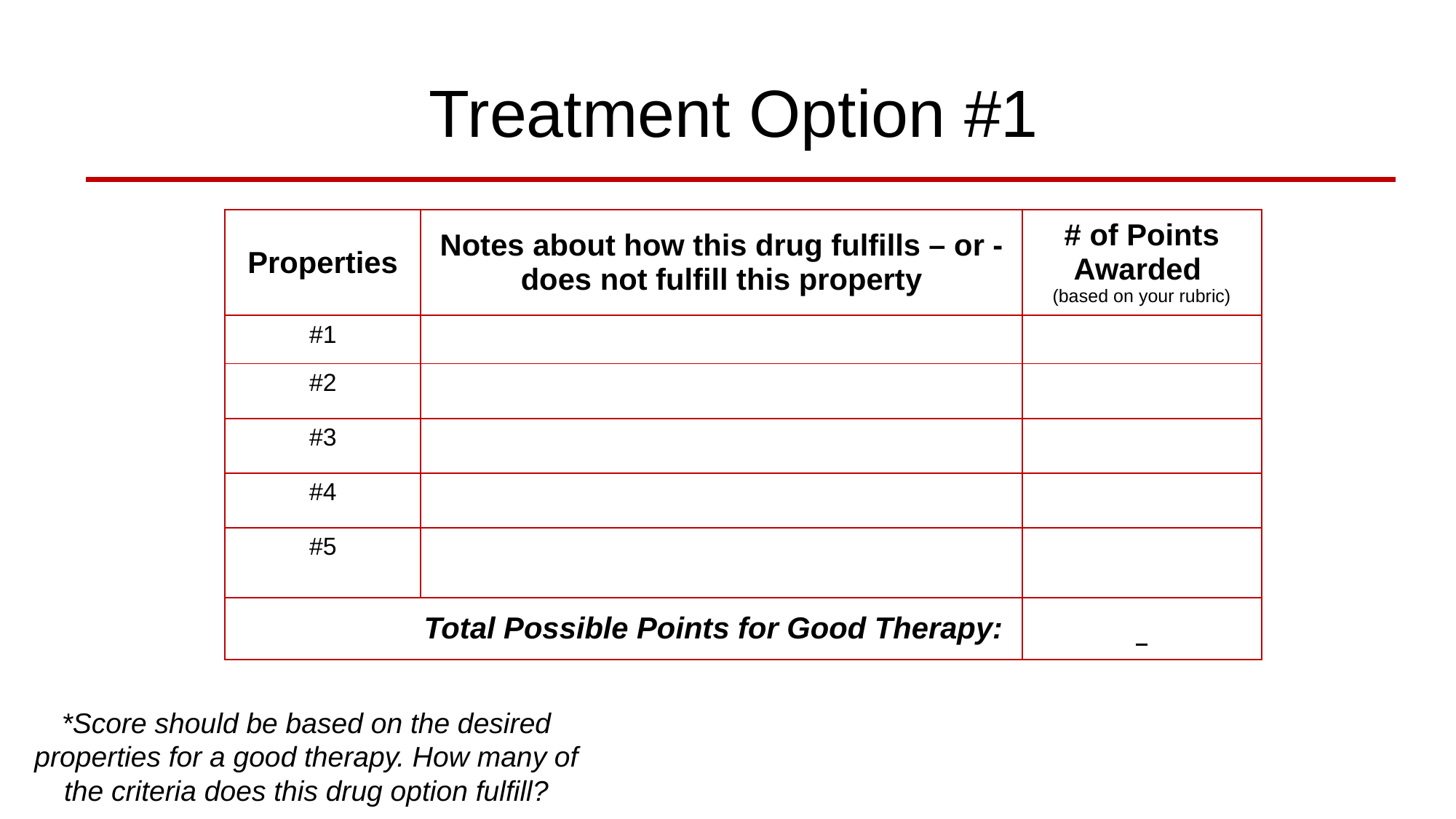

# Treatment Option #1
| Properties | Notes about how this drug fulfills – or -does not fulfill this property | # of Points Awarded (based on your rubric) |
| --- | --- | --- |
| #1 | | |
| #2 | | |
| #3 | | |
| #4 | | |
| #5 | | |
| Total Possible Points for Good Therapy: | | |
*Score should be based on the desired properties for a good therapy. How many of the criteria does this drug option fulfill?

## Slide 15
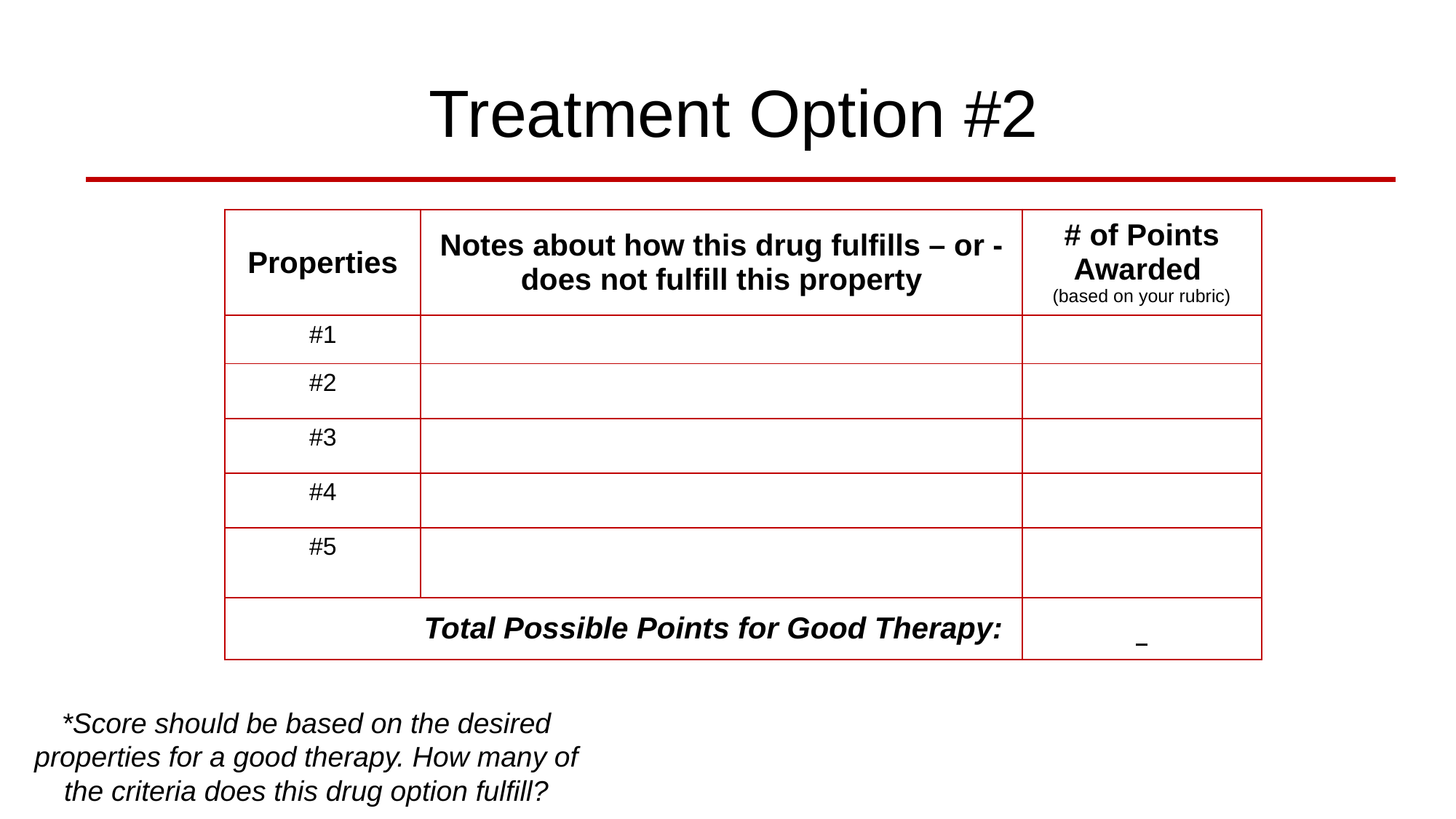

# Treatment Option #2
| Properties | Notes about how this drug fulfills – or -does not fulfill this property | # of Points Awarded (based on your rubric) |
| --- | --- | --- |
| #1 | | |
| #2 | | |
| #3 | | |
| #4 | | |
| #5 | | |
| Total Possible Points for Good Therapy: | | |
*Score should be based on the desired properties for a good therapy. How many of the criteria does this drug option fulfill?

## Slide 16
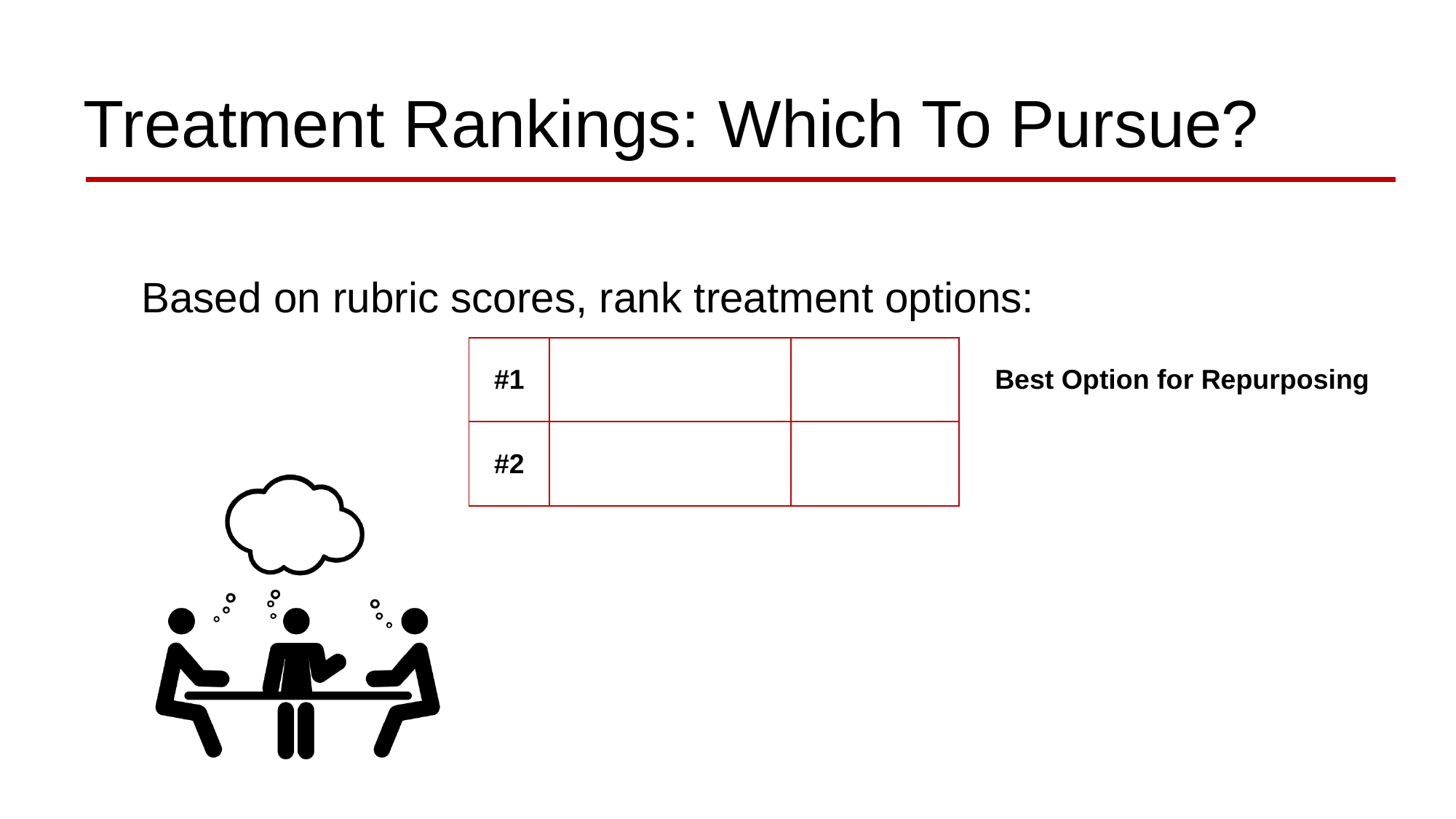

# Treatment Rankings: Which To Pursue?
Based on rubric scores, rank treatment options:
| #1 | | |
| --- | --- | --- |
| #2 | | |
Best Option for Repurposing

## Slide 17
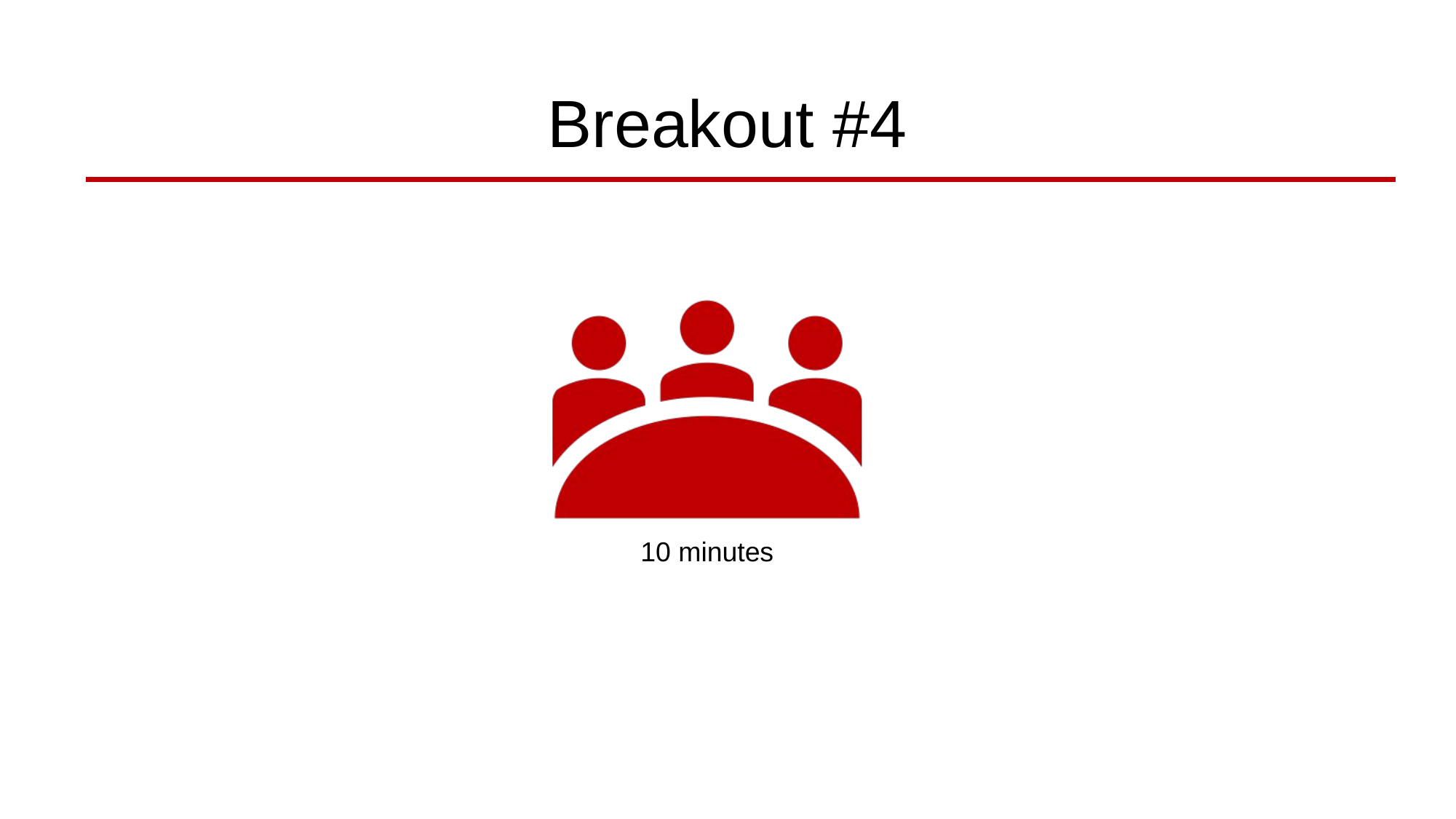

# Breakout #4
10 minutes

## Slide 18
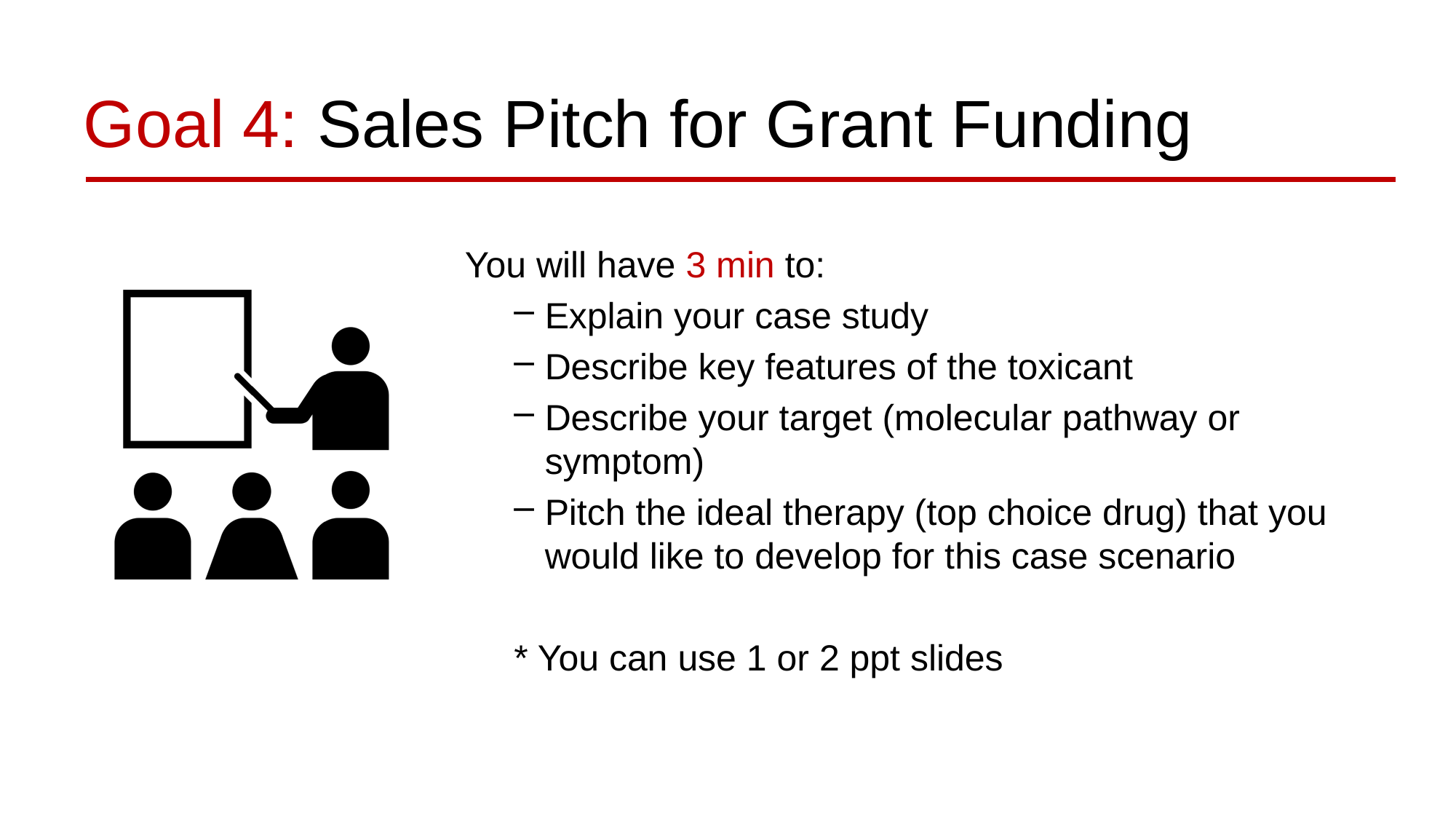

# Goal 4: Sales Pitch for Grant Funding
You will have 3 min to:
Explain your case study
Describe key features of the toxicant
Describe your target (molecular pathway or symptom)
Pitch the ideal therapy (top choice drug) that you would like to develop for this case scenario
* You can use 1 or 2 ppt slides
