# Supplementary material for: Repurposing FDA‐approved drugs to treat chemical weapon toxicities: Interactive case studies for trainees
Source: Pharmacol Res Perspect. 2024 Jul 4;12(4):e1229. doi: 10.1002/prp2.1229 (PMC11223991; doi:10.1002/prp2.1229)
Supplement: Supplementary file 3 — File S3. [file PRP2-12-e1229-s004.zip › Supporting File S6 - Case 4.pptx]

## Slide 1
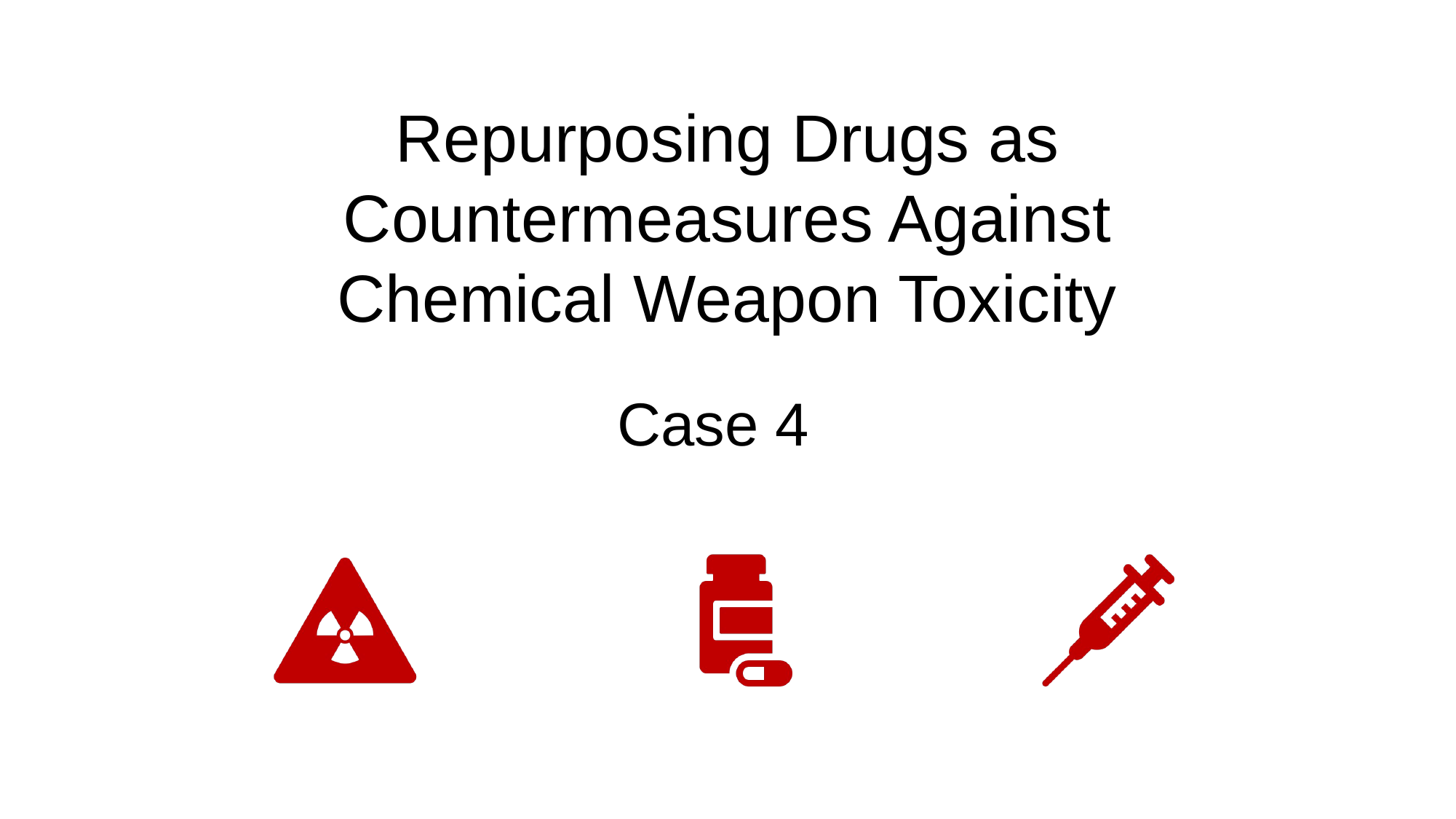

Repurposing Drugs as Countermeasures Against Chemical Weapon Toxicity
Case 4

## Slide 2
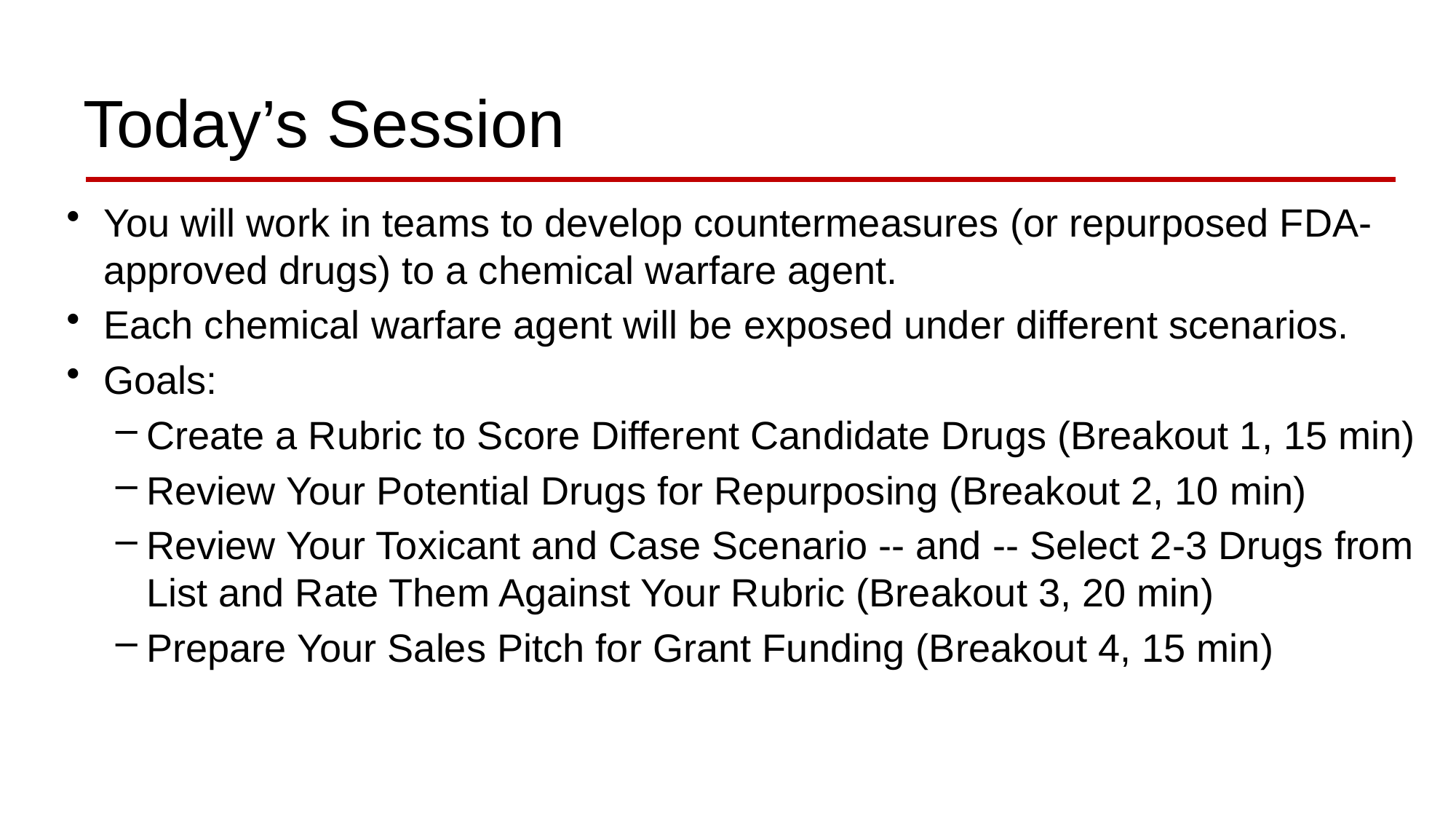

# Today’s Session
You will work in teams to develop countermeasures (or repurposed FDA-approved drugs) to a chemical warfare agent.
Each chemical warfare agent will be exposed under different scenarios.
Goals:
Create a Rubric to Score Different Candidate Drugs (Breakout 1, 15 min)
Review Your Potential Drugs for Repurposing (Breakout 2, 10 min)
Review Your Toxicant and Case Scenario -- and -- Select 2-3 Drugs from List and Rate Them Against Your Rubric (Breakout 3, 20 min)
Prepare Your Sales Pitch for Grant Funding (Breakout 4, 15 min)

## Slide 3
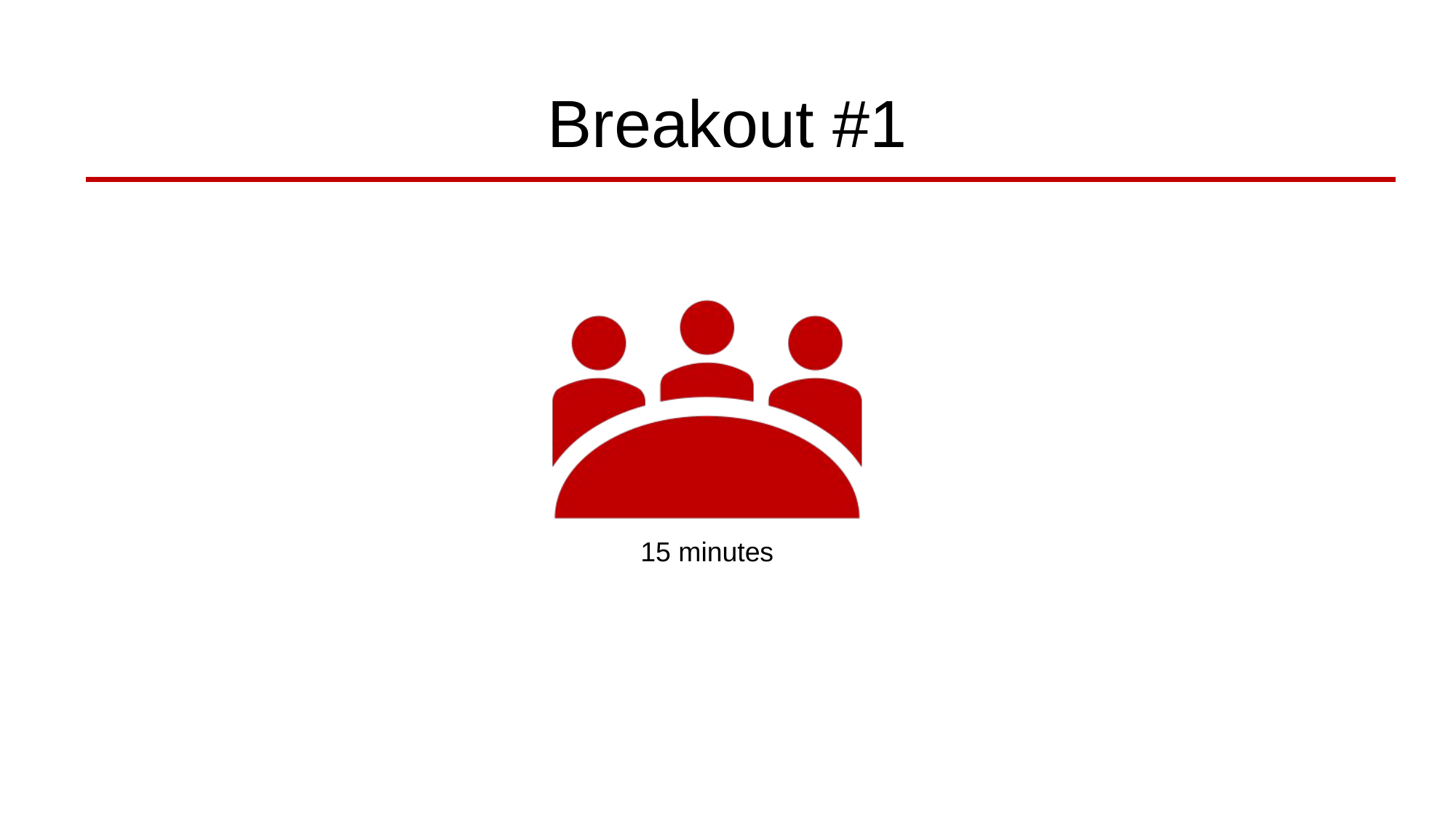

# Breakout #1
15 minutes

## Slide 4
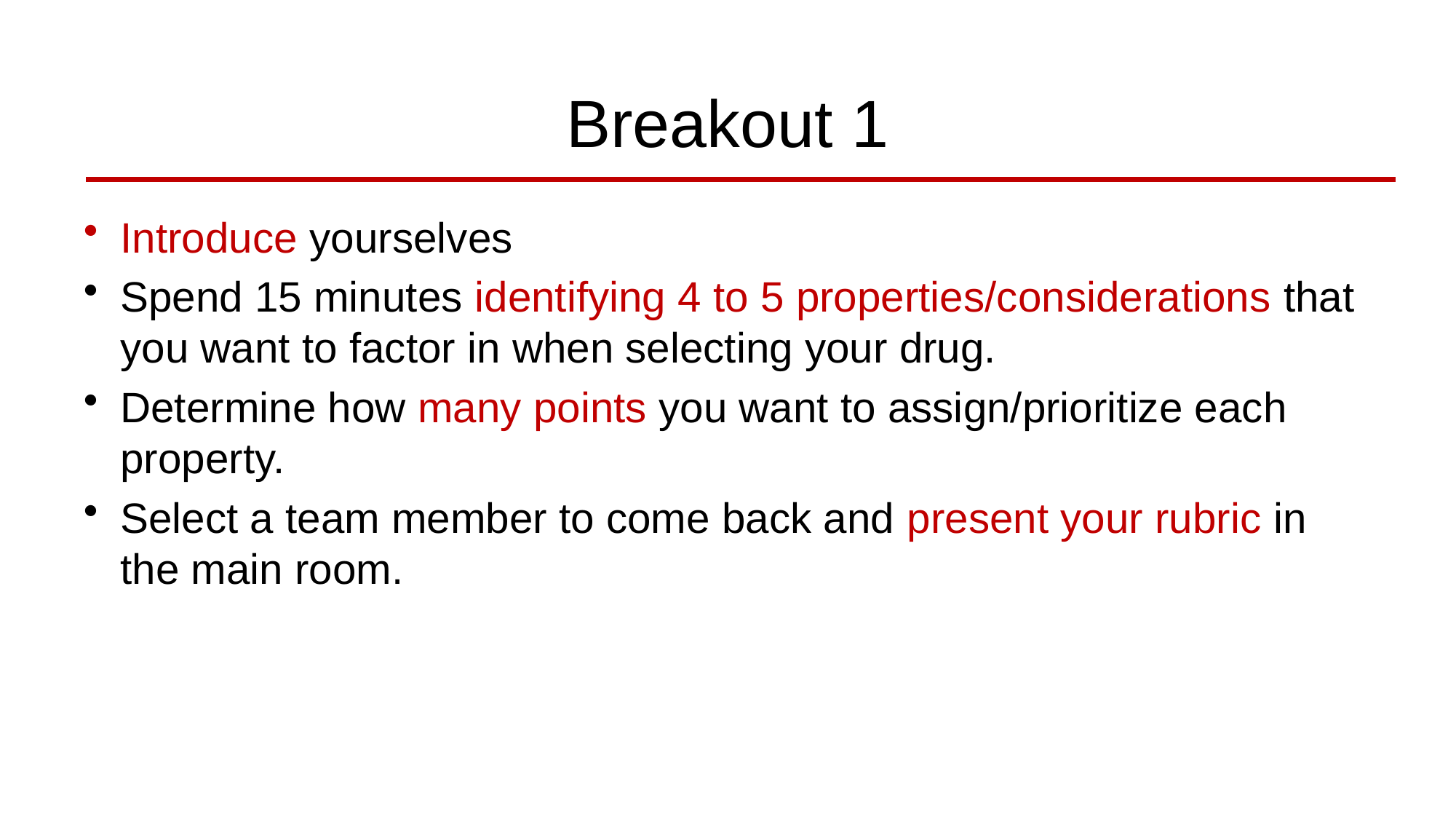

# Breakout 1
Introduce yourselves
Spend 15 minutes identifying 4 to 5 properties/considerations that you want to factor in when selecting your drug.
Determine how many points you want to assign/prioritize each property.
Select a team member to come back and present your rubric in the main room.

## Slide 5
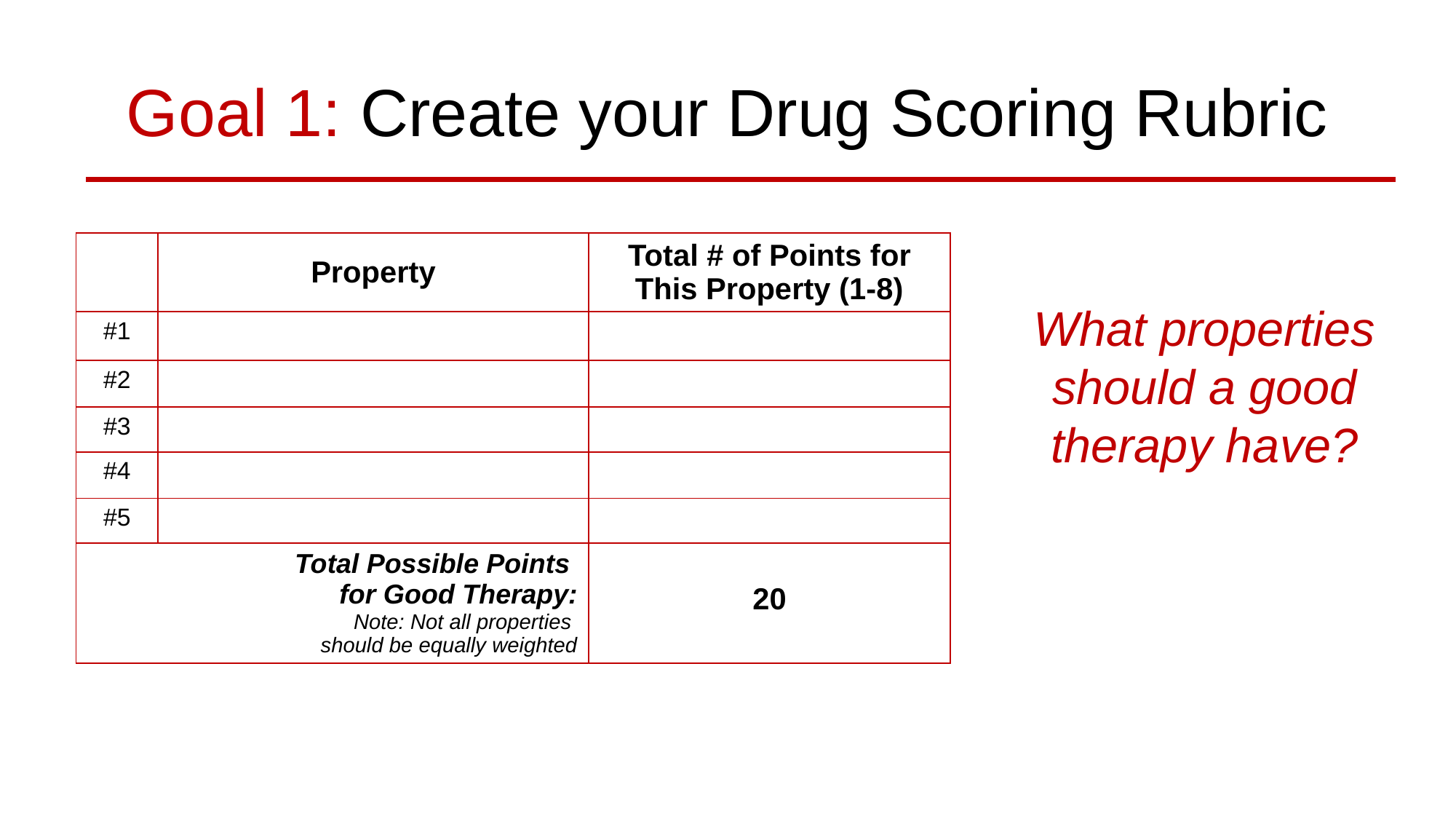

# Goal 1: Create your Drug Scoring Rubric
| | Property | Total # of Points for This Property (1-8) |
| --- | --- | --- |
| #1 | | |
| #2 | | |
| #3 | | |
| #4 | | |
| #5 | | |
| Total Possible Points for Good Therapy: Note: Not all properties should be equally weighted | | 20 |
What properties should a good therapy have?

## Slide 6
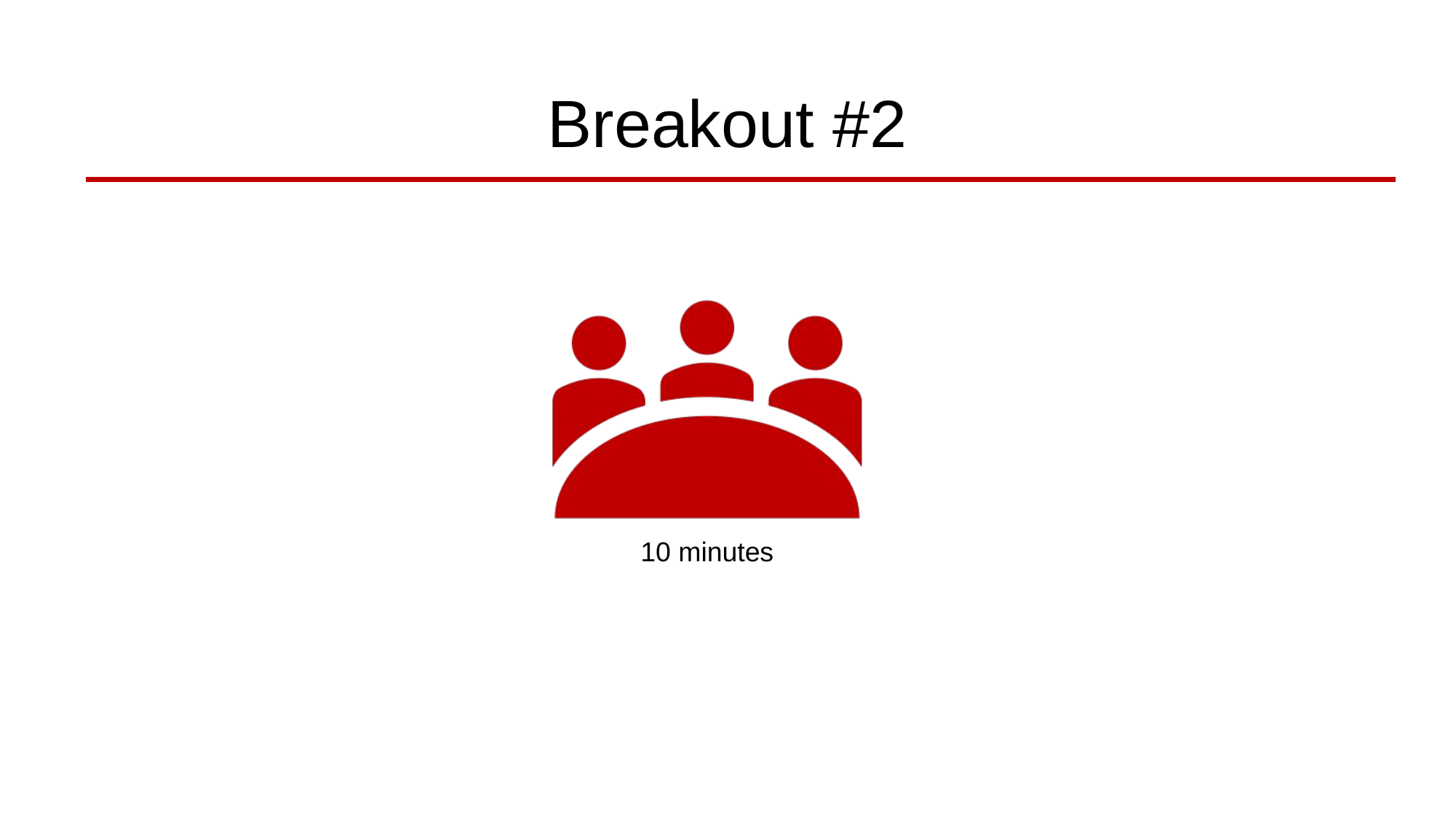

# Breakout #2
10 minutes

## Slide 7
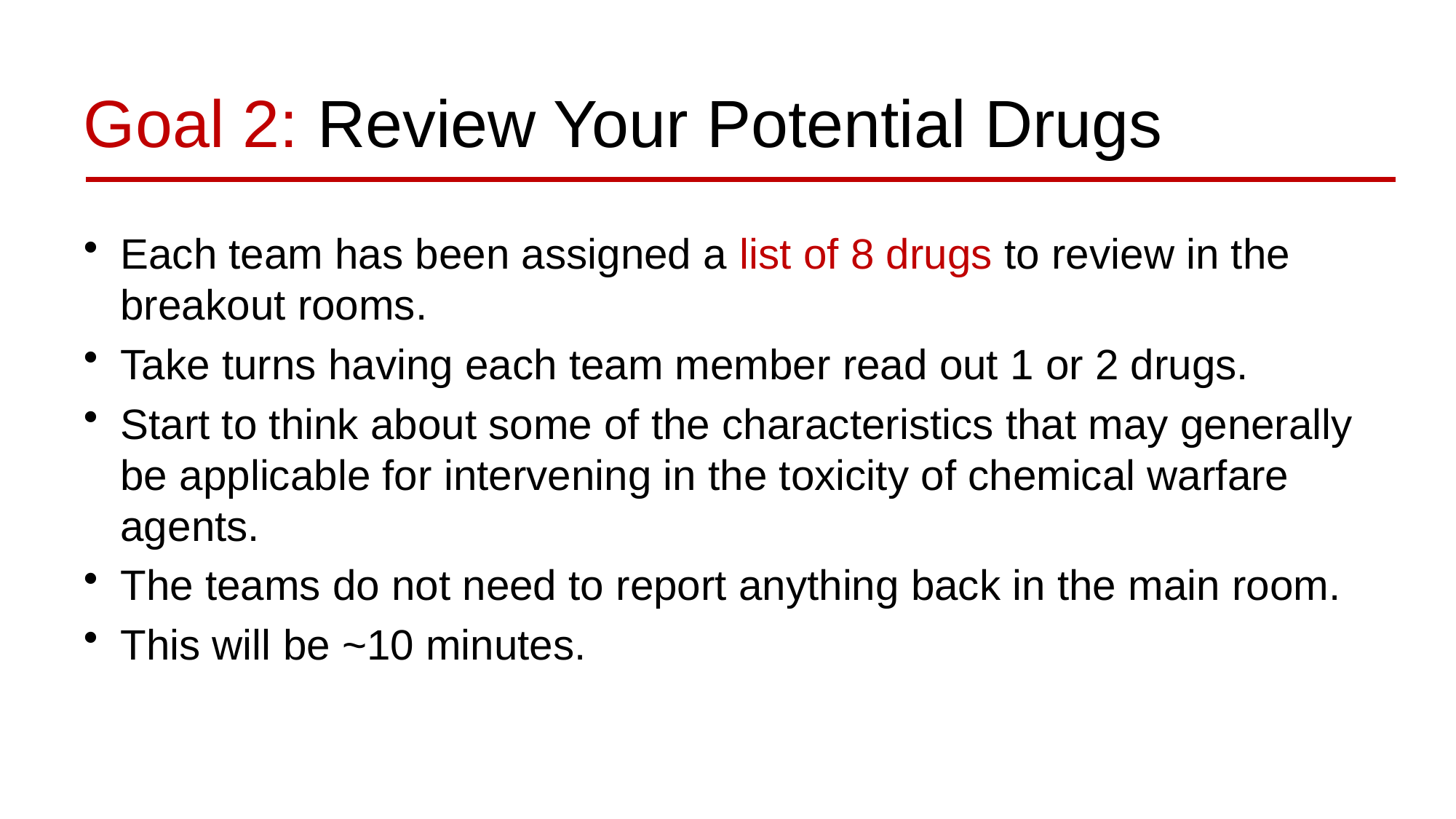

# Goal 2: Review Your Potential Drugs
Each team has been assigned a list of 8 drugs to review in the breakout rooms.
Take turns having each team member read out 1 or 2 drugs.
Start to think about some of the characteristics that may generally be applicable for intervening in the toxicity of chemical warfare agents.
The teams do not need to report anything back in the main room.
This will be ~10 minutes.

## Slide 8
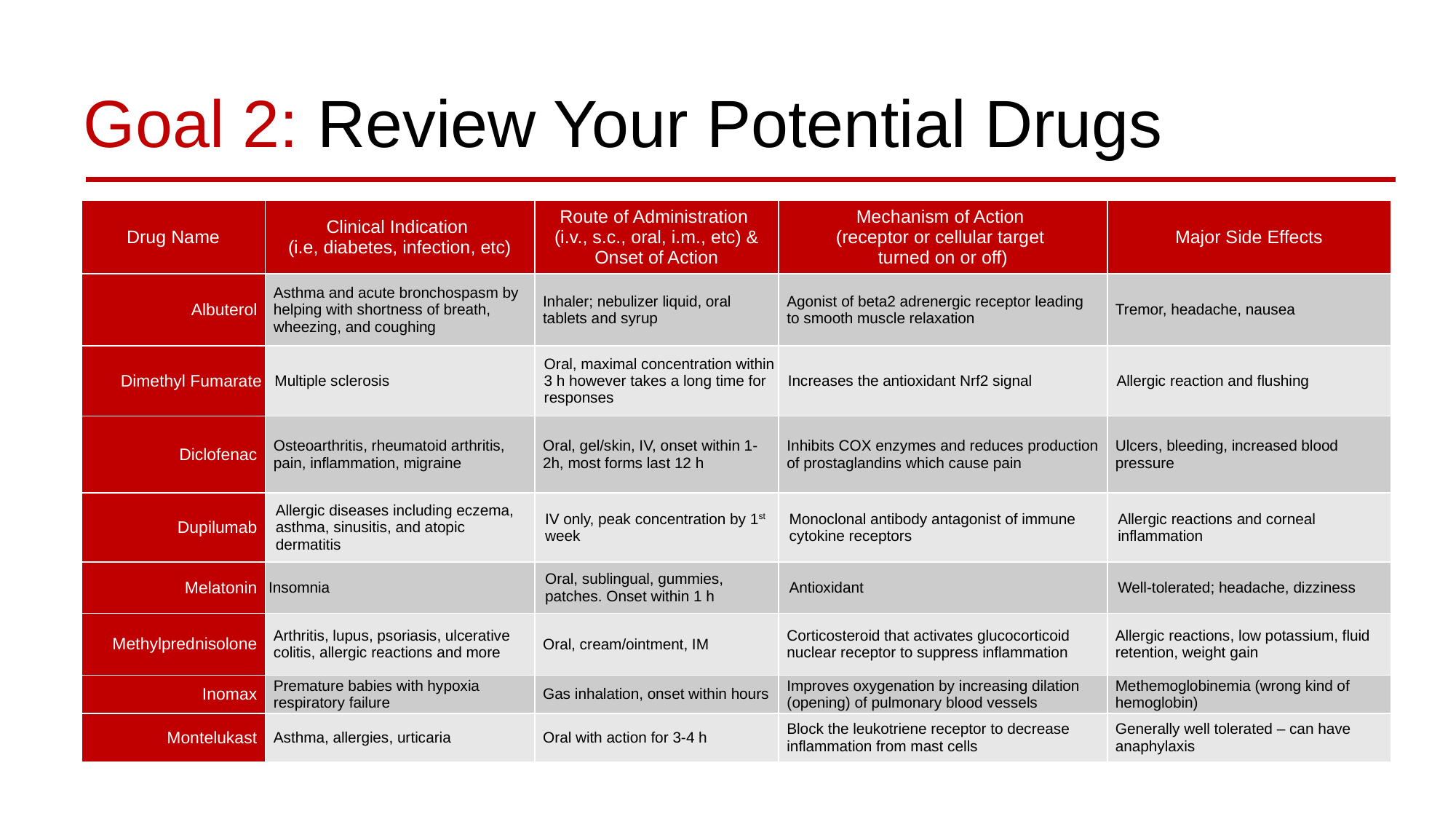

# Goal 2: Review Your Potential Drugs
| Drug Name | Clinical Indication (i.e, diabetes, infection, etc) | Route of Administration (i.v., s.c., oral, i.m., etc) & Onset of Action | Mechanism of Action (receptor or cellular target turned on or off) | Major Side Effects |
| --- | --- | --- | --- | --- |
| Albuterol | Asthma and acute bronchospasm by helping with shortness of breath, wheezing, and coughing | Inhaler; nebulizer liquid, oral tablets and syrup | Agonist of beta2 adrenergic receptor leading to smooth muscle relaxation | Tremor, headache, nausea |
| Dimethyl Fumarate | Multiple sclerosis | Oral, maximal concentration within 3 h however takes a long time for responses | Increases the antioxidant Nrf2 signal | Allergic reaction and flushing |
| Diclofenac | Osteoarthritis, rheumatoid arthritis, pain, inflammation, migraine | Oral, gel/skin, IV, onset within 1-2h, most forms last 12 h | Inhibits COX enzymes and reduces production of prostaglandins which cause pain | Ulcers, bleeding, increased blood pressure |
| Dupilumab | Allergic diseases including eczema, asthma, sinusitis, and atopic dermatitis | IV only, peak concentration by 1st week | Monoclonal antibody antagonist of immune cytokine receptors | Allergic reactions and corneal inflammation |
| Melatonin | Insomnia | Oral, sublingual, gummies, patches. Onset within 1 h | Antioxidant | Well-tolerated; headache, dizziness |
| Methylprednisolone | Arthritis, lupus, psoriasis, ulcerative colitis, allergic reactions and more | Oral, cream/ointment, IM | Corticosteroid that activates glucocorticoid nuclear receptor to suppress inflammation | Allergic reactions, low potassium, fluid retention, weight gain |
| Inomax | Premature babies with hypoxia respiratory failure | Gas inhalation, onset within hours | Improves oxygenation by increasing dilation (opening) of pulmonary blood vessels | Methemoglobinemia (wrong kind of hemoglobin) |
| Montelukast | Asthma, allergies, urticaria | Oral with action for 3-4 h | Block the leukotriene receptor to decrease inflammation from mast cells | Generally well tolerated – can have anaphylaxis |

## Slide 9
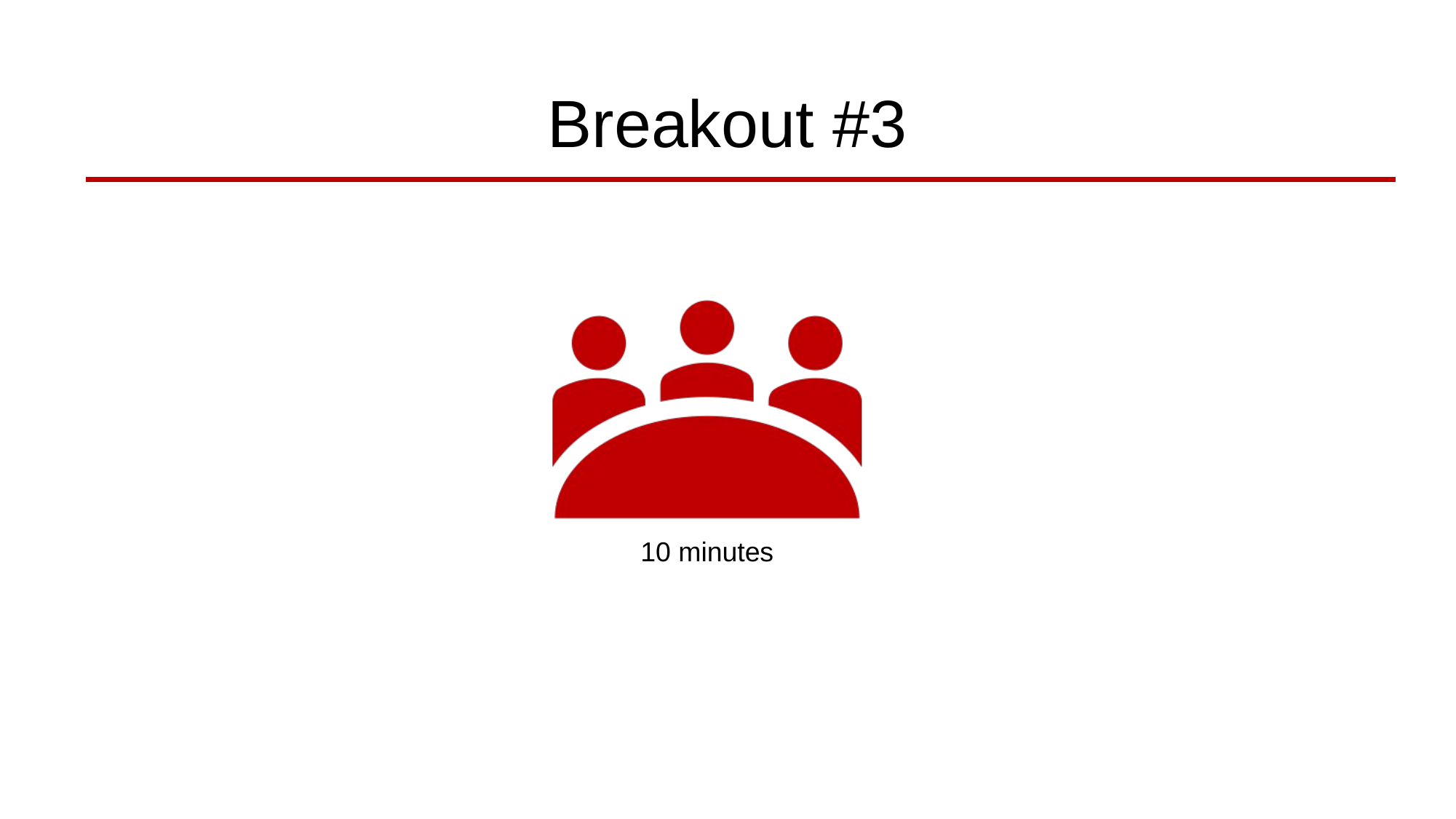

# Breakout #3
10 minutes

## Slide 10
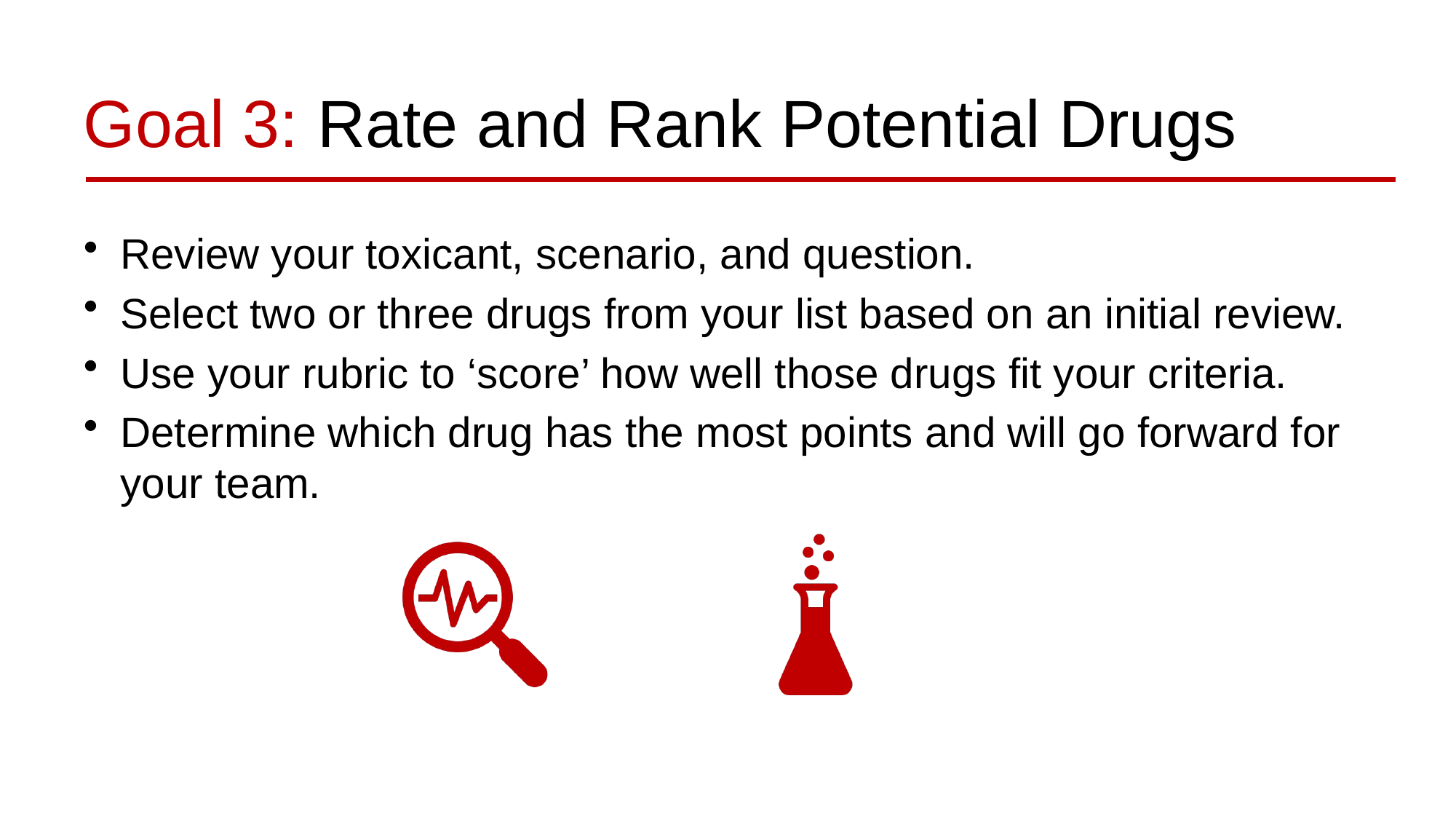

# Goal 3: Rate and Rank Potential Drugs
Review your toxicant, scenario, and question.
Select two or three drugs from your list based on an initial review.
Use your rubric to ‘score’ how well those drugs fit your criteria.
Determine which drug has the most points and will go forward for your team.

## Slide 11
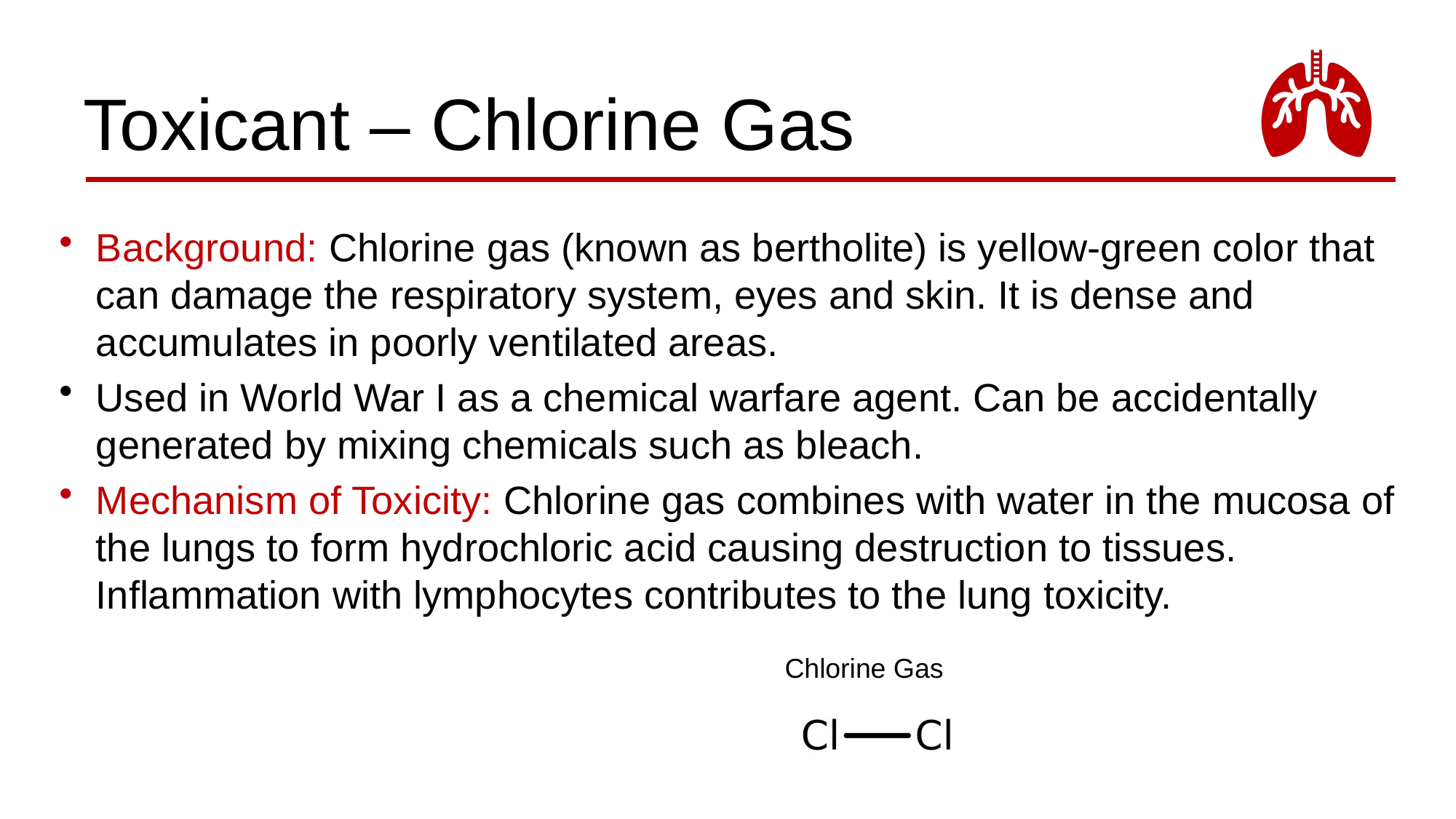

# Toxicant – Chlorine Gas
Background: Chlorine gas (known as bertholite) is yellow-green color that can damage the respiratory system, eyes and skin. It is dense and accumulates in poorly ventilated areas.
Used in World War I as a chemical warfare agent. Can be accidentally generated by mixing chemicals such as bleach.
Mechanism of Toxicity: Chlorine gas combines with water in the mucosa of the lungs to form hydrochloric acid causing destruction to tissues. Inflammation with lymphocytes contributes to the lung toxicity.
Chlorine Gas

## Slide 12
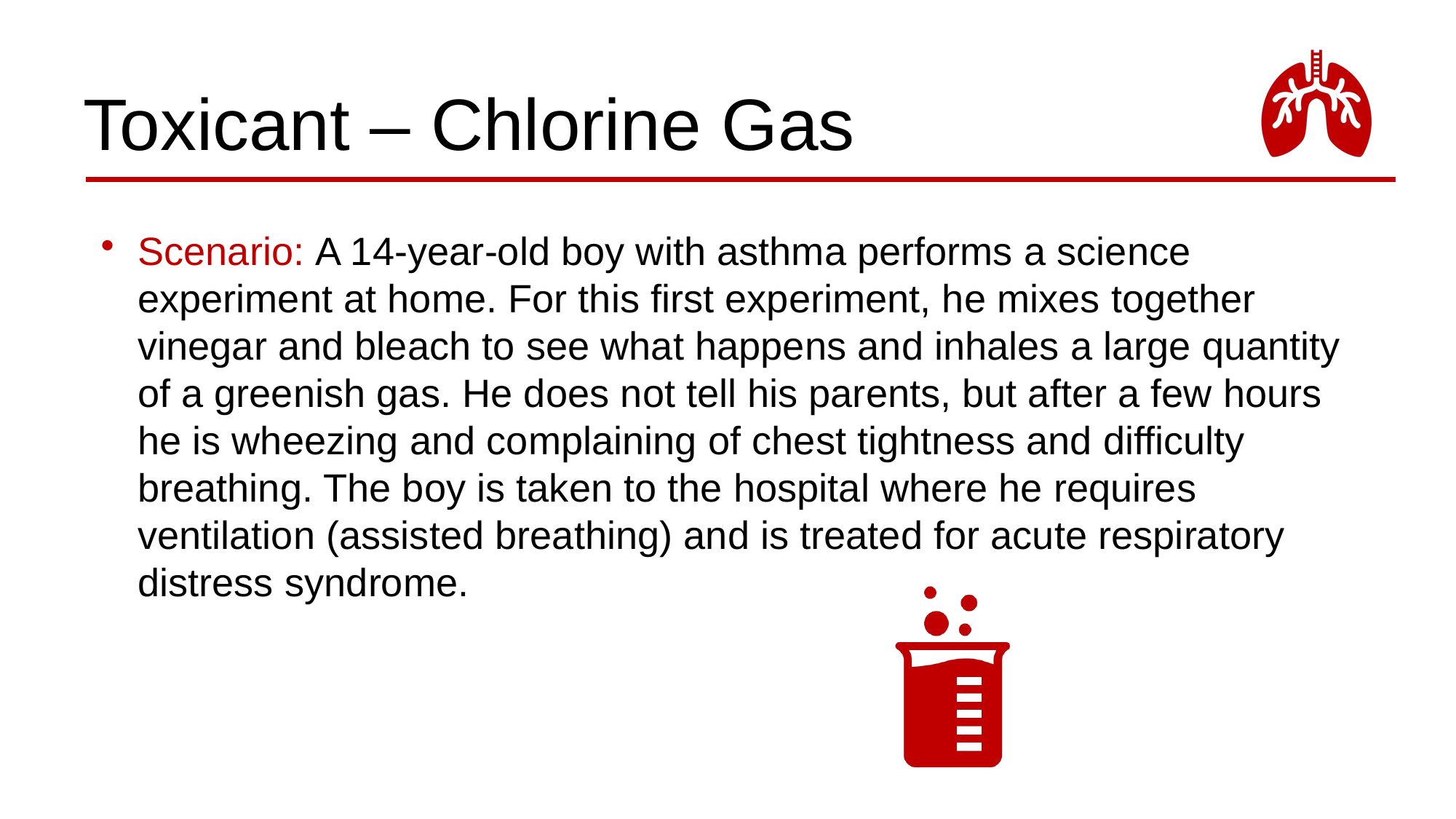

# Toxicant – Chlorine Gas
Scenario: A 14-year-old boy with asthma performs a science experiment at home. For this first experiment, he mixes together vinegar and bleach to see what happens and inhales a large quantity of a greenish gas. He does not tell his parents, but after a few hours he is wheezing and complaining of chest tightness and difficulty breathing. The boy is taken to the hospital where he requires ventilation (assisted breathing) and is treated for acute respiratory distress syndrome.

## Slide 13
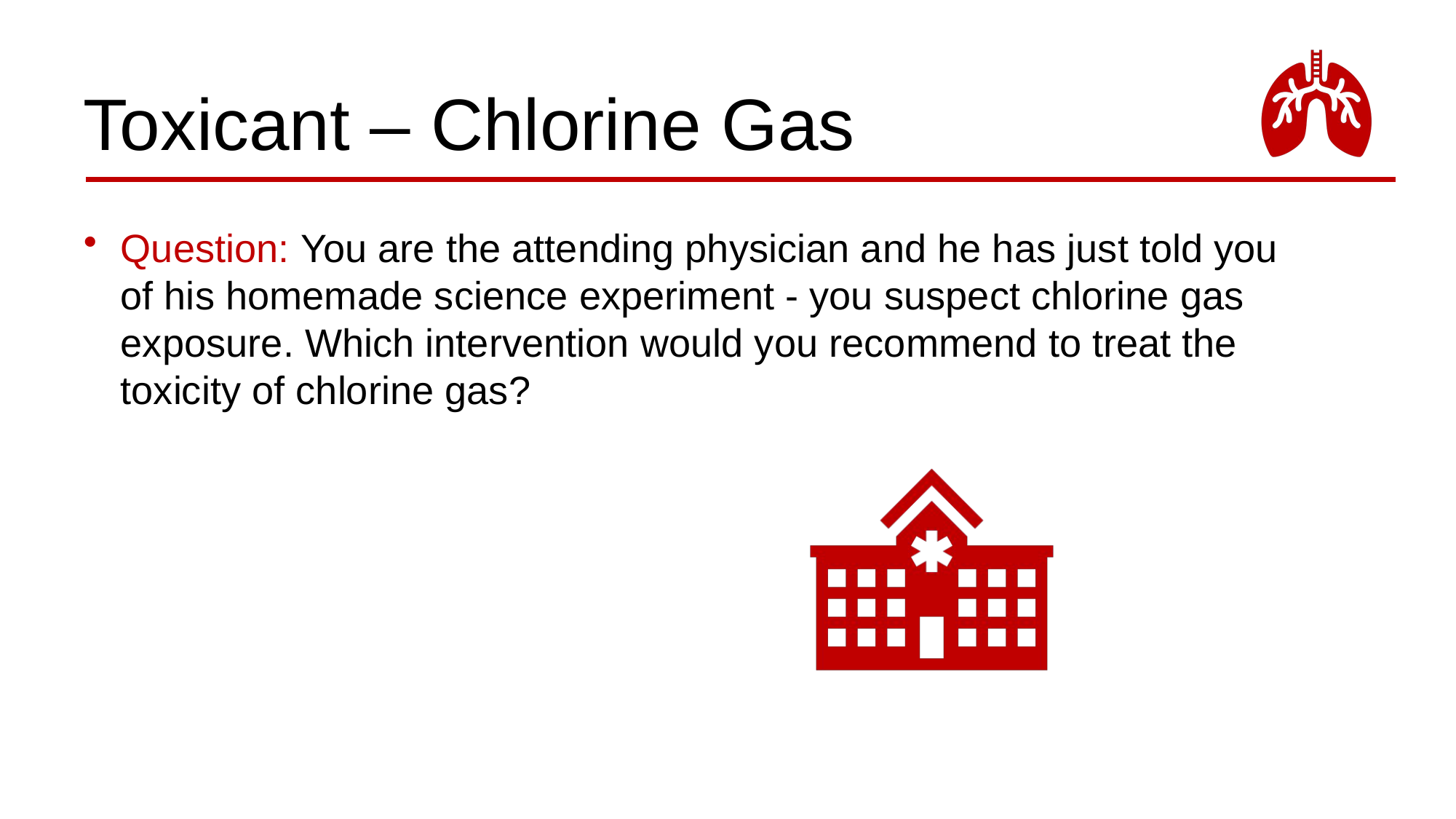

# Toxicant – Chlorine Gas
Question: You are the attending physician and he has just told you of his homemade science experiment - you suspect chlorine gas exposure. Which intervention would you recommend to treat the toxicity of chlorine gas?

## Slide 14
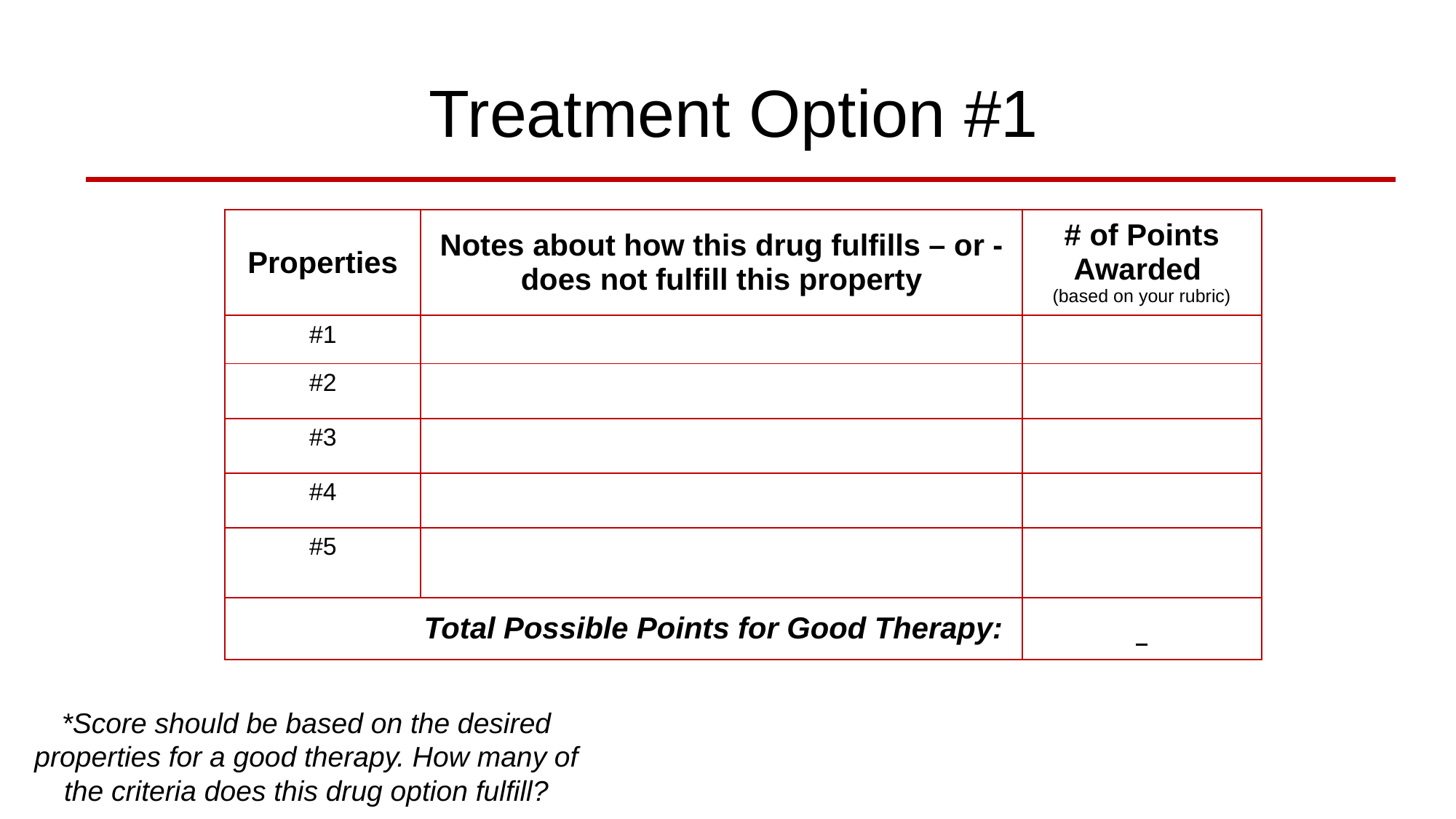

# Treatment Option #1
| Properties | Notes about how this drug fulfills – or -does not fulfill this property | # of Points Awarded (based on your rubric) |
| --- | --- | --- |
| #1 | | |
| #2 | | |
| #3 | | |
| #4 | | |
| #5 | | |
| Total Possible Points for Good Therapy: | | |
*Score should be based on the desired properties for a good therapy. How many of the criteria does this drug option fulfill?

## Slide 15
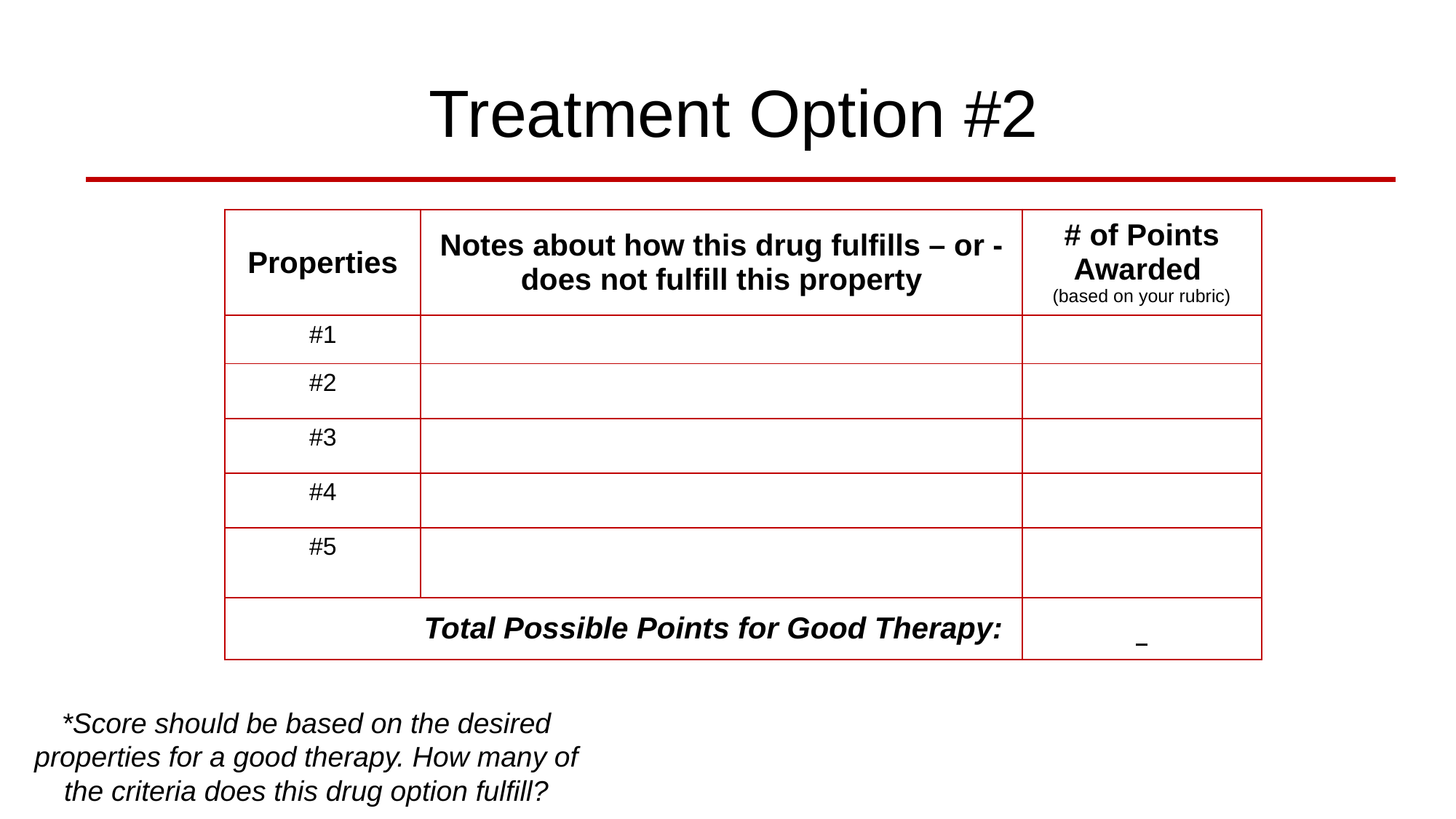

# Treatment Option #2
| Properties | Notes about how this drug fulfills – or -does not fulfill this property | # of Points Awarded (based on your rubric) |
| --- | --- | --- |
| #1 | | |
| #2 | | |
| #3 | | |
| #4 | | |
| #5 | | |
| Total Possible Points for Good Therapy: | | |
*Score should be based on the desired properties for a good therapy. How many of the criteria does this drug option fulfill?

## Slide 16
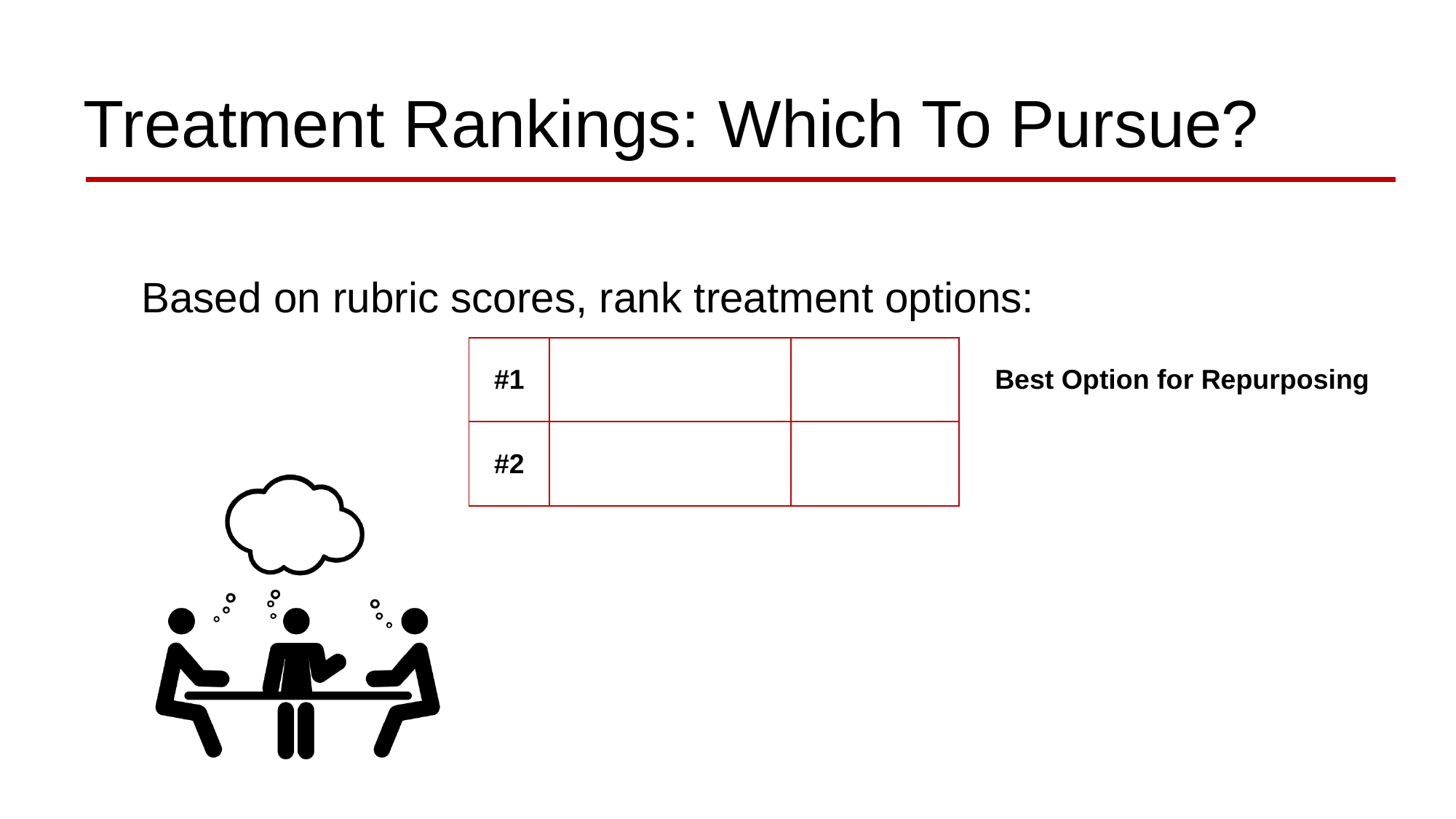

# Treatment Rankings: Which To Pursue?
Based on rubric scores, rank treatment options:
| #1 | | |
| --- | --- | --- |
| #2 | | |
Best Option for Repurposing

## Slide 17
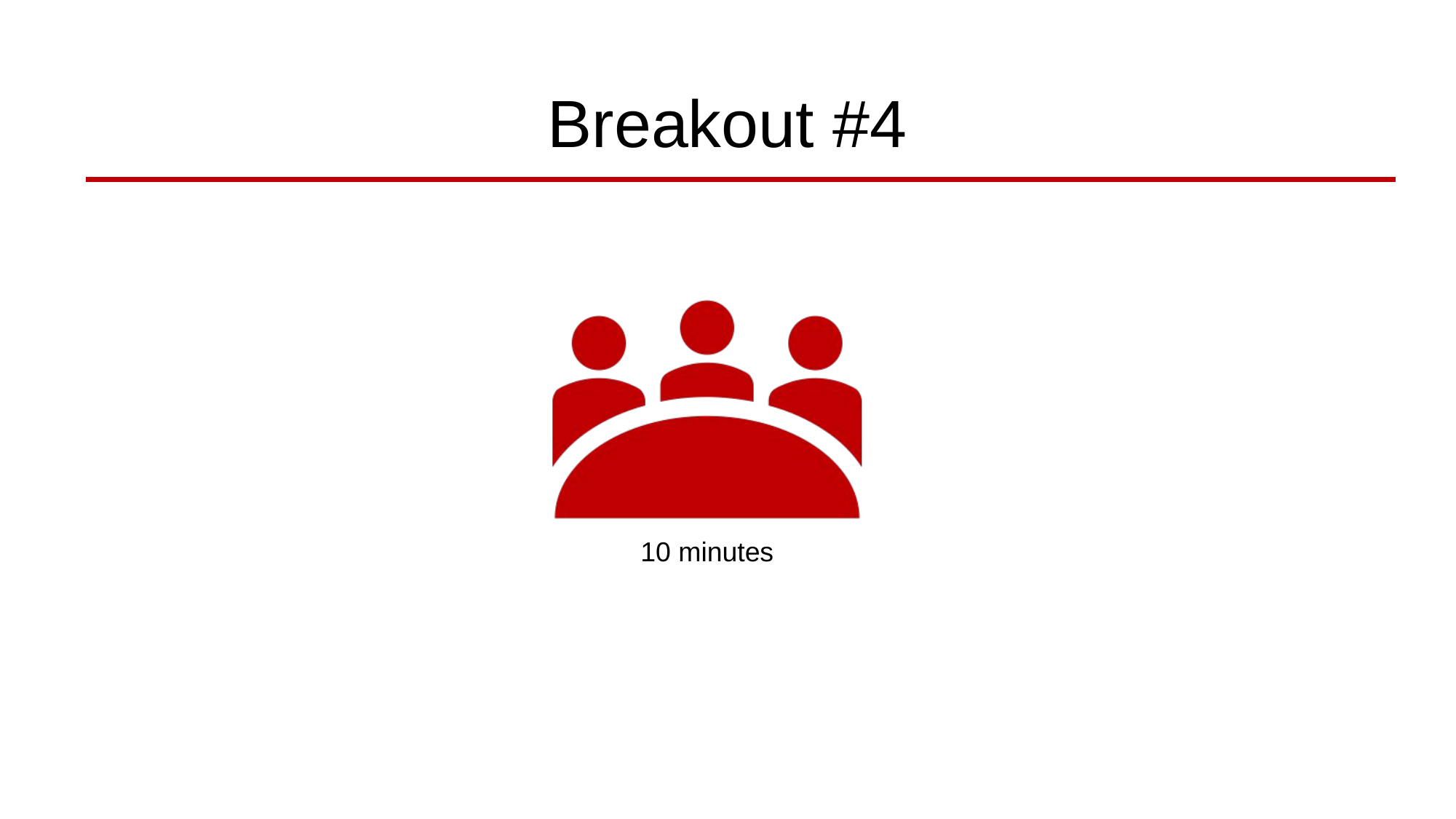

# Breakout #4
10 minutes

## Slide 18
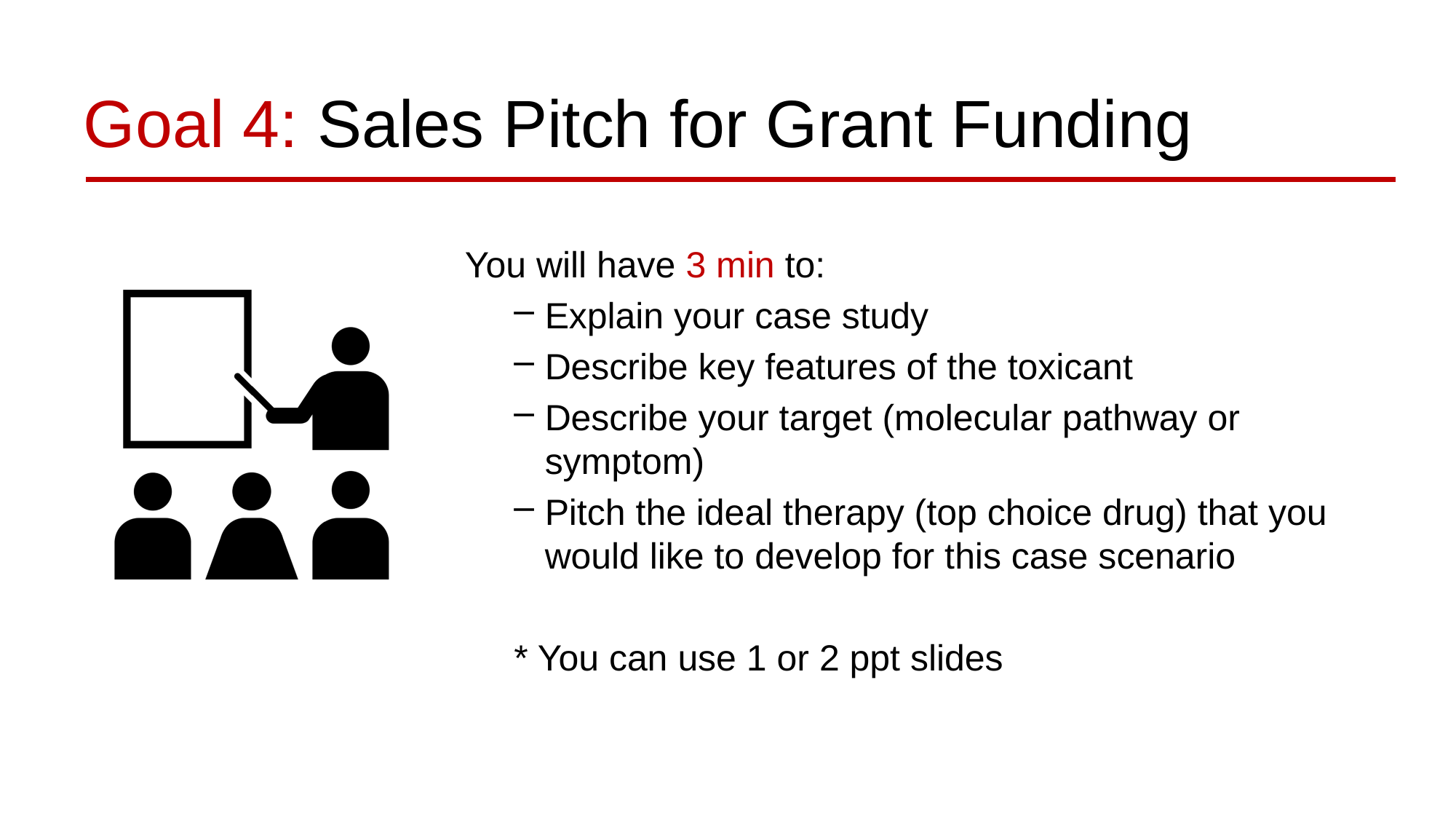

# Goal 4: Sales Pitch for Grant Funding
You will have 3 min to:
Explain your case study
Describe key features of the toxicant
Describe your target (molecular pathway or symptom)
Pitch the ideal therapy (top choice drug) that you would like to develop for this case scenario
* You can use 1 or 2 ppt slides
