# Supplementary material for: Repurposing FDA‐approved drugs to treat chemical weapon toxicities: Interactive case studies for trainees
Source: Pharmacol Res Perspect. 2024 Jul 4;12(4):e1229. doi: 10.1002/prp2.1229 (PMC11223991; doi:10.1002/prp2.1229)
Supplement: Supplementary file 4 — File S7. [file PRP2-12-e1229-s001.docx]

**Moderator Instructions**

Screen share the powerpoint slides from your computer and do *not* provide them a copy until the final breakout (if they want them for their sales pitch). At the bottom of this outline are the specific scenario and drugs for each case.

**Breakout session 1 (15 minutes):**

- Introductions of group members
-
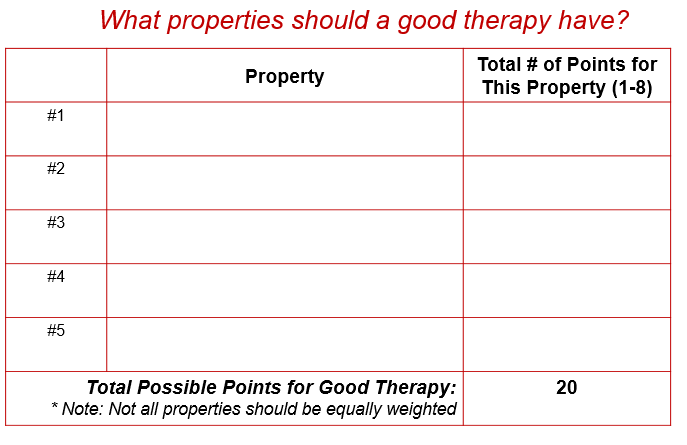
Students will spend about half the time identifying 4 to 5 important properties for their scoring rubric and weighting of points. They have 20 points to allocate.
- We will do a polling question about properties so the students should have some ideas but properties for you to encourage include *targeted mechanism of action* (rather than symptom relief if possible), *available route of administration*, *safety* of the drug, *storage conditions* for the drug, *cost* (injectables usually more expensive), *expertise required to administer* (tablets and patches and inhalers do not require much expertise; iv or im drugs require expertise to administer).
- Generally, mechanism of action should be weighed more than cost or expertise.
- You will be the note taker for your room and add their comments to the rubric. The team needs to select someone to present their rubric in the main room (you will need to screen share for them). They can edit their rubric after this breakout based on what they hear in the main room.

**Breakout session 2 (10 minutes):**

- Have your group read out loud about 1 or 2 of the drugs from your table. If questions about the drug, they can look up for more information online. There is nothing to report back in the main room.

**Return to main room:**

-
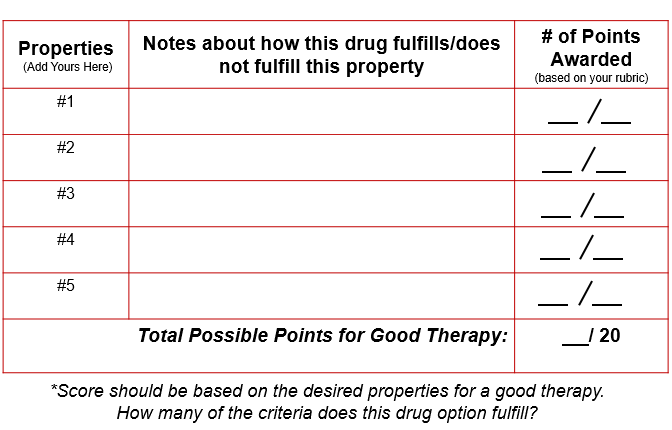
We will provide an example of what to do in the 3^rd^ breakout. Review a toxicant, scenario and rank potential drugs for therapies using our rubric.

**Breakout session 3 (20 minutes):**

- Have one volunteer read the background and mechanism of toxicity slide and a different volunteer read the scenario and question slides.
- Based on the information ask the group to nominate 2 or 3 of the drugs from your list to score using the rubric. You will take notes on the rubric for each of the drugs (copy the properties and # of points that they determined earlier). Ask the students how well that drug fulfills each ideal property and enter in the rubric. Add up the total number of points for each of the drugs. The one with the highest number of points should be the one they will go forward with.
- Your group will return to the main room. When prompted, please type in the team number, toxicant name, and drug that scored highest for repurposing.


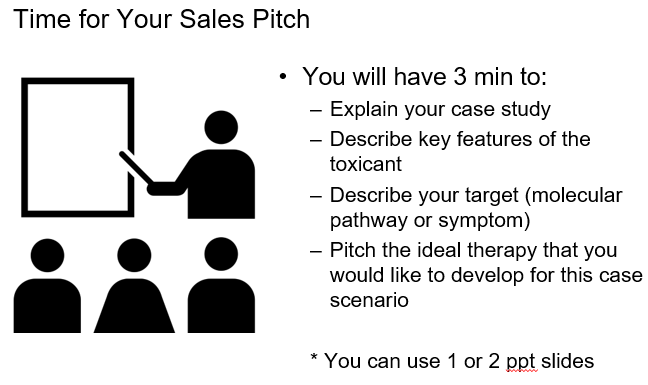
**Breakout session 4 (15 minutes): Moderator send out google link to work on a document at the same time.**

- - Your students should prepare a 3-minute sales pitch to earn a big NIH grant.
  - You can now upload a copy of the powerpoint you have been working from in the chat box for your room so they have copies of the materials
  - The goal is to pick the best compound and tell everyone why it’s the right choice for NIH to fund.

**Return to Main Room**

- Students will present their 3 minute pitches. All participants will vote on the best sales pitch.

| **Group** | **Toxicant** | **Moderator** |
| --- | --- | --- |
| 1 | Phosgene Oxime |  |
| 2 | TETS |  |
| 3 | Parathion |  |
| 4 | Chlorine Gas |  |

**Case Scenarios and Talking Points**

**Case 1: Phosgene Oxime**

Scenario: There are 12 Soldiers and 3 civilians in a building in Syria. A bomb goes off and with it the tendrils and vapors of an agent identified to be Phosgene Oxime penetrate the space. 5 Soldiers and 2 civilians start to immediately suffer from extreme pain and irritation, the others are further from the site and evacuate immediately, but still show signs of exposure.

Main things to consider for this scenario (just in case your students need some leading):

- You are on site, so you will be unable to easily do something that requires an IV or special storage
- Injury starts happening very quickly
- You can pick drugs that target inflammation, oxidative stress, and any of the symptoms possibly

Potential Drugs to pick:

- Diclofenac (target pain and inflammation)
- Dupilumab (target inflammation)
- Fexofenadine (target [histamine](https://www.guidetopharmacology.org/GRAC/FamilyDisplayForward?familyId=33) signaling from mast cells) ** Very good candidate
- Montelukast (block inflammation and used for urticaria) ** Pretty good candidate

**Case 2: TETS**

Scenario: There has been a string of neurotoxic poisonings in China. 13 people have been identified as patients who have suffered or died from exposure to unknown entity in the last 14hrs. Further investigation reveals that all patients use the same rural subway station which has recently experienced budget cuts and has been treating a rat infestation with TETS. Contamination was found in the fans of one waiting area and inhalation exposure is suspected.

It is a Sunday and few riders have entered the station, but you have identified 7 additional potential exposures, 4 of which present with convulsive status epilepticus (seizures), within the last 45 minutes and are able to get all to a hospital.

Main things to consider for this scenario (just in case your students need some leading):

- You are able to get all patients to a hospital and treat them at the full capacity of a hospital
- Exposure is by INHALATION
- GABA inhibition is the main mechanism and is the primary route for on-target treatment
- You can pick drugs that target GABA, convulsions, and energy levels

Potential Drugs to pick:

- Ketamine (increase [GABA_A_](https://www.guidetopharmacology.org/GRAC/FamilyDisplayForward?familyId=72) signaling)
- Midazolam (increase GABA_A_ signaling)
- Topiramate (increase GABA_A_ signaling)
- Maybe Zoplidem (not as specific for seizures)
- Definitely NOT baclofen (wrong GABA receptor)

**Case 3: Parathion**

Scenario: A 7-year-old girl and her father arrive at the ER. The child is having extreme respiratory complications (including shortness of breath, wheezing, and chest tightness) and shows early signs of imminent seizure. She is also sweating, teary-eyed, and has pinpoint pupils.

The father explains that they were in the lake and the child found an old black cylinder; the little girl pulled it out of the water and dropped it on a rock as she was walking on the shore. Powder, identified to be parathion, enters the child’s lung and contaminates her face. The child starts crying and the father rushes to pick her up and put her in his car and immediately drives her to the ER, leaving the canister behind.

Main things to consider for this scenario (just in case your students need some leading):

- You are treating in a hospital and have all the resources of the hospital at your disposal
- Exposure is most likely dermal, but could be inhaled or ingested (simply put they can be creative here)
- Parathion is very contaminating: Note that the dad will probably start showing symptoms and that the car is contaminated
- FOCUS: On the respiratory symptoms; respiratory failure is usually the primary cause of death
- Children typically have a more severe response
- This compound needs to be metabolized in order to be activated
- You can pick drugs that target cholinesterase, metabolism, etc.

Potential Drugs to pick:

- Pralidoxime and atropine (reactive [acetylcholinesterase](https://www.guidetopharmacology.org/GRAC/ObjectDisplayForward?objectId=2465&familyId=765&familyType=ENZYME) to breakdown excess acetylcholine)
- Possibly albuterol (to treat respiratory distress symptoms) – not likely as effective since not targeting acetylcholine
- Good to discuss the ability of using ritonavir very quickly after exposure since can prevent generation of oxon toxic metabolite (although probably to late to be an effective intervention in this scenario)

**Case 4: Chlorine Gas**

Scenario: A 14-year old boy with asthma performs a science experiment at home during quarantine. He mixes together HCl and bleach to see what happens and inhales a large quantity of a greenish gas. He does not tell his parents, but after a few hours of deteriorating is taken to the hospital. He requires ventilation and is treated for acute respiratory distress syndrome.

- Respiratory exposure is your primary concern
- A lot of time has passed after the kid is exposed; make sure to consider this during treatment
- You are in a hospital and have all the resources of the hospital at your disposal
- You can pick drugs that target inflammation, oxidative stress, acute respiratory distress syndrome – prevent long-term lung damage

Potential Drugs to pick:

- Dimethyl fumarate (improve antioxidant response)
- Dupilumab (block immune signaling)
- Methylprednisolone (block immune signaling)
- Nitric oxide (improve breathing)
- Less likely to use montelukast since not really mast cells involved (more lymphocytes)
